# Phosphine-catalyzed enantioselective [3 + 2] cycloadditions of γ-substituted allenoates with β-perfluoroalkyl enones†
†Electronic supplementary information (ESI) available: Experimental details, analytical data, NMR spectra of products. CCDC 1503840 (**3aa**). For ESI and crystallographic data in CIF or other electronic format see DOI: 10.1039/c7sc01432e



**DOI:** 10.1039/c7sc01432e

**Published:** 2017-04-19

**Authors:** Wei Zhou, Huamin Wang, Mengna Tao, Chao-Ze Zhu, Tao-Yan Lin, Junliang Zhang

**Affiliations:** a Shanghai Key Laboratory of Green Chemistry and Chemical Processes , School of Chemistry and Molecular Engineering , East China Normal University , Shanghai , 200062 , P. R. China . Email: jlzhang@chem.ecnu.edu.cn ; http://faculty.ecnu.edu.cn/s/1811/main.jspy

## Abstract

The enantioselective construction of densely functionalized cyclopentene bearing contiguous three stereocenters has been a challenging task in organic synthesis. Herein, we present a phoshine-catalyzed highly regio-, diastereo- and enantioselective [3 + 2] cycloaddition of γ-substituted allenoates with β-perfluoroalkyl enones, delivering a wide range of densely functionalized perfluoroalkylated cyclopentenes with three contiguous chiral stereocenters.

## Abstract 2

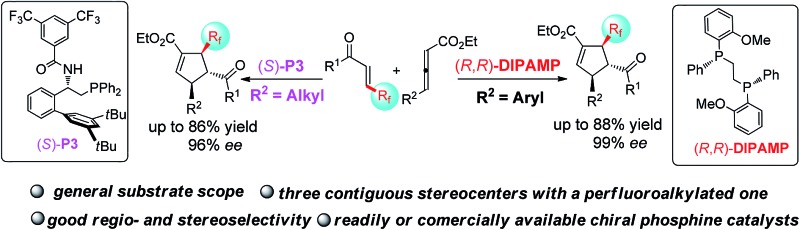
Herein we present a phoshine-catalyzed highly regio-, diastereo- and enantioselective [3 + 2] cycloaddition of γ-substituted allenoates with β-perfluoroalkyl enones, delivering a wide range of densely functionalized perfluoroalkylated cyclopentenes with three contiguous chiral stereocenters.

## Introduction

**Fig. 1:**
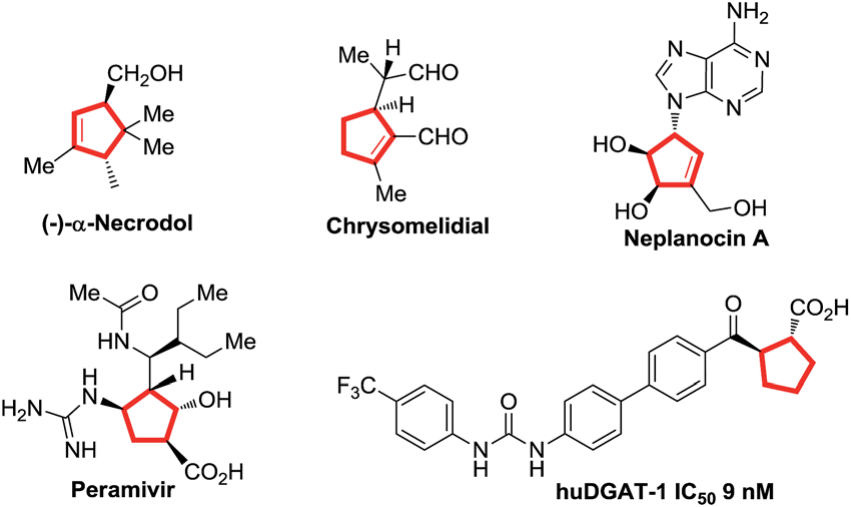
Selected natural products and pharmaceuticals contain cyclopentene or cyclopentane rings.

**Scheme 1:**
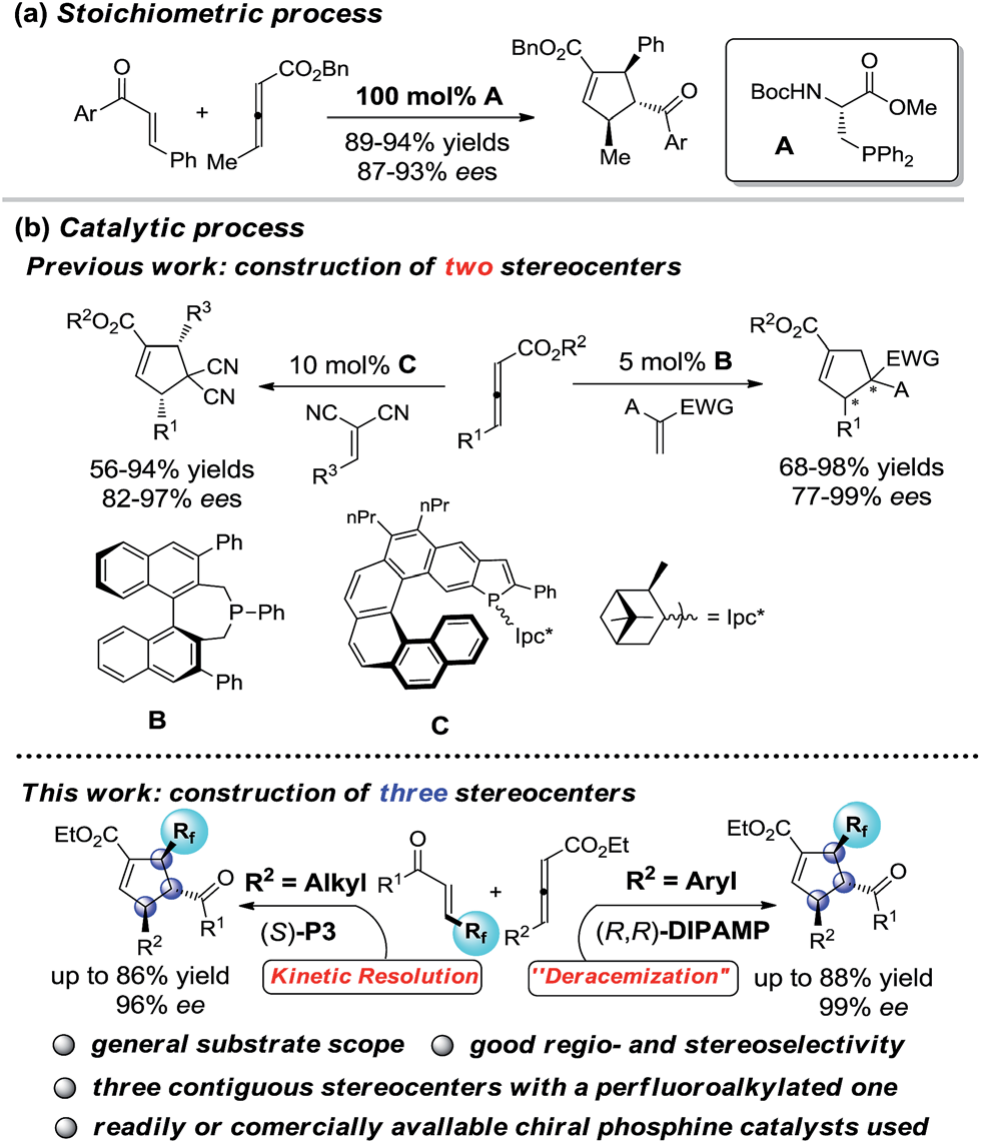
[3 + 2] cycloaddition reaction of γ-substituted allenoates and olefins.

Cyclopentenes (or cyclopentanes) are valuable skeletons found in several natural products and pharmaceuticals (Fig. 1).^1^ Among existing methodologies for their preparation, phosphine-catalyzed [3 + 2] cycloaddition of allenoates with electron-deficient olefins was first reported by Lu in 1995 as a powerful and straightforward strategy for the construction of functionalized cyclopentene rings.^2,3^ As a result of tremendous effort from numerous research groups, Lu's enantioselective [3 + 2] cycloaddition reaction of terminal allenoates with electron-deficient olefins has been well established over the past years.^4^ However, asymmetric [3 + 2] cycloaddition reaction of γ-substituted allenoates with electron-deficient olefins has been less explored despite the increase in stereochemical diversity of the cycloaddition products. In 2007, Miller's group first realized a unique “deracemization” reaction upon cycloaddition of chalcone with racemic γ-methyl allenoates but requisite the use of a stoichiometric amount of chiral phosphine catalyst **A** (Scheme 1a).^4*c*^ Subsequently, Fu and co-workers have accomplished the cycloaddition reaction of racemic γ-substituted allenoates with heteroatom-bearing olefins with the use of a catalytic amount of chiral phosphine **B**, furnishing a facile access to functionalized cyclopentenes with two adjacent stereo centers (Scheme 1b).^5^ Recently, Marinetti and coworkers have reported a highly enantioselective [3 + 2] cycloaddition of γ-substituted allenoates with benzylidenemalononitrile by utilizing chiral phosphahelicenes catalyst **C** (Scheme 1b).^6^

Despite this progress, the scope of γ-substituted allenoates and electron-deficient olefin partner for enantioselective Lu's annulation is very limited, and the construction of cyclopentene derivatives with three contiguous chiral stereocenters has been a major challenge but a highly desirable task. Moreover, introduction of perfluoroalkylated, particularly trifluoromethylated, stereocenters into chiral compounds have garnered special attention in pharmaceutical and pesticide industry since the polarity, bioavailability, metabolic stability and other properties of the parent molecules could be influenced greatly by these perfluoroalkyl groups.^7^ During the course of our continuous interest in design, synthesis and application of novel chiral β-aminephosphines^8,9^ in asymmetric catalysis and the synthesis of enantio-enriched trifluoromethylated building blocks,^8d,g^ we envisaged that the challenging enantioselective [3 + 2] cycloadditions of γ-substituted allenoates with β-perfluoralkyl α,β-enones might be addressed by systematic screening of known phosphines or rational design of new catalysts (Scheme 1b). In the present study, we report our efforts in addressing this challenging reaction by identifying two phosphine catalysts, commercially available bisphosphine (*R*,*R*)-**DIPAMP** and novel multifunctional (*S*)-**P3** which have been developed in our group. Further control experiments have shown that the reaction under the catalysis of (*R*,*R*)-**DIPAMP** was a deracemization process, while the kinetic resolution reaction was observed under the multifunctional phosphine catalyst (Scheme 1).

## Results and discussion

In order to validate the feasibility of the asymmetric [3 + 2] cycloaddition of γ-substituted allenoates with β-perfluoralkyl α,β-enones, allenoate **2a** and enone **1a** were exposed to a range of commercially available chiral bisphosphine catalysts (Table 1). A small amount of the desired **3aa** was observed when (*S*,*S*)-**DIOP** or (*R*,*R*)-**Et**-**DUPHOS** was utilized as the catalyst (Table 1, entries 1 and 2). Interestingly, (*R*,*R*)-**Et**-**BPE** exhibited a promising level of reactivity with 64% yield and stereoinduction with 39% ee (Table 1, entry 3). Fortunately, 86% yield of **3aa** with 89% ee was obtained using (*R*,*R*)-**DIPAMP** as a catalyst (Table 1, entry 4). It can be noted that multifunctional chiral phosphines (*S*)-**P1–P6** bearing hydrogen bond donors, such as amide and (thio) urea groups, could deliver higher chemical yield but with unacceptable enantioselectivity (Table 1, entries 5–10). Gratifyingly, the enantioselectivity was improved to 92%, albeit with a slightly lower yield when decreasing the reaction temperature from 25 °C to –20 °C (Table 1, entries 11–13). However, much lower reaction temperature was not beneficial for enantioselectivity and reactivity (Table 1, entry 14). In addition, much lower yield and enantioselectivity was observed when (*Z*)-**1a** was utilized in the reaction, indicating that the configuration of enone also affected the reaction significantly (Table 1, entry 15). Further screening of solvents demonstrated that toluene was the best reaction medium for this transformation (see ESI† for details). Then, the optimized reaction conditions were identified: 10 mol% (*R*,*R*)-**DIPAMP** as the catalyst and toluene as the reaction medium at –20 °C.

**Table 1:** Optimization of reaction conditions^*a*^

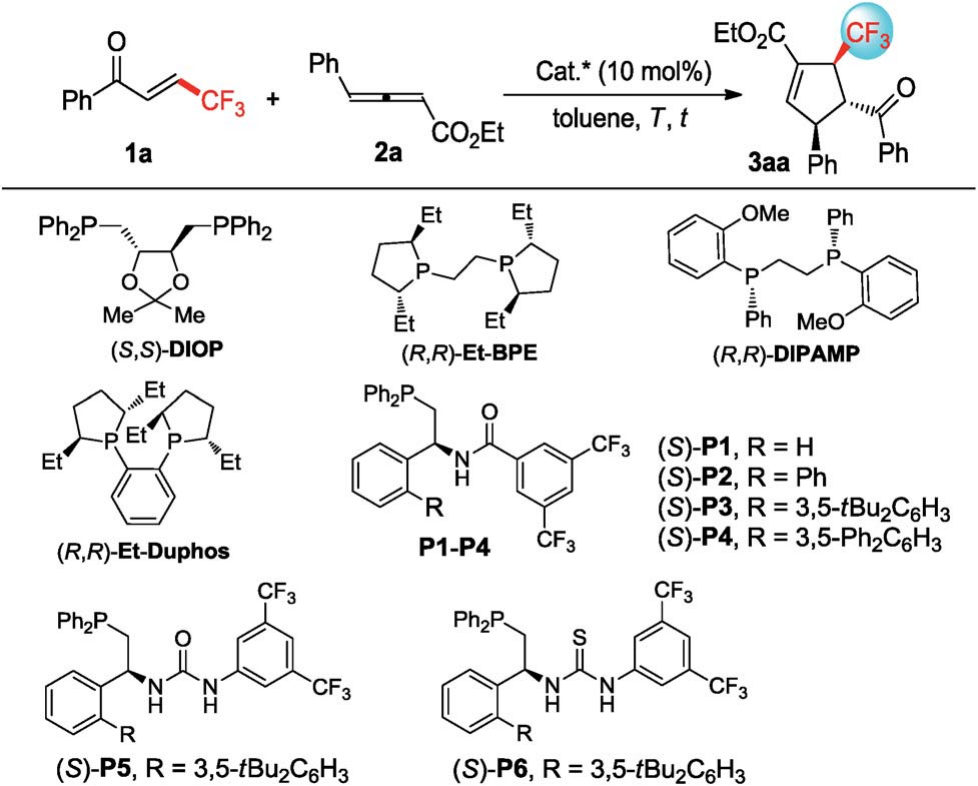					
Entry	Cat.	*T* (°C)	*t* (h)	Yield^*b*^ (%)	ee^*c*^ (%)
1	(*S*,*S*)-**DIOP**	25	12	<10	—
2	(*R*,*R*)-**Et**-**Duphos**	25	12	<10	—
3	(*R*,*R*)-**Et**-**BPE**	25	8	64	39
4	(*R*,*R*)-**DIPAMP**	25	6	81	89
5	(*S*)-**P1**	25	3	79	3
6	(*S*)-**P2**	25	3	85	14
7	(*S*)-**P3**	25	3	84	40
8	(*S*)-**P4**	25	3	86	21
9	(*S*)-**P5**	25	0.5	91	31
10	(*S*)-**P6**	25	0.5	88	38
11	(*R*,*R*)-**DIPAMP**	0	6	84	90
12	(*R*,*R*)-**DIPAMP**	–10	8	81	91
13	(*R*,*R*)-**DIPAMP**	–20	12	78	92
14	(*R*,*R*)-**DIPAMP**	–25	20	63	92
15^*d*^	(*R*,*R*)-**DIPAMP**	–20	24	23	50

^*a*^Unless otherwise specified, all reactions were carried out with (E)-**1a** (0.1 mmol), racemic **2a** (0.15 mmol) in toluene (1 mL).

^*b*^Yield of isolated products; d.r. and r.r. > 20 : 1.

^*c*^Determined by HPLC analysis.

^*d*^(Z)-**1a** was used.

**Scheme 2:**
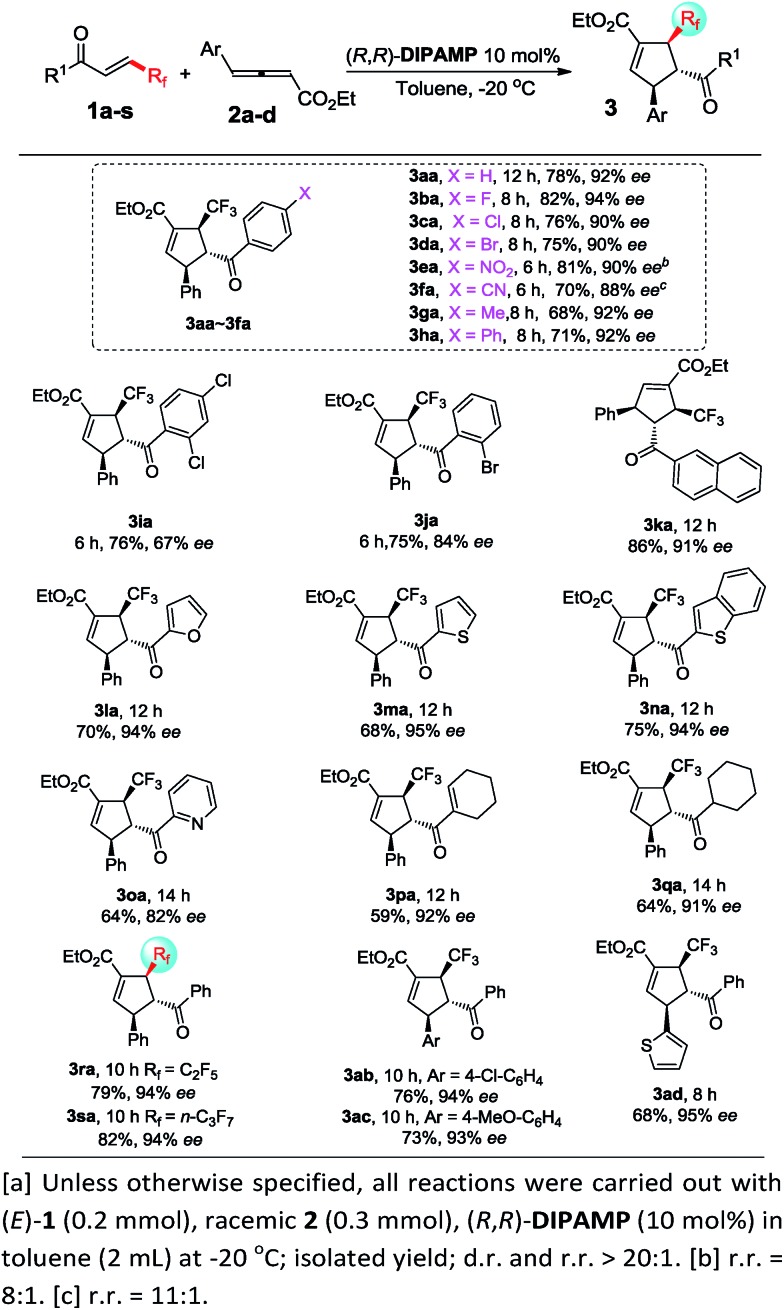
Enantioselective [3 + 2] cycloadditions of γ-aryl substituted allenoates with β-perfluoro substituted enone^*a*^.

Under optimal reaction conditions, we investigated the scope of the enantioselective [3 + 2] cycloaddition reaction (Scheme 2). Remarkably, a wide range of β-trifluoromethyl substituted enones containing different electron nature functional groups worked well with allenoate **2a**, thereby resulting in a highly regioselective α-addition products **3ba–3ha** in good yields with 88–94% ee. However, the introduction of an *ortho* substituent, such as Cl and Br, to the phenyl ring of enone led to dramatic decrease in the enantioselectivity (**3ia** and **3ja**). To our delight, naphthyl- and heteroaryl-containing substrates **1k–1o** were also compatible, efficiently furnishing a set of trifluoromethylated cyclopentenes containing naphthyl- and heteroaryl frameworks **3ka–3oa**. In addition, the present protocol could be readily extended to the challenging synthesis of cyclohexenyl and cyclohexyl based trifluoromethyl enone **1p** and **1q**. It was noteworthy that both β-pentafluoroethyl and β-heptafluoropropyl enone were particularly effective in the present transformation, forming valuable perfluoroalkyl substituted cyclopentene **3ra** and **3sa** in good yields with 94% ee. Furthermore, γ-aryl allenoates **2b–2d** with substituted aryl and hetereoaryl groups were well applicable and formed corresponding products **3ab–3ad** with high regioselectivity and enantioselectivity. The absolute configuration of product **3aa** was confirmed by single-crystal X-ray diffraction analysis.^10^

**Scheme 3:**
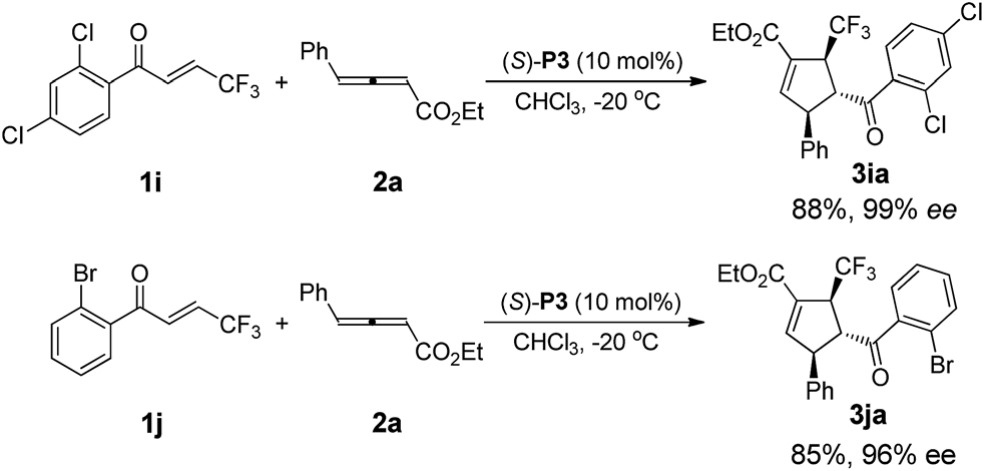
(S)-**P3** catalysed enantioselective [3 + 2] cycloadditions of **1i** and **1j** with **2a**.

After intensive screening of various chiral phosphine catalysts, it was found that multifunctional phosphine (*S*)-**P3** displayed good performance in the substrates with *ortho*-substituent, and the desired products **3ia** and **3ja** could be isolated in 85–88% yields with 96% and 99% ee, respectively (Scheme 3).

**Scheme 4:**
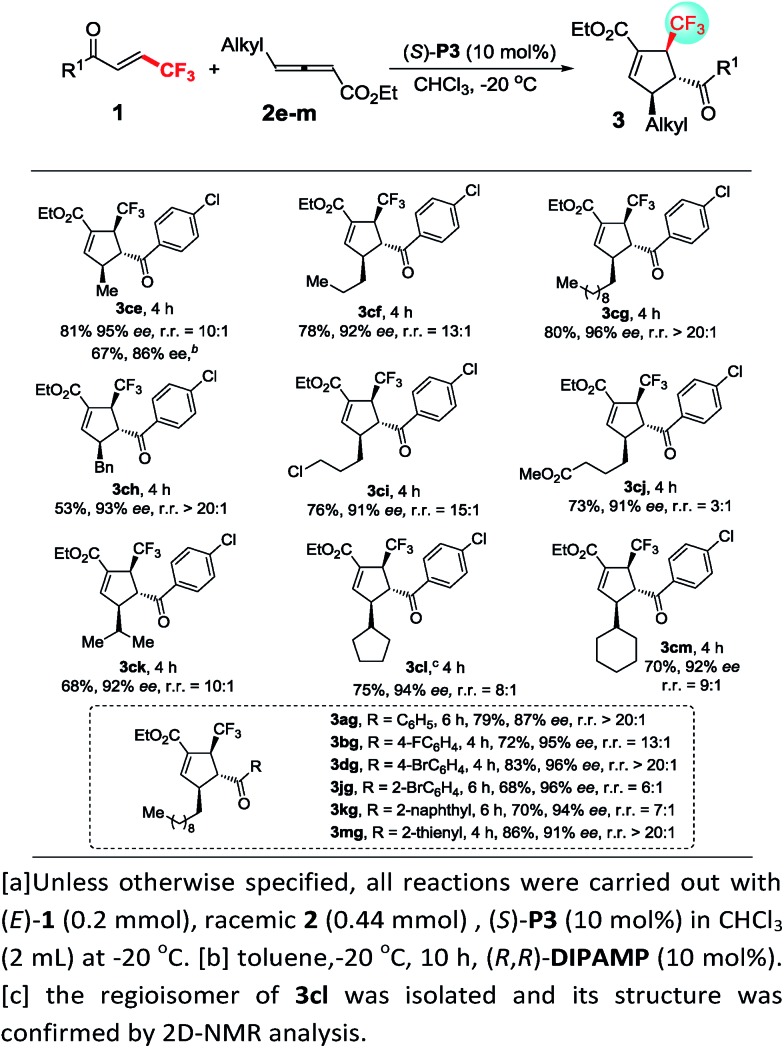
Enantioselective [3 + 2] cycloadditions of γ-alkyl substituted allenoates with β-perfluoro substituted enone^*a*^.

Unfortunately, the performance of (*R*,*R*)-**DIPAMP** in the cycloaddition of γ-alkyl substituted allenoates was not as good as that in the cases of γ-aryl substituted allenoates. For example, the reaction of **2e** with **1c** resulted in the formation of desired **3ce** in 67% yield but with only 86% ee. After further screening of a series of chiral phosphine catalysts, solvents and reaction temperature, it was found that (*S*)-**P3** was a privileged catalyst for cycloaddition of γ-alkyl allenoates. In general, allenoates **2e–2g** with different alkyl substituents at γ position participated in the annulation process with good regio- and enantioselectivity. In addition, diverse alkyl substituents such as benzyl, halogen and ester group were well tolerant, furnishing the corresponding cycloadducts **3ch–3cj** in moderate to good yields with high enantioselectivity. Furthermore, allenoates with bulky substituents such as isopropyl, cyclopentyl and cyclohexyl at γ position worked well, thereby forming the desired **3ck–3cm** in good yields with 92–94% ee. Good to excellent regioselectivity and enantioselectivity were also obtained in the cycloaddition reactions of allenoate **2g** with a wide range of β-trifluoromethyl substituted enones (Scheme 4).

Next, we turned our attention to gain insight into catalytic process for the proposed [3 + 2] cycloaddition reaction. In case of (*R*,*R*)-**DIPAMP** catalysed cycloaddition of **1d** and racemic **2a**, the starting material **2a** was recovered in 38% yield (based on **2a**) with 0% ee (eqn (1)). Furthermore, when optically active allenoate (+)-**2a** (76% ee) served as the substrate, ee of **3da** did not improve but the recovered (+)-**2a** had a higher ee (eqn (2)). These results have supported that a deracemization process was followed in the (*R*,*R*)-**DIPAMP** catalysed cycloaddition of **1d** and **2a**.1
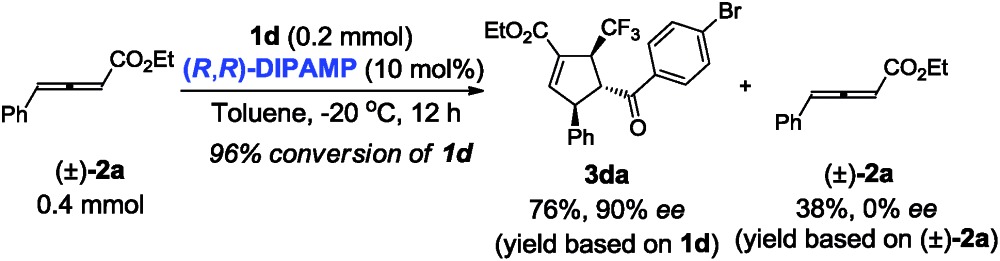

2
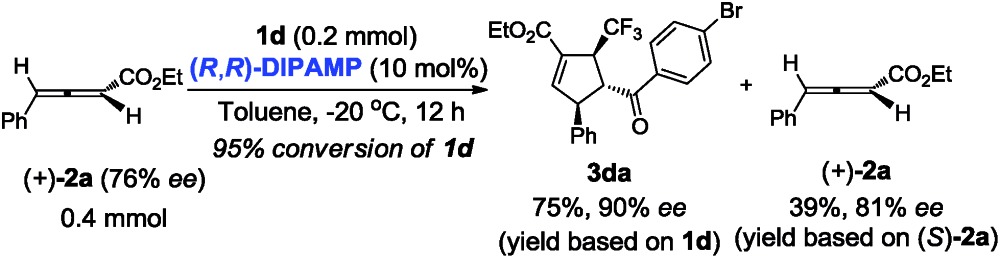



**Scheme 5:**
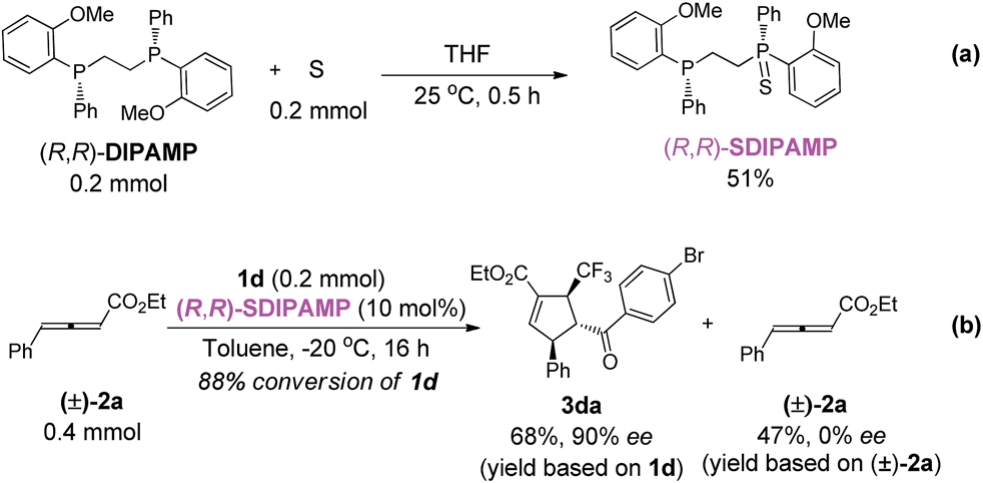
Synthesis of (*R*,*R*)-**SDIPAMP** and its application in the asymmetric [3 + 2] cycloaddition of **2a** and **1d**.

To examine both the phosphines in (*R*,*R*)-**DIPAMP** induce enantioselectivity independently or cooperatively, (*R*,*R*)-**SDIPAMP** that contained only one nucleophilic phosphine was synthesized and subjected to the reaction of **1d** and racemic **2a** (Scheme 5). Although the reaction became slower, enantio-selectivity of **3da** remained unchanged, demonstrating that both the phosphines in (*R*,*R*)-**DIPAMP** might induce enantioselectivity independently (Scheme 5b).

**Scheme 6:**
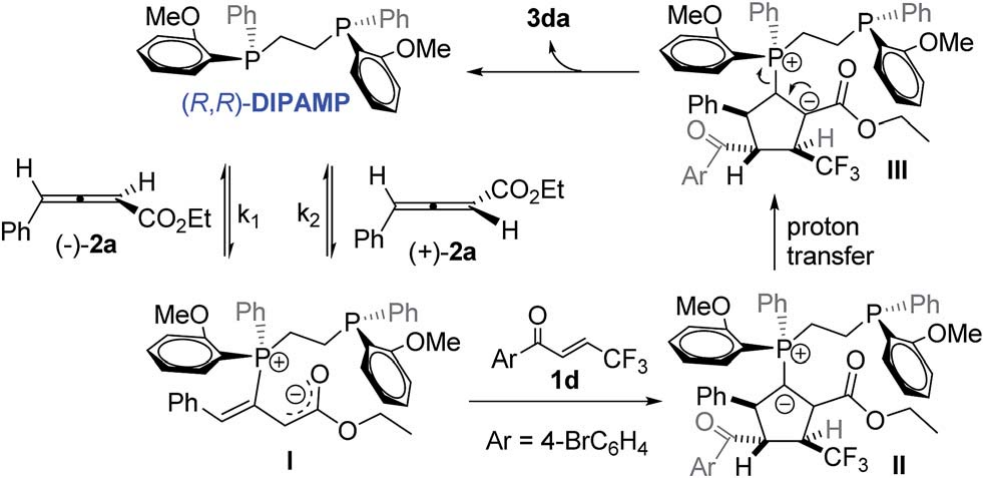
Possible catalytic cycle for (*R*,*R*)-**DIPAMP** catalysed asymmetric [3 + 2] cycloaddition.

Based on the abovementioned results and earlier reports,^11^ a plausible catalytic cycle for (*R*,*R*)-**DIPAMP** catalysed asymmetric [3 + 2] cycloaddition reaction of γ-aryl allenoates with trifluoromethyl enones has been illustrated in Scheme 6. The zwitterionic intermediate **I** was formed through nucleophilic addition of (*R*,*R*)-**DIPAMP** to racemic **2a**. The deracemization process resulted from the same nucleophilic attack rate (*K*_1_ = *K*_2_) of (*R*,*R*)-**DIPAMP** to both the enantiomers of allenoates **2a**. The subsequent [3 + 2] cycloaddition favoured α-addition to provide intermediate **II**, which then underwent proton transfer to provide intermediate **III**. Finally, (*R*,*R*)-**DIPAMP** and cyclopetene **3da** were released from intermediate **III**.

**Scheme 7:**
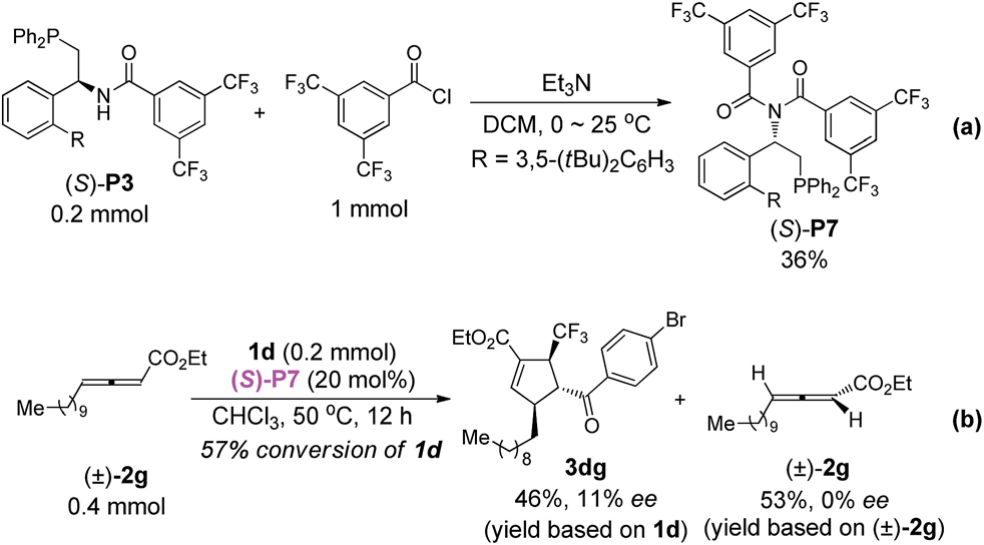
Synthesis of (*S*)-**P7** and its application in the asymmetric [3 + 2] cycloaddition of **2g** and **1d**.

In contrast to (*R*,*R*)-**DIPAMP**, a kinetic resolution reaction takes place with multifunctional chiral phosphine (*S*)-**P3** as the catalyst and (+)-**2a** ^12^ and (+)-**2g** ^13^ is recovered in 76% ee (in toluene, 77% ee in CHCl_3_) and 81% ee respectively (eqn (3) and (4)). In order to confirm the possible hydrogen-bonding interaction during the catalytic process, (*S*)-**P7** without hydrogen-bond donor was synthesized and subjected to the cycloaddition reaction (Scheme 7). The conversion decreased dramatically under higher catalyst loading and higher reaction temperature. The ee value of the recovered **2g** also vanished (Scheme 7b). These results demonstrated that the hydrogen-bond donor in (*S*)-**P3** was crucial for enantioselective formation of cycloaddition product *via* kinetic resolution process.3
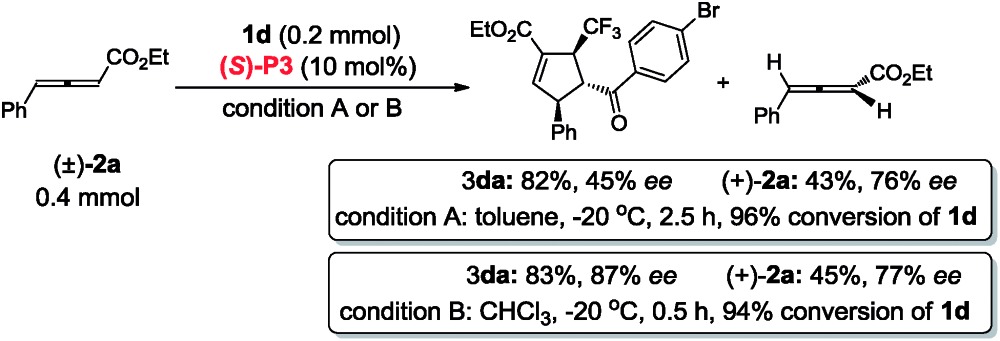

4
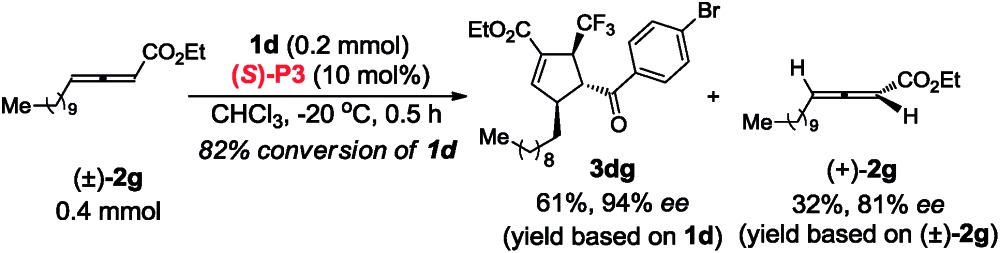



**Scheme 8:**
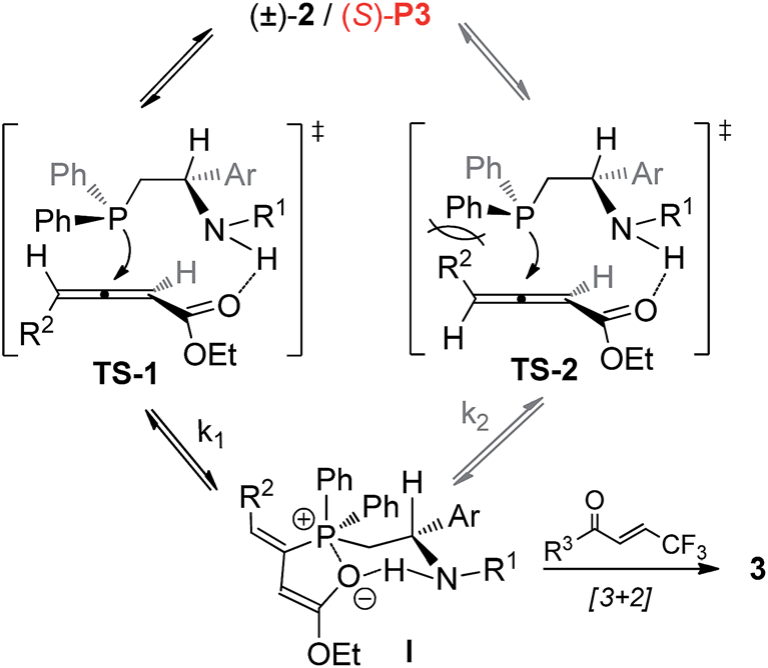
Possible catalytic cycle for (*S*)-**P3** catalysed asymmetric [3 + 2] cycloaddition reaction of racemic allenoate with trifluoro-methyl enone.

On the basis of above control experiments and recent excellent mechanistic studies^11^ on the [3 + 2] cycloaddition of allenoates with electron-deficient olefins, a tentatively proposed catalytic cycle for (*S*)-**P3** catalysed asymmetric [3 + 2] cycloaddition reaction of racemic allenoate with trifluoromethyl enone is shown in Scheme 8. (–)-**2** might prefer a configuration that would facilitate hydrogen-bonding interactions of N–H and carbonyl group (Scheme 8, **TS-1**). On the other hand, the nucleophilic attack of (*S*)-**P3** with (+)-**2** might be suppressed by the steric interaction of the bulky *R* ^2^ group with the phenyl moiety (Scheme 8, **TS-2**). Accordingly, different nucleophilic attack rates (*K*_1_ > *K*_2_) of (*S*)-**P3** to both the enantiomers of allenoates **2** contribute to the kinetic resolution process. It should be note that further experimental and theoretical studies are required to gain insights into kinetic resolution process.

## Conclusions

In conclusion, we have developed a highly regio-, diastereo- and enantioselective [3 + 2] cycloaddition of γ-substituted allenoates with β-perfluoroalkyl enones with (*R*,*R*)-**DIPAMP** or (*S*)-**P3** as a catalyst; it provides a facile access to a wide range of trifluoromethylated cyclopentenes with three contiguous chiral centers (up to 88% yield with 99% ee). In case of γ-aryl allenoate, commercially available chiral phosphine (*R*,*R*)-**DIPAMP** was identified as an efficient catalyst. In contrast, presently developed multifunctional phosphine (*S*)-**P3** has displayed high performance in the asymmetric cycloaddition of γ-alkyl allenoates with trifluoromethyl enones. In addition, control experiments have demonstrated that under the catalysis of (*R*,*R*)-**DIPAMP**, racemic allenoate reacted with trifluoromethyl enone through a “deracemization” process, whereas a clearly kinetic resolution reaction takes place with multifunctional chiral phosphine (*S*)-**P3** as a catalyst due to the hydrogen-bonding interaction between catalyst and the allenoate. Efforts toward other transformations of allenoate under the catalysis of our developed catalysts **P1–P6** are currently underway and will be reported in due course.

## References

[cit1] Meinwald J., Jones T. H. (1978). J. Am. Chem. Soc..

[cit2] Zhang C., Lu X. (1995). J. Org. Chem..

[cit3] Lu X., Zhang C., Xu Z. (2001). Acc. Chem. Res..

[cit4] Zhu G., Chen Z., Jiang Q., Xiao D., Cao P., Zhang X. (1997). J. Am. Chem. Soc..

[cit5] Fujiwara Y., Fu G. C. (2011). J. Am. Chem. Soc..

[cit6] Gicquel M., Zhang Y., Aillard P., Retailleau P., Voituriez A., Marinetti A. (2015). Angew. Chem., Int. Ed..

[cit7] Müller K., Faeh C., Diederich F. (2007). Science.

[cit8] Zhang Z.-M., Chen P., Li W., Niu Y., Zhao X.-L., Zhang J. (2014). Angew. Chem., Int. Ed..

[cit9] Wei Y., Shi M. (2010). Acc. Chem. Res..

[cit10] ESI.

[cit11] Dudding T., Kwon O., Mercier E. (2006). Org. Lett..

[cit12] The absolute configuration of (+)-**2a** were assigned by comparison with optical rotation in previous report: Inokuma T. Furukawa M. Uno T. Suzuki Y. Yoshida K. Yano Y. Matsuzaki K. Takemoto Y., Chem.–Eur. J., 2011, 17 , 10470 . 21812044 10.1002/chem.201101338

[cit13] The absolute configuration of (+)-**2g** were assigned by comparison with optical rotation in previous report: Li C.-Y. Sun X.-L. Jing Q. Tang Y., Chem. Commun., 2006 , 2980 .

