# Supplementary material for: Phosphine-catalyzed enantioselective [3 + 2] cycloadditions of γ-substituted allenoates with β-perfluoroalkyl enones
Source: Chem Sci. 2017 Apr 19;8(6):4660–5. doi: 10.1039/c7sc01432e (PMC5590097; doi:10.1039/c7sc01432e)
Supplement: Supplementary file 1 [file SC-008-C7SC01432E-s001.pdf]

Electronic Supplementary Material (ESI) for Chemical Science.
This journal is © The Royal Society of Chemistry 2017

Supporting Information

Table of Contents

1. General Information……………………………………….……………………………………….……….2

2. Optimization of Reaction Conditions for the Enantioselective [3+2] Cycloaddition of 1a and 2a………. 3

3. Typical Synthetic Procedure and Data for Novel Chiral Phosphines Catalyst……………………………4

4. Typical Procedure for the Enantioselective [3+2] Cycloaddition of Allenes with *β*–Perfluoroalkyl *α,β*–Enones…………………………………………………………………………………………………11

5. The Structural Assignment for the Regioisomer 3cl'……………………….………………………..12

6. Procedure for the "Deracemization" and Kinetic Resolution of Recemic Allenoates……………………16

7. X-ray Crystal Structure for 3aa……………………………………………………………………...18

8. General Data and HPLC Spectra for Cycloaddition Product 3……………………………………....19

9. References……………………………………………………………………………………………...49

10. ^{1}H, ^{13}C, ^{31}P and ^{19}F NMR Spectra…………………………………………………………………...50

1. General Information

Unless otherwise noted, all reactions were carried out under a nitrogen atmosphere; materials obtained from commercial suppliers were used directly without further purification. The $[\alpha]_D$ was recorded using PolAAr 3005 High Accuracy Polarimeter. ^{1}H NMR spectra, ^{13}C NMR spectra, ^{31}P NMR spectra and ^{19}F NMR spectra were recorded on a Bruker 400 (or 500) MHz spectrometer in chloroform-d_3. Chemical shifts (in ppm) were referenced to tetramethylsilane (δ = 0 ppm) in $CDCl_3$ as an internal standard. ^{13}C NMR spectra were obtained by using the same NMR spectrometers and were calibrated with $CDCl_3$ (δ = 77.00 ppm). The data is being reported as (s = singlet, d = doublet, dd = doublet of doublet, t = triplet, m = multiplet or unresolved, br = broad signal, coupling constant(s) in Hz, integration). Noteworthy, splitting signals between ^{13}C nucleus and ^{13}P nucleus in some chiral phosphine catalysts were difficult to distinguish and these ^{13}C NMR signals were reported as singlet entirely.

Trichloromethane ($CHCl_3$), dichloromethane, dichloroethane and ethyl acetate were freshly distilled from CaH_2; tetrahydrofuran (THF), toluene and ether were dried with sodium benzophenone and distilled before use.

Reactions were monitored by thin layer chromatography (TLC) using silicycle pre-coated silica gel plates. Flash column chromatography was performed on silica gel 60 (particle size 200-400 mesh ASTM, purchased from Yantai, China) and eluted with petroleum ether/ethyl acetate.

2. Optimization of Reaction Conditions for the Enantioselective [3+2] Cycloaddition of 1a and 2a[a]

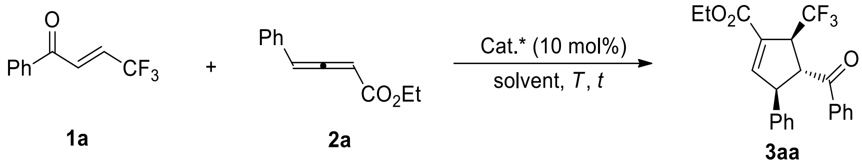

1a **2a** **3aa**

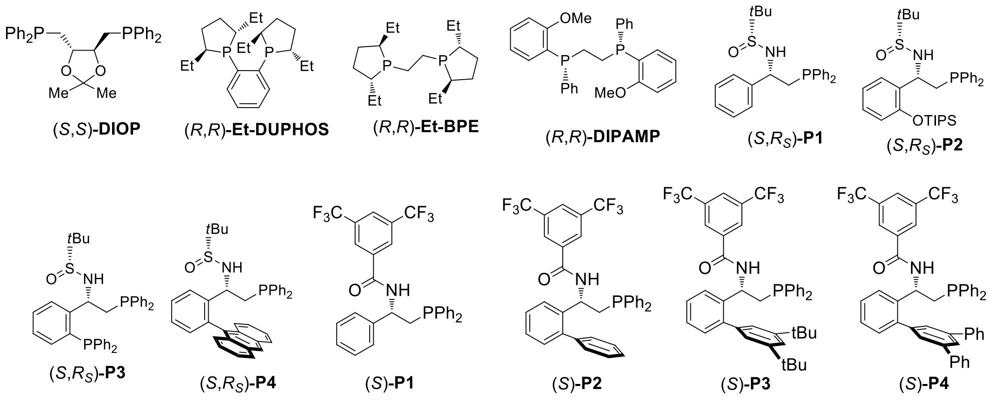

Entry	Cat.	*T* (°C)	*t* (h)	Solvent	Yield (%)[b]	*ee* (%)[c]
1	(*S*,*S*)-**DIOP**	25	12	toluene	<10	--
2	(*R*,*R*)-**Et-DUPHOS**	25	12	toluene	<10	--
3	(*R*,*R*)-**Et-BPE**	25	8	toluene	64	39
4	(*R*,*R*)-**DIPAMP**	25	6	toluene	81	89
5	(S,R_S)-**P1**	25	12	toluene	51	5
6	(S,R_S)-**P2**	25	12	toluene	54	11
7	(S,R_S)-**P3**	25	12	toluene	62	30
8	(S,R_S)-**P4**	25	12	toluene	58	29
9	(*S*)-**P1**	25	3	toluene	79	3
10	(*S*)-**P2**	25	3	toluene	85	14
11	(*S*)-**P3**	25	3	toluene	84	40
12	(*S*)-**P4**	25	3	toluene	86	21
13	(*R*,*R*)-**DIPAMP**	25	6	DCM	51	72
14	(*R*,*R*)-**DIPAMP**	25	6	$CHCl_3$	68	84
15	(*R*,*R*)-**DIPAMP**	25	6	EA	79	86
16	(*R*,*R*)-**DIPAMP**	25	6	acetone	62	26
17	(*R*,*R*)-**DIPAMP**	25	6	Et_2O	74	87
18	(*R*,*R*)-**DIPAMP**	25	6	MTBE	73	88
19	(*R*,*R*)-**DIPAMP**	25	6	THF	60	86
20	(*R*,*R*)-**DIPAMP**	25	6	CH_3CN	49	32
21	(*R*,*R*)-**DIPAMP**	0	6	toluene	84	90
22	(*R*,*R*)-**DIPAMP**	-10	8	toluene	81	91
23	(*R*,*R*)-**DIPAMP**	-20	12	toluene	78	92
24	(*R*,*R*)-**DIPAMP**	-25	20	toluene	63	92

[a] Unless otherwise specified, all reactions were carried out with (*E*)-**1a** (0.1 mmol), recemic **2a** (0.15 mmol) in solvent (1 mL). [b] Yield of isolated products; both diastereoselectivity and regioselectivity were more than 20:1. [c] Determined by HPLC analysis using a chiral stationary phase.

3. Typical Synthetic Procedure and Data for Novel Chiral Phosphines Catalyst

3.1 Typical Procedure for the Synthesis of Phosphines (*S*)-P1~4

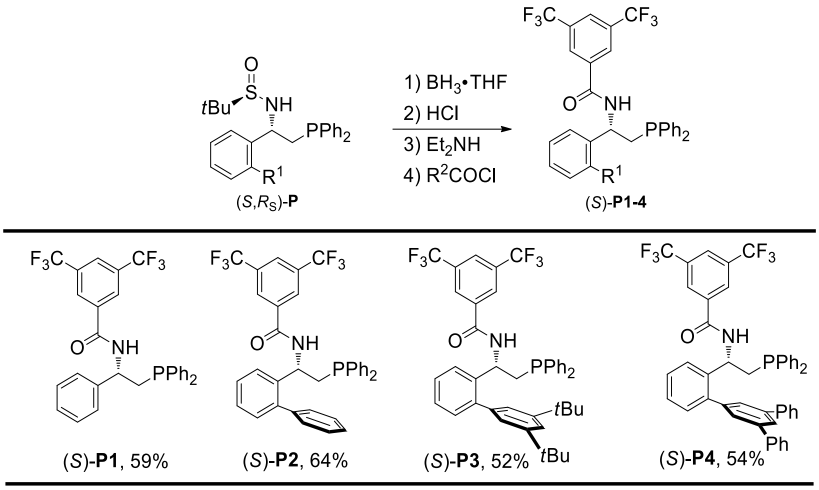

Step 1: BH_3•THF (3.0 mmol) was added slowly to the solution of (*S*,*R*$_S$)-**P** (2.0 mmol)[1] in dry THF (5 mL) at – 30 °C and the reaction mixture was stirred for 2 h until completion of the material as indicated by TLC followed by adding 10 mL of water and 20 mL EtOAc. The aqueous phase was separated and extracted three times with 20 mL EtOAc. The combined organic phases were dried over $MgSO_4$ and the solvents were removed in vacuo.

Step 2: 4 M HCl (1 mL) was added slowly to the above residue which disolved in MeOH (10 mL) and the reaction mixture was stirred at room temperature for 3 h until completion of material as indicated by TLC analysis, followed by washing with aq $NaHCO_3$ and 10 mL aq brine water. The organic layers was separated and extracted three times with 20 mL EtOAc. The combined organic phases were dried over $MgSO_4$ and the solvents were removed in vacuo.

Step 3: Et_2NH (5.0 mL) was added to the above residue and the mixture was stirred at 55 °C for 6 h under the protection of N_2 until completion of material as indicated by TLC analysis. The solvent was then removed in vacuo and the residue was used directly for the next step.

Step 4: Under the protection of Ar, 3,5-bis(trifluoromethyl)benzoyl chloride (1.1 eq.) was added slowly to the above residue which was disolved in dry DCM (0.1 M) at 0 °C. This reaction mixture was then stirred at 25 °C for another 1 h, after completion of the reaction, the solvent was then removed in vacuo and the residue was directly purified by silica gel chromatography using petroleum ether/EtOAc as the eluent to afford the desired (*S*)-**P1~4**.

3.2 Typical Procedure for the Synthesis of Phosphines (*S*)-P5 and (*S*)-P6

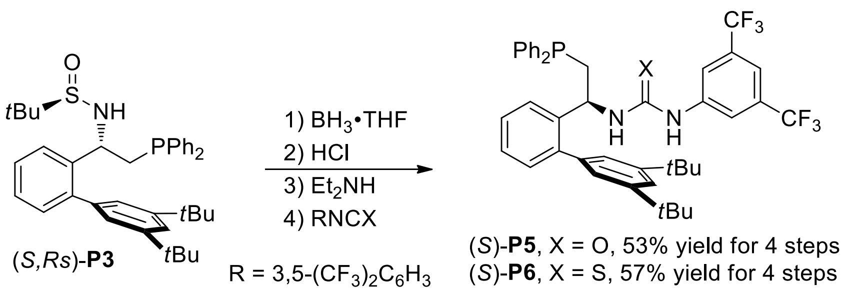

Step 1: BH_3•THF (3.0 mmol) was added slowly to the solution of (*S*,*R_S*)-**P3** (2.0 mmol)[1] in dry THF (5 mL) at –30 °C and the reaction mixture was stirred for 2 h until completion of the material as indicated by TLC followed by adding 10 mL of water and 20 mL of EtOAc. The aqueous phase was separated and extracted three times with 20 mL EtOAc. The combined organic phases were dried over $MgSO_4$ and the solvents were removed in vacuo.

Step 2: 4 M HCl (1 mL) was added slowly to the above residue which disolved in MeOH (10 mL) and the reaction mixture was stirred at room temperature for 3 h until completion of material as indicated by TLC analysis, followed by washing with aq $NaHCO_3$ and 10 mL of aq brine water. The organic layers was separated and extracted three times with 20 mL of EtOAc. The combined organic phases were dried over $MgSO_4$ and the solvents were removed in vacuo.

Step 3: Et_2NH (5.0 mL) was added to the above residue and the mixture was stirred at 55 °C for 6 h under the protection of N_2 until completion of material as indicated by TLC analysis. The solvent was then removed in vacuo and the residue was used directly for the next step.

Step 4: Under the protection of Ar, 3,5-Bis(trifluoromethyl)phenylisocyanate or 3,5-Bis(trifluoromethyl)phenyl isothiocyanate (1.2 eq.) was added slowly to the above residue which was disolved in dry DCM (0.1 M) at 0 °C. This reaction mixture was then stirred at 25 °C for another 1 h, after completion of the reaction, the solvent was then removed in vacuo and the residue was directly purified by silica gel chromatography using petroleum ether/EtOAc as the eluent to afford the desired (*S*)-**P5** and (*S*)-**P6**.

3.3 Typical Procedure for the Synthesis of Phosphines (*S*)-P7

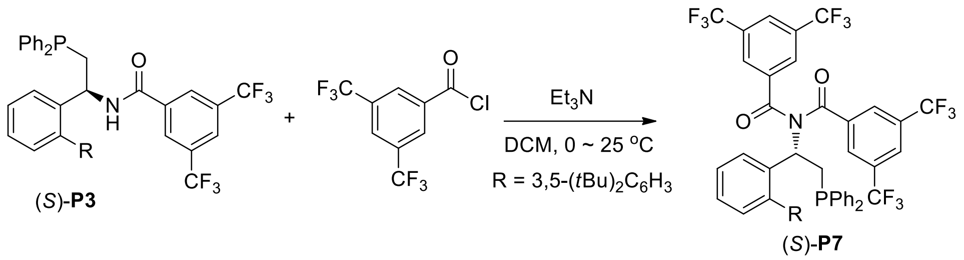

Under the protection of Ar, 3,5-bis(trifluoromethyl)benzoyl chloride (2 eq.) was added slowly to the solution of (*S*)-**P3** (0.2 mmol) in dry DCM (2 mL) at 0 °C and the reaction mixture was stirred for 1 h at this temperature. This reaction mixture was then stirred at 25 °C for another 4 h, until completion of the material as indicated by TLC followed by adding 5 mL of water and 10 mL of EtOAc. The aqueous phase was separated and extracted three times with 15 mL of EtOAc. The combined organic phases were dried over $MgSO_4$ and the solvents were removed in vacuo. The residue was directly purified by silica gel chromatography using petroleum ether/EtOAc as the eluent to afford the desired (*S*)-**P7** (36% yield).

3.4 Typical Procedure for the Synthesis of Phosphines (*R*,*R*)-SDIPAMP

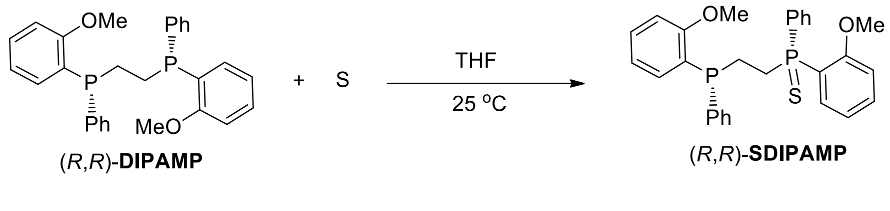

Under the protection of Ar, (*R*,*R*)-**DIPAMP** (0.2 mmol) was added to the solution of

sulfur (0.2 mmol) in dry THF (2 mL) at 25 °C and the reaction mixture was stirred for 0.5 h at this temperature. After completion of the material as indicated by TLC, the solvent was then removed in vacuo and the residue was directly purified by silica gel chromatography using petroleum ether/EtOAc as the eluent to afford the desired (*R*,*R*)-**SDIPAMP** (51% yield).

General Data for (*S*)-P1~7 and (*R,R*)-SDIPAMP

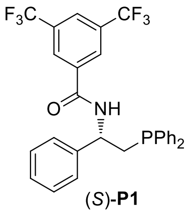

(*S*)-**P1**

(*S*)-**P1**; white solid; $[\alpha]_D^{20}$ = + 15.0 (*c* = 0.33, $CHCl_3$); ^{1}H NMR (500 MHz, $CDCl_3$): δ 8.01 (s, 2H), 7.97 (s, 1H), 7.55–7.51 (m, 2H), 7.45–7.42 (m, 2H), 7.35–7.34 (m, 9H), 7.30–7.26 (m, 1H), 6.73 (d, *J* = 7.5 Hz, 1H), 5.42–5.36 (m, 1H), 2.90–2.85 (m, 1H), 2.73–2.69 (m, 1H); ^{13}C NMR (125 MHz, $CDCl_3$): δ 163.62, 141.94 (d, *J* = 6.50 Hz), 137.78 (d, *J* = 12.13 Hz), 136.16, 132.98 (d, *J* = 19.38 Hz), 132.63 (d, *J* = 19.0 Hz), 131.94 (q, *J* = 33.63 Hz), 130.49 (q, *J* = 5.63 Hz), 129.25, 128.98, 128.88, 128.81, 128.75, 128.72, 128.67, 127.90, 127.28 (d, *J* = 2.88 Hz), 126.40, 125.85, 124.98–124.93 (m), 122.87 (q, *J* = 271.25 Hz), 53.00 (d, *J* = 17.13 Hz), 35.91 (d, *J* = 16.38 Hz); ^{31}P NMR (121.5 MHz, $CDCl_3$) δ = -22.82 ppm; HRMS (ESI) m/z calcd. for $C_{29}H_{22}F_6NNaOP$ $[M+Na]^+$ = 568.1258, found = 568.1235.

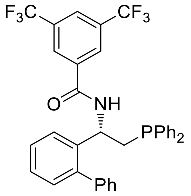

(*S*)-**P2**

(*S*)-**P2**; white solid; $[\alpha]_D^{20}$ = -43.0 (*c* = 0.33, $CHCl_3$); ^{1}H NMR (500 MHz, $CDCl_3$): δ 8.08 (s, 2H), 8.02 (s, 1H), 7.52–7.50 (m, 1H), 7.44–7.36 (m, 9H), 7.35–7.25 (m, 7H), 7.21–7.17 (m, 2H), 6.82 (d, *J* = 7.0 Hz, 1H), 5.62–5.57 (m, 1H), 2.69–2.63 (m, 1H), 2.57–2.53 (m, 1H); ^{13}C NMR (125 MHz, $CDCl_3$): δ 163.27, 140.89, 140.62, 139.90 (d, *J* = 6.38 Hz), 137.39 (d, *J* = 11.75, Hz), 136.85 (d, *J* = 12.13 Hz), 136.10, 132.62 (d, *J*

= 19.38 Hz), 132.32 (d, J = 19.0 Hz), 131.88 (q, J = 33.5 Hz), 130.79, 129.24, 128.95, 128.75, 128.62 (d, J = 1.38 Hz), 128.56 (d, J = 1.50 Hz), 128.39, 127.95, 127.33 (d, J = 2.25 Hz), 127.23 (d, J = 2.63 Hz), 125.52, 124.88–124.83 (m), 122.83 (q, J = 271.25 Hz), 50.70 (d, J = 15.0 Hz), 36.21 (d, J = 17.13 Hz); ^{31}P NMR (121.5 MHz, $CDCl_3$) δ = -23.13 ppm; HRMS (ESI) m/z calcd. for $C_{35}H_{26}F_6NNaOP$ [M+Na] $^+$ = 644.1548, found = 644.1540.

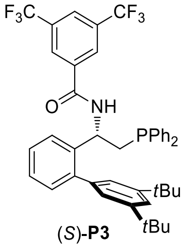

(*S*)-**P3**

(*S*)-**P3**; white solid; $[\alpha]_D^{20}$ = -34.7 (c = 0.33, $CHCl_3$); ^{1}H NMR (400 MHz, $CDCl_3$): δ 7.96 (s, 3H), 7.46–7.16 (m, 15H), 7.09–7.06 (m, 2H), 6.45–6.46 (m, 1H), 5.65–5.58 (m, 1H), 2.54–2.44 (m, 2H), 1.30 (s, 18H); ^{13}C NMR (100 MHz, $CDCl_3$): δ 163.29, 150.87, 142.00, 140.05, 139.95, 137.85 (d, J = 11.9 Hz), 136.99 (d, J = 11.90 Hz), 136.53, 132.75, 132.56, 132.36, 132.17, 131.82, 131.49, 130.97, 128.99, 128.71, 128.67, 128.64, 128.60, 128.57, 127.72, 127.33, 127.22, 125.21, 124.87, 124.23, 123.65, 121.52, 121.05, 50.86 (d, J = 15.60 Hz), 36.42 (d, J = 16.70 Hz), 34.91, 31.44; ^{31}P NMR (121.5 MHz, $CDCl_3$) δ = -62.72 ppm; HRMS (ESI) m/z calcd. for $C_{44}H_{43}F_6NOP$ [M+H] $^+$ = 734.2981, found = 734.2998.

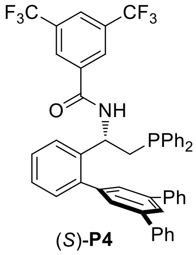

(*S*)-**P4**

(*S*)-**P4**; white solid; $[\alpha]_D^{20}$ = -41.6 (c = 0.33, $CHCl_3$); ^{1}H NMR (400 MHz, $CDCl_3$): δ 7.96 (s, 2H), 7.88 (s, 1H), 7.82–7.81 (m, 1H), 7.61–7.59 (m, 6H), 7.48–7.46 (m, 1H), 7.41–7.27 (m, 11H), 7.17–7.03 (m, 8H), 6.72 (d, J = 6.8 Hz, 1H), 5.64–5.57 (m, 1H), 2.68–2.62 (m, 1H), 2.57–2.52 (m, 1H); ^{13}C NMR (100 MHz, $CDCl_3$): δ 163.32, 141.74, 141.67, 140.81, 140.52, 139.99 (d, J = 6.40 Hz), 137.23 (d, J = 12.0 Hz), 136.89 (d, J = 12.30 Hz), 136.25, 132.58 (d, J = 19.40 Hz), 132.26 (d, J = 19.20 Hz),

131.88 (q, J = 33.7 Hz), 130.80, 128.99, 128.75, 128.61, 128.54, 128.48, 128.16, 127.50, 127.43, 127.19, 126.89, 125.52, 124.90, 124.82, 124.78, 122.81 (q, J = 271.4 Hz), 50.87 (d, J = 15.60 Hz), 36.60 (d, J = 17.10 Hz); ^{31}P NMR (121.5 MHz, $CDCl_3$) δ = -22.96 ppm; HRMS (ESI) m/z calcd. for $C_{47}H_{34}F_6NNaOP$ [M+Na] $^+$ = 796.2174, found = 796.2171.

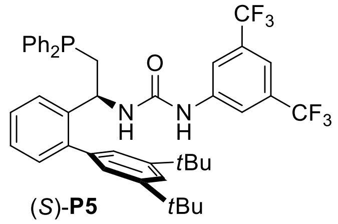

(*S*)-**P5**

(*S*)-**P5**; white solid; $[\alpha]_D^{20}$ = -16.6 (c = 0.33, $CHCl_3$); ^{1}H NMR (500 MHz, CD_3COCD_3): δ 8.80 (s, 1H), 8.17 (s, 2H), 7.66 (d, J = 8.0 Hz, 1H), 7.57–7.52 (m, 3H), 7.34–7.31 (m, 1H), 7.28–7.13 (m, 10H), 7.01–6.98 (m, 2H), 6.88 (d, J = 7.0 Hz, 1H), 5.51–5.46 (m, 1H), 3.74 (s, 1H), 2.48–2.43 (m, 1H), 2.37–2.31 (m, 1H), 1.36 (s, 18H); ^{13}C NMR (125 MHz, CD_3COCD_3): δ 154.66, 151.28, 143.51, 142.99 (d, J = 4.63 Hz), 142.80, 141.04, 140.17 (d, J = 13.88 Hz), 138.28 (d, J = 13.75 Hz), 133.16 (d, J = 12.25 Hz), 133.01 (d, J = 12.63 Hz), 132.31 (q, J = 32.63 Hz), 131.12, 129.45, 129.39, 129.20, 129.15, 129.11, 129.10, 128.43, 127.63, 126.24, 124.95, 124.49 (q, J = 270.13 Hz), 121.56, 118.41 (d, J = 3.5 Hz), 114.69 (m), 49.73 (d, J = 16.25 Hz), 38.18 (d, J = 16.38 Hz), 35.52, 31.81; ^{31}P NMR (202.5 MHz, CD_3COCD_3) δ = -24.58 ppm; ^{19}F NMR (376 MHz, CD_3COCD_3) δ = -63.56 ppm; HRMS (ESI) m/z calcd. for $C_{43}H_{44}F_6N_2OP$ [M+H] $^+$ = 749.3090, found = 749.3082.

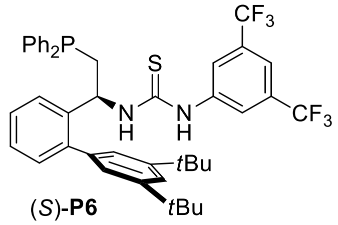

(*S*)-**P6**

(*S*)-**P6**; white solid; $[\alpha]_D^{20}$ = -8.8 (c = 0.33, $CHCl_3$); ^{1}H NMR (500 MHz, CD_3COCD_3): δ 9.55 (s, 1H), 8.42 (s, 2H), 7.76 (s, 1H), 7.63–7.59 (m, 3H), 7.34–7.18 (m, 10H), 7.00–6.97 (m, 2H), 6.06 (br, 1H), 3.75 (d, J = 4.0 Hz, 1H), 2.64–2.60 (m, 1H), 2.45–2.40 (m, 1H), 1.39 (s, 18H); ^{13}C NMR (125 MHz, CD_3COCD_3): δ 181.11,

151.29, 142.67 (d, J = 16.00 Hz), 141.92 (d, J = 5.38 Hz), 141.03, 139.96 (d, J = 13.38 Hz), 138.04 (d, J = 13.13 Hz), 133.29 (d, J = 19.25 Hz), 132.92 (d, J = 19.25 Hz), 132.51 (q, J = 32.88 Hz), 131.29, 129.41 (d, J = 6.5 Hz), 129.11 (d, J = 8.00 Hz), 129.01 (d, J = 7.00 Hz), 128.30, 127.61, 125.42, 124.58, 124.29 (q, J = 270.75 Hz), 121.68, 117.36-117.30 (m), 54.43 (d, J = 15.25 Hz), 37.19 (d, J = 17.00 Hz), 35.50, 31.88; ^{31}P NMR (202.5 MHz, CD_3COCD_3) δ = -23.86 ppm; ^{19}F NMR (376 MHz, CD_3COCD_3) δ = -67.71 ppm; HRMS (ESI) m/z calcd. for $C_{43}H_{44}F_6N_2SP$ [M+H] $^+$ = 765.2862, found = 765.2865.

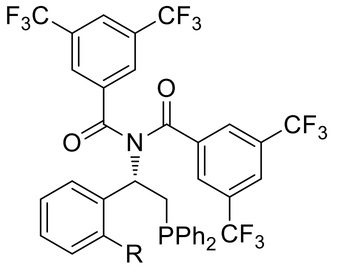

R = 3,5-(tBu)$_2$C$_6$H$_3$

(S)-**P7**

(S)-**P6**; white solid; $[\alpha]_D^{20}$ = -70.8 (c = 0.33, $CHCl_3$); ^{1}H NMR (500 MHz, CD_3COCD_3): δ 8.18–8.17 (m, 1H), 7.77–7.34 (m, 6H), 7.61–7.60 (m, 1H), 7.54–7.51 (m, 1H), 7.40–7.19 (m, 10H), 7.10–7.06 (m, 2H), 6.99–6.96 (m, 2H), 6.34–6.30 (m, 1H), 3.15–3.08 (m, 1H), 2.83–2.78 (m, 1H), 1.39–1.29 (m, 18H); ^{13}C NMR (125 MHz, CD_3COCD_3): δ 170.67, 150.87, 142.38, 139.80, 139.65, 139.381 (d, J = 7.25 Hz), 138.14 (d, J = 10.38 Hz), 138.81 (d, J = 10.88 Hz), 132.85 (d, J = 20.05 Hz), 132.191 (q, J = 34.25 Hz), 131.64 (d, J = 17.75 Hz), 131.06, 129.35, 129.21 (d, J = 7.38 Hz), 128.61, 128.35, 128.34, 128.30, 128.21, 127.73, 126.68, 124.84, 123.70, 122.23 (q, J = 271.63 Hz), 58.33 (d, J = 13.50 Hz), 34.99, 32.49 (d, J = 17.13 Hz), 31.44; ^{31}P NMR (202.5 MHz, CD_3COCD_3) δ = -21.96 ppm; ^{19}F NMR (376 MHz, $CDCl_3$) δ = -63.57 ppm; HRMS (ESI) m/z calcd. for $C_{52}H_{45}F_{12}NO_2P$ [M+H] $^+$ = 974.2991, found = 974.2993.

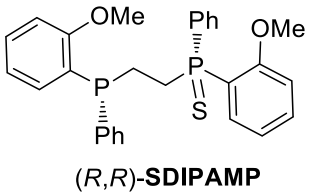

(R,R)-**SDIPAMP**

(*R*,*R*)-**SDIPAMP**; white solid; $[\alpha]_D^{20}$ = +39.9 (*c* = 0.33, $CHCl_3$); ^{1}H NMR (500 MHz, CD_3COCD_3): δ 8.30–8.25 (m, 1H), 7.70–7.65 (m, 2H), 7.53–7.29 (m, 10H), 7.15–7.12 (m, 1H), 7.07–7.04 (m, 1H), 6.91–6.82 (m, 3H), 3.69 (s, 3H), 3.59 (s, 3H), 2.95–2.85 (m, 1H), 2.66–2.56 (m, 1H), 2.38–2.23 (m, 2H); ^{13}C NMR (125 MHz, CD_3COCD_3): δ 161.02 (d, *J* = 12.75 Hz), 159.75 (d, *J* = 2.50 Hz), 136.717 (d, *J* = 12.88 Hz), 136.31 (d, *J* = 9.75 Hz), 134.28, 133.94 (q, *J* = 2.13 Hz), 133.62, 133.23, 133.08, 132.09 (d, *J* = 4.38 Hz), 130.76 (d, *J* = 2.75 Hz), 130.59, 130.51, 130.10, 128.34, 128.32 (d, *J* = 6.88 Hz), 128.05 (d, *J* = 12.25 Hz), 125.69 (d, *J* = 15.75 Hz), 121.05 (d, *J* = 12.25 Hz), 120.80, 119.08, 118.47, 110.72 (d, *J* = 7.25 Hz), 110.24, 55.21 (d, *J* = 34.75 Hz), 27.61 (dd, *J* = 55, 19.63 Hz), 18.31 (dd, *J* = 14.25, 3.38, Hz); ^{31}P NMR (202.5 MHz, CD_3COCD_3) δ = 45.17, -21.96 ppm; HRMS (ESI) m/z calcd. for $C_{28}H_{29}O_2P_2S$ $[M+H]^+$ = 491.1358, found = 491.1370.

4. Typical Procedure for the Enantioselective [3+2] Cycloaddition of Allenes with *β*–Perfluoroalkyl *α,β*–Enones

Enantioselective [3+2] cycloadditions of *γ*-aryl substituted allenoates with enone

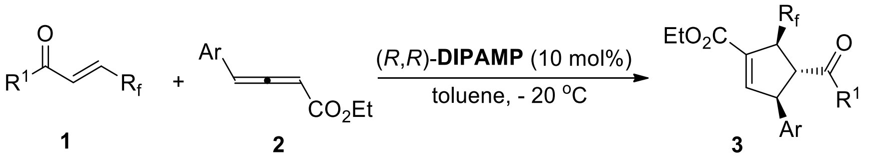

Under Ar, a stirred solution of **1**[2] (0.2 mmol) and recemic **2** (0.3 mmol) in toluene (2 mL) was cooled to -20 °C. Subsequently, (*R*,*R*)-**DIPAMP** (0.02 mmol) was added in one portion. The reaction mixture was stirred at -20 °C until completion of the material as indicated by TLC. Then the solvents were removed in vacuo and the residue was directly purified by silica gel chromatography using petroleum ether/EtOAc as the eluent to afford the desired cycloaddition product **3**.

Enantioselective [3+2] cycloadditions of *γ*-alkyl substituted allenoates with enone

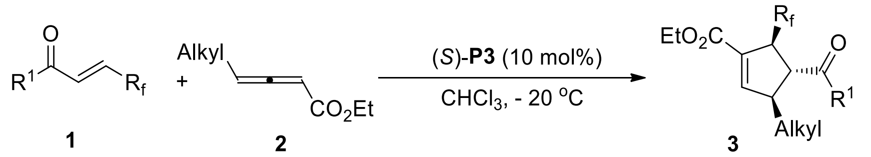

Under Ar, a stirred solution of **1** (0.2 mmol) and recemic **2** (0.44 mmol) in $CHCl_3$ (2

mL) was cooled to -20 °C. Subsequently, (*S*)-**P3** (0.02 mmol) was added in one portion. The reaction mixture was stirred at -20 °C until completion of the material as indicated by TLC. Then the solvents were removed in vacuo and the residue was directly purified by silica gel chromatography using petroleum ether/EtOAc as the eluent to afford the desired cycloaddition product **3**.

5. The Structural Assignment for the Regioisomer

In order to confirm the structure of the minor product which was observed in the (*S*)-**P3** catalyzed enantioselective [3+2] cycloadditions of *γ*-alkyl substituted allenoates with *β*-perfluoro substituted enone, we have isolated the isomer of **3cl** and confirmed its structurue by NMR analysis. According to these NMR spectra, the minor product **3cl'** was assigned to the regioisomer of **3cl**.

(a) ^{1}H NMR spectra for **3cl'**

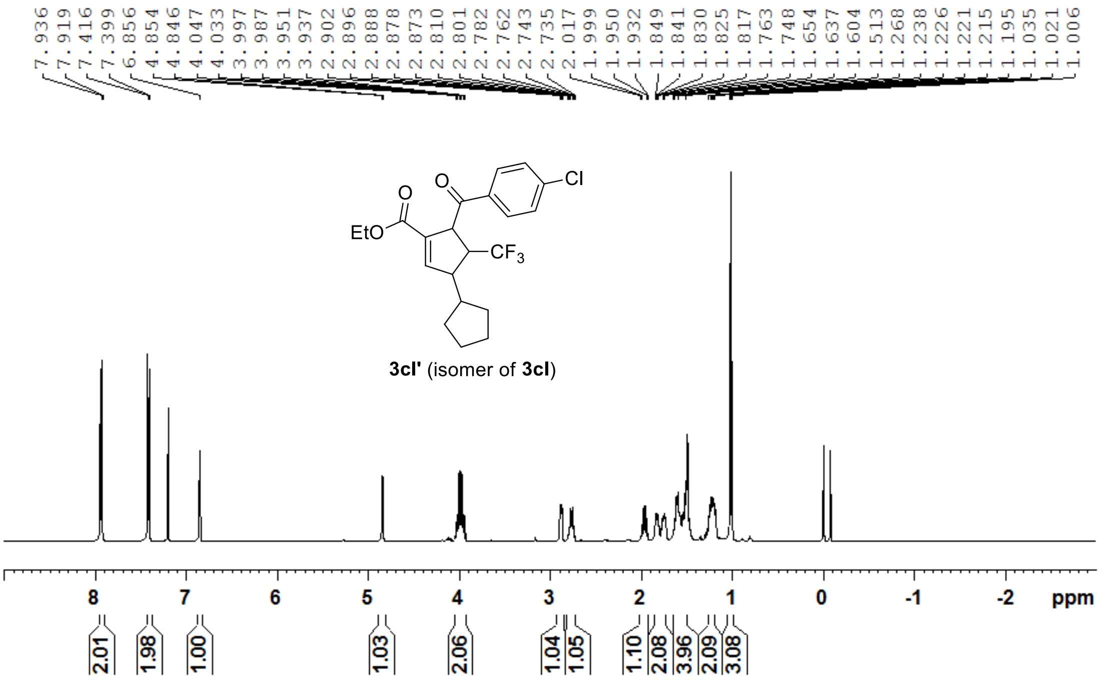

(b) ^{13}C NMR spectra for **3cl'**

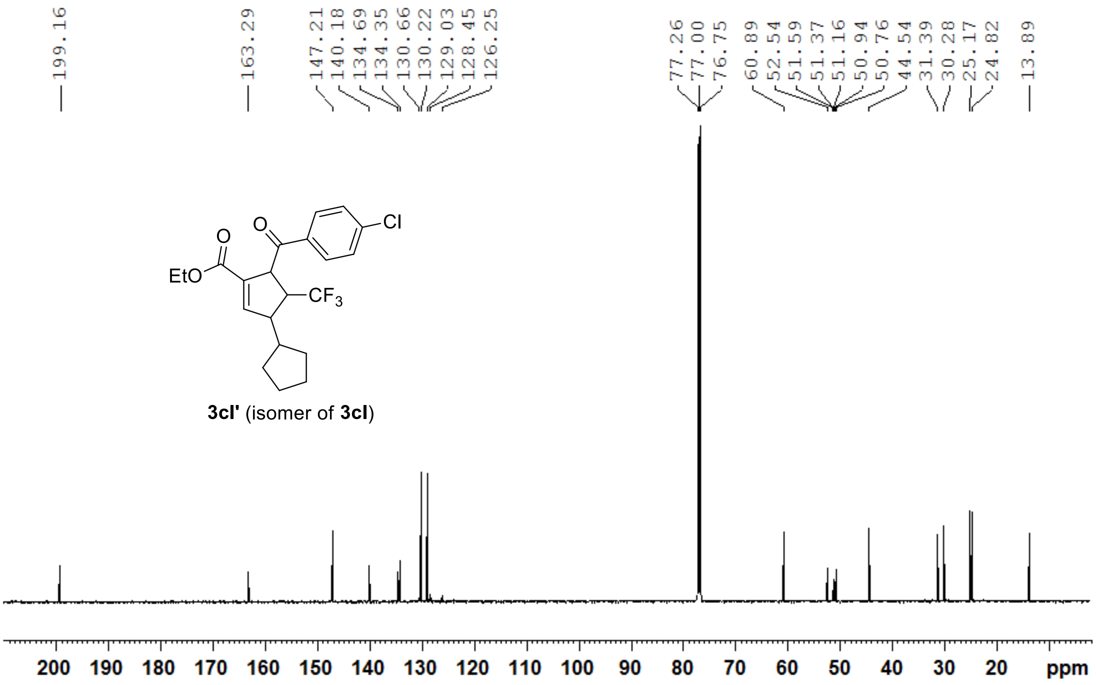

(c) DEPT 90 spectra for **3cl'**

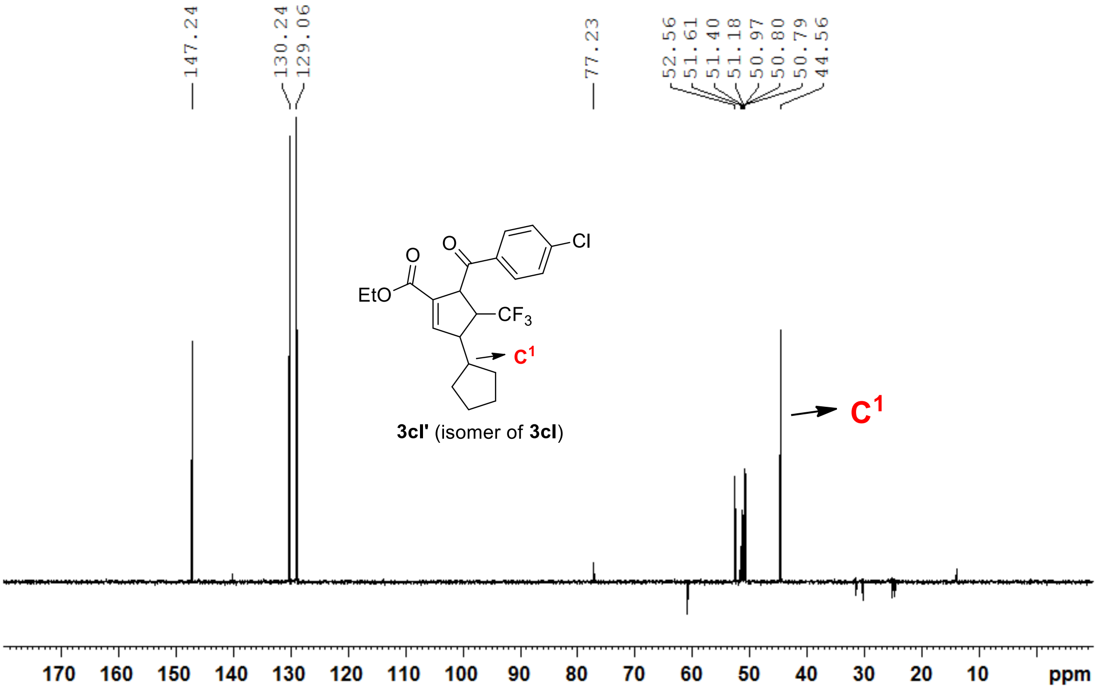

(**d**) DEPT 135 spectra for **3cl'**

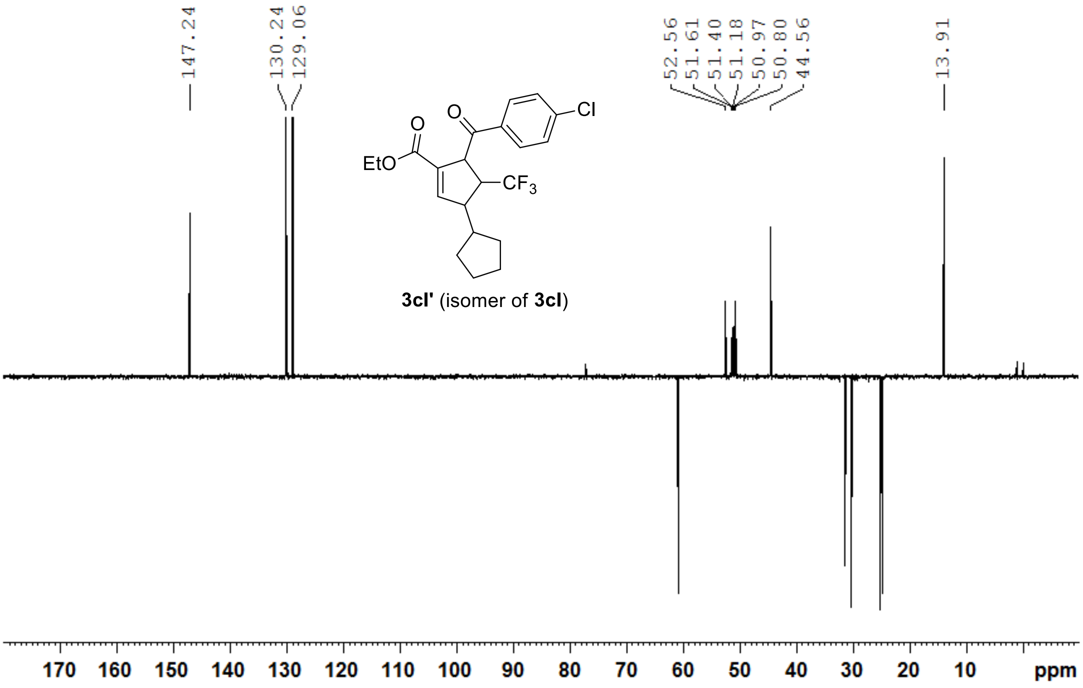

(**e**) HSQC spectra for **3cl'**

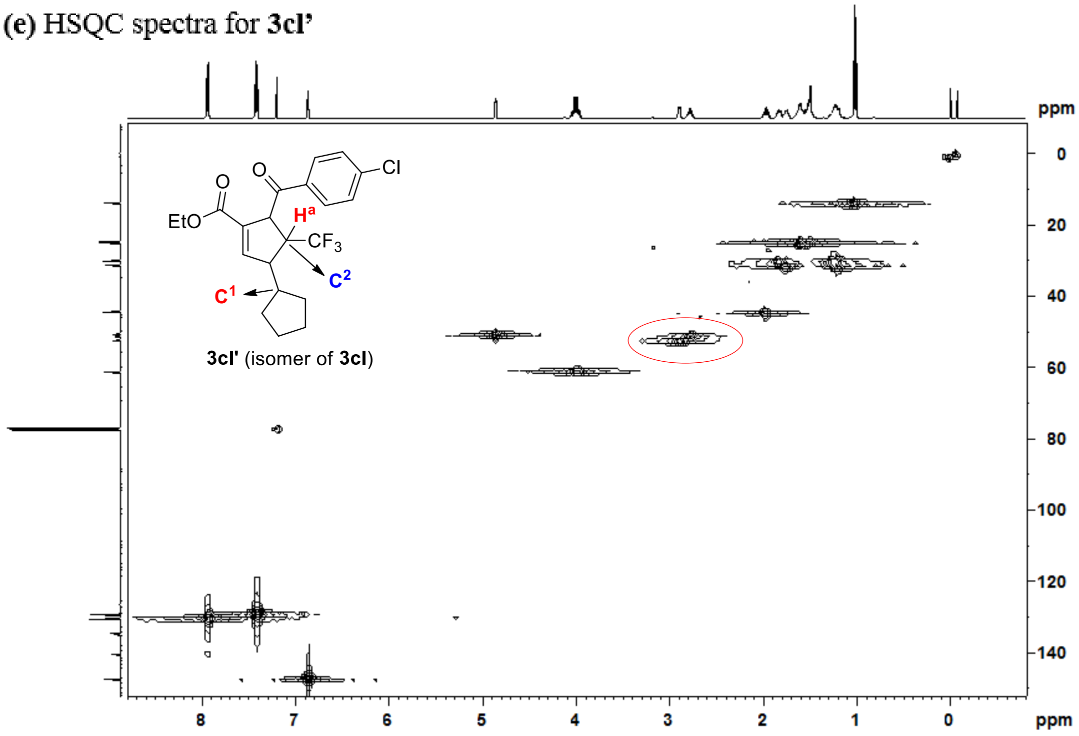

(e) HSQC spectra for **3cl'**

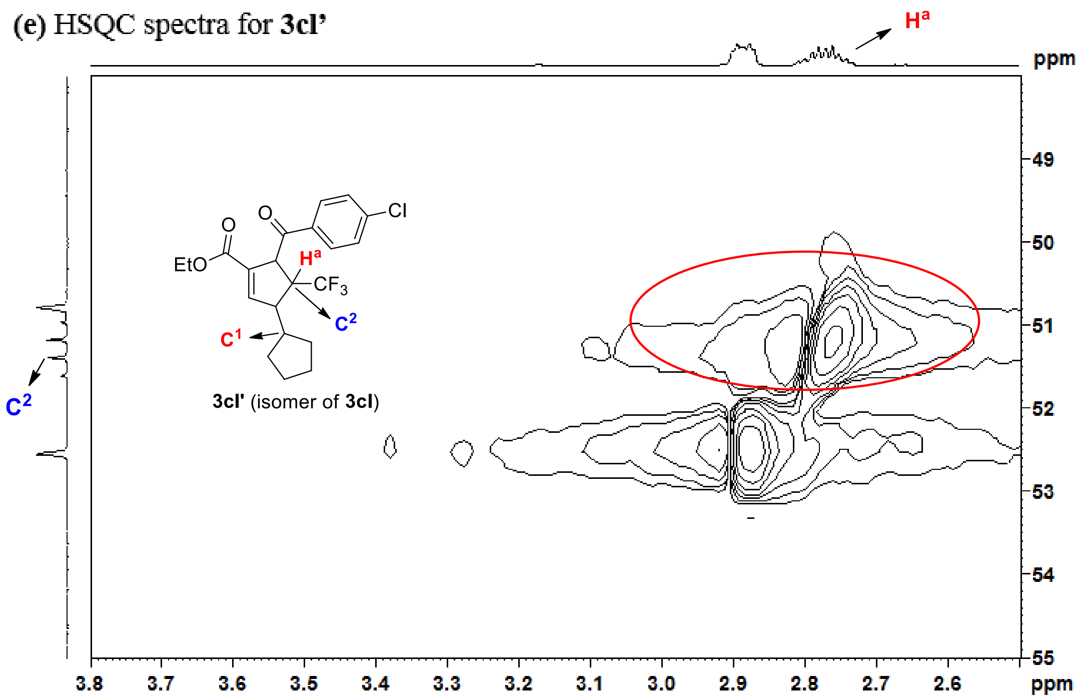

(f) HMBC spectra for **3l'**

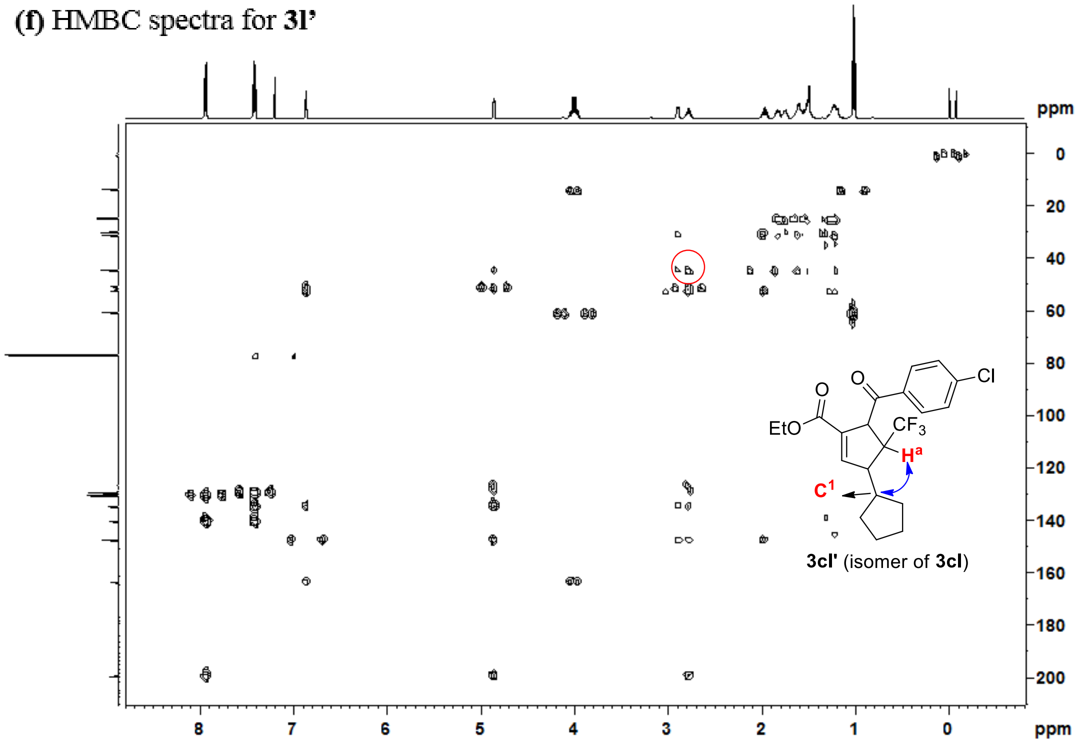

(f) HMBC spectra for 3l'

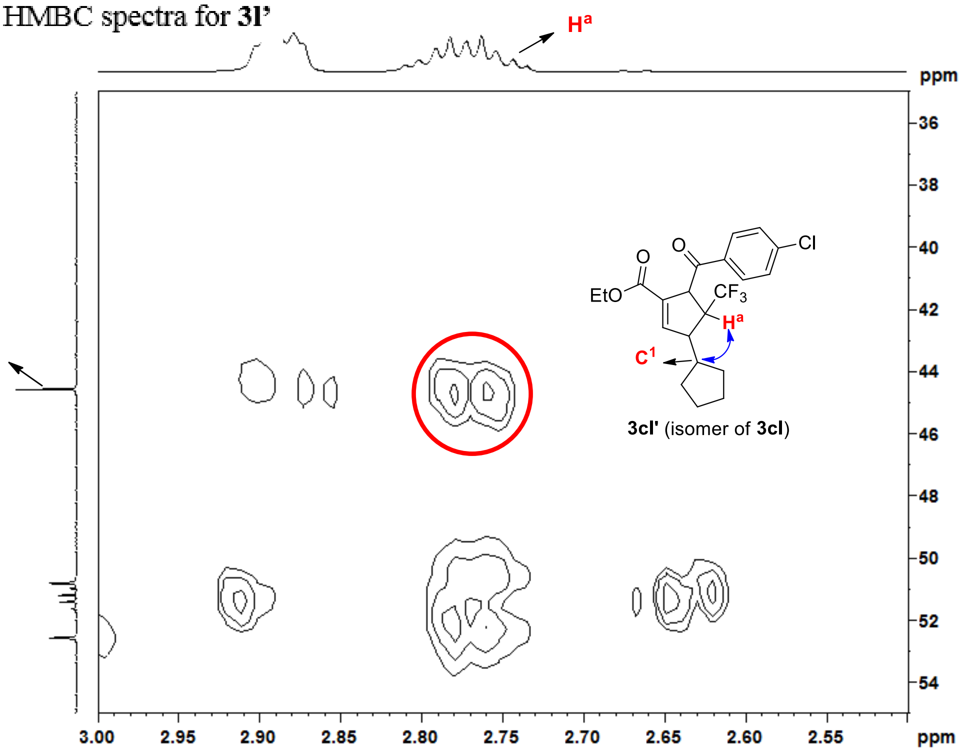

6. Procedure for the "Deracemization" and Kinetic Resolution of Recemic Allenoates

Procedure for the "Deracemization" of Racemic 2a

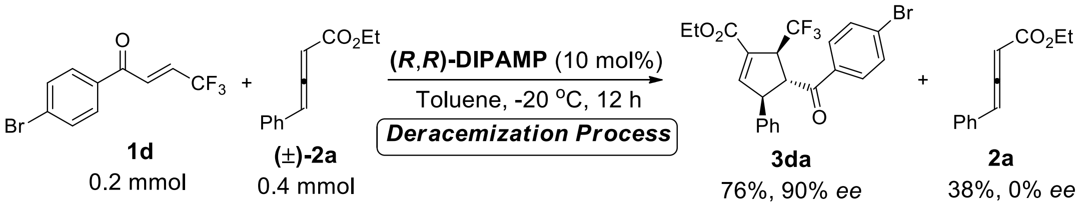

Under Ar, a stirred solution of **1d** (0.2 mmol) and racemic **2a** (0.4 mmol) in toluene (2 mL) was cooled to -20 °C. Subsequently, (*R*,*R*)-**DIPAMP** (0.02 mmol) was added in one portion. The reaction mixture was stirred at -20 °C for 12 h. Then the solvents were removed in vacuo and the residue was directly purified by silica gel chromatography using petroleum ether/EtOAc as the eluent to afford the **3da** (76%, 90% *ee*) and **2a** (38%, 0% *ee*).

Procedure for the Kinetic Resolution of Racemic 2g

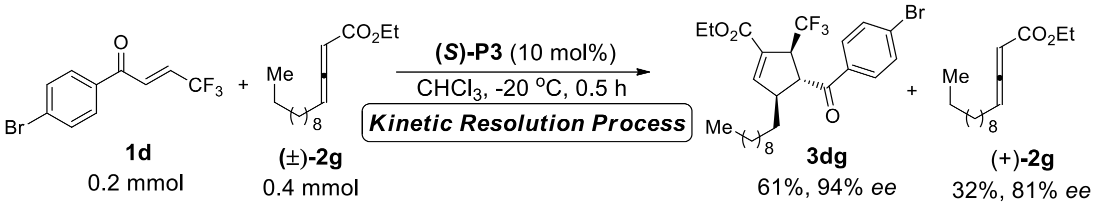

Under Ar, a stirred solution of **1d** (0.2 mmol) and racemic **2g** (0.4 mmol) in $CHCl_3$ (2 mL) was cooled to -20 °C. Subsequently, (*S*)-**P3** (0.02 mmol) was added in one portion. The reaction mixture was stirred at -20 °C for 0.5 h. Then the solvents were removed in vacuo and the residue was directly purified by silica gel chromatography using petroleum ether/EtOAc as the eluent to afford the **3dg** (61%, 94% *ee*) and **2g** (32%, 81% *ee*).

7. X-ray Crystal Structure for 3aa

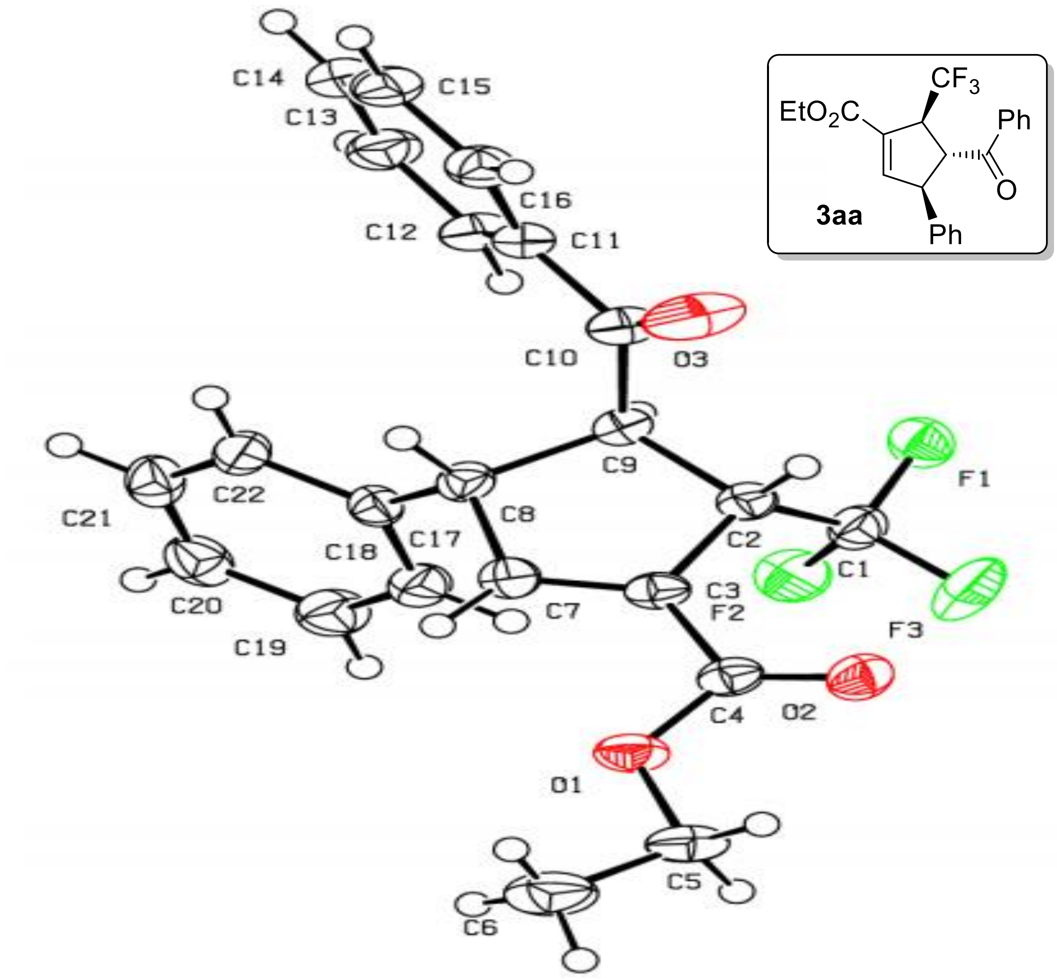

8. General Data and HPLC Spectra for Cycloaddition Product 3

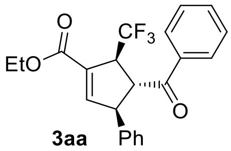

3aa

3aa; white solid; $[\alpha]_D^{20}$ = + 67.2 (c = 0.33, $CHCl_3$); ^{1}H NMR (400 MHz, $CDCl_3$): δ 7.70–7.68 (m, 2H), 7.57–7.54 (m, 1H), 7.38–7.30 (m, 5H), 7.09–7.07 (m, 2H), 6.82 (s, 1H), 4.61–4.53 (m, 1H), 4.37–4.21 (m, 3H), 4.00–3.99 (m, 1H), 1.33 (t, J = 7.20 Hz, 3H); ^{13}C NMR (100 MHz, $CDCl_3$): δ 198.22, 163.27, 147.41, 140.19, 135.26, 133.82, 131.95, 129.13, 128.97, 128.62, 128.03, 127.86, 127.68, 126.29 (q, J = 277.90 Hz), 61.06, 55.47, 54.10, 51.35 (q, J = 28.90 Hz), 14.08; ^{19}F NMR (376 MHz, $CDCl_3$) δ = -67.48 ppm; Enantiomeric excess: 92%, determined by HPLC (Chiralpak IC, hexane/i-PrOH = 95/05; flow rate 0.5 ml/min; 25 °C; 230 nm), first peak: t_R = 15.34 min, second peak: t_R = 17.34 min; HRMS (ESI) m/z calcd. for $C_{22}H_{19}F_3NaO_3$ $[M+Na]^+$ = 411.1179, found = 411.1182.

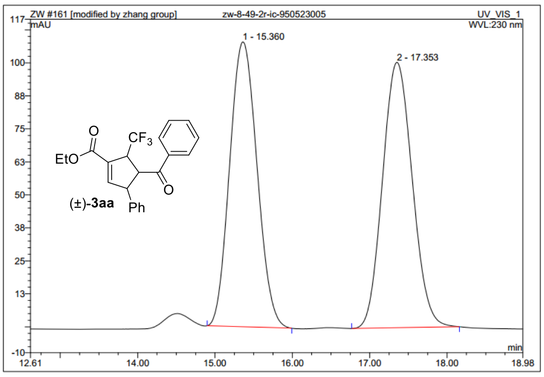

No.	Ret.Time min	Peak Name	Height mAU	Area mAU*min	Rel.Area %	Amount	Type
1	15.36	n.a.	107.931	44.250	49.68	n.a.	BMB*
2	17.35	n.a.	100.530	44.823	50.32	n.a.	BMB*
Total:			208.461	89.072	100.00	0.000	

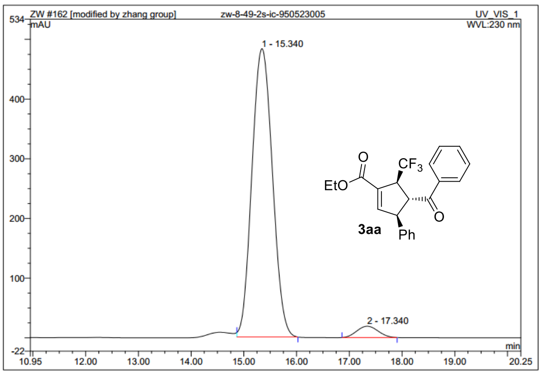

No.	Ret.Time min	Peak Name	Height mAU	Area mAU*min	Rel.Area %	Amount	Type
1	15.34	n.a.	482.951	217.640	96.11	n.a.	MB*
2	17.34	n.a.	18.914	8.816	3.89	n.a.	BMB*
Total:			501.865	226.456	100.00	0.000	

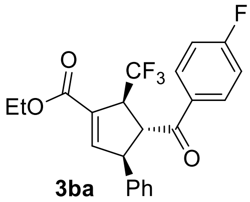

3ba

3ba; colorless oil; $[\alpha]_D^{20}$ = + 122.4 (c = 0.33, $CHCl_3$); ^{1}H NMR (400 MHz, $CDCl_3$): δ 7.72–7.68 (m, 2H), 7.36–7.32 (m, 3H), 7.10–7.00 (m, 4H), 6.81 (s, 1H), 4.61–4.53 (m,

1H), 4.37–4.18 (m, 3H), 3.98–3.96 (m, 1H), 1.33 (t, J = 7.20 Hz, 3H); ^{13}C NMR (100 MHz, $CDCl_3$): δ 196.61, 166.22 (d, J = 255.00 Hz), 163.22, 147.26, 140.10, 131.85 (d, J = 9.50 Hz), 131.67 (d, J = 2.80 Hz), 129.08, 127.99, 126.25 (d, J = 278.00 Hz), 115.81 (d, J = 21.9 Hz), 61.10, 55.55, 54.22, 51.46 (q, J = 29.00 Hz), 14.07; ^{19}F NMR (376 MHz, $CDCl_3$) δ = -67.52, -103.61 ppm; Enantiomeric excess: 94%, determined by HPLC (Chiralpak IC, hexane/*i*-PrOH = 95/05; flow rate 0.5 ml/min; 25 °C; 230 nm), first peak: t_R = 14.49 min, second peak: t_R = 16.46 min; HRMS (ESI) m/z calcd. for $C_{22}H_{18}F_4NaO_3$ $[M+Na]^+$ = 429.1084, found = 429.1093.

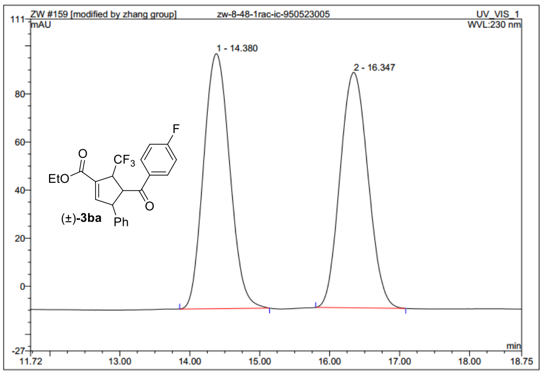

No.	Ret.Time min	Peak Name	Height mAU	Area mAU*min	Rel.Area %	Amount	Type
1	14.38	n.a.	106.085	45.973	50.38	n.a.	BMB*
2	16.35	n.a.	97.983	45.282	49.62	n.a.	BMB*
Total:			204.068	91.256	100.00	0.000	

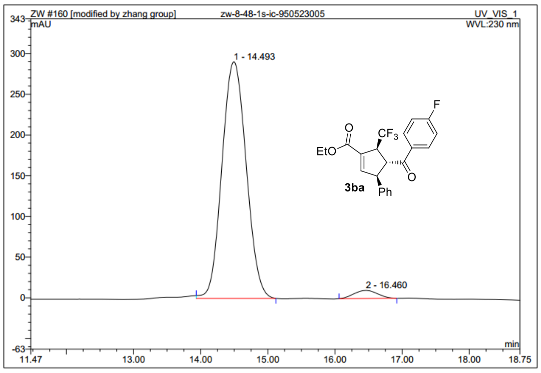

No.	Ret.Time min	Peak Name	Height mAU	Area mAU*min	Rel.Area %	Amount	Type
1	14.49	n.a.	290.623	121.731	96.79	n.a.	MB*
2	16.46	n.a.	9.933	4.034	3.21	n.a.	BMB*
Total:			300.556	125.765	100.00	0.000	

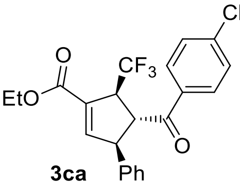

3ca

3ca; colorless oil; $[\alpha]_D^{20}$ = + 80.6 (c = 0.33, $CHCl_3$); ^{1}H NMR (500 MHz, $CDCl_3$): δ 7.64–7.62 (m, 2H), 7.38–7.33 (m, 5H), 7.12–7.10 (m, 2H), 6.83 (s, 1H), 4.62–4.56 (m, 1H), 4.38–4.24 (m, 2H), 4.21–4.19 (m, 1H), 4.00–3.98 (m, 1H), 1.35 (t, J = 7.00 Hz, 3H); ^{13}C NMR (125 MHz, $CDCl_3$): δ 196.93, 163.16, 147.24, 140.49, 139.98, 133.48, 131.85 (d, J = 1.75 Hz), 130.49, 129.08, 128.94, 128.01, 127.96, 126.19 (q, J = 278.13 Hz), 61.09, 55.45, 54.21, 51.36 (q, J = 29.00 Hz), 14.06; ^{19}F NMR (376 MHz, $CDCl_3$) δ = -67.49 ppm; Enantiomeric excess: 90%, determined by HPLC (Chiralpak AS-H, hexane/*i*-PrOH = 95/05; flow rate 0.5 ml/min; 25 °C; 230 nm), first peak: t_R = 10.82 min, second peak: t_R = 12.86 min; HRMS (ESI) m/z calcd. for $C_{22}H_{18}ClF_3NaO_3$ $[M+Na]^+$ = 445.0789, found = 445.0794.

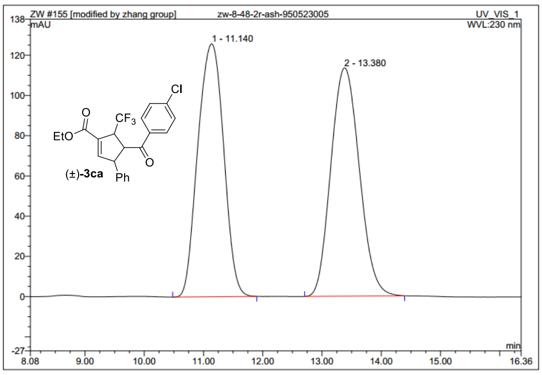

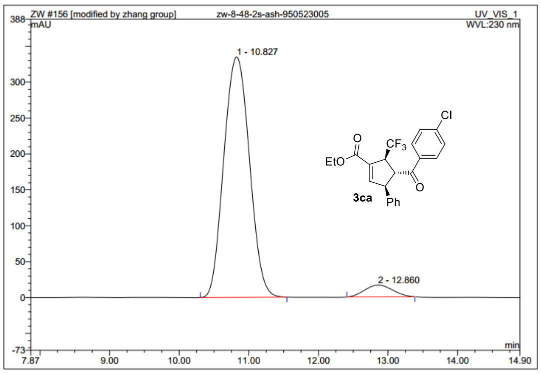

No.	Ret.Time min	Peak Name	Height mAU	Area mAU*min	Rel.Area %	Amount	Type
1	11.14	n.a.	125.749	63.568	50.17	n.a.	BMB*
2	13.38	n.a.	113.582	63.145	49.83	n.a.	BMB*
Total:			239.331	126.713	100.00	0.000	

No.	Ret.Time min	Peak Name	Height mAU	Area mAU*min	Rel.Area %	Amount	Type
1	10.83	n.a.	334.926	142.934	94.92	n.a.	BMB*
2	12.86	n.a.	16.537	7.655	5.08	n.a.	BMB*
Total:			351.463	150.589	100.00	0.000	

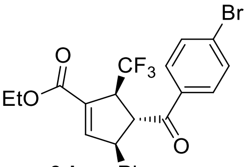

3da

3da; colorless oil; $[\alpha]_D^{20}$ = + 92.8 (c = 0.33, $CHCl_3$); ^{1}H NMR (400 MHz, $CDCl_3$): δ 7.54–7.48 (m, 4H), 7.36–7.32 (m, 3H), 7.10–7.08 (m, 2H), 6.80 (s, 1H), 4.60–4.52 (m, 1H), 4.37–4.21 (m, 2H), 4.18–4.16 (m, 1H), 3.98–3.95 (m, 1H), 1.33 (t, J = 7.20 Hz, 3H); ^{13}C NMR (100 MHz, $CDCl_3$): δ 197.19, 163.17, 147.22, 140.00, 133.95, 131.96, 131.90, 130.56, 129.31, 129.11, 128.04, 127.97, 126.20 (q, J = 278.20 Hz), 61.11, 55.46, 54.23, 51.43 (q, J = 28.90 Hz), 14.07; ^{19}F NMR (376 MHz, $CDCl_3$) δ = -67.49 ppm; Enantiomeric excess: 90%, determined by HPLC (Chiralpak AS-H, hexane/*i*-PrOH = 95/05; flow rate 0.5 ml/min; 25 °C; 230 nm), first peak: t_R = 11.01 min, second peak: t_R = 13.26 min; HRMS (ESI) m/z calcd. for $C_{22}H_{18}BrF_3NaO_3$ $[M+Na]^+$ = 489.0284, found = 489.0287.

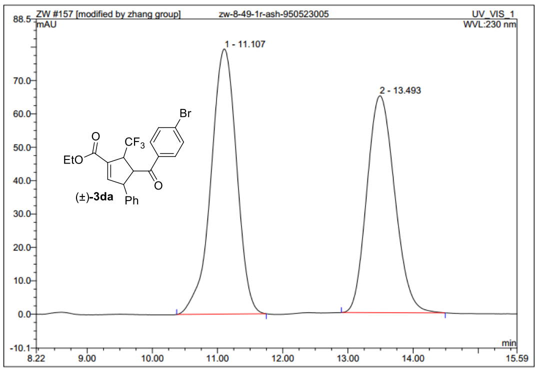

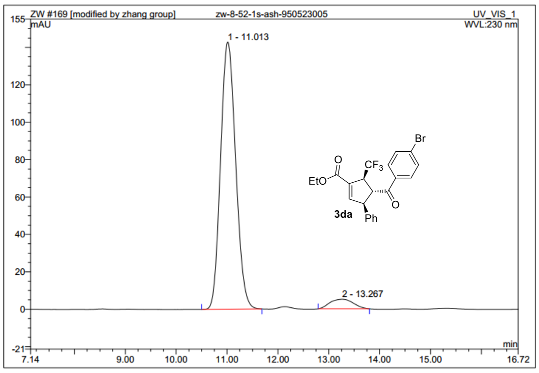

No.	Ret.Time min	Peak Name	Height mAU	Area mAU*min	Rel.Area %	Amount	Type
1	11.11	n.a.	79.420	36.236	53.01	n.a.	BMB*
2	13.49	n.a.	64.998	32.117	46.99	n.a.	BMB*
Total:			144.418	68.353	100.00	0.000	

No.	Ret.Time min	Peak Name	Height mAU	Area mAU*min	Rel.Area %	Amount	Type
1	11.01	n.a.	142.859	49.184	94.79	n.a.	BMB*
2	13.27	n.a.	5.084	2.704	5.21	n.a.	BMB*
Total:			147.943	51.888	100.00	0.000	

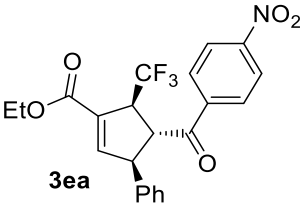

3ea

3ea; colorless oil; $[\alpha]_D^{20}$ = + 68.3 (*c* = 0.33, $CHCl_3$); ^1H NMR (500 MHz, $CDCl_3$): δ 8.21–8.19 (m, 2H), 7.83–7.81 (m, 2H), 7.37–7.36 (m, 3H), 7.11–7.09 (m, 2H), 6.82 (s, 1H), 4.64–4.57 (m, 1H), 4.39–4.25 (m, 3H), 4.01–3.99 (m, 1H), 1.36 (t, *J* = 7.00 Hz, 3H); ^{13}C NMR (125 MHz, $CDCl_3$): δ 196.77, 163.02, 150.67, 146.94, 139.71, 139.65, 131.81, 130.09, 129.28, 128.32, 127.87, 126.06 (q, *J* = 280.25 Hz), 123.75, 61.22, 55.29, 55.04, 51.38 (q, *J* = 29.13 Hz), 14.07; ^{19}F NMR (376 MHz, $CDCl_3$) δ = -67.49 ppm; Enantiomeric excess: 90%, determined by HPLC (Chiralpak AS-H, hexane/*i*-PrOH = 95/05; flow rate 0.5 ml/min; 25 °C; 230 nm), first peak: t_R = 19.11 min, second peak: t_R = 22.64 min; HRMS (ESI) m/z calcd. for $C_{22}H_{18}F_3NNaO_5$ $[M+Na]^+$ = 456.1029, found = 456.1042.

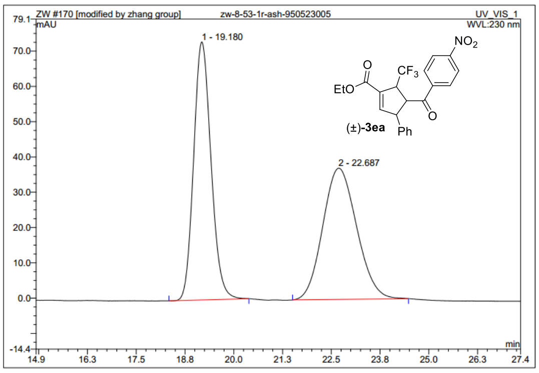

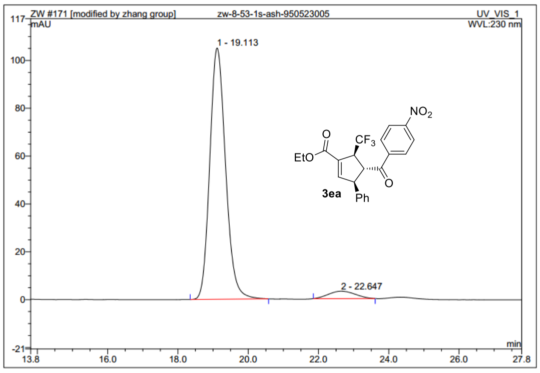

No.	Ret.Time min	Peak Name	Height mAU	Area mAU*min	Rel.Area %	Amount	Type
1	19.18	n.a.	73.108	37.699	50.21	n.a.	BMB*
2	22.69	n.a.	37.196	37.384	49.79	n.a.	BMB*
Total:			110.304	75.083	100.00	0.000	

No.	Ret.Time min	Peak Name	Height mAU	Area mAU*min	Rel.Area %	Amount	Type
1	19.11	n.a.	105.037	54.745	95.29	n.a.	BMB*
2	22.65	n.a.	3.092	2.704	4.71	n.a.	BMB*
Total:			108.129	57.450	100.00	0.000	

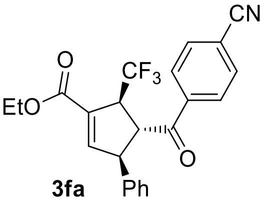

3fa

3fa; colorless oil; $[\alpha]_D^{20}$ = + 82.8 (*c* = 0.33, $CHCl_3$); ^1H NMR (500 MHz, $CDCl_3$): δ 7.75 (d, *J* = 8.00 Hz, 2H), 7.66 (d, *J* = 8.50 Hz, 2H), 7.36–7.35 (m, 3H), 7.09–7.08 (m,

2H), 6.81 (s, 1H), 4.62–4.56 (m, 1H), 4.38–4.21 (m, 3H), 3.98–3.97 (m, 1H), 1.35 (t, *J* = 7.00 Hz, 3H); ^{13}C NMR (125 MHz, $CDCl_3$): δ 196.96, 163.05, 146.98, 139.69, 138.23, 132.40, 131.82, 129.42, 129.23, 128.26, 127.88, 126.07 (q, *J* = 278.13 Hz), 117.60, 117.07, 61.21, 55.30, 54.75 (d, *J* = 1.25 Hz), 51.32 (q, *J* = 29.38 Hz), 14.06; ^{19}F NMR (376 MHz, $CDCl_3$) δ = -67.51 ppm; Enantiomeric excess: 88%, determined by HPLC (Chiralpak AD-H, hexane/*i*-PrOH = 90/10; flow rate 0.8 ml/min; 25 °C; 230 nm), first peak: t_R = 10.58 min, second peak: t_R = 12.53 min; HRMS (ESI) m/z calcd. for $C_{23}H_{18}F_3NNaO_3$ $[M+Na]^+$ = 436.1131, found = 436.1131.

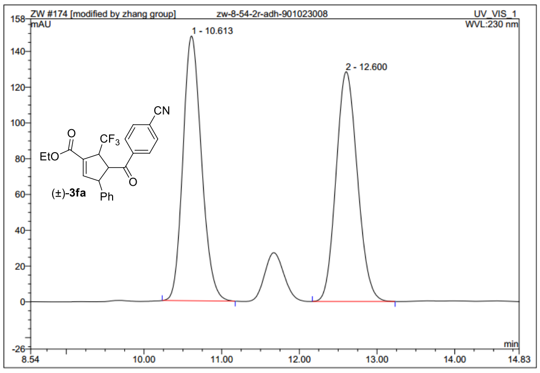

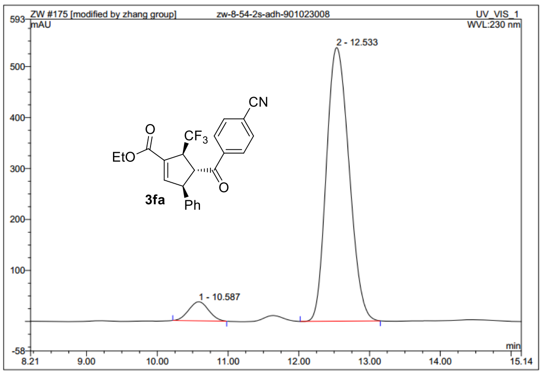

No.	Ret.Time min	Peak Name	Height mAU	Area mAU*min	Rel.Area %	Amount	Type
1	10.61	n.a.	147.938	41.076	50.08	n.a.	BMB*
2	12.60	n.a.	128.339	40.949	49.92	n.a.	BMB*
Total:			276.277	82.025	100.00	0.000	

No.	Ret.Time min	Peak Name	Height mAU	Area mAU*min	Rel.Area %	Amount	Type
1	10.59	n.a.	37.365	12.153	5.76	n.a.	BMB*
2	12.53	n.a.	536.718	198.660	94.24	n.a.	BMB*
Total:			574.084	210.813	100.00	0.000	

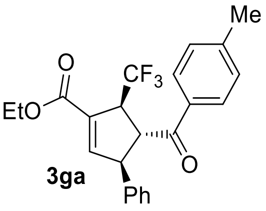

3ga; colorless oil; $[\alpha]_D^{20}$ = + 76.5 (*c* = 0.33, $CHCl_3$); ^{1}H NMR (400 MHz, $CDCl_3$): δ 7.59 (d, *J* = 8.40 Hz, 2H), 7.35–7.30 (m, 3H), 7.15 (d, *J* = 8.00 Hz, 2H), 7.11–7.09 (m, 2H), 6.82 (s, 1H), 4.59–4.51 (m, 1H), 4.37–4.20 (m, 3H), 3.99–3.98 (m, 1H), 2.38 (s, 3H), 1.33 (t, *J* = 7.20 Hz, 3H); ^{13}C NMR (100 MHz, $CDCl_3$): δ 197.78, 163.30, 147.52, 144.88, 140.27, 132.66, 131.91, 129.31, 129.27, 128.93, 128.04, 127.79, 126.31 (q, *J* = 278.00 Hz), 61.02, 55.53, 53.86, 51.42 (q, *J* = 28.80 Hz), 21.62, 14.07; ^{19}F NMR (376 MHz, $CDCl_3$) δ = -67.47 ppm; Enantiomeric excess: 92%, determined by HPLC (Chiralpak AD-H, hexane/*i*-PrOH = 90/10; flow rate 0.6 ml/min; 25 °C; 230 nm), first peak: t_R = 8.88 min, second peak: t_R = 12.70 min; HRMS (ESI) m/z calcd.

for $C_{23}H_{21}F_3NaO_3$ $[M+Na]^+$ = 425.1335, found = 425.1343.

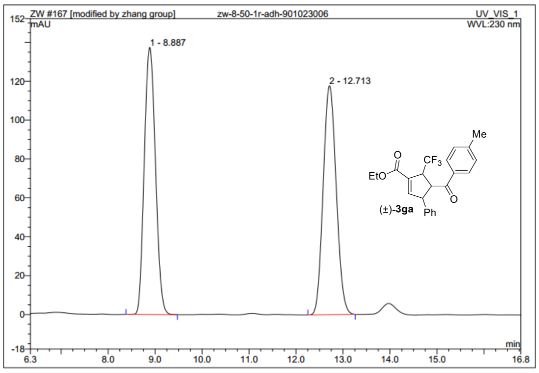

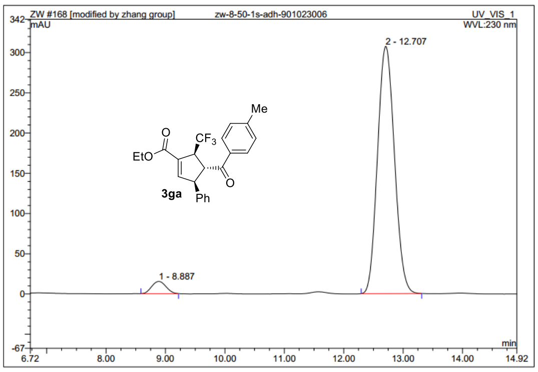

No.	Ret.Time min	Peak Name	Height mAU	Area mAU*min	Rel.Area %	Amount	Type
1	8.89	n.a.	137.406	37.902	50.01	n.a.	BMB*
2	12.71	n.a.	117.814	37.883	49.99	n.a.	BMB*
Total:			255.220	75.785	100.00	0.000	

No.	Ret.Time min	Peak Name	Height mAU	Area mAU*min	Rel.Area %	Amount	Type
1	8.89	n.a.	15.245	4.216	3.99	n.a.	BMB*
2	12.71	n.a.	307.460	101.468	96.01	n.a.	BMB*
Total:			322.706	105.684	100.00	0.000	

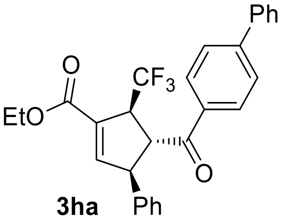

3ha

3jd; colorless oil; $[\alpha]_D^{20}$ = + 109.3 (c = 0.33, $CHCl_3$); ^{1}H NMR (400 MHz, $CDCl_3$): δ 7.76 (d, J = 8.40 Hz, 2H), 7.59–7.56 (m, 4H), 7.47–7.29 (m, 6H), 7.14–7.11 (m, 2H), 6.83 (s, 1H), 4.64–4.56 (m, 1H), 4.37–4.21 (m, 3H), 4.03–4.02 (m, 1H), 1.33 (t, J = 7.20 Hz, 3H); ^{13}C NMR (100 MHz, $CDCl_3$): δ 197.71, 163.27, 147.44, 146.50, 140.23, 139.49, 133.85, 131.93, 129.74, 129.00, 128.95, 128.41, 128.06, 127.88, 127.22, 127.19, 126.32 (q, J = 278.10 Hz), 61.05, 55.56, 54.12, 51.44 (q, J = 28.90 Hz), 14.07; ^{19}F NMR (376 MHz, $CDCl_3$) δ = -67.40 ppm; Enantiomeric excess: 92%, determined by HPLC (Chiralpak AD-H, hexane/i-PrOH = 90/10; flow rate 0.6 ml/min; 25 °C; 230 nm), first peak: t_R = 11.10 min, second peak: t_R = 13.98 min; HRMS (ESI) m/z calcd. for $C_{28}H_{23}F_3NaO_3$ $[M+Na]^+$ = 487.1491, found = 487.1497.

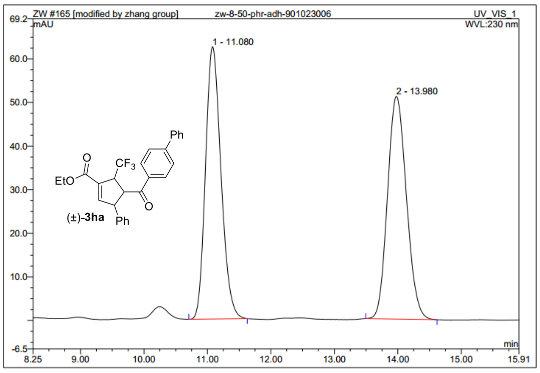

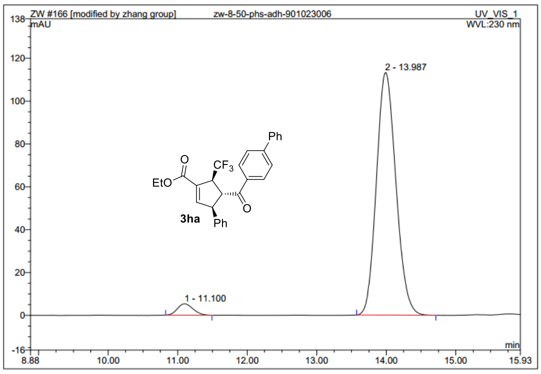

No.	Ret.Time min	Peak Name	Height mAU	Area mAU*min	Rel.Area %	Amount	Type
1	11.08	n.a.	62.449	17.611	49.99	n.a.	BMB*
2	13.98	n.a.	51.084	17.619	50.01	n.a.	BMB*
Total:			113.533	35.230	100.00	0.000	

No.	Ret.Time min	Peak Name	Height mAU	Area mAU*min	Rel.Area %	Amount	Type
1	11.10	n.a.	5.341	1.382	3.62	n.a.	BMB*
2	13.99	n.a.	113.226	36.767	96.38	n.a.	BMB*
Total:			118.568	38.149	100.00	0.000	

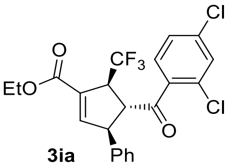

3ia; colorless oil; $[\alpha]_D^{20}$ = + 102.0 (*c* = 0.33, $CHCl_3$); ^1H NMR (400 MHz, $CDCl_3$): δ 7.42 (d, *J* = 5.60 Hz, 1H), 7.26–7.17 (m, 5H), 6.93–6.90 (m, 2H), 6.81 (s, 1H), 4.52–4.44 (m, 1H), 4.36–4.21 (m, 2H), 4.15–4.11 (m, 2H), 1.33 (t, *J* = 6.80 Hz, 3H); ^{13}C NMR (100 MHz, $CDCl_3$): δ 198.48, 163.07, 147.02, 140.05, 137.91, 135.99, 132.54, 131.76 (q, *J* = 2.00 Hz), 130.60, 130.10, 128.90, 127.78, 127.41, 127.18, 126.02 (q, *J* = 276.30 Hz), 61.14, 58.27, 53.68, 50.12 (q, *J* = 29.30 Hz), 14.06; ^{19}F NMR (376 MHz, $CDCl_3$) δ = -67.96 ppm; Enantiomeric excess: 99%, determined by HPLC (Chiralpak AS-H, hexane/*i*-PrOH = 90/10; flow rate 0.8 ml/min; 25 °C; 230 nm), first peak: t_R = 6.37 min, second peak: t_R = 7.08 min; HRMS (ESI) m/z calcd. for $C_{22}H_{17}Cl_2F_3NaO_3$ $[M+Na]^+$ = 479.0399, found = 479.0399.

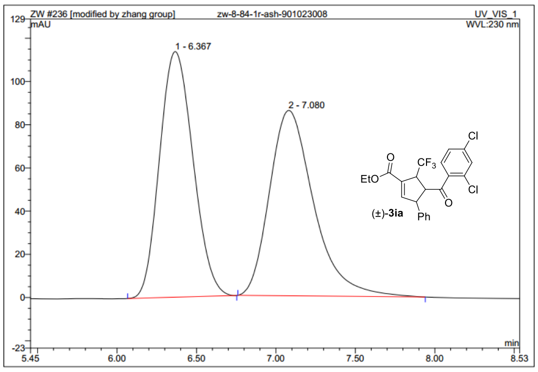

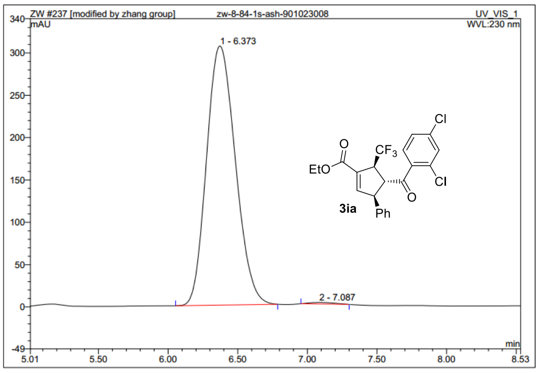

No.	Ret.Time min	Peak Name	Height mAU	Area mAU*min	Rel.Area %	Amount	Type
1	6.37	n.a.	113.726	27.785	50.04	n.a.	BMB*
2	7.08	n.a.	85.790	27.739	49.96	n.a.	BMB*
Total:			199.517	55.525	100.00	0.000	

No.	Ret.Time min	Peak Name	Height mAU	Area mAU*min	Rel.Area %	Amount	Type
1	6.37	n.a.	306.181	73.539	99.48	n.a.	BMB*
2	7.09	n.a.	1.854	0.388	0.52	n.a.	BMB*
Total:			308.035	73.926	100.00	0.000	

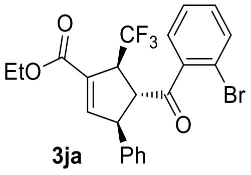

3ja; colorless oil; $[\alpha]_D^{20}$ = + 69.1 (c = 0.33, $CHCl_3$); ^1H NMR (400 MHz, $CDCl_3$): δ 7.63–7.61 (m, 1H), 7.33–7.26 (m, 2H), 7.21–7.19 (m, 4H), 6.89–6.83 (m, 3H), 4.57–4.49 (m, 1H), 4.37–4.20 (m, 3H), 4.14–4.12 (m, 1H), 1.34 (t, J = 7.20 Hz, 3H); ^{13}C NMR (100 MHz, $CDCl_3$): δ 199.85, 163.15, 147.14, 140.29, 139.82, 133.86, 132.15, 131.85 (d, J = 2.10 Hz), 129.00, 128.77, 127.59, 127.42, 127.31, 126.13 (q, J = 278.10 Hz), 119.57, 61.11, 58.29, 53.20, 49.80 (q, J = 29.20 Hz), 14.08; ^{19}F NMR (376 MHz, $CDCl_3$) δ = -67.97 ppm; Enantiomeric excess: 96%, determined by HPLC (Chiralpak AD-H, hexane/i-PrOH = 90/10; flow rate 0.8 ml/min; 25 °C; 230 nm), first peak: t_R = 7.08 min, second peak: t_R = 7.75 min; HRMS (ESI) m/z calcd. for $C_{22}H_{18}BrF_3NaO_3$ $[M+Na]^+$ = 489.0284, found = 489.0288.

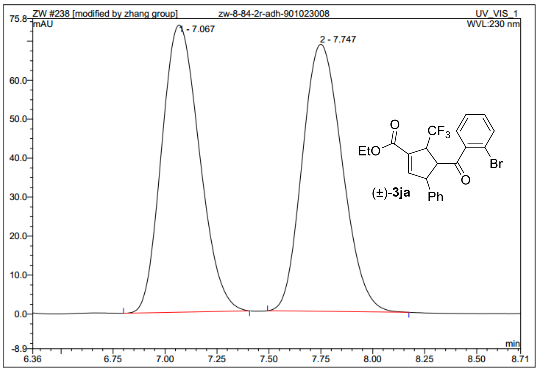

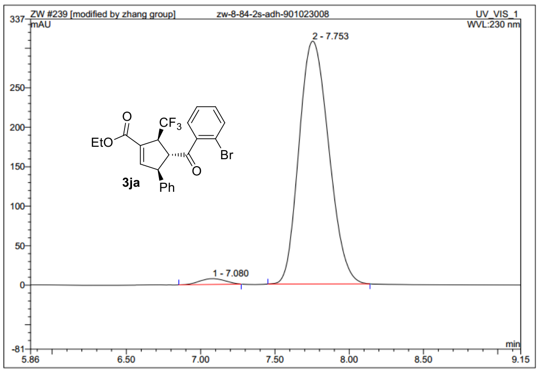

No.	Ret.Time min	Peak Name	Height mAU	Area mAU*min	Rel.Area %	Amount	Type
1	7.07	n.a.	73.701	15.543	50.42	n.a.	BMB*
2	7.75	n.a.	68.497	15.283	49.58	n.a.	BMB*
Total:			142.198	30.825	100.00	0.000	

No.	Ret.Time min	Peak Name	Height mAU	Area mAU*min	Rel.Area %	Amount	Type
1	7.08	n.a.	7.438	1.495	2.05	n.a.	BMB*
2	7.75	n.a.	307.709	71.568	97.95	n.a.	BMB*
Total:			315.147	73.062	100.00	0.000	

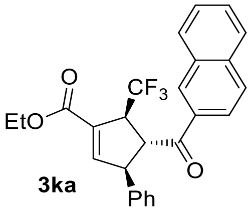

3ka; colorless oil; $[\alpha]_D^{20}$ = - 28.0 (c = 0.33, $CHCl_3$); ^1H NMR (400 MHz, $CDCl_3$): δ 7.96–7.94 (m, 1H), 7.90 (s, 1H), 7.84 (d, J = 8.40 Hz, 2H), 7.60–7.54 (m, 2H), 7.50–7.46 (m, 1H), 7.35–7.29 (m, 3H), 7.13–7.11 (m, 2H), 6.83 (s, 1H), 4.74–4.68 (m, 1H), 4.41–4.24 (m, 3H), 4.02–4.00 (m, 1H), 1.34 (t, J = 7.20 Hz, 3H); ^{13}C NMR (100

MHz, $CDCl_3$): δ 197.61, 163.37, 147.35, 140.41, 135.88, 132.34, 132.14, 132.03, 131.75, 129.67, 129.10, 128.99, 128.63, 128.30, 128.00, 127.72, 126.89, 126.44 (q, *J* = 278.00 Hz), 124.37, 61.12, 55.73, 54.57, 51.22 (q, *J* = 28.90 Hz), 14.13; ^{19}F NMR (376 MHz, $CDCl_3$) δ = -67.39 ppm; Enantiomeric excess: 91%, determined by HPLC (Chiralpak AD-H, hexane/*i*-PrOH = 90/10; flow rate 0.6 ml/min; 25 °C; 230 nm), first peak: t_R = 10.26 min, second peak: t_R = 15.57 min; HRMS (ESI) m/z calcd. for $C_{26}H_{21}F_3NaO_3$ $[M+Na]^+$ = 461.1335, found = 461.1344.

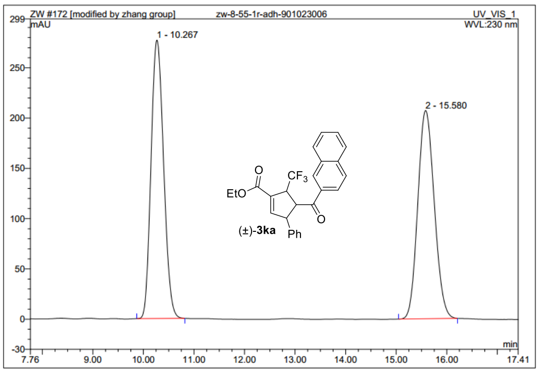

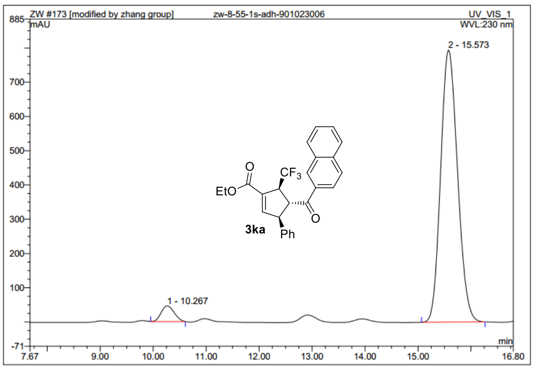

No.	Ret.Time min	Peak Name	Height mAU	Area mAU*min	Rel.Area %	Amount	Type
1	10.27	n.a.	276.883	79.781	50.54	n.a.	BMB*
2	15.58	n.a.	207.102	78.089	49.46	n.a.	BMB*
Total:			483.985	157.870	100.00	0.000	

No.	Ret.Time min	Peak Name	Height mAU	Area mAU*min	Rel.Area %	Amount	Type
1	10.27	n.a.	45.944	13.055	4.27	n.a.	MB*
2	15.57	n.a.	793.997	293.026	95.73	n.a.	BMB*
Total:			839.942	306.081	100.00	0.000	

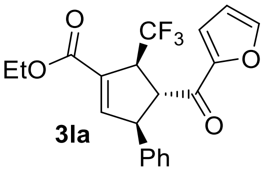

3la

3la; colorless oil; $[\alpha]_D^{20}$ = + 104.0 (*c* = 0.33, $CHCl_3$); ^1H NMR (500 MHz, $CDCl_3$): δ 7.57 (d, *J* = 1.0 Hz, 1H), 7.37–7.30 (m, 3H), 7.16–7.15 (m, 2H), 6.94 (d, *J* = 3.50 Hz, 1H), 6.87 (s, 1H), 6.50–6.49 (m, 1H), 4.49–4.43 (m, 1H), 4.37–4.23 (m, 2H), 4.15–4.14 (m, 1H), 4.02–4.00 (m, 1H), 1.34 (t, *J* = 7.00 Hz, 3H); ^{13}C NMR (125 MHz, $CDCl_3$): δ 186.43, 163.16, 151.15, 147.79, 147.63, 140.34, 131.82, 128.88, 127.82, 127.74, 126.08 (q, *J* = 278.13 Hz), 119.79, 112.55, 61.05, 55.20, 54.68, 51.17 (q, *J* = 29.13 Hz), 14.04; ^{19}F NMR (376 MHz, $CDCl_3$) δ = -67.74 ppm; Enantiomeric excess: 94%, determined by HPLC (Chiralpak AD-H, hexane/*i*-PrOH = 90/10; flow rate 1.0 ml/min; 25 °C; 230 nm), first peak: t_R = 6.18 min, second peak: t_R = 9.40 min; HRMS (ESI) m/z calcd. for $C_{20}H_{17}F_3NaO_4$ $[M+Na]^+$ = 401.0971, found = 401.0974.

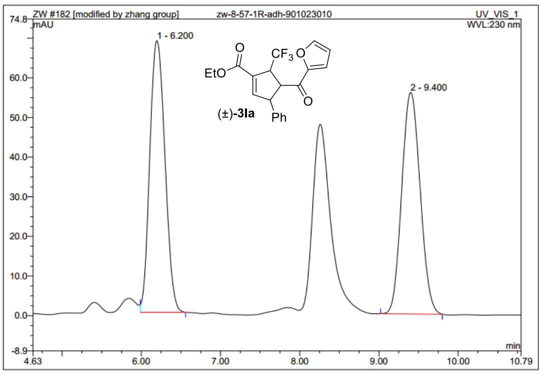

No.	Ret.Time min	Peak Name	Height mAU	Area mAU*min	Rel.Area %	Amount	Type
1	6.20	n.a.	68.487	15.204	50.29	n.a.	MB*
2	9.40	n.a.	55.899	15.031	49.71	n.a.	BMB*
Total:			124.386	30.235	100.00	0.000	

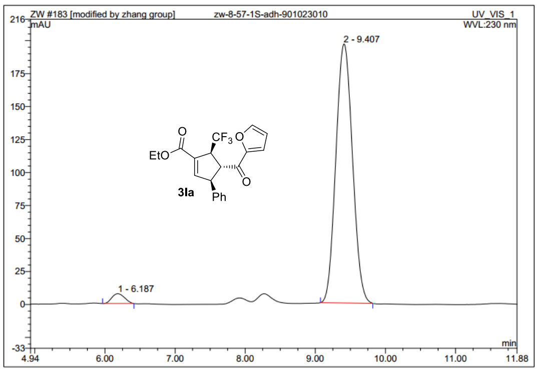

No.	Ret.Time min	Peak Name	Height mAU	Area mAU*min	Rel.Area %	Amount	Type
1	6.19	n.a.	7.459	1.565	2.83	n.a.	BMB*
2	9.41	n.a.	196.351	53.763	97.17	n.a.	BMB*
Total:			203.810	55.328	100.00	0.000	

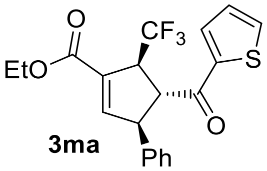

3ma

3ma; colorless oil; $[\alpha]_D^{20}$ = + 90.7 (c = 0.33, $CHCl_3$); ^{1}H NMR (500 MHz, $CDCl_3$): δ 7.71–7.70 (m, 1H), 7.38–7.33 (m, 3H), 7.20–7.19 (m, 1H), 7.17–7.15 (m, 2H), 7.02–7.00 (m, 1H), 6.87 (s, 1H), 4.55–4.49 (m, 1H), 4.38–4.24 (m, 2H), 4.13–4.11 (m, 1H), 4.07–4.05 (m, 1H), 1.35 (t, J = 7.00 Hz, 3H); ^{13}C NMR (125 MHz, $CDCl_3$): δ 190.90, 163.16, 147.55, 142.77, 140.25, 135.64, 133.57, 131.82, 128.97, 128.24, 127.92, 127.87, 126.13 (q, J = 278.13 Hz), 61.05, 55.68, 55.50, 51.65 (q, J = 29.13 Hz), 14.04; ^{19}F NMR (376 MHz, $CDCl_3$) δ = -67.58 ppm; Enantiomeric excess: 95%, determined by HPLC (Chiralpak AD-H, hexane/*i*-PrOH = 90/10; flow rate 1.0 ml/min; 25 °C; 230 nm), first peak: t_R = 5.72 min, second peak: t_R = 7.49 min; HRMS (ESI) m/z calcd. for $C_{20}H_{17}F_3NaO_3S$ $[M+Na]^+$ = 417.0743, found = 417.0748.

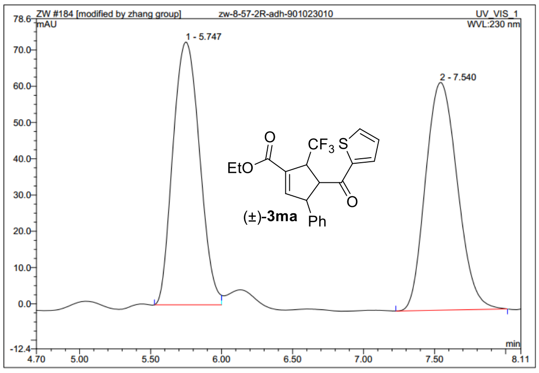

No.	Ret.Time min	Peak Name	Height mAU	Area mAU*min	Rel.Area %	Amount	Type
1	5.75	n.a.	72.437	15.807	49.16	n.a.	BM *
2	7.54	n.a.	62.809	16.347	50.84	n.a.	BMB*
Total:			135.246	32.154	100.00	0.000	

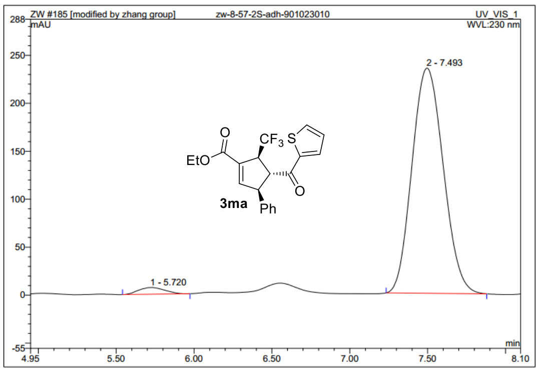

No.	Ret.Time min	Peak Name	Height mAU	Area mAU*min	Rel.Area %	Amount	Type
1	5.72	n.a.	7.008	1.368	2.44	n.a.	BMB*
2	7.49	n.a.	234.839	54.744	97.56	n.a.	BMB*
Total:			241.847	56.112	100.00	0.000	

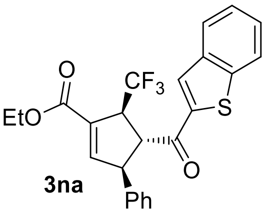

3na

3na; colorless oil; $[\alpha]_D^{20}$ = + 46.3 (c = 0.33, $CHCl_3$); ^1H NMR (400 MHz, $CDCl_3$): δ 7.85–7.83 (m, 1H), 7.64 (d, J = 8.00 Hz, 1H), 7.47–7.43 (m, 1H), 7.37–7.30 (m, 5H), 7.17–7.14 (m, 2H), 6.86 (s, 1H), 4.61–4.53 (m, 1H), 4.37–4.26 (m, 2H), 4.18–4.15 (m, 1H), 4.12–4.10 (m, 1H), 1.33 (t, J = 7.20 Hz, 3H); ^{13}C NMR (100 MHz, $CDCl_3$): δ 192.26, 163.13, 147.34, 143.13, 142.04, 140.25, 138.74, 131.91, 131.22, 129.04, 128.04, 127.96, 127.93, 126.20, 126.14 (q, J = 278.00 Hz), 125.07, 122.86, 61.08, 55.72, 55.52, 51.53 (q, J = 29.10 Hz), 14.04; ^{19}F NMR (376 MHz, $CDCl_3$) δ = -67.55 ppm; Enantiomeric excess: 94%, determined by HPLC (Chiralpak AD-H, hexane/*i*-PrOH = 90/10; flow rate 0.8 ml/min; 25 °C; 230 nm), first peak: t_R = 7.64 min, second peak: t_R = 9.56 min; HRMS (ESI) m/z calcd. for $C_{24}H_{19}F_3NaO_3S$ $[M+Na]^+$ = 467.0899, found = 467.0900.

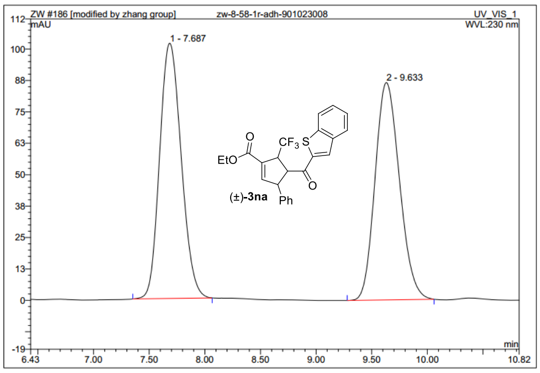

No.	Ret.Time min	Peak Name	Height mAU	Area mAU*min	Rel.Area %	Amount	Type
1	7.69	n.a.	101.581	22.857	50.96	n.a.	BMB*
2	9.63	n.a.	86.490	21.993	49.04	n.a.	BMB*
Total:			188.071	44.850	100.00	0.000	

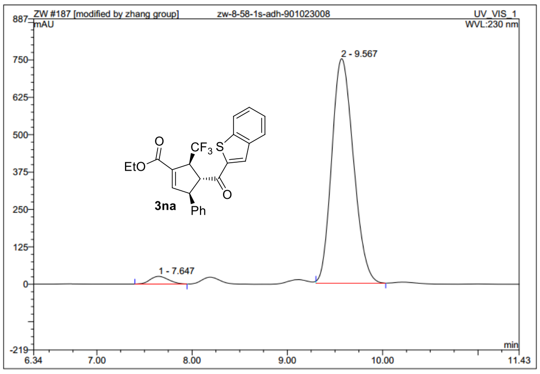

No.	Ret.Time min	Peak Name	Height mAU	Area mAU*min	Rel.Area %	Amount	Type
1	7.65	n.a.	25.897	5.813	2.85	n.a.	BMB*
2	9.57	n.a.	750.026	198.071	97.15	n.a.	MB*
Total:			775.923	203.884	100.00	0.000	

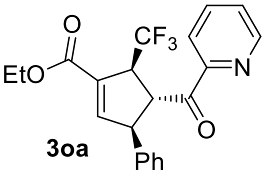

3oa

3oa; colorless oil; $[\alpha]_D^{20}$ = + 50.7 (c = 0.33, $CHCl_3$); ^1H NMR (500 MHz, $CDCl_3$): δ 8.50 (d, J = 4.00 Hz, 1H), 8.13 (d, J = 8.00 Hz, 1H), 7.89–7.86 (m, 1H), 7.49–7.46 (m, 1H), 7.33–7.28 (m, 3H), 7.18–7.16 (m, 2H), 6.91 (s, 1H), 5.01–4.99 (m, 1H),

4.54–4.47 (m, 1H), 4.39–4.25 (m, 2H), 4.07 (br, 1H), 1.36 (t, J = 7.00 Hz, 3H); ^{13}C NMR (125 MHz, $CDCl_3$): δ 199.28, 163.40, 151.56, 148.89, 147.76, 140.44, 136.93, 132.07, 128.40, 128.10, 127.52, 127.26, 126.24 (q, J = 278.25 Hz), 123.14, 60.98, 54.87, 52.42, 50.55 (q, J = 29.00 Hz), 14.09; ^{19}F NMR (376 MHz, $CDCl_3$) δ = -67.38 ppm; Enantiomeric excess: 82%, determined by HPLC (Chiralpak AD-H, hexane/*i*-PrOH = 90/10; flow rate 1.0 ml/min; 25 °C; 230 nm), first peak: t_R = 6.22 min, second peak: t_R = 9.91 min; HRMS (ESI) m/z calcd. for $C_{21}H_{19}F_3NO_3$ $[M+Na]^+$ = 390.1312, found = 390.1318.

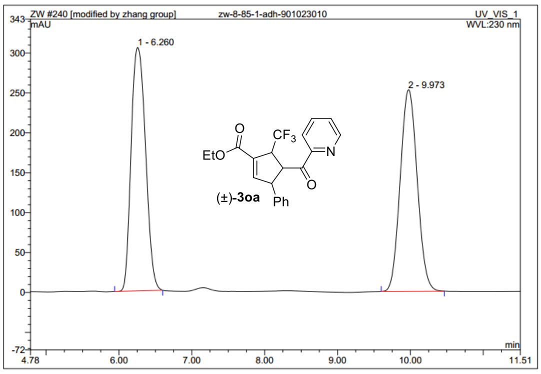

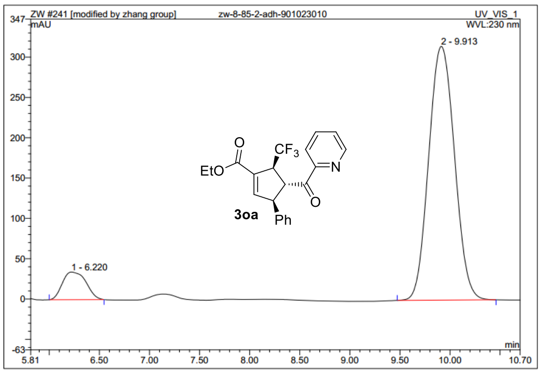

No.	Ret.Time min	Peak Name	Height mAU	Area mAU*min	Rel.Area %	Amount	Type
1	6.26	n.a.	304.956	70.028	50.64	n.a.	BMB*
2	9.97	n.a.	252.791	68.270	49.36	n.a.	BMB*
Total:			557.747	138.298	100.00	0.000	

No.	Ret.Time min	Peak Name	Height mAU	Area mAU*min	Rel.Area %	Amount	Type
1	6.22	n.a.	34.265	9.319	9.01	n.a.	BMB*
2	9.91	n.a.	314.708	94.063	90.99	n.a.	BMB*
Total:			348.973	103.382	100.00	0.000	

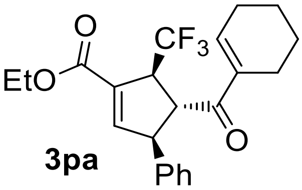

3pa; colorless oil; $[\alpha]_D^{20}$ = + 54.5 (c = 0.33, $CHCl_3$); ^{1}H NMR (400 MHz, $CDCl_3$): δ 7.37–7.27 (m, 3H), 7.16–7.14 (m, 2H), 6.77 (s, 1H), 6.33–6.31 (m, 1H), 4.47–4.38 (m, 1H), 4.35–4.19 (m, 2H), 3.93–3.90 (m, 1H), 3.84–3.81 (m, 1H), 2.33–1.93 (m, 4H), 1.65–1.54 (m, 4H), 1.32 (t, J = 6.80 Hz, 3H); ^{13}C NMR (100 MHz, $CDCl_3$): δ 198.57, 163.37, 147.38, 143.51, 140.60, 137.88, 132.04, 128.85, 128.00, 126.33 (q, J = 277.00 Hz), 60.95, 55.71, 52.81, 51.25 (q, J = 28.70 Hz), 26.11, 23.55, 21.79, 21.32, 14.05; ^{19}F NMR (376 MHz, $CDCl_3$) δ = -67.65 ppm; Enantiomeric excess: 92%, determined by HPLC (Chiralpak AD-H, hexane/*i*-PrOH = 90/10; flow rate 0.5 ml/min; 25 °C; 230 nm), first peak: t_R = 9.18 min, second peak: t_R = 12.20 min; HRMS (ESI) m/z calcd. for $C_{22}H_{23}F_3NaO_3$ $[M+Na]^+$ = 415.1491, found = 415.1496.

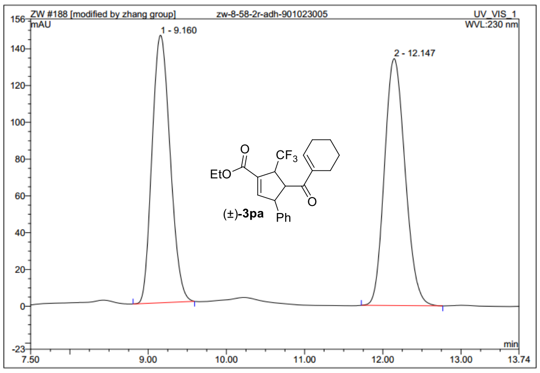

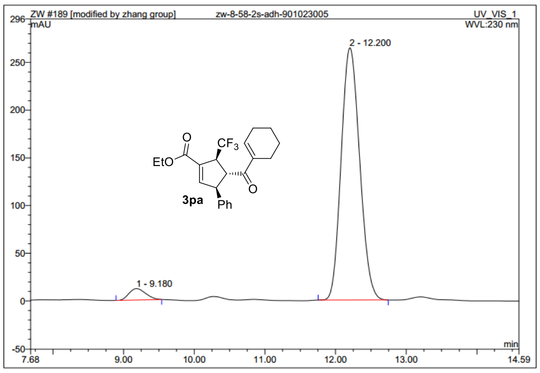

No.	Ret.Time min	Peak Name	Height mAU	Area mAU*min	Rel.Area %	Amount	Type
1	9.16	n.a.	145.469	39.026	48.69	n.a.	BMB*
2	12.15	n.a.	134.260	41.117	51.31	n.a.	BMB*
Total:			279.729	80.143	100.00	0.000	

No.	Ret.Time min	Peak Name	Height mAU	Area mAU*min	Rel.Area %	Amount	Type
1	9.18	n.a.	11.985	3.293	3.95	n.a.	BMB*
2	12.20	n.a.	264.321	80.019	96.05	n.a.	BMB*
Total:			276.306	83.312	100.00	0.000	

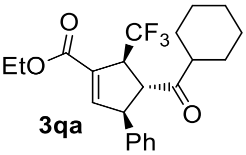

3qa

3qa; colorless oil; $[\alpha]_D^{20}$ = + 116.8 (c = 0.33, $CHCl_3$); ^1H NMR (500 MHz, $CDCl_3$): δ 7.41–7.38 (m, 2H), 7.35–7.32 (m, 1H), 7.23–7.21 (m, 2H), 6.79 (s, 1H), 4.35–4.21 (m, 3H), 4.06–4.04 (m, 1H), 3.55–3.53 (m, 1H), 2.42–2.36 (m, 1H), 1.79–1.72 (m, 4H), 1.46–1.16 (m, 9H); ^{13}C NMR (125 MHz, $CDCl_3$): δ 210.89, 163.23, 147.67, 140.83, 131.54, 129.09, 127.80, 127.62, 126.17 (q, J = 278.00 Hz), 61.01, 57.01, 53.94, 50.70 (q, J = 28.88 Hz), 50.10, 28.57, 28.02, 25.59, 25.49, 25.35, 14.06; ^{19}F NMR (376 MHz, $CDCl_3$) δ = -68.02 ppm; Enantiomeric excess: 91%, determined by HPLC (Chiralpak AD-H, hexane/i-PrOH = 90/10; flow rate 0.4 ml/min; 25 °C; 230 nm), first peak: t_R = 10.63 min, second peak: t_R = 12.86 min; HRMS (ESI) m/z calcd. for $C_{22}H_{25}F_3NaO_3$ $[M+Na]^+$ = 417.1648, found = 417.1653.

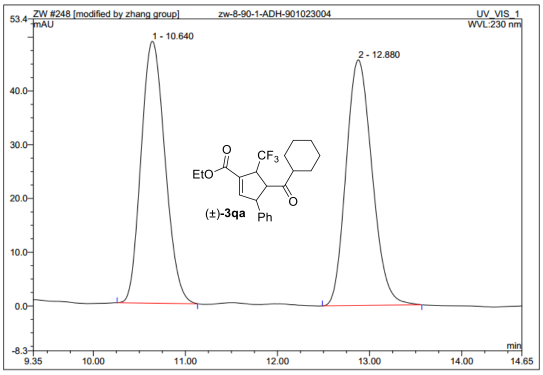

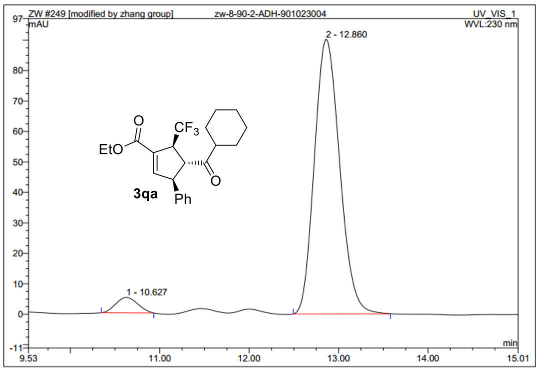

No.	Ret.Time min	Peak Name	Height mAU	Area mAU*min	Rel.Area %	Amount	Type
1	10.64	n.a.	48.712	14.676	49.50	n.a.	BMB*
2	12.88	n.a.	45.688	14.975	50.50	n.a.	BMB*
Total:			94.399	29.651	100.00	0.000	

No.	Ret.Time min	Peak Name	Height mAU	Area mAU*min	Rel.Area %	Amount	Type
1	10.63	n.a.	5.072	1.432	4.63	n.a.	BMB*
2	12.86	n.a.	90.168	29.537	95.37	n.a.	BMB*
Total:			95.241	30.970	100.00	0.000	

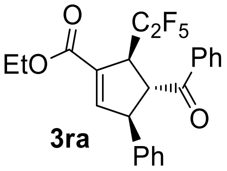

3ra

3ra; colorless oil; $[\alpha]_D^{20}$ = + 33.9 (c = 0.33, $CHCl_3$); ^1H NMR (500 MHz, $CDCl_3$): δ 7.72–7.70 (m, 2H), 7.61–7.58 (m, 1H), 7.41–7.38 (m, 2H), 7.35–7.33 (m, 3H), 7.10–7.08 (m, 2H), 6.77–6.76 (m, 1H), 4.84–4.77 (m, 1H), 4.38–4.26 (m, 3H), 3.92–3.91 (m, 1H), 1.36 (t, J = 7.00 Hz, 3H); ^{13}C NMR (125 MHz, $CDCl_3$): δ 197.87, 163.78, 146.36, 139.96, 135.00, 133.87, 132.20, 129.18, 128.91, 128.64, 128.18, 127.92, 122.47 (t, J = 36.25 Hz), 120.19 (t, J = 36.50 Hz), 118.20–116.58 (m), 115.63–114.55 (m), 112.94 (t, J = 36.75 Hz), 61.11, 56.17, 53.47, 48.33 (t, J = 20.50 Hz), 14.02; ^{19}F NMR (376 MHz, $CDCl_3$) δ = -82.94, -116.11 (q) ppm; Enantiomeric excess: 94%, determined by HPLC (Chiralpak AD-H, hexane/i-PrOH = 90/10; flow rate 0.6 ml/min; 25 °C; 230 nm), first peak: t_R = 7.82 min, second peak: t_R = 11.14 min; HRMS (ESI) m/z calcd. for $C_{23}H_{19}F_5NaO_3$ $[M+Na]^+$ = 461.1147, found = 461.1154.

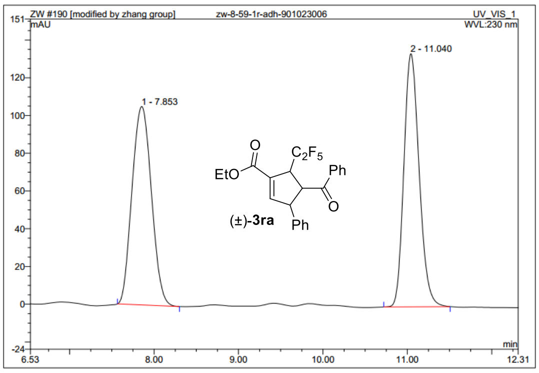

No.	Ret.Time min	Peak Name	Height mAU	Area mAU*min	Rel.Area %	Amount	Type
1	7.85	n.a.	105.141	27.970	49.84	n.a.	BMB*
2	11.04	n.a.	134.095	28.150	50.16	n.a.	BMB*
Total:			239.236	56.120	100.00	0.000	

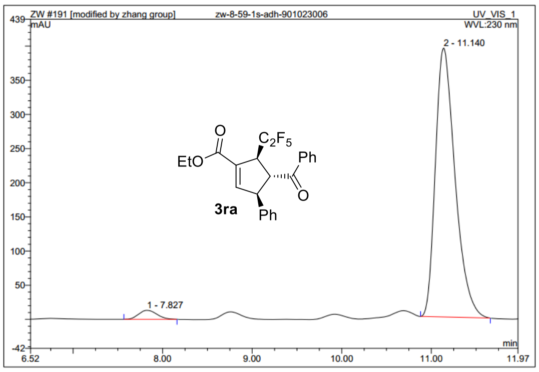

No.	Ret.Time min	Peak Name	Height mAU	Area mAU*min	Rel.Area %	Amount	Type
1	7.83	n.a.	13.230	3.105	3.00	n.a.	BMB*
2	11.14	n.a.	393.574	100.313	97.00	n.a.	BMB*
Total:			406.804	103.418	100.00	0.000	

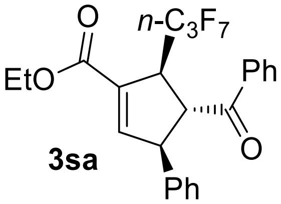

3sa

3sa; colorless oil; $[\alpha]_D^{20}$ = + 18.8 (c = 0.33, $CHCl_3$); ^1H NMR (500 MHz, $CDCl_3$): δ

7.73 (d, J = 7.50 Hz, 2H), 7.62–7.59 (m, 1H), 7.42–7.39 (m, 2H), 7.35–7.33 (m, 3H), 7.10–7.08 (m, 2H), 6.77–6.76 (m, 1H), 4.95–4.90 (m, 1H), 4.39–4.25 (m, 3H), 3.90 (br, 1H), 1.36 (t, J = 7.50 Hz, 3H); ^{13}C NMR (125 MHz, $CDCl_3$): δ 197.72, 163.83, 146.17, 139.93, 134.96, 133.88, 132.32, 129.18, 128.92, 128.67, 128.21, 127.93, 121.08, 119.24–118.52 (m), 117.19–116.23 (m), 115.14–114.21 (m), 111.80–110.90 (m), 109.70–108.80 (m), 107.59–106.69 (m), 61.08, 56.49, 53.39, 48.39 (t, J = 20.63 Hz), 14.00; ^{19}F NMR (376 MHz, $CDCl_3$) δ = -80.38 (t), -111.06–-115.15 (m), -124.53–-126.91 (m) ppm; Enantiomeric excess: 94%, determined by HPLC (Chiralpak AD-H, hexane/*i*-PrOH = 90/10; flow rate 0.6 ml/min; 25 °C; 230 nm), first peak: t_R = 7.24 min, second peak: t_R = 9.74 min; HRMS (ESI) m/z calcd. for $C_{24}H_{19}F_7NaO_3$ $[M+Na]^+$ = 511.1115, found = 511.1125.

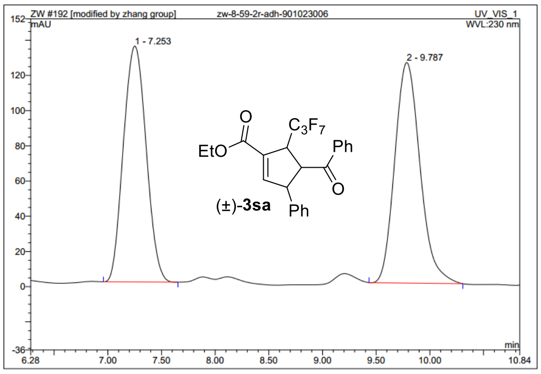

No.	Ret.Time min	Peak Name	Height mAU	Area mAU*min	Rel.Area %	Amount	Type
1	7.25	n.a.	133.988	32.976	49.09	n.a.	BMB*
2	9.79	n.a.	125.257	34.205	50.91	n.a.	BMB*
Total:			259.246	67.181	100.00	0.000	

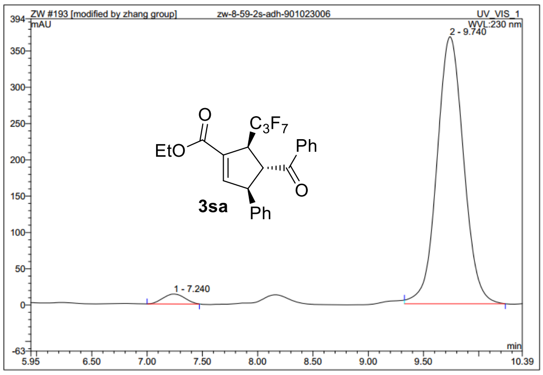

No.	Ret.Time min	Peak Name	Height mAU	Area mAU*min	Rel.Area %	Amount	Type
1	7.24	n.a.	13.696	3.124	2.95	n.a.	BMB*
2	9.74	n.a.	368.014	102.761	97.05	n.a.	MB*
Total:			381.709	105.885	100.00	0.000	

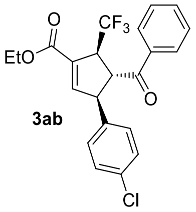

3ab

3ab; colorless oil; $[\alpha]_D^{20}$ = + 137.4 (c = 0.33, $CHCl_3$); ^{1}H NMR (500 MHz, $CDCl_3$): δ 7.74–7.72 (m, 2H), 7.62–7.59 (m, 1H), 7.43–7.40 (m, 2H), 7.32–7.30 (m, 2H), 7.04–7.02 (m, 2H), 6.80 (s, 1H), 4.59–4.53 (m, 1H), 4.38–4.32 (m, 1H), 4.30–4.25 (m, 1H), 4.21–4.19 (m, 1H), 4.02–4.01 (m, 1H), 1.35 (t, J = 7.00 Hz, 3H); ^{13}C NMR (125 MHz, $CDCl_3$): δ 197.85, 163.08, 146.74, 138.68, 135.09, 133.99, 133.77, 132.39, 129.35, 129.10, 129.05, 128.73, 126.18 (q, J = 278.00 Hz), 61.15, 54.61, 54.84, 51.25

(q, J = 28.88 Hz), 14.05; ^{19}F NMR (376 MHz, $CDCl_3$) δ = -67.53 ppm; Enantiomeric excess: 94%, determined by HPLC (Chiralpak AD-H, hexane/*i*-PrOH = 90/10; flow rate 0.8 ml/min; 25 °C; 230 nm), first peak: t_R = 6.88 min, second peak: t_R = 10.44 min; HRMS (ESI) m/z calcd. for $C_{22}H_{18}ClF_3NaO_3$ $[M+Na]^+$ = 445.0789, found = 445.0792.

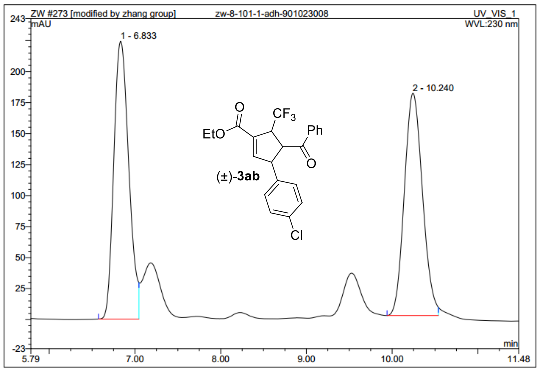

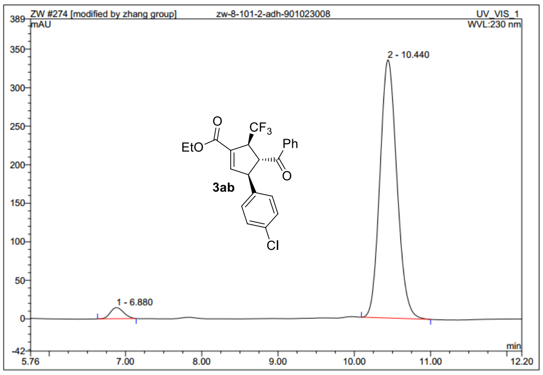

No.	Ret.Time min	Peak Name	Height mAU	Area mAU*min	Rel.Area %	Amount	Type
1	6.83	n.a.	224.092	45.257	50.74	n.a.	BM *
2	10.24	n.a.	179.301	43.945	49.26	n.a.	BM *
Total:			403.393	89.202	100.00	0.000	

No.	Ret.Time min	Peak Name	Height mAU	Area mAU*min	Rel.Area %	Amount	Type
1	6.88	n.a.	14.392	2.895	3.24	n.a.	BMB*
2	10.44	n.a.	335.048	86.353	96.76	n.a.	BMB*
Total:			349.440	89.248	100.00	0.000	

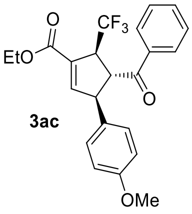

3ac

3ac; colorless oil; $[\alpha]_D^{20}$ = + 121.6 (c = 0.33, $CHCl_3$); ^{1}H NMR (500 MHz, $CDCl_3$): δ 7.73 (d, J = 7.50 Hz, 2H), 7.60–7.57 (m, 1H), 7.41–7.38 (m, 2H), 7.02 (d, J = 8.50 Hz, 2H), 6.87 (d, J = 8.50 Hz, 2H), 6.80 (s, 1H), 4.63–4.57 (m, 1H), 4.38–4.31 (m, 1H), 4.30–4.25 (m, 1H), 4.24–4.21 (m, 1H), 3.96–3.95 (m, 1H), 3.83 (s, 3H), 1.35 (t, J = 7.00 Hz, 3H); ^{13}C NMR (125 MHz, $CDCl_3$): δ 198.19, 163.32, 159.18, 147.69, 135.20, 133.79, 132.18, 131.53, 129.15, 128.61, 126.32 (q, J = 278.00 Hz), 114.26, 61.01, 55.26, 54.83, 54.25, 51.07 (q, J = 28.88 Hz), 14.06; ^{19}F NMR (376 MHz, $CDCl_3$) δ = -67.50 ppm; Enantiomeric excess: 93%, determined by HPLC (Chiralpak AD-H, hexane/*i*-PrOH = 90/10; flow rate 0.8 ml/min; 25 °C; 230 nm), first peak: t_R = 7.74 min, second peak: t_R = 14.36 min; HRMS (ESI) m/z calcd. for $C_{23}H_{21}F_3NaO_4$ $[M+Na]^+$ = 441.1284, found = 441.1289.

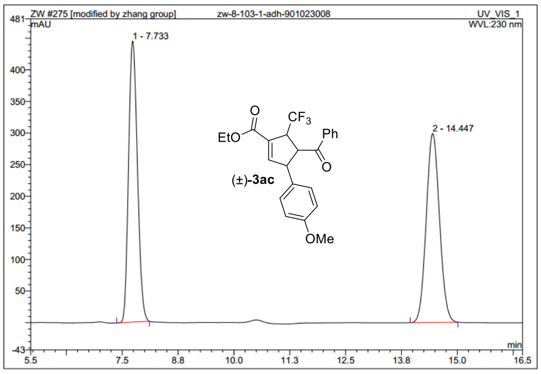

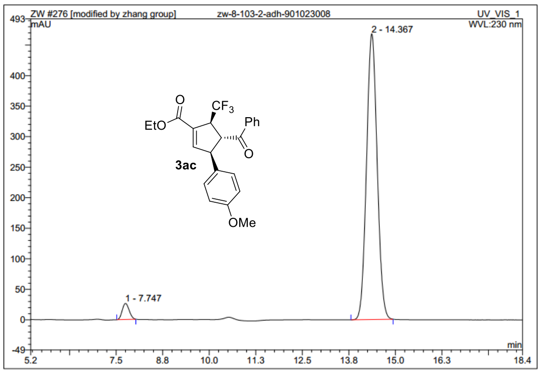

No.	Ret.Time min	Peak Name	Height mAU	Area mAU*min	Rel.Area %	Amount	Type
1	7.73	n.a.	444.424	102.157	49.85	n.a.	BMB*
2	14.45	n.a.	298.874	102.790	50.15	n.a.	BMB*
Total:			743.298	204.948	100.00	0.000	

No.	Ret.Time min	Peak Name	Height mAU	Area mAU*min	Rel.Area %	Amount	Type
1	7.75	n.a.	26.271	5.700	3.54	n.a.	BMB*
2	14.37	n.a.	468.067	155.284	96.46	n.a.	BMB*
Total:			494.338	160.984	100.00	0.000	

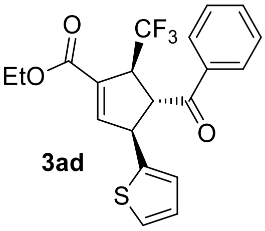

3ad

3ad; colorless oil; $[\alpha]_D^{20}$ = + 52.5 (c = 0.33, $CHCl_3$); ^{1}H NMR (500 MHz, $CDCl_3$): δ 7.87–7.85 (m, 2H), 7.64–7.61 (m, 1H), 7.47–7.44 (m, 2H), 7.29–7.28 (m, 1H), 7.00–6.98 (m, 1H), 6.86–6.84 (m, 2H), 4.55–4.49 (m, 1H), 4.38–4.33 (m, 3H), 4.29–4.24 (m, 1H), 1.35 (t, J = 7.50 Hz, 3H); ^{13}C NMR (125 MHz, $CDCl_3$): δ 197.62, 163.07, 146.57, 142.68, 134.96, 133.98, 131.56, 129.14, 128.75, 127.17, 126.07, 126.06 (q, J = 276.75 Hz), 125.33, 61.13, 54.10, 51.01 (q, J = 29.00 Hz), 49.98, 14.06; ^{19}F NMR (376 MHz, $CDCl_3$) δ = -67.53 ppm; Enantiomeric excess: 95%, determined by HPLC (Chiralpak AD-H, hexane/i-PrOH = 90/10; flow rate 0.8 ml/min; 25 °C; 230 nm), first peak: t_R = 7.18 min, second peak: t_R = 9.66 min; HRMS (ESI) m/z calcd. for $C_{20}H_{17}F_3NaO_3S$ $[M+Na]^+$ = 417.0743, found = 417.0745.

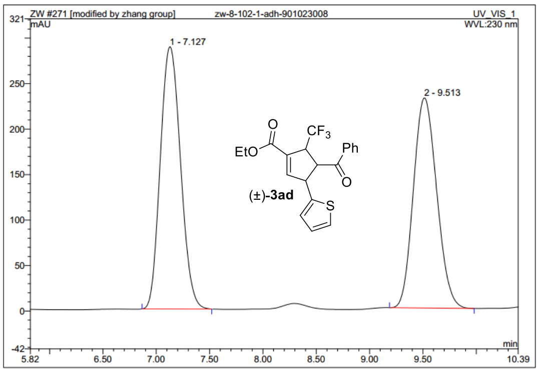

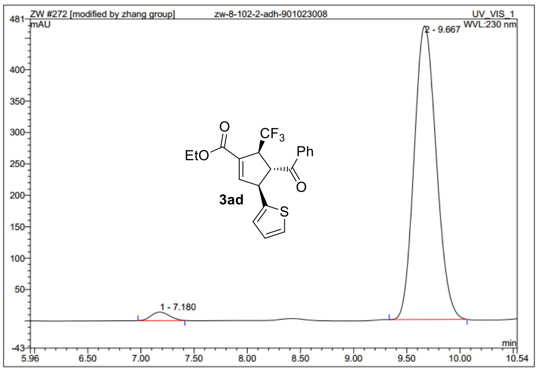

No.	Ret.Time min	Peak Name	Height mAU	Area mAU*min	Rel.Area %	Amount	Type
1	7.13	n.a.	288.314	63.366	52.01	n.a.	BMB*
2	9.51	n.a.	231.090	58.470	47.99	n.a.	BMB*
Total:			519.404	121.836	100.00	0.000	

No.	Ret.Time min	Peak Name	Height mAU	Area mAU*min	Rel.Area %	Amount	Type
1	7.18	n.a.	13.437	2.653	2.30	n.a.	BMB*
2	9.67	n.a.	467.211	112.793	97.70	n.a.	BMB*
Total:			480.647	115.446	100.00	0.000	

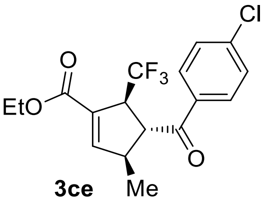

3ce

3ce; colorless oil; $[\alpha]_D^{20}$ = - 63.7 (*c* = 0.33, $CHCl_3$); ^{1}H NMR (500 MHz, $CDCl_3$): δ 7.94–7.91 (m, 2H), 7.53–7.50 (m, 2H), 6.80 (s, 1H), 4.40–4.20 (m, 3H), 3.84–3.83 (m, 1H), 2.99–2.94 (m, 1H), 1.37 (d, *J* = 7.00 Hz, 3H), 1.33 (t, *J* = 7.00 Hz, 3H); ^{13}C NMR (125 MHz, $CDCl_3$): δ 196.67, 163.24, 150.32, 140.35, 133.60, 130.24, 130.00, 129.29, 126.21 (q, *J* = 277.88 Hz), 60.94, 52.38, 50.36 (q, *J* = 29.00 Hz), 44.68, 19.07, 14.07; ^{19}F NMR (376 MHz, $CDCl_3$) δ = -68.10 ppm; Enantiomeric excess: 95%, determined by HPLC (Chiralpak IE, hexane/*i*-PrOH = 97/03; flow rate 1.0 ml/min; 25 °C; 230 nm), first peak: t_R = 11.52 min, second peak: t_R = 15.77 min; HRMS (ESI) m/z calcd. for $C_{17}H_{16}ClF_3NaO_3$ $[M+Na]^+$ = 383.0632, found = 383.0633.

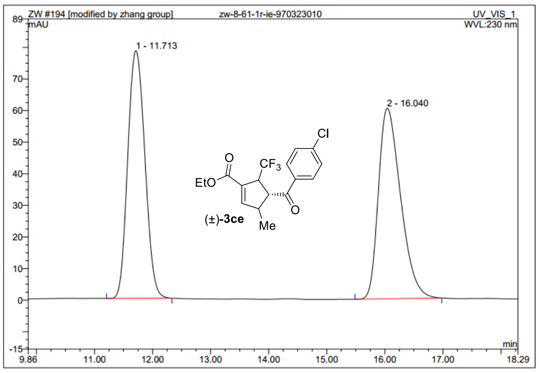

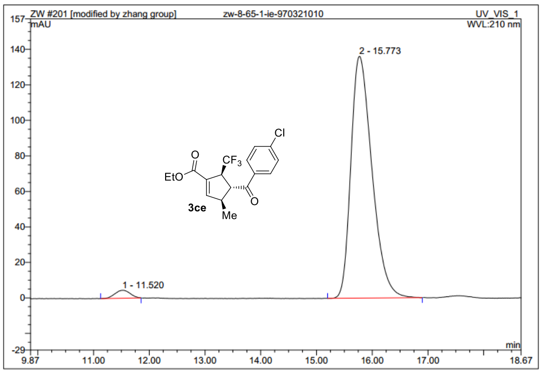

No.	Ret.Time min	Peak Name	Height mAU	Area mAU*min	Rel.Area %	Amount	Type
1	11.71	n.a.	78.409	26.789	50.07	n.a.	BMB*
2	16.04	n.a.	60.317	26.713	49.93	n.a.	BMB*
Total:			138.726	53.502	100.00	0.000	

No.	Ret.Time min	Peak Name	Height mAU	Area mAU*min	Rel.Area %	Amount	Type
1	11.52	n.a.	4.501	1.423	2.33	n.a.	BMB*
2	15.77	n.a.	136.232	59.712	97.67	n.a.	BMB*
Total:			140.732	61.135	100.00	0.000	

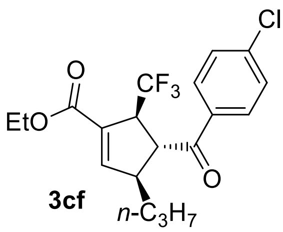

3cf; colorless oil; $[\alpha]_D^{20}$ = - 31.4 (*c* = 0.33, $CHCl_3$); ^{1}H NMR (500 MHz, $CDCl_3$): δ 7.94–7.91 (m, 2H), 7.52–7.50 (m, 2H), 6.93 (s, 1H), 4.33–4.27 (m, 1H), 4.24–4.20 (m, 2H), 3.89–3.88 (m, 1H), 2.95–2.91 (m, 1H), 1.72–1.65 (m, 2H), 1.48–1.39 (m, 1H), 1.37–1.31 (m, 4H), 0.90 (t, *J* = 7.50 Hz, 3H); ^{13}C NMR (125 MHz, $CDCl_3$): δ 197.47, 163.23, 149.52, 140.33, 133.72, 130.41, 130.01, 129.25, 126.19 (q, *J* = 278.00 Hz), 60.90, 50.94, 50.85 (q, *J* = 28.50 Hz), 49.87, 36.52, 20.96, 14.07, 13.87; ^{19}F NMR (376 MHz, $CDCl_3$) δ = -67.88 ppm; Enantiomeric excess: 92%, determined by HPLC (Chiralpak IE, hexane/*i*-PrOH = 97/03; flow rate 1.0 ml/min; 25 °C; 230 nm), first peak: t_R = 9.78 min, second peak: t_R = 11.91 min; HRMS (ESI) m/z calcd. for $C_{19}H_{20}ClF_3NaO_3$ $[M+Na]^+$ = 411.0945, found = 411.0946.

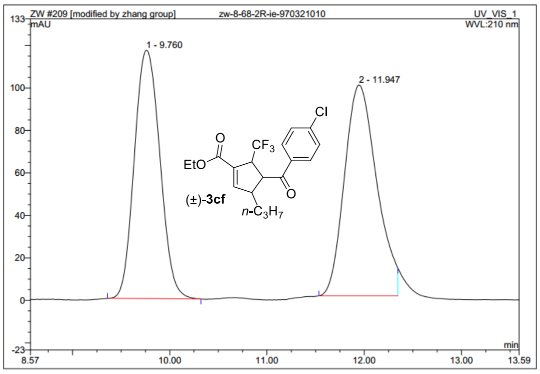

No.	Ret.Time min	Peak Name	Height mAU	Area mAU*min	Rel.Area %	Amount	Type
1	9.76	n.a.	116.986	35.680	48.35	n.a.	BMB*
2	11.95	n.a.	99.336	38.119	51.65	n.a.	BM *
Total:			216.322	73.799	100.00	0.000	

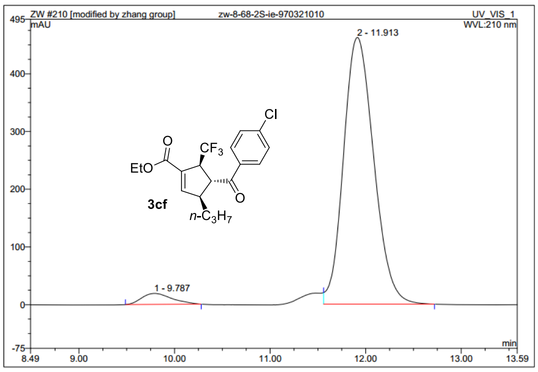

No.	Ret.Time min	Peak Name	Height mAU	Area mAU*min	Rel.Area %	Amount	Type
1	9.79	n.a.	18.986	6.966	3.95	n.a.	BMB*
2	11.91	n.a.	462.060	169.430	96.05	n.a.	MB*
Total:			481.046	176.396	100.00	0.000	

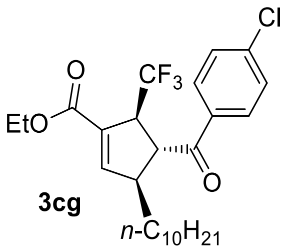

3cg; colorless oil; $[\alpha]_D^{20}$ = - 27.2 (*c* = 0.33, $CHCl_3$); ^{1}H NMR (400 MHz, $CDCl_3$): δ 7.90 (d, *J* = 8.40 Hz, 2H), 7.49 (d, *J* = 8.80 Hz, 2H), 6.92 (s, 1H), 4.32–4.16 (m, 3H), 3.88–3.86 (m, 1H), 2.90–2.87 (m, 1H), 1.71–1.64 (m, 2H), 1.33–1.22 (m, 19H), 0.88

(t, J = 6.80 Hz, 3H); ^{13}C NMR (100 MHz, CDCl$_3$): δ 197.50, 163.25, 149.57, 140.33, 133.74, 130.40, 130.01, 129.24, 126.20 (q, J = 277.90 Hz), 60.90, 50.96, 50.82 (q, J = 28.80 Hz), 50.10, 34.36, 31.84, 29.51, 29.46, 29.33, 29.26, 27.72, 22.64, 14.07; ^{19}F NMR (376 MHz, CDCl$_3$) δ = -67.87 ppm; Enantiomeric excess: 96%, determined by HPLC (Chiralpak IE, hexane/*i*-PrOH = 97/03; flow rate 1.0 ml/min; 25 °C; 230 nm), first peak: t_R = 7.00 min, second peak: t_R = 8.33 min; HRMS (ESI) m/z calcd. for $C_{26}H_{34}ClF_3NaO_3$ [M+Na]$^+$ = 509.2041, found = 509.2044.

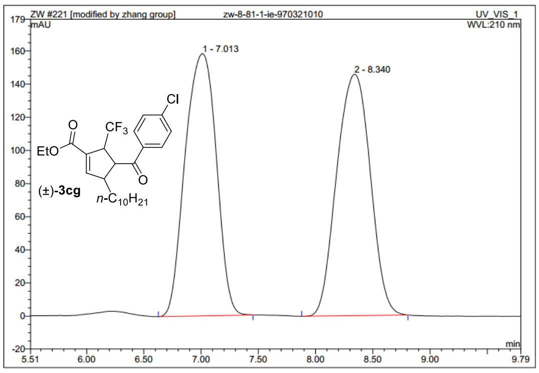

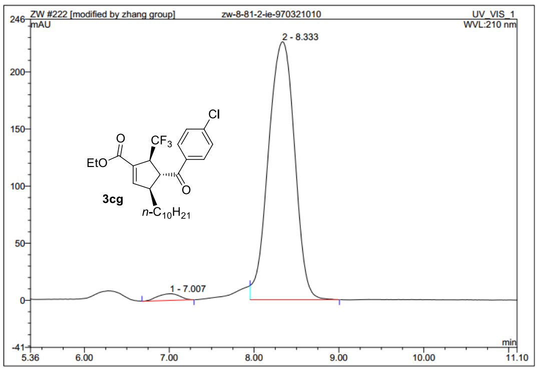

No.	Ret.Time min	Peak Name	Height mAU	Area mAU*min	Rel.Area %	Amount	Type
1	7.01	n.a.	158.132	49.998	50.21	n.a.	BMB*
2	8.34	n.a.	145.611	49.571	49.79	n.a.	BMB*
Total:			303.743	99.570	100.00	0.000	

No.	Ret.Time min	Peak Name	Height mAU	Area mAU*min	Rel.Area %	Amount	Type
1	7.01	n.a.	5.827	1.737	2.10	n.a.	BMB*
2	8.33	n.a.	225.800	80.810	97.90	n.a.	MB*
Total:			231.627	82.547	100.00	0.000	

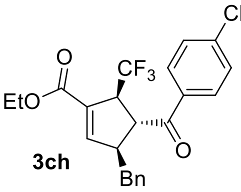

3ch; colorless oil; $[\alpha]_D^{20}$ = - 101.3 (c = 0.33, CHCl$_3$); ^{1}H NMR (500 MHz, CDCl$_3$): δ 7.44 (d, J = 9.0 Hz, 2H),7.33–7.27 (m, 5H), 7.17–7.15 (m, 2H), 6.89 (s, 1H), 4.35–4.20 (m, 3H), 3.97–3.96 (m, 1H), 3.24–3.20 (m, 1H), 3.07–3.03 (m, 1H), 2.88–2.83 (m, 1H), 1.34 (t, J = 7.00 Hz, 3H); ^{13}C NMR (125 MHz, CDCl$_3$): δ 197.36, 163.13, 149.03, 139.93, 138.38, 133.14, 131.10, 129.87, 129.06, 129.03, 128.99, 126.97, 126.23 (q, J = 278.13 Hz), 61.01, 51.98,50.69 (q, J = 29.00 Hz), 49.10, 39.89, 14.09; ^{19}F NMR (376 MHz, CDCl$_3$) δ = -67.32 ppm; Enantiomeric excess: 93%, determined by HPLC (Chiralpak IE, hexane/*i*-PrOH = 97/03; flow rate 1.0 ml/min; 25 °C; 230 nm), first peak: t_R = 12.21 min, second peak: t_R = 13.82 min; HRMS (ESI) m/z calcd. for $C_{23}H_{20}ClF_3NaO_3$ [M+Na]$^+$ = 459.0945, found = 459.0958.

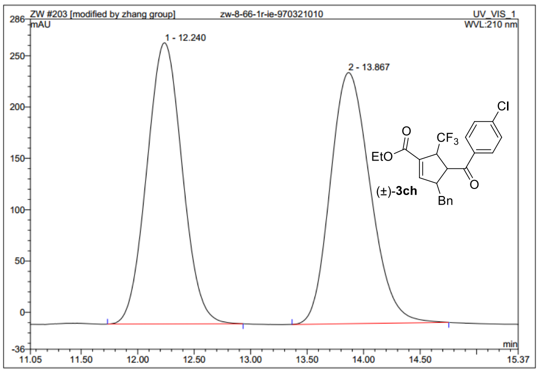

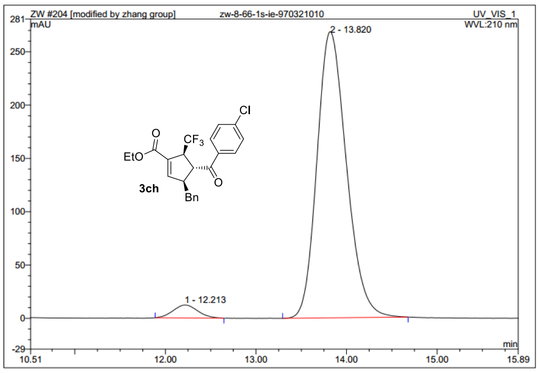

No.	Ret.Time min	Peak Name	Height mAU	Area mAU*min	Rel.Area %	Amount	Type
1	12.24	n.a.	273.775	99.522	49.83	n.a.	BMB*
2	13.87	n.a.	244.529	100.211	50.17	n.a.	BMB*
Total:			518.305	199.734	100.00	0.000	

No.	Ret.Time min	Peak Name	Height mAU	Area mAU*min	Rel.Area %	Amount	Type
1	12.21	n.a.	12.131	3.808	3.63	n.a.	BMB*
2	13.82	n.a.	268.486	101.241	96.37	n.a.	BMB*
Total:			280.617	105.049	100.00	0.000	

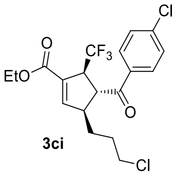

3ci

3ci; colorless oil; $[\alpha]_D^{20}$ = + 8.7 (c = 0.33, $CHCl_3$); ^1H NMR (500 MHz, $CDCl_3$): δ 7.94–7.91 (m, 2H), 7.54–7.51 (m, 2H), 6.91 (s, 1H), 4.34–4.19 (m, 3H), 3.92–3.90 (m, 1H), 3.54–3.52 (m, 2H), 2.99–2.96 (m, 1H), 1.97–1.75 (m, 4H), 1.33 (t, J = 7.50 Hz, 3H); ^{13}C NMR (125 MHz, $CDCl_3$): δ 197.09, 163.06, 148.55, 140.55, 133.50, 131.09 (d, J = 1.50 Hz), 130.04, 129.36, 126.11 (q, J = 278.00 Hz), 61.05, 50.91 (q, J = 28.88 Hz), 50.83, 49.12, 44.22, 31.37, 30.38, 14.08; ^{19}F NMR (376 MHz, $CDCl_3$) δ = -67.84 ppm; Enantiomeric excess: 91%, determined by HPLC (Chiralpak IE, hexane/i-PrOH = 97/03; flow rate 1.0 ml/min; 25 °C; 230 nm), first peak: t_R = 12.10 min, second peak: t_R = 15.73 min; HRMS (ESI) m/z calcd. for $C_{19}H_{19}Cl_2F_3NaO_3$ $[M+Na]^+$ = 445.0556, found = 445.0560.

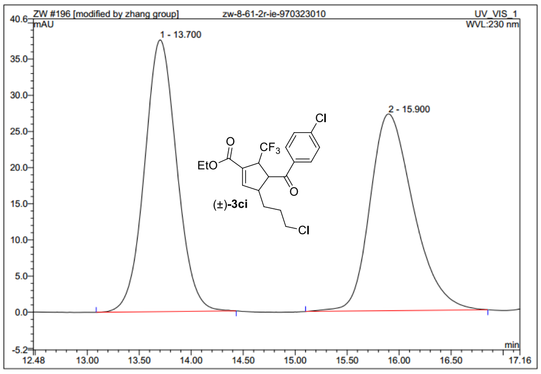

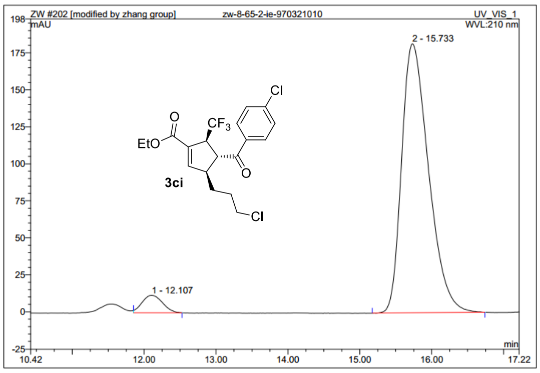

No.	Ret.Time min	Peak Name	Height mAU	Area mAU*min	Rel.Area %	Amount	Type
1	13.70	n.a.	37.576	13.509	50.63	n.a.	BMB*
2	15.90	n.a.	27.205	13.171	49.37	n.a.	BMB*
Total:			64.781	26.680	100.00	0.000	

No.	Ret.Time min	Peak Name	Height mAU	Area mAU*min	Rel.Area %	Amount	Type
1	12.11	n.a.	11.815	3.886	4.70	n.a.	MB*
2	15.73	n.a.	181.824	78.836	95.30	n.a.	BMB*
Total:			193.639	82.723	100.00	0.000	

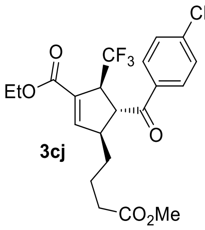

3cj

3cj; colorless oil; $[\alpha]_D^{20}$ = - 18.6 (c = 0.33, $CHCl_3$); ^{1}H NMR (400 MHz, $CDCl_3$): δ 7.91 (d, J = 8.40 Hz, 2H), 7.50 (d, J = 8.40 Hz, 2H), 6.92 (s, 1H), 4.32–4.18 (m, 3H), 3.91–3.89 (m, 1H), 3.66 (s, 3H), 2.94–2.93 (m, 1H), 2.32–2.29 (m, 2H), 1.80–1.70 (m, 4H), 1.31 (t, J = 7.20 Hz, 3H); ^{13}C NMR (100 MHz, $CDCl_3$): δ 197.25, 173.30, 163.09, 148.90, 140.42, 133.58, 130.79, 130.06, 129.30, 126.12 (q, J = 278.00 Hz), 60.96, , 51.60, 50.67, 50.90 (q, J = 29.00 Hz), 49.59, 33.50, 22.91, 14.07; ^{19}F NMR (376 MHz, $CDCl_3$) δ = -67.86 ppm; Enantiomeric excess: 91%, determined by HPLC (Chiralpak IE, hexane/i-PrOH = 95/05; flow rate 1.0 ml/min; 25 °C; 230 nm), first peak: t_R = 20.74 min, second peak: t_R = 27.18 min; HRMS (ESI) m/z calcd. for $C_{21}H_{22}ClF_3NaO_5$ $[M+Na]^+$ = 469.1000, found = 469.1008.

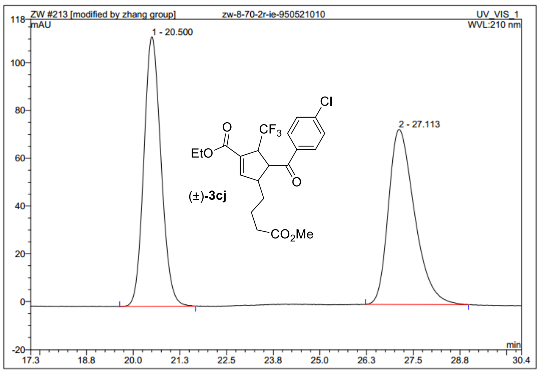

No.	Ret.Time min	Peak Name	Height mAU	Area mAU*min	Rel.Area %	Amount	Type
1	20.50	n.a.	112.885	60.247	50.34	n.a.	BMB*
2	27.11	n.a.	73.246	59.429	49.66	n.a.	BMB*
Total:			186.131	119.676	100.00	0.000	

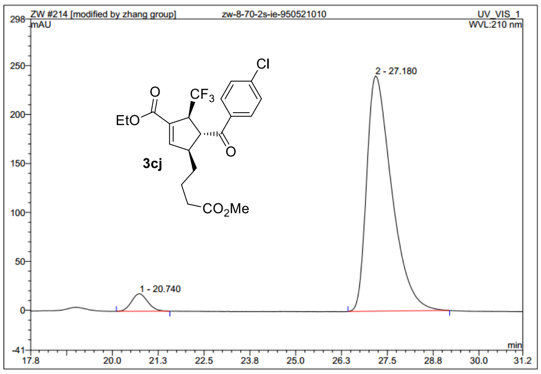

No.	Ret.Time min	Peak Name	Height mAU	Area mAU*min	Rel.Area %	Amount	Type
1	20.74	n.a.	17.852	9.163	4.59	n.a.	BMB*
2	27.18	n.a.	240.139	190.323	95.41	n.a.	BMB*
Total:			257.991	199.486	100.00	0.000	

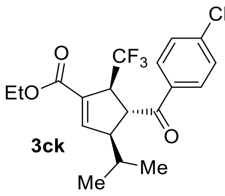

3ck

3ck; colorless oil; $[\alpha]_D^{20}$ = + 20.6 (c = 0.33, $CHCl_3$); ^{1}H NMR (400 MHz, $CDCl_3$): δ 7.91 (d, J = 8.40 Hz, 2H), 7.49 (d, J = 8.80 Hz, 2H), 6.96 (s, 1H), 4.32–4.16 (m, 2H), 4.07–3.99 (m, 2H), 2.89–2.86 (m, 1H), 1.87–1.78 (m, 1H), 1.30 (t, J = 7.60 Hz, 3H), 0.97 (d, J = 6.80 Hz, 3H), 0.93 (d, J = 6.80 Hz, 3H); ^{13}C NMR (100 MHz, $CDCl_3$): δ

199.30, 163.24, 149.09, 140.39, 134.06, 130.34, 130.08, 129.23, 126.17 (q, *J* = 278.30 Hz), 60.91, 57.44, 52.61 (q, *J* = 28.70 Hz), 48.06, 31.52, 21.69, 19.80, 14.07; ^{19}F NMR (376 MHz, $CDCl_3$) δ = -67.56 ppm; Enantiomeric excess: 92%, determined by HPLC (Chiralpak IE, hexane/*i*-PrOH = 97/03; flow rate 1.0 ml/min; 25 °C; 230 nm), first peak: t_R = 9.32 min, second peak: t_R = 12.16 min; HRMS (ESI) m/z calcd. for $C_{19}H_{20}ClF_3NaO_3$ $[M+Na]^+$ = 411.0945, found = 411.0948.

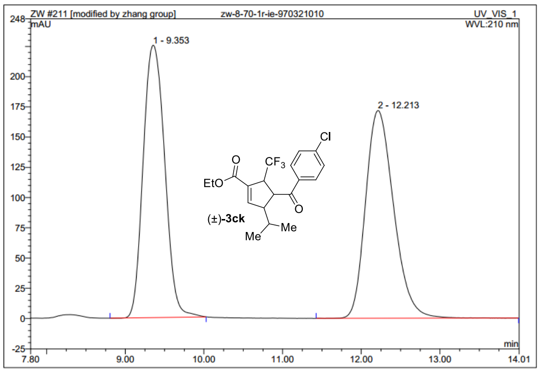

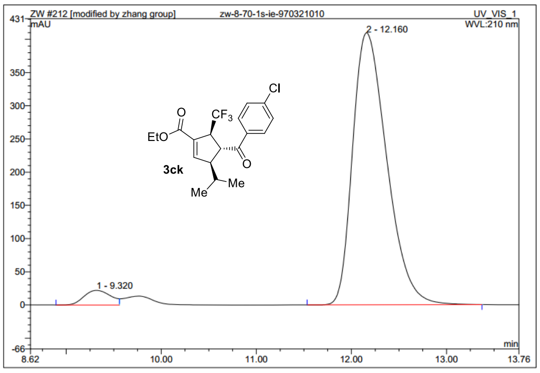

No.	Ret.Time min	Peak Name	Height mAU	Area mAU*min	Rel.Area %	Amount	Type
1	9.35	n.a.	225.252	71.548	50.16	n.a.	BMB*
2	12.21	n.a.	171.721	71.088	49.84	n.a.	BMB*
Total:			396.973	142.635	100.00	0.000	

No.	Ret.Time min	Peak Name	Height mAU	Area mAU*min	Rel.Area %	Amount	Type
1	9.32	n.a.	22.040	7.153	4.03	n.a.	BM *
2	12.16	n.a.	409.563	170.511	95.97	n.a.	BMB*
Total:			431.603	177.663	100.00	0.000	

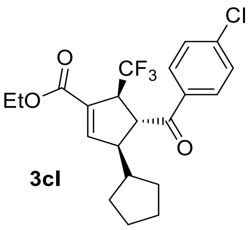

3cl

3cl; colorless oil; $[\alpha]_D^{20}$ = + 32.4 (*c* = 0.33, $CHCl_3$); ^{1}H NMR (500 MHz, $CDCl_3$): δ 7.95–7.92 (m, 2H), 7.52–7.49 (m, 2H), 6.95 (s, 1H), 4.28–4.18 (m, 2H), 4.05–3.99 (m, 2H), 2.95–2.93 (m, 1H), 2.03–1.78 (m, 3H), 1.67–1.54 (m, 4H), 1.32 (t, *J* = 7.00 Hz, 3H), 1.28–1.20 (m, 1H), 1.07–0.99 (m, 1H); ^{13}C NMR (125 MHz, $CDCl_3$): δ 199.15, 163.30, 149.95, 140.37, 134.02, 130.11, 130.03, 129.20, 126.18 (q, *J* = 278.25 Hz), 60.90, 56.05, 52.52 (q, *J* = 28.75 Hz), 49.54, 44.12, 32.24, 30.68, 25.16, 24.94, 14.08; ^{19}F NMR (376 MHz, $CDCl_3$) δ = -67.71 ppm; Enantiomeric excess: 94%, determined by HPLC (Chiralpak IE, hexane/*i*-PrOH = 97/03; flow rate 1.0 ml/min; 25 °C; 230 nm), first peak: t_R = 10.66 min, second peak: t_R = 12.14 min; HRMS (ESI) m/z calcd. for $C_{21}H_{22}ClF_3NaO_3$ $[M+Na]^+$ = 437.1102, found = 437.1112.

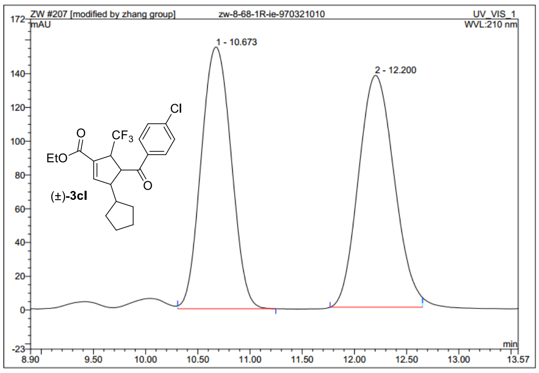

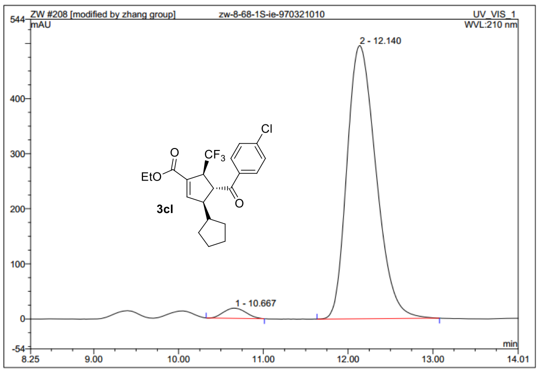

No.	Ret.Time min	Peak Name	Height mAU	Area mAU*min	Rel.Area %	Amount	Type
1	10.67	n.a.	155.049	51.788	48.81	n.a.	MB*
2	12.20	n.a.	137.340	54.304	51.19	n.a.	BM *
Total:			292.389	106.092	100.00	0.000	

No.	Ret.Time min	Peak Name	Height mAU	Area mAU*min	Rel.Area %	Amount	Type
1	10.67	n.a.	18.479	5.618	2.78	n.a.	BMB*
2	12.14	n.a.	495.720	196.750	97.22	n.a.	BMB*
Total:			514.199	202.368	100.00	0.000	

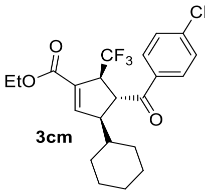

3cm; colorless oil; $[\alpha]_D^{20}$ = + 37.4 (c = 0.33, $CHCl_3$); ^1H NMR (500 MHz, $CDCl_3$): δ 7.94–7.92 (m, 2H), 7.51–7.49 (m, 2H), 7.01 (s, 1H), 4.32–4.17 (m, 2H), 4.09–4.07 (m, 1H), 4.03–3.97 (m, 1H), 2.92–2.90 (m, 1H), 1.87–1.66 (m, 5H), 1.51–1.45 (m, 1H), 1.31 (t, J = 7.00 Hz, 3H), 1.24–1.11 (m, 3H), 1.02–0.93 (m, 2H); ^{13}C NMR (125 MHz, $CDCl_3$): δ 199.24, 163.25, 149.41, 140.35, 133.96, 130.09, 129.94, 129.20, 126.17 (q, J = 278.50 Hz), 60.87, 56.34, 52.43 (q, J = 28.75 Hz), 47.66, 41.32, 32.21, 30.46, 26.04, 26.03, 25.95, 14.06; ^{19}F NMR (376 MHz, $CDCl_3$) δ = -67.59 ppm; Enantiomeric excess: 92%, determined by HPLC (Chiralpak IE, hexane/*i*-PrOH = 97/03; flow rate 1.0 ml/min; 25 °C; 230 nm), first peak: t_R = 9.35 min, second peak: t_R = 10.76 min; HRMS (ESI) m/z calcd. for $C_{22}H_{24}ClF_3NaO_3$ $[M+Na]^+$ = 451.1258, found = 451.1266.

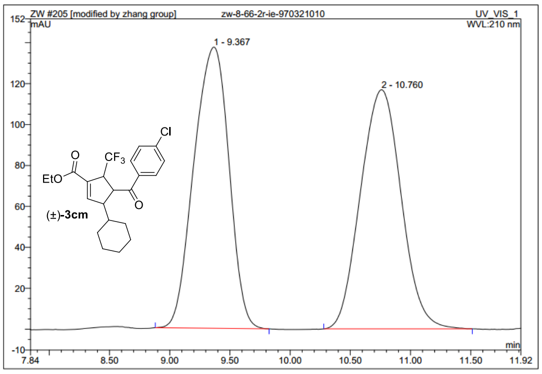

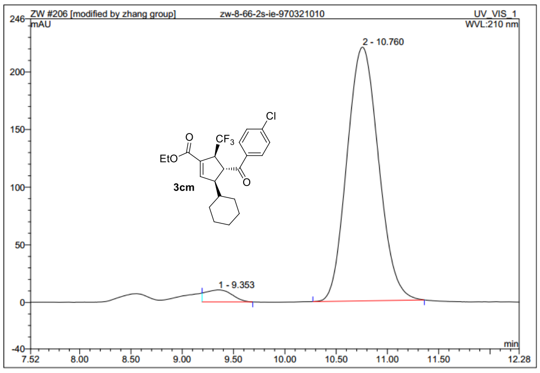

No.	Ret.Time min	Peak Name	Height mAU	Area mAU*min	Rel.Area %	Amount	Type
1	9.37	n.a.	137.422	46.391	50.32	n.a.	BMB*
2	10.76	n.a.	116.801	45.805	49.68	n.a.	BMB*
Total:			254.223	92.196	100.00	0.000	

No.	Ret.Time min	Peak Name	Height mAU	Area mAU*min	Rel.Area %	Amount	Type
1	9.35	n.a.	10.408	3.107	3.80	n.a.	MB*
2	10.76	n.a.	220.165	78.693	96.20	n.a.	BMB*
Total:			230.573	81.799	100.00	0.000	

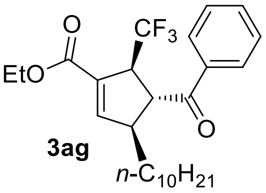

3ag; colorless oil; $[\alpha]_D^{20}$ = - 19.6 (c = 0.33, $CHCl_3$); ^{1}H NMR (400 MHz, $CDCl_3$): δ 7.97–7.95 (m, 2H), 7.64–7.61 (m, 1H), 7.53–7.50 (m, 2H), 6.92 (s, 1H), 4.33–4.16 (m, 3H), 3.94–3.93 (m, 1H), 2.91–2.88 (m, 1H), 1.74–1.61 (m, 2H), 1.33–1.21 (m, 19H), 0.88 (t, J = 6.80 Hz, 3H); ^{13}C NMR (100 MHz, $CDCl_3$): δ 198.66, 163.34, 149.67, 135.47, 133.66, 130.47, 128.86, 128.62, 126.19 (q, J = 277.90 Hz), 60.84, 51.00, 50.72 (q, J = 28.90 Hz), 50.12, 34.35, 31.83, 29.50, 29.44, 29.32, 29.30, 29.24, 27.68, 22.62, 14.05; ^{19}F NMR (376 MHz, $CDCl_3$) δ = -67.87 ppm; Enantiomeric excess: 87%, determined by HPLC (Chiralpak IE, hexane/i-PrOH = 97/03; flow rate 1.0 ml/min; 25 °C; 230 nm), first peak: t_R = 8.48 min, second peak: t_R = 11.09 min; HRMS (ESI) m/z calcd. for $C_{26}H_{35}F_3NaO_3$ $[M+Na]^+$ = 475.2431, found = 475.2435.

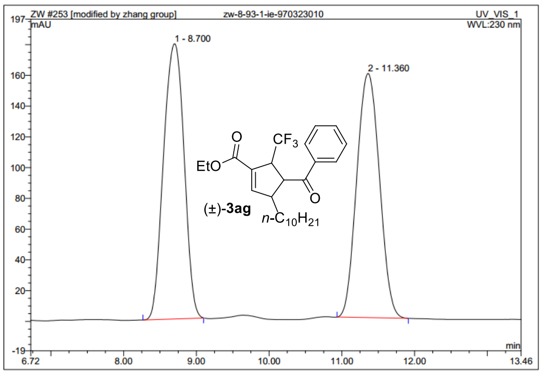

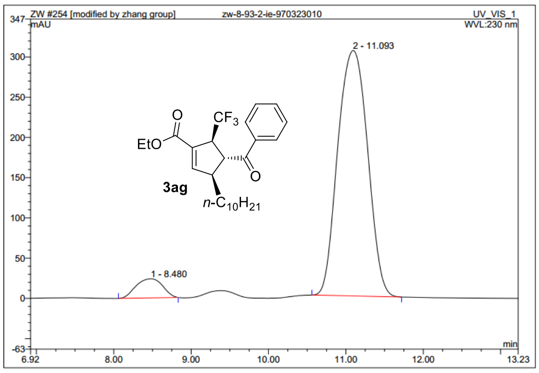

No.	Ret.Time min	Peak Name	Height mAU	Area mAU*min	Rel.Area %	Amount	Type
1	8.70	n.a.	179.029	58.703	50.58	n.a.	BMB*
2	11.36	n.a.	158.847	57.365	49.42	n.a.	BMB*
Total:			337.876	116.068	100.00	0.000	

No.	Ret.Time min	Peak Name	Height mAU	Area mAU*min	Rel.Area %	Amount	Type
1	8.48	n.a.	23.773	9.485	6.50	n.a.	BMB*
2	11.09	n.a.	305.037	136.542	93.50	n.a.	BMB*
Total:			328.810	146.028	100.00	0.000	

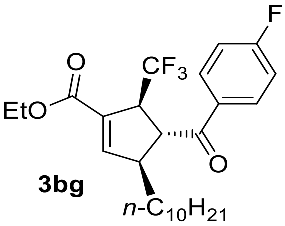

3bg

3bg; colorless oil; $[\alpha]_D^{20}$ = -7.4 (c = 0.33, $CHCl_3$); ^{1}H NMR (400 MHz, $CDCl_3$): δ 8.02–7.98 (m, 2H), 7.21–7.17 (m, 2H), 6.92 (s, 1H), 4.33–4.16 (m, 3H), 3.89–3.88 (m, 1H), 2.93–2.87 (m, 1H), 1.72–1.65 (m, 2H), 1.33–1.22 (m, 19H), 0.88 (t, J = 6.80 Hz, 3H); ^{13}C NMR (100 MHz, $CDCl_3$): δ 197.18, 166.13 (d, J = 254.60 Hz), 149.62, 131.84 (d, J = 2.90 Hz), 131.31 (d, J = 9.40 Hz), 130.41, 126.23 (q, J = 278.10 Hz), 116.07 (d, J = 21.80 Hz), 60.87, 50.94 (q, J = 28.80 Hz), 50.91, 50.16, 34.35, 31.83, 29.50, 29.45, 29.33, 29.32, 29.24, 27.72, 22.62, 14.06, 14.04; ^{19}F NMR (376 MHz, $CDCl_3$) δ = -67.89, -103.91 ppm; Enantiomeric excess: 95%, determined by HPLC (Chiralpak IE, hexane/i-PrOH = 95/05; flow rate 0.5 ml/min; 25 °C; 230 nm), first peak: t_R = 11.87 min, second peak: t_R = 13.81 min; HRMS (ESI) m/z calcd. for $C_{26}H_{34}F_4NaO_3$ $[M+Na]^+$ = 493.2336, found = 493.2338.

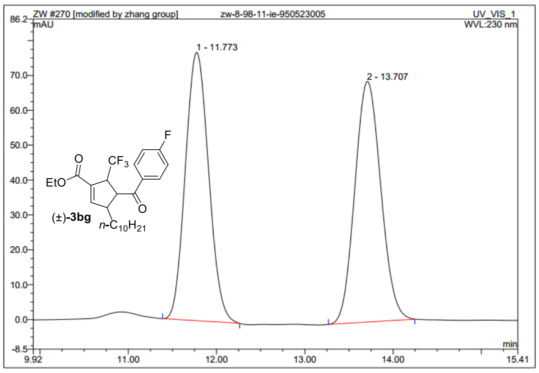

No.	Ret.Time min	Peak Name	Height mAU	Area mAU*min	Rel.Area %	Amount	Type
1	11.77	n.a.	76.928	23.535	50.00	n.a.	BMB*
2	13.71	n.a.	69.018	23.536	50.00	n.a.	BMB*
Total:			145.946	47.071	100.00	0.000	

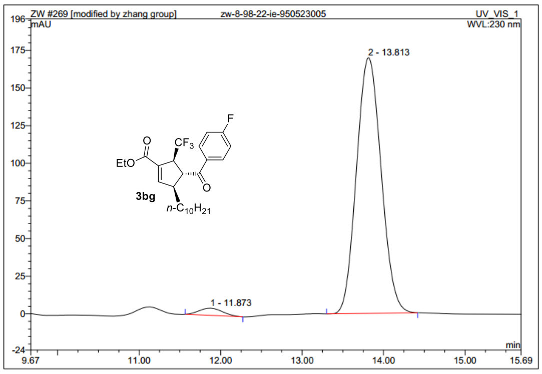

No.	Ret.Time min	Peak Name	Height mAU	Area mAU*min	Rel.Area %	Amount	Type
1	11.87	n.a.	4.758	1.571	2.57	n.a.	BMB*
2	13.81	n.a.	169.820	59.497	97.43	n.a.	BMB*
Total:			174.578	61.068	100.00	0.000	

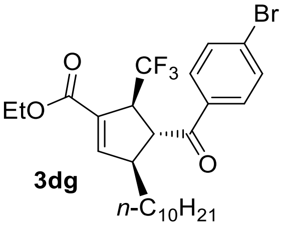

3dg

3dg; colorless oil; $[\alpha]_D^{20}$ = -30.7 (c = 0.33, $CHCl_3$); ^{1}H NMR (400 MHz, $CDCl_3$): δ 7.82 (d, J = 8.40 Hz, 2H), 7.66 (d, J = 8.40 Hz, 2H), 6.92 (s, 1H), 4.33–4.16 (m, 3H),

3.87–3.85 (m, 1H), 2.90–2.86 (m, 1H), 1.69–1.64 (m, 2H), 1.33–1.22 (m, 19H), 0.88 (t, J = 6.80 Hz, 3H); ^{13}C NMR (100 MHz, $CDCl_3$): δ 197.70, 163.26, 149.58, 134.13, 132.25, 130.37, 130.10, 129.09, 126.19 (q, J = 278.00 Hz), 60.93, 50.94, 50.78 (q, J = 32.40 Hz), 50.07, 34.38, 31.86, 29.53, 29.48, 29.34, 29.28, 27.73, 22.66, 14.10; ^{19}F NMR (376 MHz, $CDCl_3$) δ = -67.86 ppm; Enantiomeric excess: 96%, determined by HPLC (Chiralpak IE, hexane/*i*-PrOH = 97/03; flow rate 1.0 ml/min; 25 °C; 230 nm), first peak: t_R = 7.53 min, second peak: t_R = 8.68 min; HRMS (ESI) m/z calcd. for $C_{26}H_{34}BrF_3NaO_3$ [M+Na]$^+$ = 553.1536, found = 553.1541.

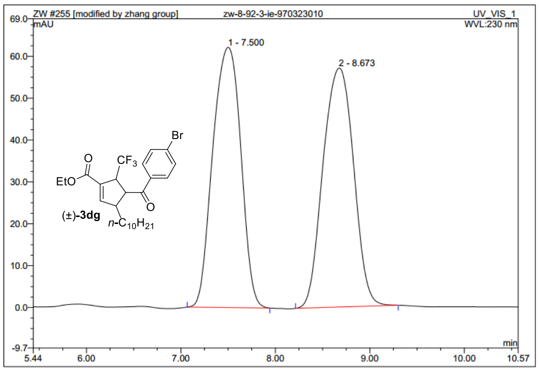

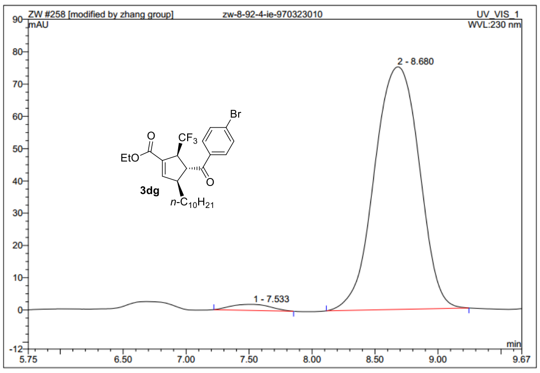

No.	Ret.Time min	Peak Name	Height mAU	Area mAU*min	Rel.Area %	Amount	Type
1	7.50	n.a.	62.158	21.379	49.93	n.a.	BMB*
2	8.67	n.a.	57.082	21.442	50.07	n.a.	BMB*
Total:			119.240	42.821	100.00	0.000	

No.	Ret.Time min	Peak Name	Height mAU	Area mAU*min	Rel.Area %	Amount	Type
1	7.53	n.a.	1.898	0.678	2.14	n.a.	BMB*
2	8.68	n.a.	75.193	31.039	97.86	n.a.	BMB*
Total:			77.091	31.717	100.00	0.000	

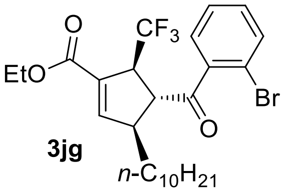

3jg

3jg; colorless oil; $[\alpha]_D^{20}$ = -11.7 (c = 0.33, $CHCl_3$); ^{1}H NMR (500 MHz, $CDCl_3$): δ 7.67–7.66 (m, 1H), 7.45–7.41 (m, 1H), 7.38–7.34 (m, 2H), 6.86 (s, 1H), 4.44–4.20 (m, 3H), 3.86–3.85 (m, 1H), 3.02–2.98 (m, 1H), 1.55–1.40 (m, 2H), 1.35–1.12 (m, 19H), 0.90 (t, J = 7.00 Hz, 3H); ^{13}C NMR (125 MHz, $CDCl_3$): δ 200.67, 163.28, 149.17, 140.

10, 133.77, 131.92, 130.21, 128.65, 127.40, 126.15 (q, J = 277.88 Hz), 119.21, 60.89, 54.87, 49.13 (q, J = 29.00 Hz), 48.52, 34.36, 31.85, 29.50, 29.38, 29.27, 29.25, 29.05, 27.39, 22.64, 14.08; ^{19}F NMR (376 MHz, $CDCl_3$) δ = -68.29 ppm; Enantiomeric excess: 96%, determined by HPLC (Chiralpak IE, hexane/*i*-PrOH = 97/03; flow rate 1.0 ml/min; 25 °C; 230 nm), first peak: t_R = 7.99 min, second peak: t_R = 9.54 min;

HRMS (ESI) m/z calcd. for $C_{26}H_{34}BrF_3NaO_3$ $[M+Na]^+$ = 553.1536, found = 553.1550.

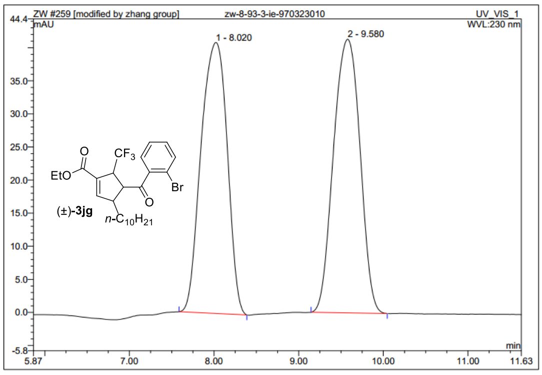

No.	Ret.Time min	Peak Name	Height mAU	Area mAU*min	Rel.Area %	Amount	Type
1	8.02	n.a.	40.952	14.690	49.44	n.a.	BMB*
2	9.58	n.a.	41.348	15.021	50.56	n.a.	BMB*
Total:			82.300	29.711	100.00	0.000	

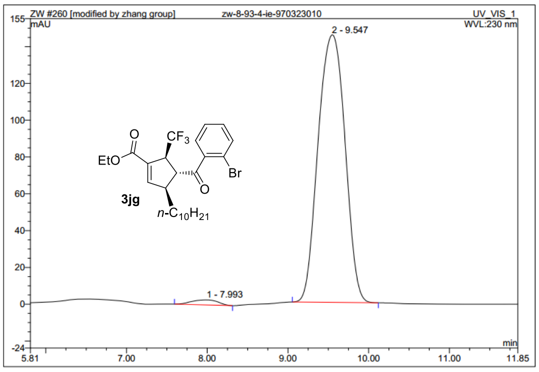

No.	Ret.Time min	Peak Name	Height mAU	Area mAU*min	Rel.Area %	Amount	Type
1	7.99	n.a.	2.771	1.039	1.81	n.a.	BMB*
2	9.55	n.a.	145.440	56.275	98.19	n.a.	BMB*
Total:			148.211	57.314	100.00	0.000	

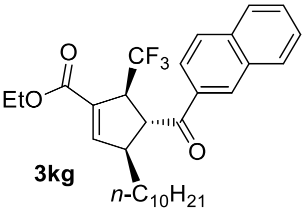

3kg; colorless oil; $[\alpha]_D^{20}$ = -52.5 (c = 0.33, $CHCl_3$); ^1H NMR (500 MHz, $CDCl_3$): δ 8.50 (s, 1H), 8.06–7.92 (m, 4H), 7.68–7.60 (m, 2H), 6.97 (s, 1H), 4.38–4.21 (m, 3H), 4.13–4.12 (m, 1H), 3.00–2.97 (m, 1H), 1.85–1.69 (m, 2H), 1.36–1.19 (m, 19H), 0.89 (t, J = 7.00 Hz, 3H); ^{13}C NMR (125 MHz, $CDCl_3$): δ 198.59, 163.39, 149.79, 135.80, 132.74, 132.45, 130.49, 130.34, 129.63, 128.89, 128.86, 127.79, 127.05, 126.37 (q, J = 278.00 Hz), 124.23, 60.87, 51.05, 50.84 (q, J = 28.63 Hz), 50.28, 34.39, 31.82, 29.48, 29.43, 29.34, 29.30, 29.22, 27.72, 22.62, 14.09, 14.07; ^{19}F NMR (376 MHz, $CDCl_3$) δ = -67.74 ppm; Enantiomeric excess: 94%, determined by HPLC (Chiralpak IE, hexane/i-PrOH = 95/05; flow rate 0.8 ml/min; 25 °C; 230 nm), first peak: t_R = 10.81 min, second peak: t_R = 13.76 min; HRMS (ESI) m/z calcd. for $C_{30}H_{37}F_3NaO_3$ $[M+Na]^+$ = 525.2587, found = 525.2591.

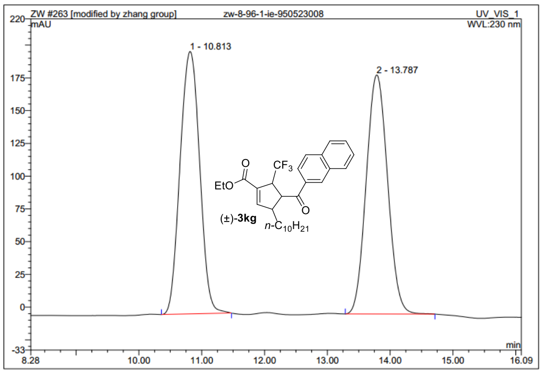

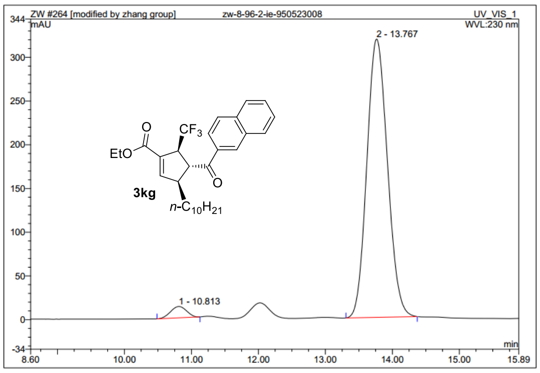

No.	Ret.Time min	Peak Name	Height mAU	Area mAU*min	Rel.Area %	Amount	Type
1	10.81	n.a.	200.325	72.365	50.16	n.a.	BMB*
2	13.79	n.a.	182.509	71.902	49.84	n.a.	BMB*
Total:			382.834	144.267	100.00	0.000	

No.	Ret.Time min	Peak Name	Height mAU	Area mAU*min	Rel.Area %	Amount	Type
1	10.81	n.a.	13.009	3.595	3.07	n.a.	BMB*
2	13.77	n.a.	318.262	113.313	96.93	n.a.	BMB*
Total:			331.272	116.908	100.00	0.000	

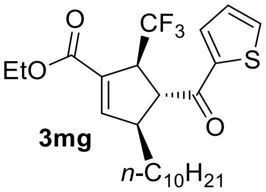

3mg

3mg; colorless oil; $[\alpha]_D^{20}$ = + 10.7 (*c* = 0.33, $CHCl_3$); ^{1}H NMR (500 MHz, $CDCl_3$): δ 7.80 (d, *J* = 3.50 Hz, 1H), 7.76 (d, *J* = 5.00 Hz, 1H), 7.22–7.20 (m, 1H), 6.96 (s, 1H), 4.33–4.18 (m, 3H), 3.75–3.73 (m, 1H), 3.06–3.03 (m, 1H), 1.77–1.63 (m, 2H), 1.33–1.25 (m, 19H), 0.89 (t, *J* = 6.50 Hz, 3H); ^{13}C NMR (125 MHz, $CDCl_3$): δ 191.66, 163.28, 149.98, 142.75, 135.20, 132.58, 130.21, 128.45,126.15 (q, *J* = 278.00 Hz), 60.86, 52.34, 51.48 (q, *J* = 28.88 Hz), 50.42, 34.38, 31.84, 29.46, 29.41, 29.35, 29.25, 27.71, 22.63, 14.07; ^{19}F NMR (376 MHz, $CDCl_3$) δ = -68.00 ppm; Enantiomeric excess: 91%, determined by HPLC (Chiralpak IE, hexane/*i*-PrOH = 95/05; flow rate 0.8 ml/min; 25 °C; 230 nm), first peak: t_R = 8.61 min, second peak: t_R = 10.73 min; HRMS (ESI) m/z calcd. for $C_{24}H_{33}F_3NaO_3S$ $[M+Na]^+$ = 481.1995, found = 481.1999.

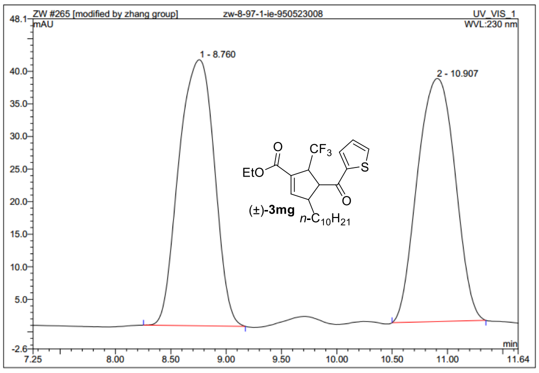

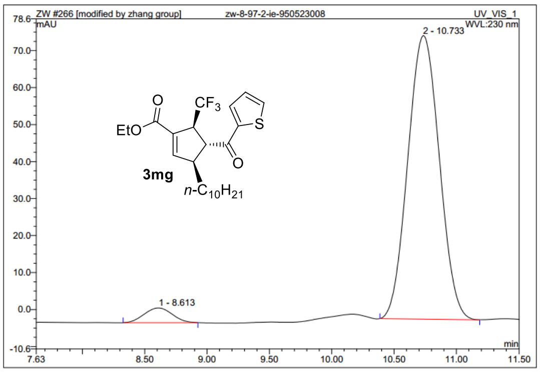

No.	Ret.Time min	Peak Name	Height mAU	Area mAU*min	Rel.Area %	Amount	Type
1	8.76	n.a.	40.837	15.210	50.78	n.a.	BMB*
2	10.91	n.a.	37.314	14.741	49.22	n.a.	BMB*
Total:			78.151	29.951	100.00	0.000	

No.	Ret.Time min	Peak Name	Height mAU	Area mAU*min	Rel.Area %	Amount	Type
1	8.61	n.a.	3.972	1.030	4.39	n.a.	BMB*
2	10.73	n.a.	76.624	22.443	95.61	n.a.	BMB*
Total:			80.597	23.473	100.00	0.000	

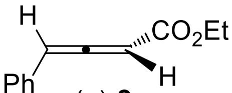

(+)-**2a**

(*S*)-**2a**;[3] colorless oil; $[\alpha]_D^{20}$ = + 239.8.3 (*c* = 0.33, $CHCl_3$); ^{1}H NMR (400 MHz, $CDCl_3$): δ 7.28–7.19 (m, 5H), 6.55 (d, *J* = 6.40 Hz, 1H), 5.94 (d, *J* = 6.40 Hz, 1H), 4.15 (q, *J* = 7.20 Hz, 2H), 1.21 (t, *J* = 7.20 Hz, 3H); ^{13}C NMR (100 MHz, $CDCl_3$): δ 214.58, 165.07, 131.11, 128.81, 128.06, 127.46, 98.63, 91.91, 61.10, 14.20; Enantiomeric excess: 76%, determined by HPLC (Chiralpak OD-H, hexane/*i*-PrOH = 99/01; flow rate 1.0 ml/min; 25 °C; 230 nm), first peak: t_R = 9.22 min, second peak: t_R = 9.63 min.

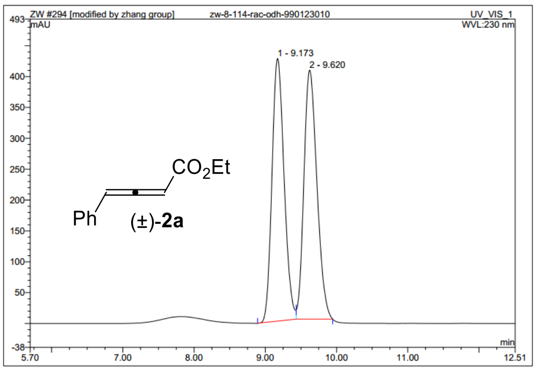

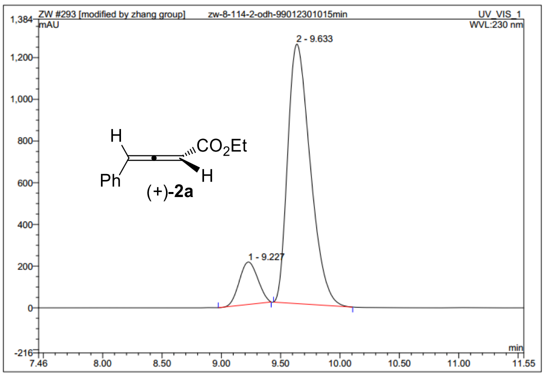

No.	Ret.Time min	Peak Name	Height mAU	Area mAU*min	Rel.Area %	Amount	Type
1	9.17	n.a.	425.332	83.754	49.57	n.a.	BM *
2	9.62	n.a.	403.358	85.212	50.43	n.a.	MB*
Total:			828.690	168.966	100.00	0.000	

No.	Ret.Time min	Peak Name	Height mAU	Area mAU*min	Rel.Area %	Amount	Type
1	9.23	n.a.	203.829	36.793	11.82	n.a.	BMB*
2	9.63	n.a.	1243.632	274.504	88.18	n.a.	BMB*
Total:			1447.461	311.298	100.00	0.000	

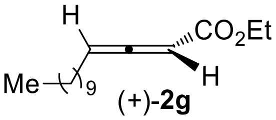

(+)-**2g**

(*S*)-**2g**;[4] colorless oil; $[\alpha]_D^{20}$ = + 306.3 (*c* = 1.0, $CHCl_3$); ^{1}H NMR (500 MHz, $CDCl_3$): δ 5.63–5.66 (m, 2H), 4.23–4.17 (m, 2H), 2.16–2.11 (m, 2H), 1.49–1.44 (m, 2H),

1.37–1.27 (m, 2H), 0.89 (t, J = 7.00 Hz, 3H); ^{13}C NMR (125 MHz, $CDCl_3$): δ 212.26, 166.27, 95.34, 88.17, 60.66, 31.86, 29.56, 29.54, 29.32, 29.28, 28.89, 28.68, 27.46, 22.64, 14.20, 14.06; ^{19}F NMR (376 MHz, $CDCl_3$) δ = -67.50 ppm; Enantiomeric excess: 83%, determined by HPLC (Chiralpak OD-H, hexane/*i*-PrOH = 99/01; flow rate 0.5 ml/min; 25 °C; 210 nm), first peak: t_R = 9.68 min, second peak: t_R = 10.23 min.

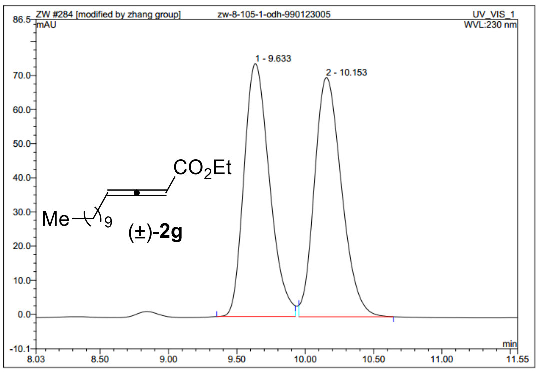

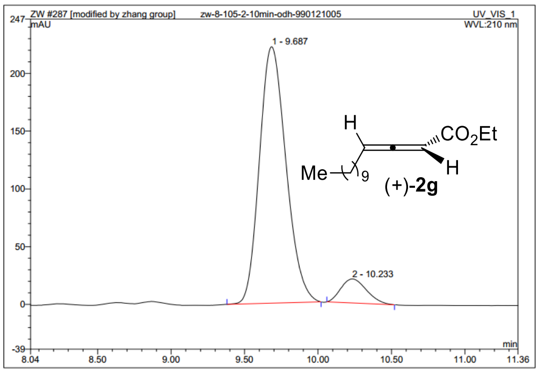

No.	Ret.Time min	Peak Name	Height mAU	Area mAU*min	Rel.Area %	Amount	Type
1	9.63	n.a.	74.159	16.028	49.83	n.a.	BM *
2	10.15	n.a.	70.152	16.135	50.17	n.a.	MB*
Total:			144.311	32.163	100.00	0.000	

No.	Ret.Time min	Peak Name	Height mAU	Area mAU*min	Rel.Area %	Amount	Type
1	9.69	n.a.	221.937	46.572	91.69	n.a.	BMB*
2	10.23	n.a.	20.885	4.221	8.31	n.a.	BMB*
Total:			242.822	50.793	100.00	0.000	

9. References

[1] a) X. Su, W. Zhou, Y. Li, J. Zhang, *Angew. Chem. Int. Ed.* **2015**, *54*, 6874; *Angew. Chem.* **2015**, *127*, 6978; b) W. Zhou, X. Su. M. Tao, C. Zhu, Q. Zhao, J. Zhang, *Angew. Chem. Int. Ed.* **2015**, *54*, 14853; *Angew. Chem.* **2015**, *127*, 15066; c) W. Zhou, P. Chen, M. Tao, X. Su, Q. Zhao, J. Zhang, *Chem. Commun.* **2016**, *52*, 7612; d) P. Chen, X. Su, W. Zhou, Y. Xiao, J. Zhang, *Tetrahedron* **2016**, *72*, 2700.

[2] T. Yamazaki, T. Kawasaki-Takasuka, A. Furuta, S. Sakamoto, *Tetrahedron*, **2009**, *65*, 5945.

[3] T. Inokuma, M. Furukawa, T. Uno, Y. Suzuki, K. Yoshida, Y. Yano, K. Matsuzaki, Y. Takemoto, *Chem. Eur. J.* **2011**, *17*, 10470.

[4] C.-Y. Li, X.-L. Sun, Q. Jing, Y. Tang, *Chem. Commun.* **2006**, 2980.

10. ^{1}H, ^{13}C, ^{31}P and ^{19}F NMR Spectra

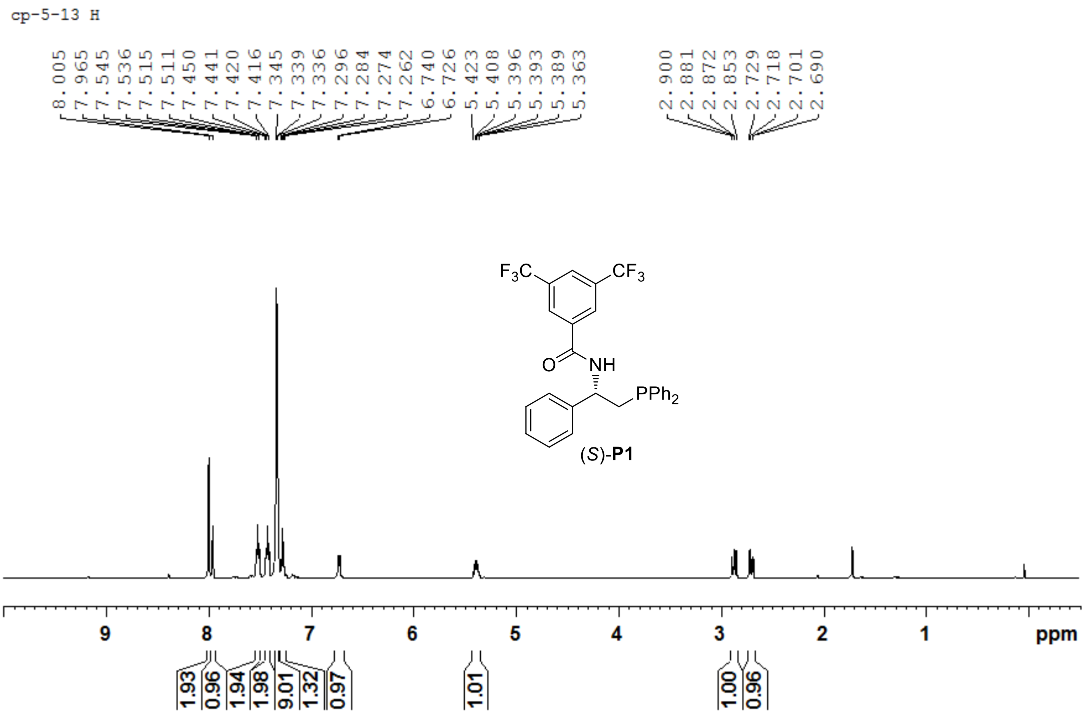

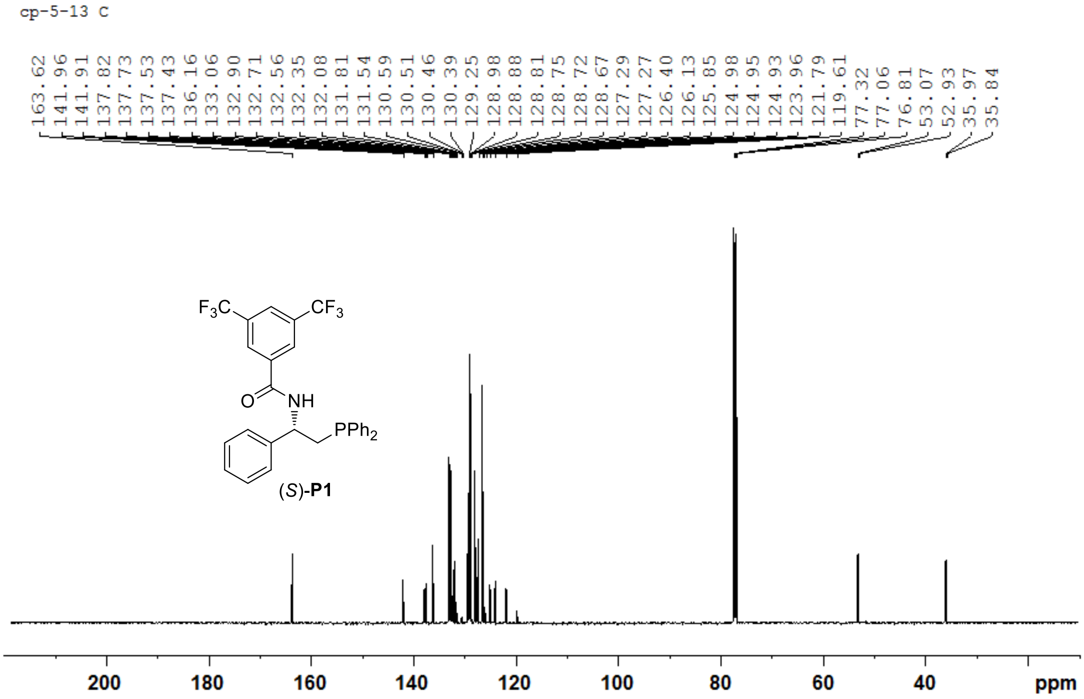

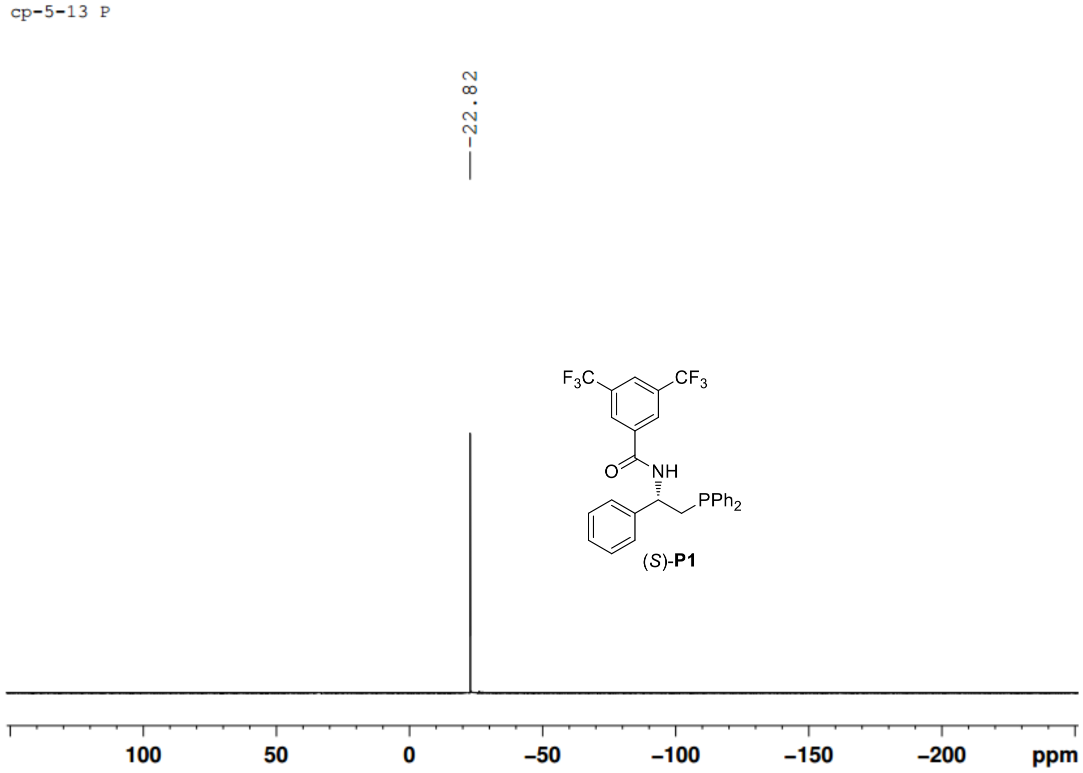
cp-5-13 P
-22.82
F3C
CF3
O
NH
PPh2
(S)-P1
100
50
0
-50
-100
-150
-200
ppm

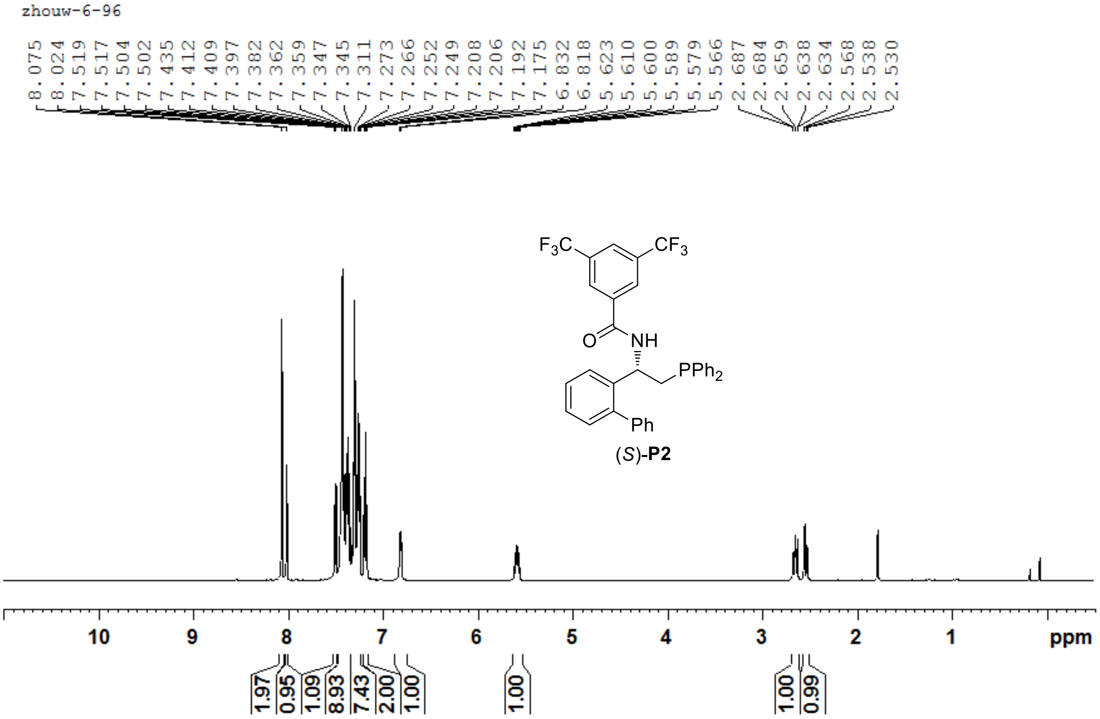
zhouw-6-96
8.075
8.024
7.519
7.517
7.504
7.502
7.435
7.412
7.409
7.397
7.382
7.362
7.359
7.347
7.345
7.311
7.273
7.266
7.252
7.249
7.208
7.206
7.192
7.175
6.832
6.818
5.623
5.610
5.600
5.589
5.579
5.566
2.687
2.684
2.659
2.638
2.634
2.568
2.538
2.530
F3C
CF3
O
NH
PPh2
Ph
(S)-P2
10
9
8
7
6
5
4
3
2
1
ppm
1.97
0.95
1.09
8.93
7.43
2.00
1.00
1.00
1.00
0.99

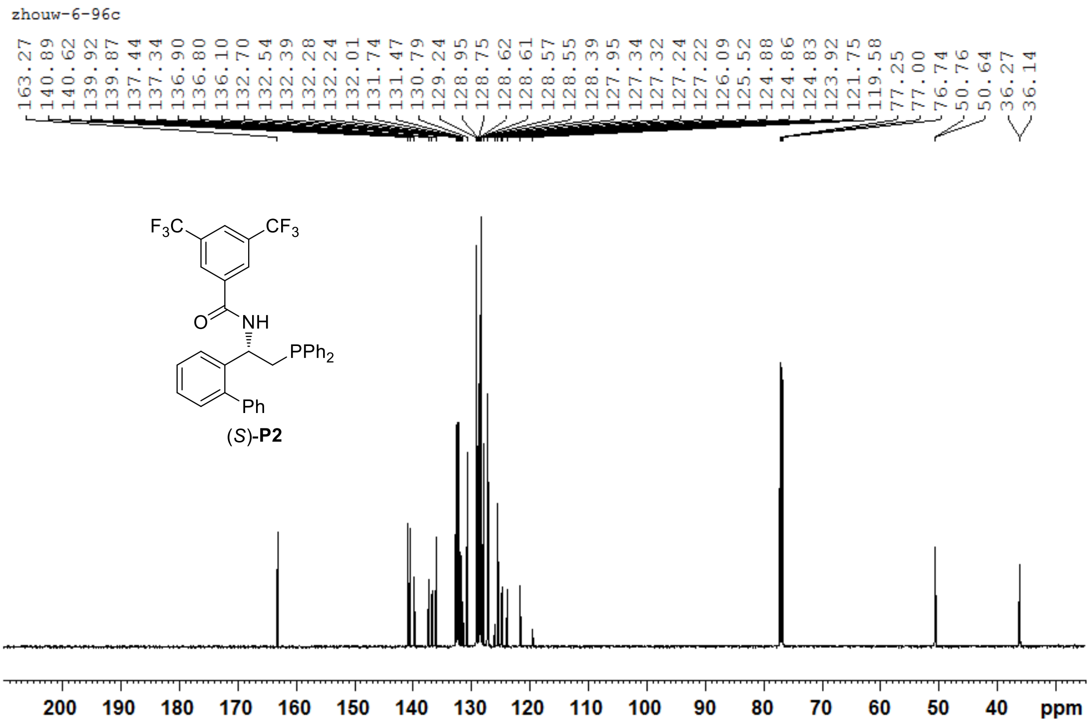
zhouw-6-96c
163.27
140.89
140.62
139.92
139.87
137.44
137.34
136.90
136.80
136.10
132.70
132.54
132.39
132.28
132.24
132.01
131.74
131.47
130.79
129.24
128.95
128.75
128.62
128.61
128.57
128.55
128.39
127.95
127.34
127.32
127.24
127.22
126.09
125.52
124.88
124.86
124.83
123.92
121.75
119.58
77.25
77.00
76.74
50.76
50.64
36.27
36.14
F_3C
CF_3
O
NH
PPh_2
Ph
(*S*)-**P2**
200 190 180 170 160 150 140 130 120 110 100 90 80 70 60 50 40 ppm

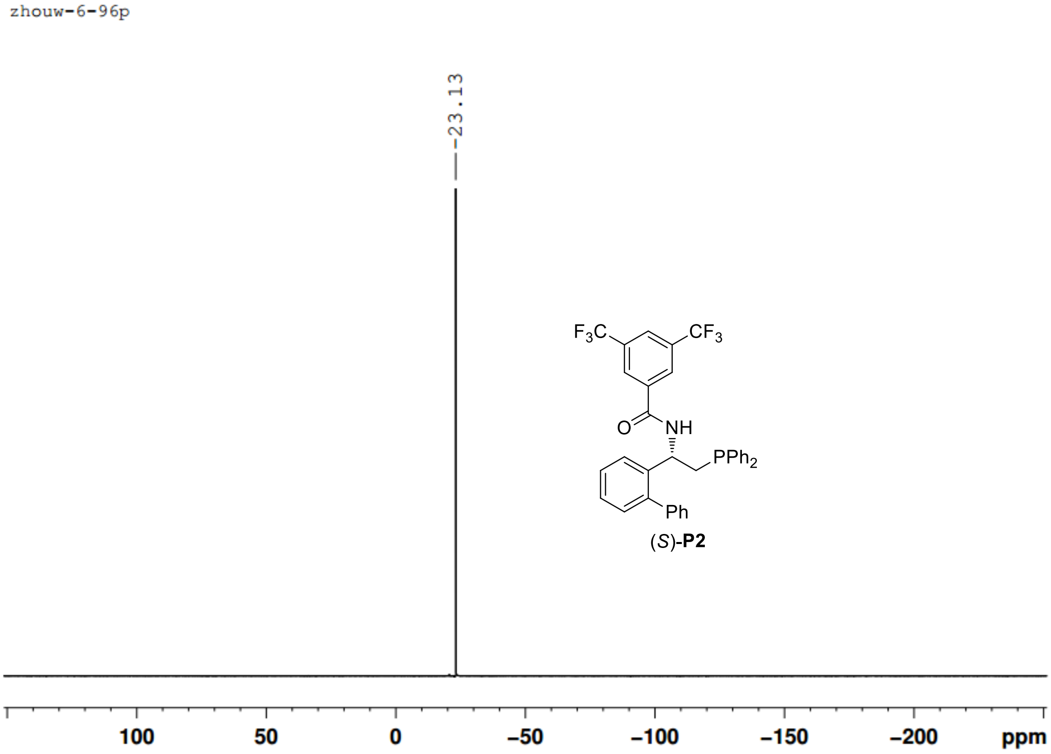
zhouw-6-96p
-23.13
F_3C
CF_3
O
NH
PPh_2
Ph
(*S*)-**P2**
100 50 0 −50 −100 −150 −200 ppm

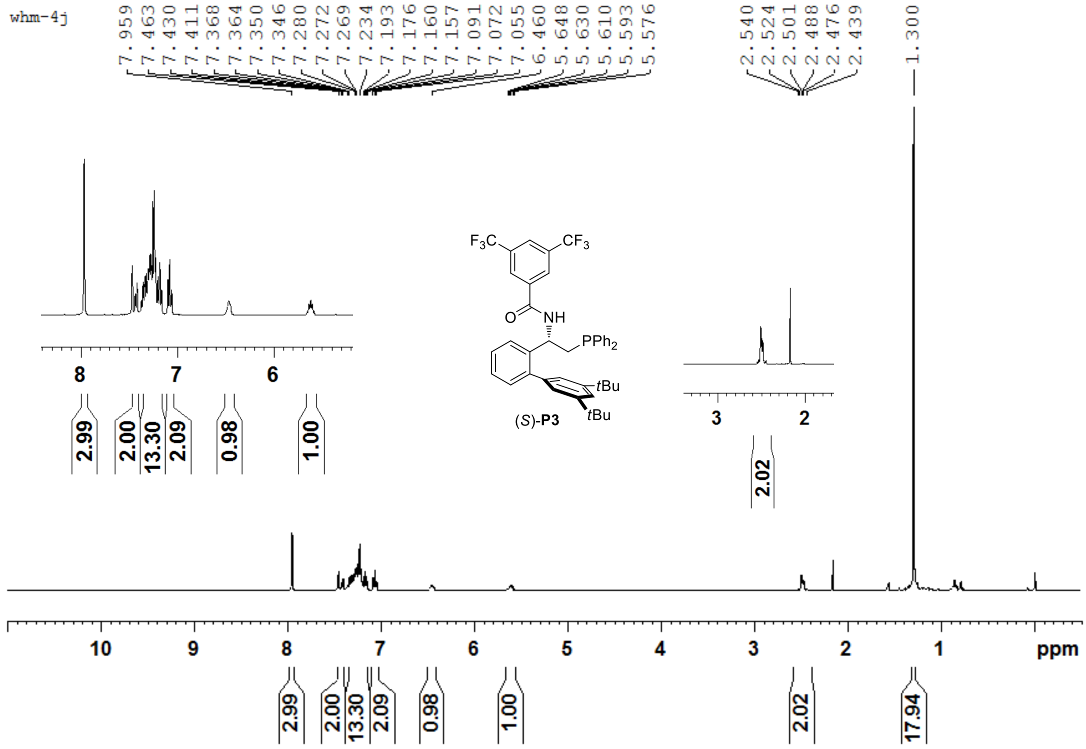

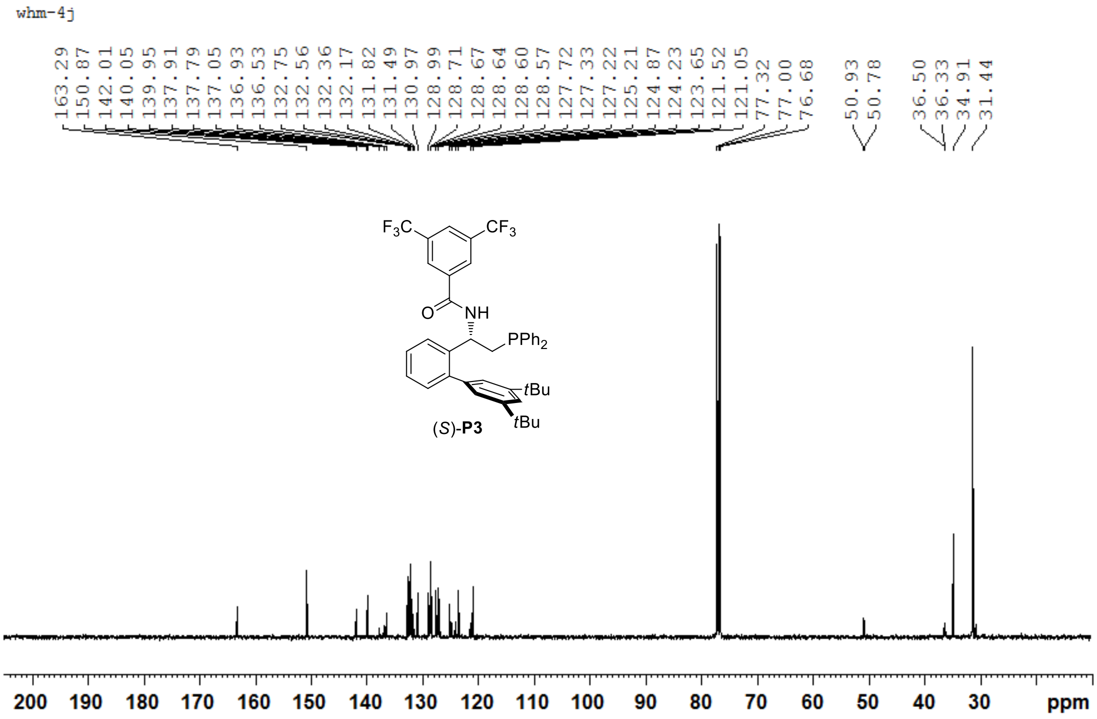

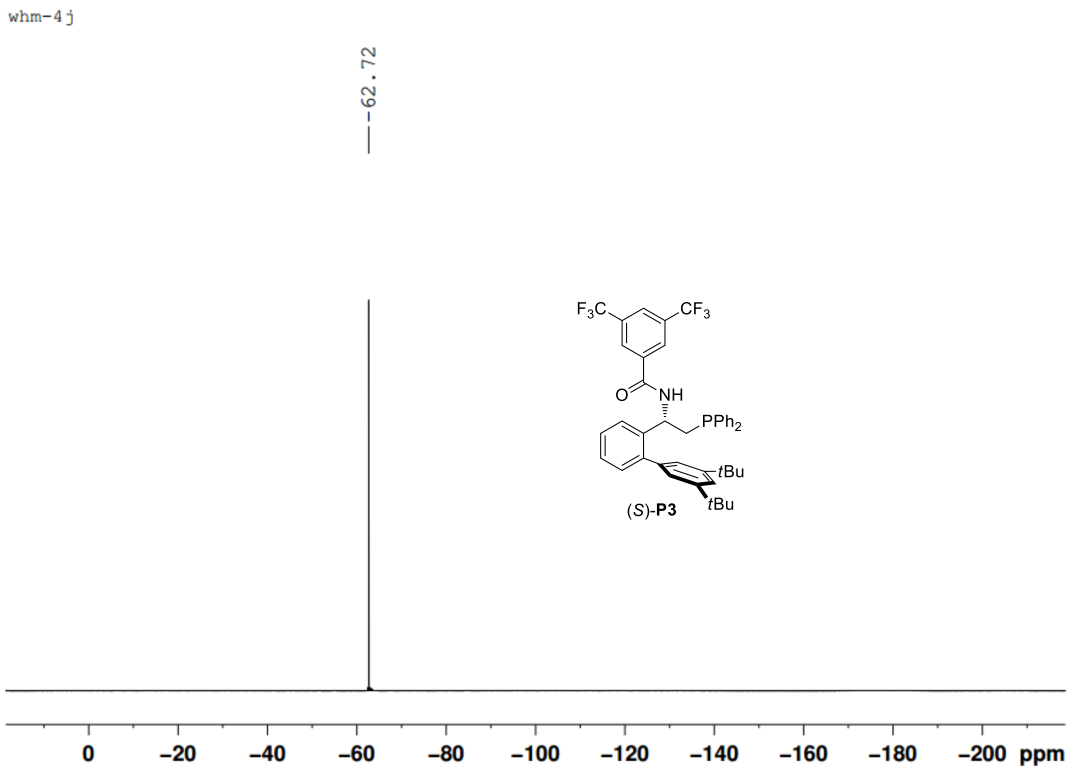
whm-4j
62.72
F3C
CF3
O
NH
PPh2
tBu
tBu
(S)-P3
0
–20
–40
–60
–80
–100
–120
–140
–160
–180
–200
ppm

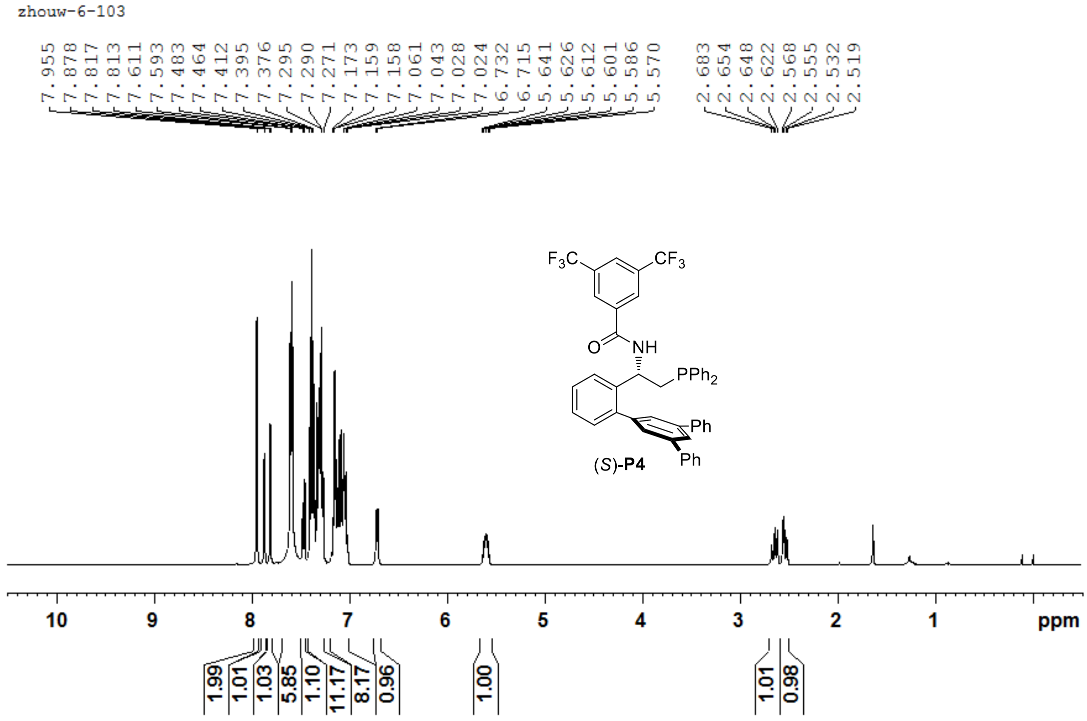
zhouw-6-103
7.955
7.878
7.817
7.813
7.611
7.593
7.483
7.464
7.412
7.395
7.376
7.295
7.290
7.271
7.173
7.159
7.158
7.061
7.043
7.028
7.024
6.732
6.715
5.641
5.626
5.612
5.601
5.586
5.570
2.683
2.654
2.648
2.622
2.568
2.555
2.532
2.519
F3C
CF3
O
NH
PPh2
Ph
Ph
(S)-P4
10
9
8
7
6
5
4
3
2
1
ppm
1.99
1.01
1.03
5.85
1.10
11.17
8.17
0.96
1.00
1.01
0.98

zhouw-6-103c

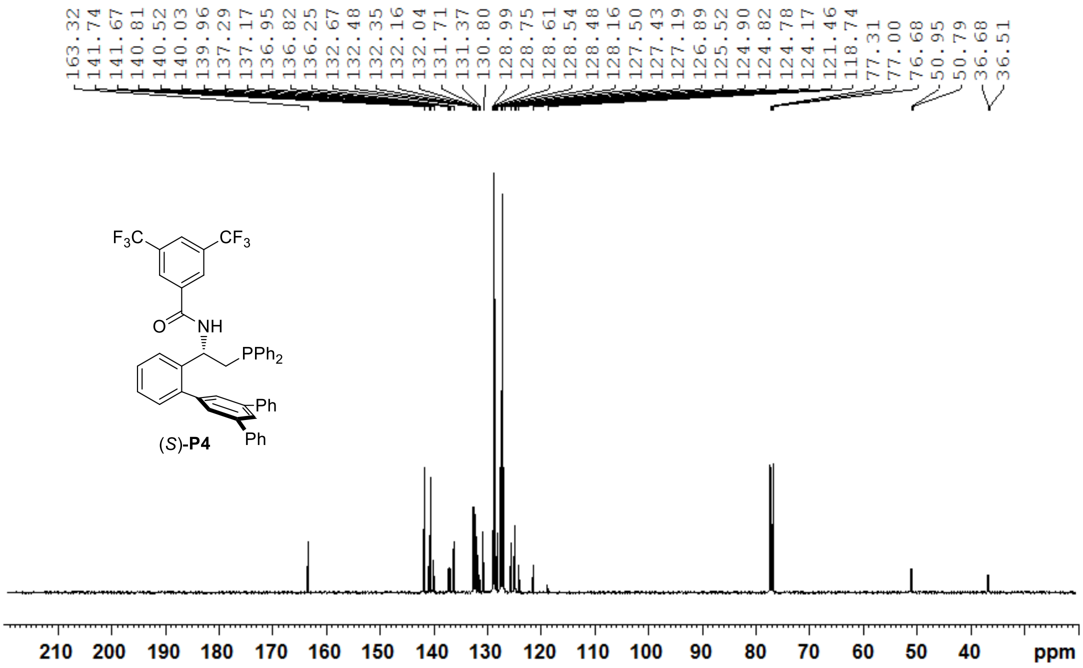

zhouw-6-103p

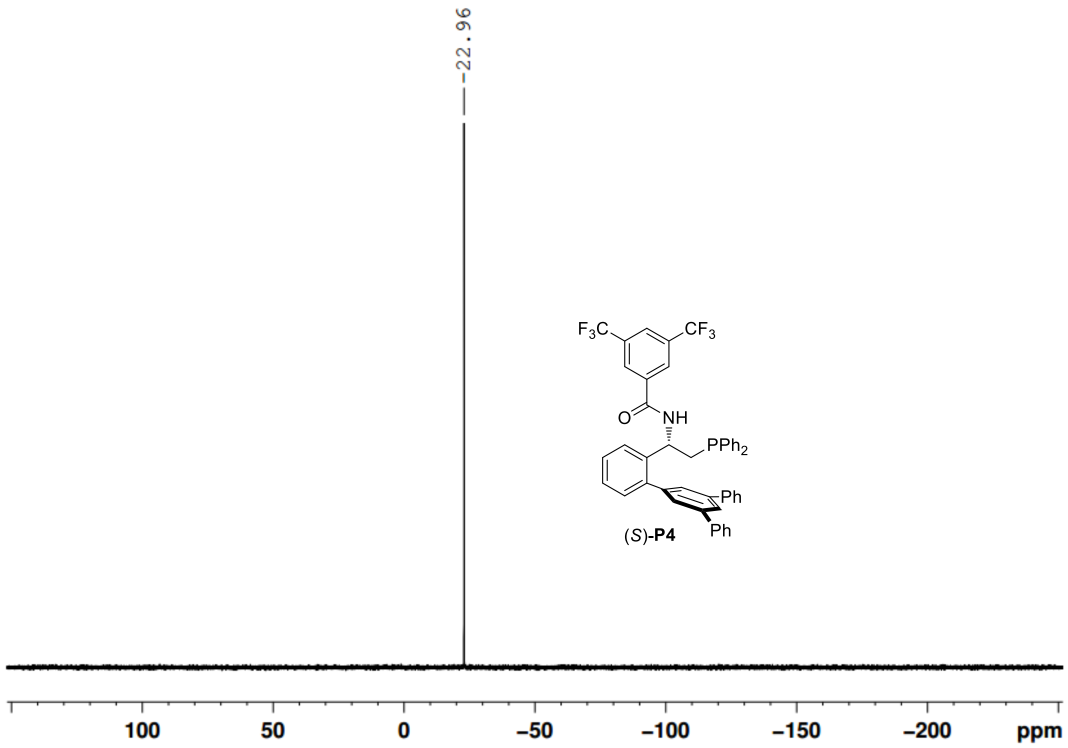

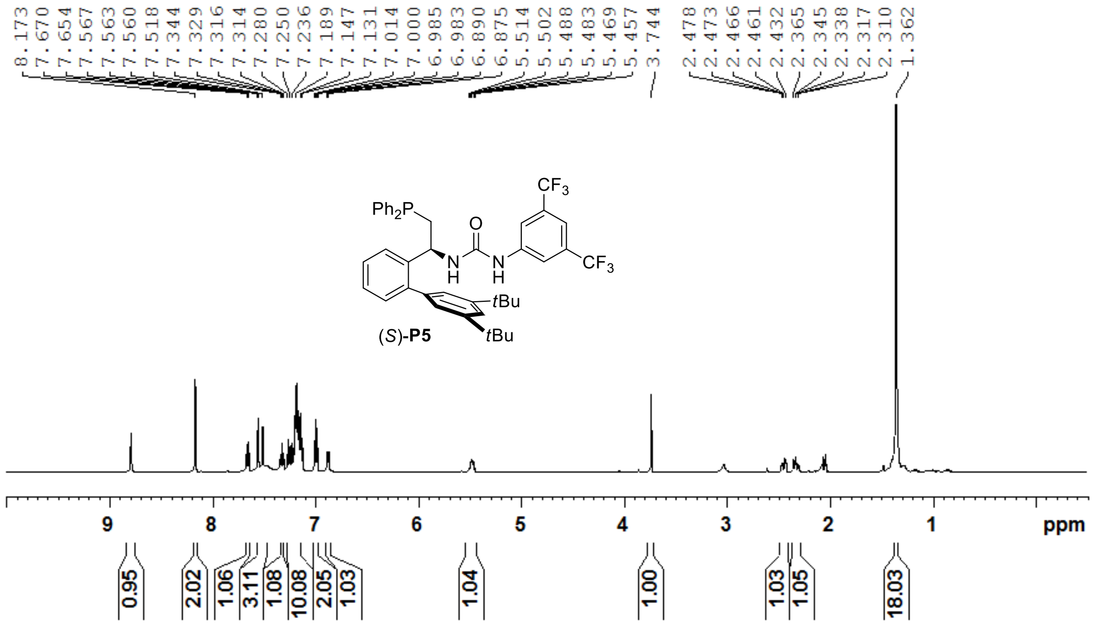

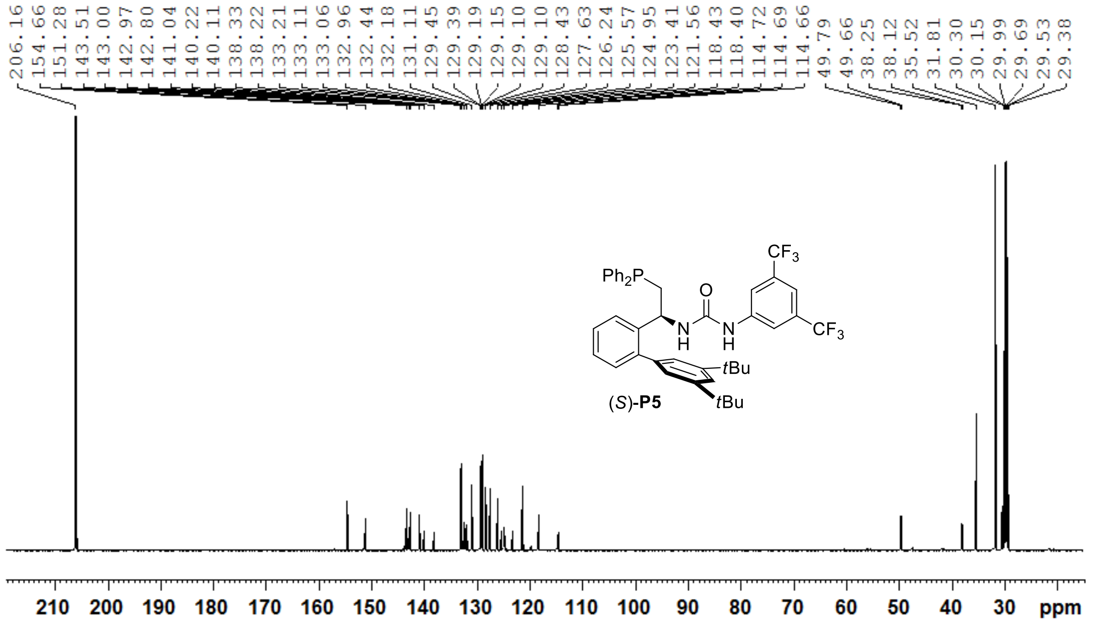

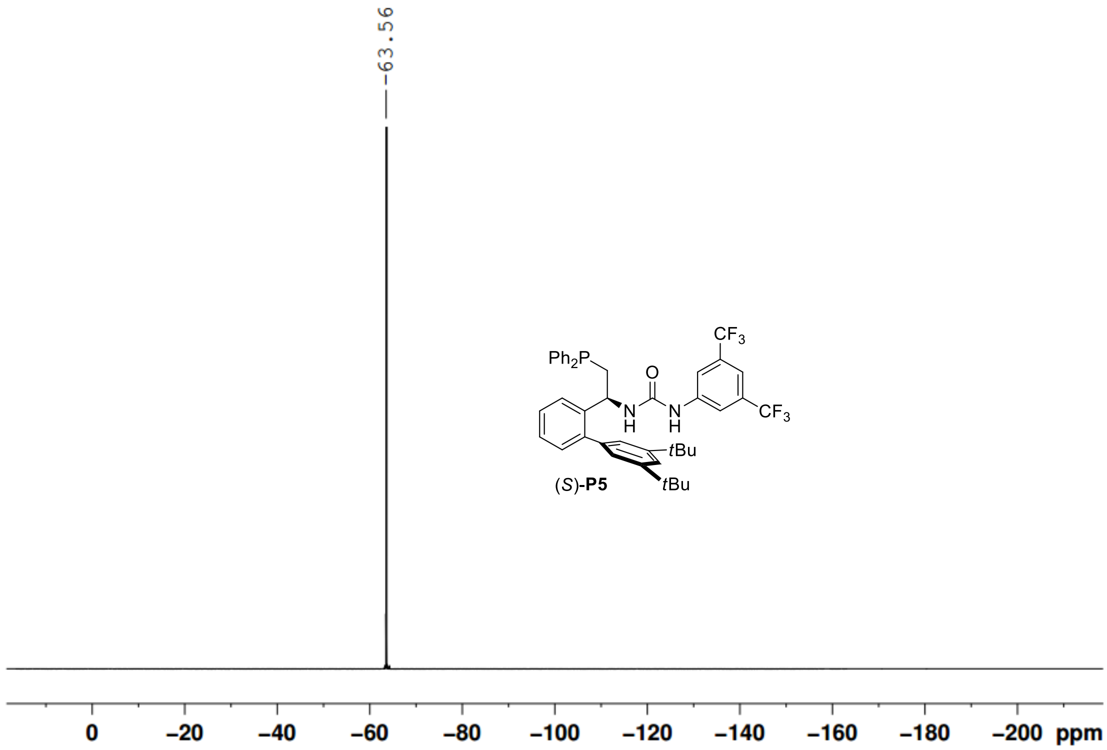
−63.56
Ph2P
O
N
H
N
H
CF3
CF3
tBu
tBu
(S)-P5
0
−20
−40
−60
−80
−100
−120
−140
−160
−180
−200
ppm

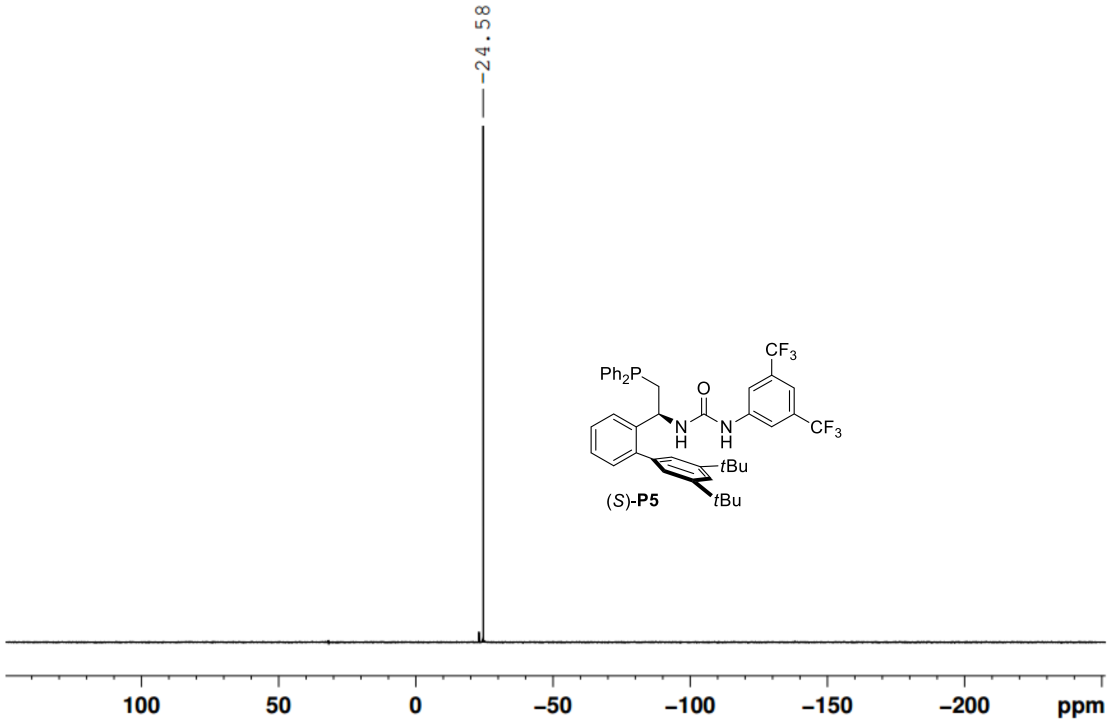
−24.58
Ph2P
O
N
H
N
H
CF3
CF3
tBu
tBu
(S)-P5
100
50
0
−50
−100
−150
−200
ppm

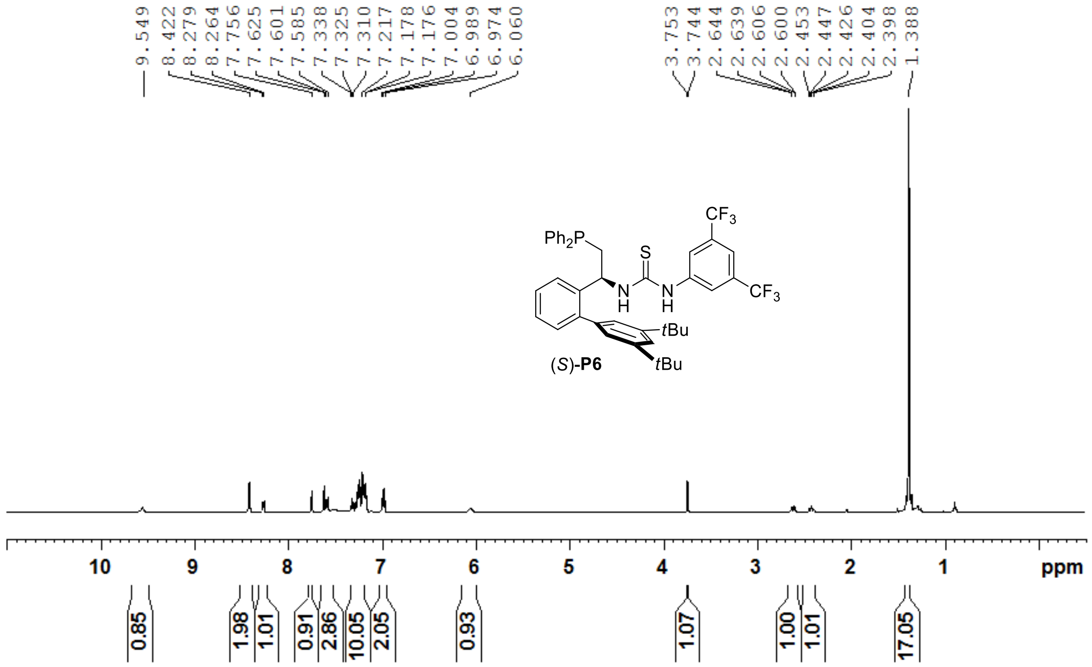
9.549
8.422
8.279
8.264
7.756
7.625
7.601
7.585
7.338
7.325
7.310
7.217
7.178
7.176
7.004
6.989
6.974
6.060
3.753
3.744
2.644
2.639
2.606
2.600
2.453
2.447
2.426
2.404
2.398
1.388
Ph$_2$P
S
N
H
N
H
CF$_3$
CF$_3$
tBu
tBu
(S)-P6
10
9
8
7
6
5
4
3
2
1
ppm
0.85
1.98
1.01
0.91
2.86
10.05
2.05
0.93
1.07
1.00
1.01
17.05

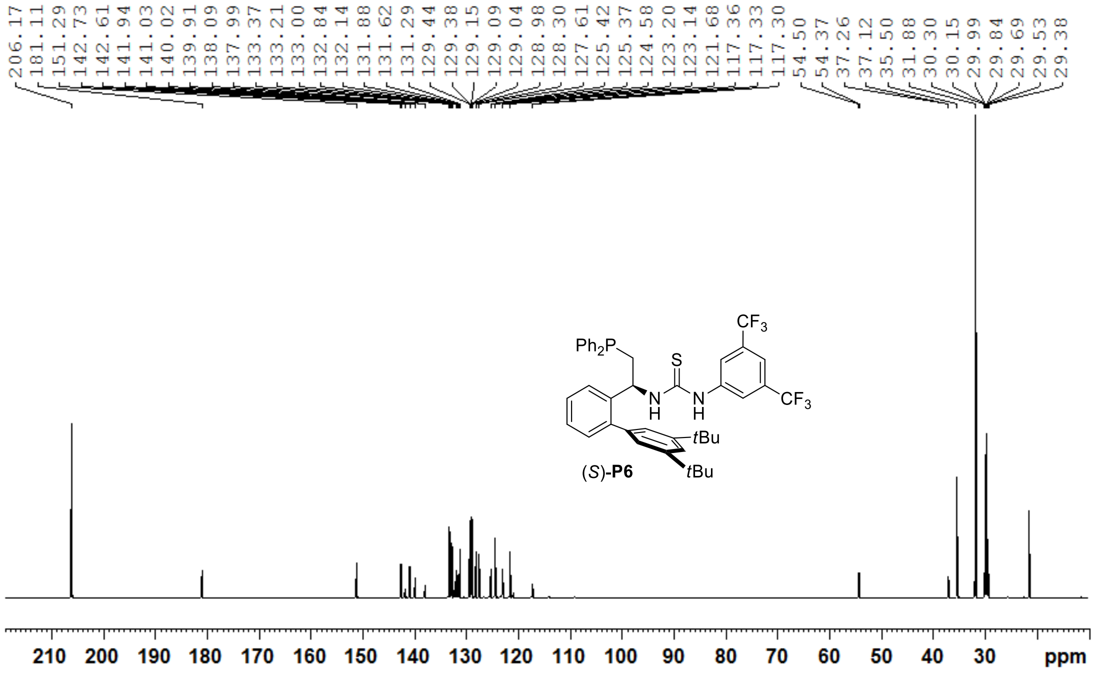
206.17
181.11
151.29
142.73
142.61
141.94
141.03
140.02
139.91
138.09
137.99
133.37
133.21
133.00
132.84
132.14
131.88
131.62
131.29
129.44
129.38
129.15
129.09
129.04
128.98
128.30
127.61
125.42
125.37
124.58
123.20
123.14
121.68
117.36
117.33
117.30
54.50
54.37
37.26
37.12
35.50
31.88
30.30
30.15
29.99
29.84
29.69
29.53
29.38
Ph$_2$P
S
N
H
N
H
CF$_3$
CF$_3$
tBu
tBu
(S)-P6
210
200
190
180
170
160
150
140
130
120
110
100
90
80
70
60
50
40
30
ppm

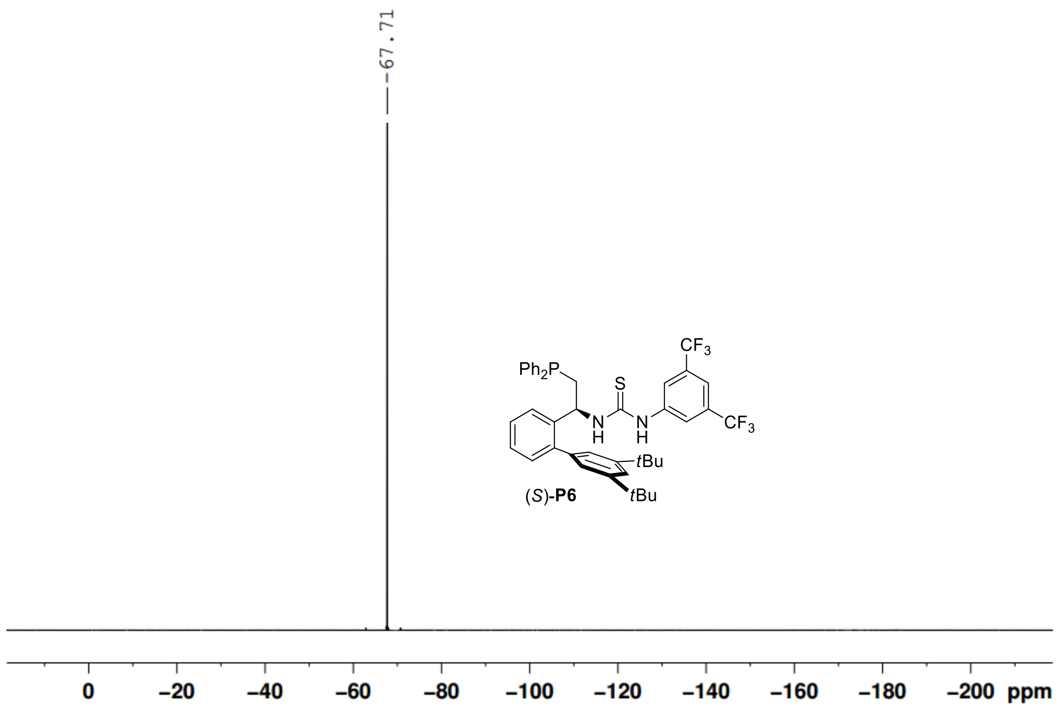

-67.71
Ph2P
S
N
H
N
H
CF3
CF3
tBu
tBu
(S)-P6
0
–20
–40
–60
–80
–100
–120
–140
–160
–180
–200
ppm

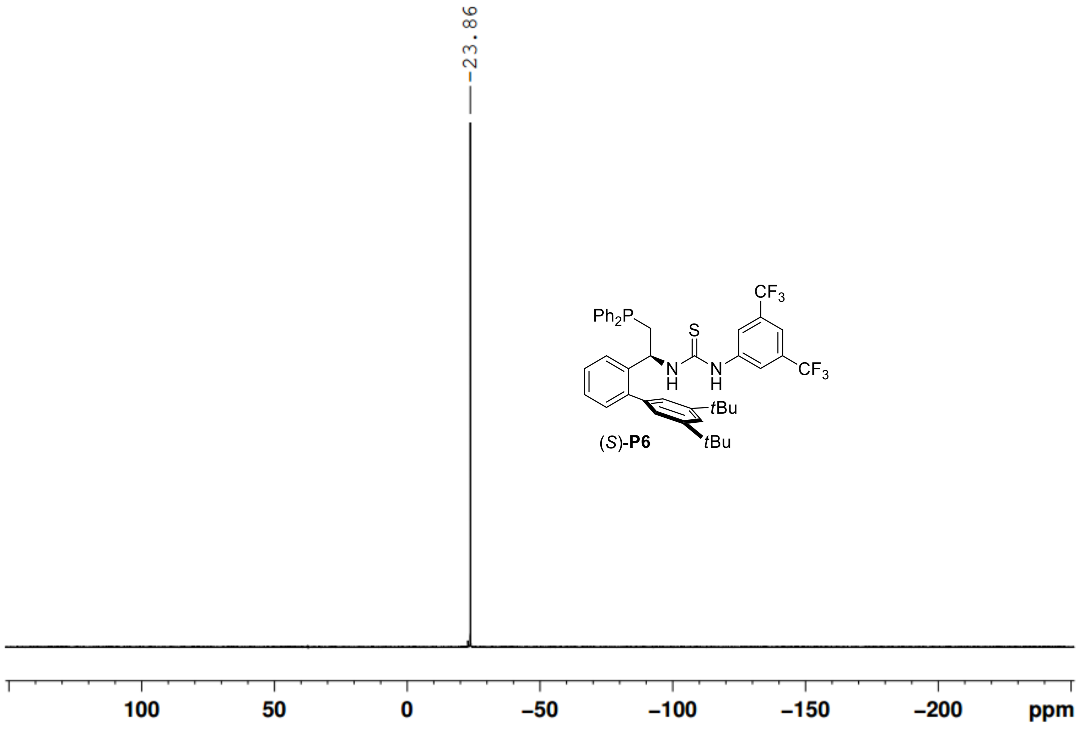

-23.86
Ph2P
S
N
H
N
H
CF3
CF3
tBu
tBu
(S)-P6
100
50
0
–50
–100
–150
–200
ppm

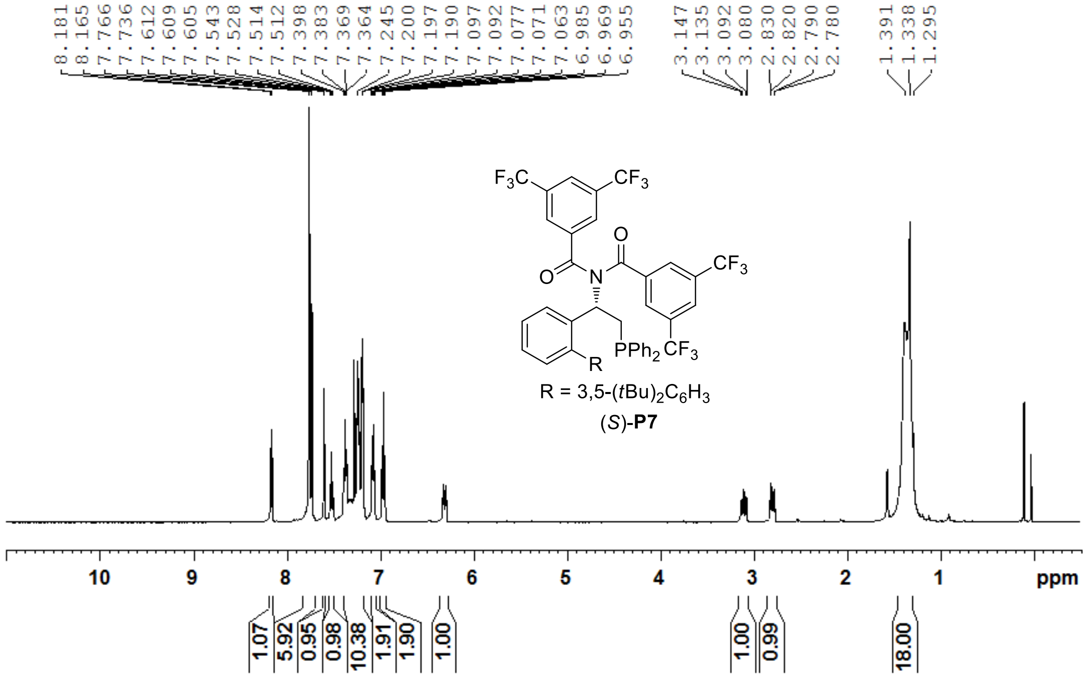
8.181 8.165 7.766 7.736 7.612 7.609 7.605 7.543 7.528 7.514 7.512 7.398 7.383 7.369 7.364 7.245 7.200 7.197 7.190 7.097 7.092 7.077 7.071 7.063 6.985 6.969 6.955
3.147 3.135 3.092 3.080 2.830 2.820 2.790 2.780
1.391 1.338 1.295
F3C
CF3
O
O
N
CF3
PPh2
CF3
R
R = 3,5-(tBu)2C6H3
(S)-P7
10 9 8 7 6 5 4 3 2 1 ppm
1.07 5.92 0.95 0.98 10.38 1.91 1.90 1.00
1.00 0.99
18.00

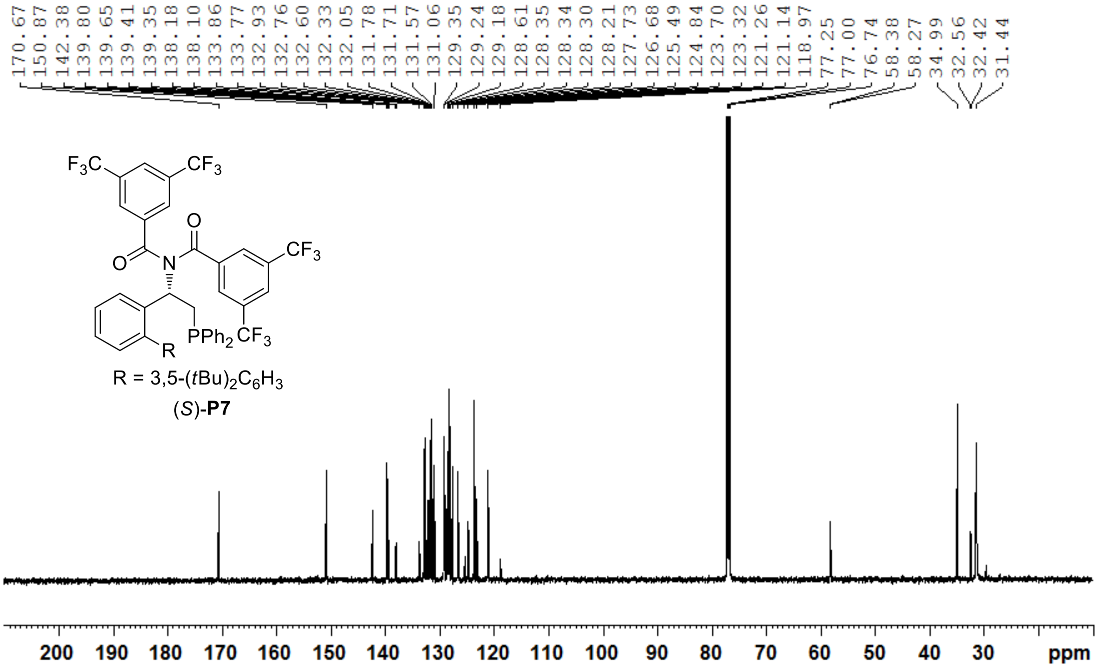
170.67 150.87 142.38 139.80 139.65 139.41 139.35 138.18 138.10 133.86 133.77 132.93 132.76 132.60 132.33 132.05 131.78 131.71 131.57 131.06 129.35 129.24 129.18 128.61 128.35 128.34 128.30 128.21 127.73 126.68 125.49 124.84 123.70 123.32 121.26 121.14 118.97 77.25 77.00 76.74 58.38 58.27 34.99 32.56 32.42 31.44
F3C
CF3
O
O
N
CF3
PPh2
CF3
R
R = 3,5-(tBu)2C6H3
(S)-P7
200 190 180 170 160 150 140 130 120 110 100 90 80 70 60 50 40 30 ppm

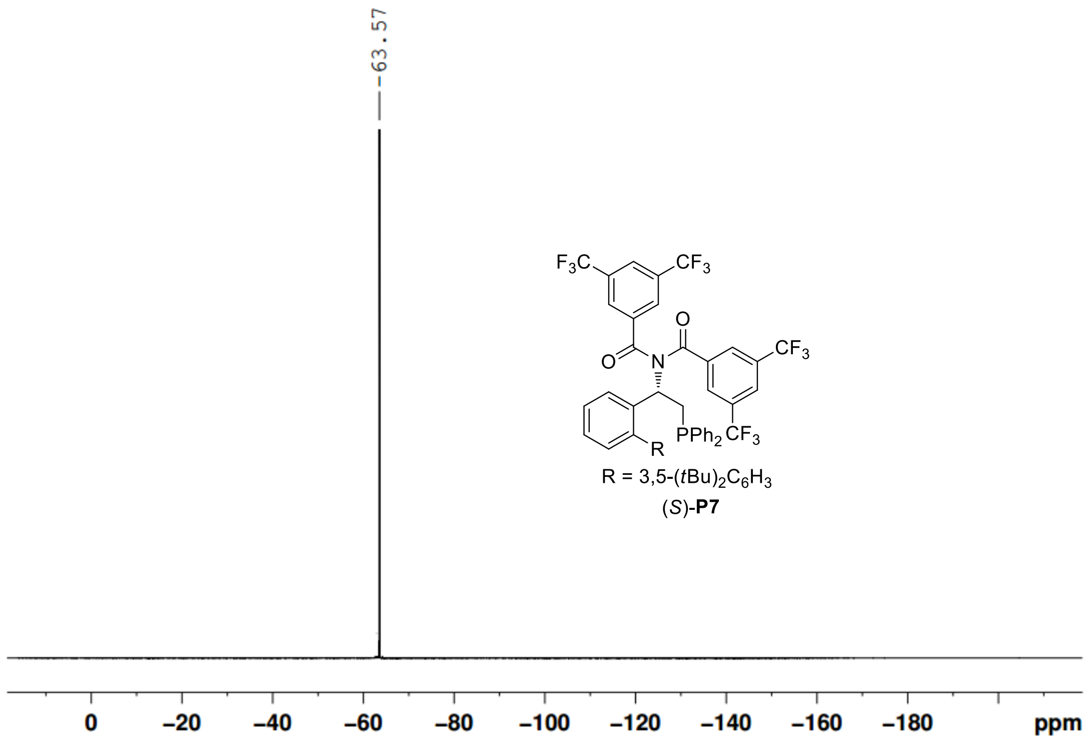
-63.57
F3C
CF3
O
O
N
CF3
PPh2
CF3
R
R = 3,5-(tBu)2C6H3
(S)-P7
0
−20
−40
−60
−80
−100
−120
−140
−160
−180
ppm

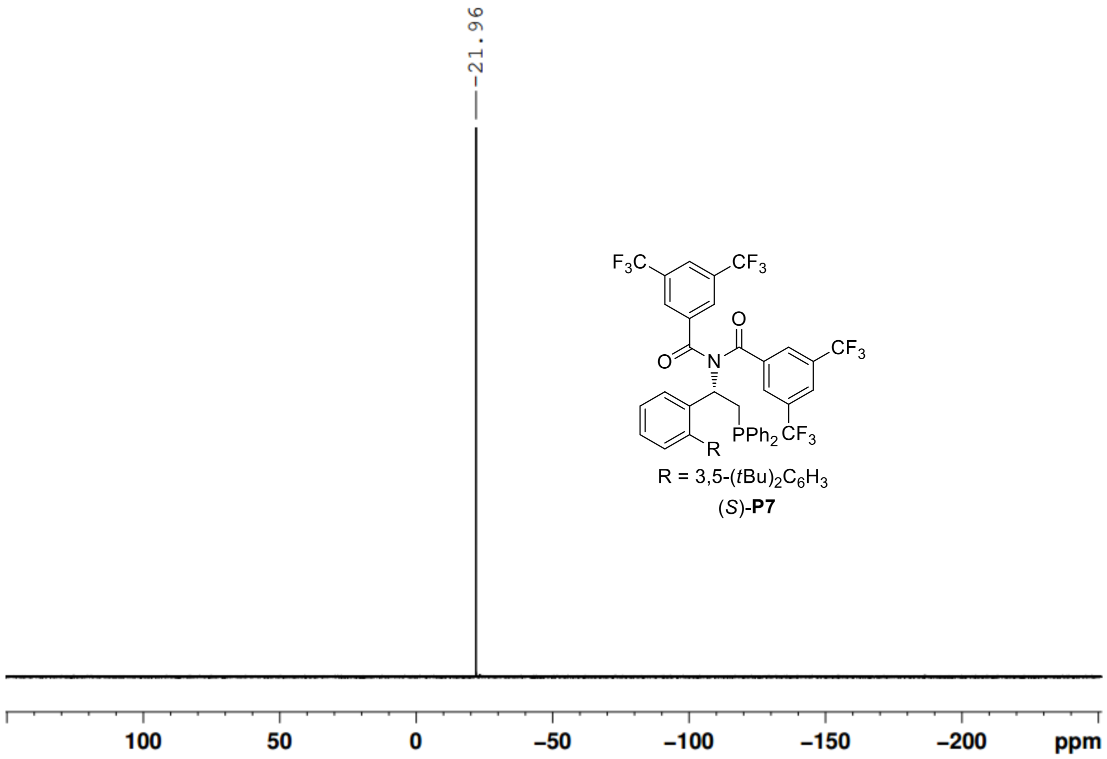
-21.96
F3C
CF3
O
O
N
CF3
PPh2
CF3
R
R = 3,5-(tBu)2C6H3
(S)-P7
100
50
0
−50
−100
−150
−200
ppm

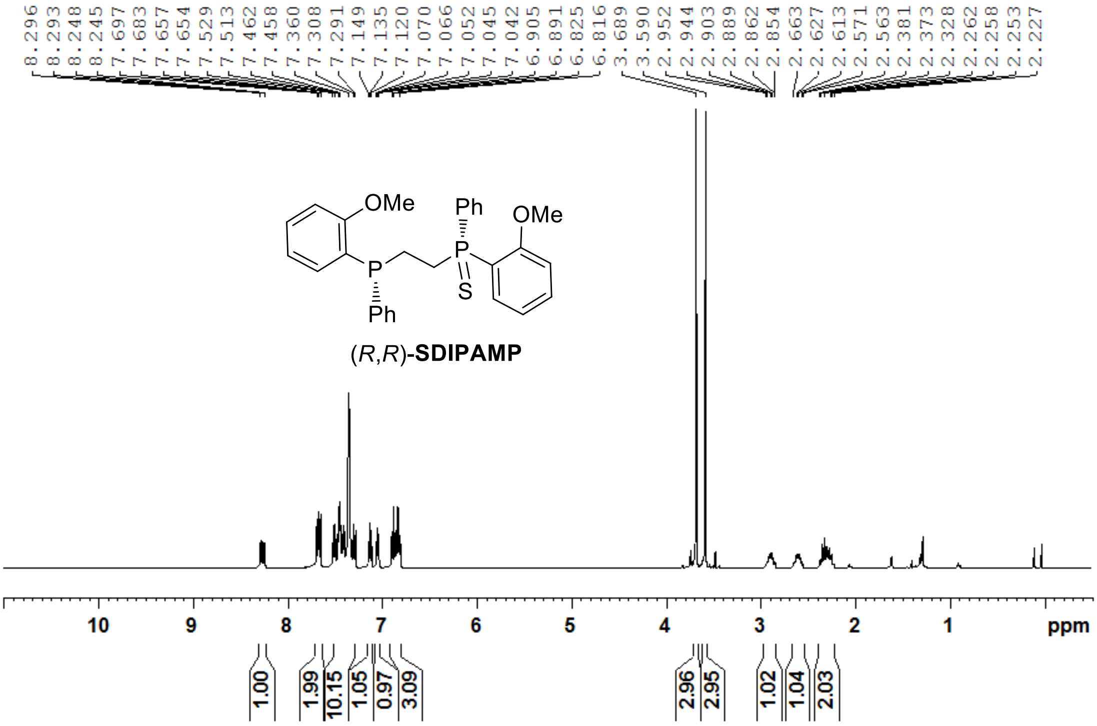
8.296 8.293 8.248 8.245 7.697 7.683 7.657 7.654 7.529 7.513 7.462 7.458 7.360 7.308 7.291 7.149 7.135 7.120 7.070 7.066 7.052 7.045 7.042 6.905 6.891 6.825 6.816 3.689 3.590 2.952 2.944 2.903 2.889 2.862 2.854 2.663 2.627 2.613 2.571 2.563 2.381 2.373 2.328 2.262 2.258 2.253 2.227
OMe
Ph
OMe
P
P
S
Ph
(R,R)-SDIPAMP
10 9 8 7 6 5 4 3 2 1 ppm
1.00 1.99 10.15 1.05 0.97 3.09 2.96 2.95 1.02 1.04 2.03

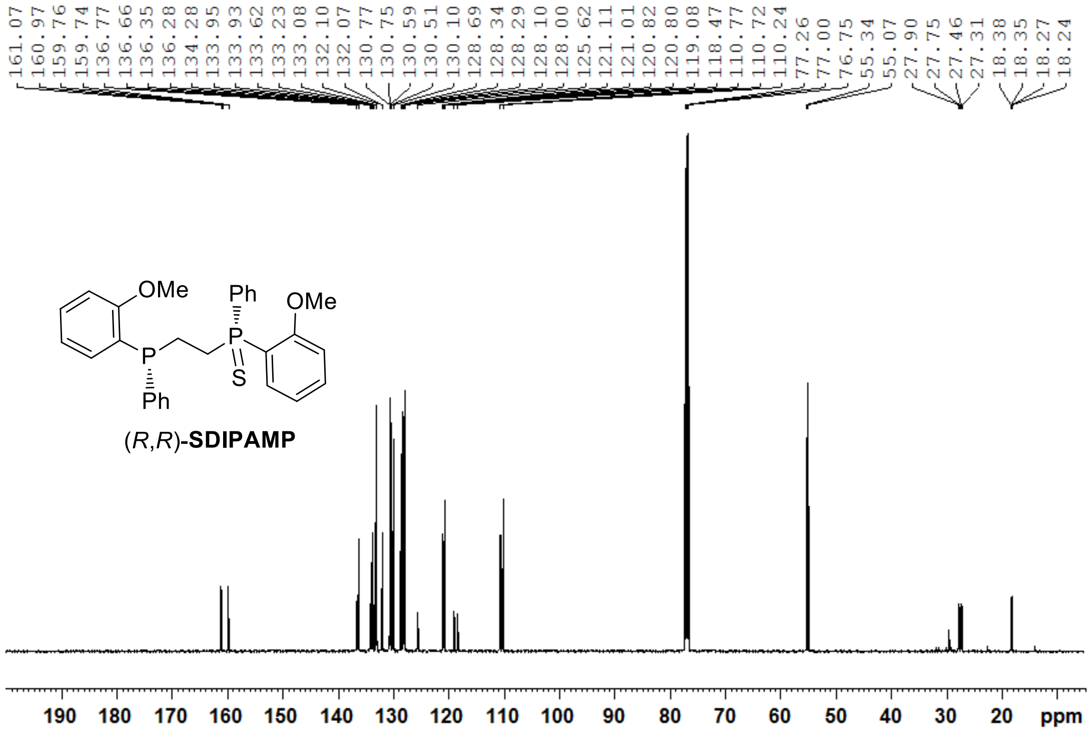
161.07 160.97 159.76 159.74 136.77 136.66 136.35 136.28 134.28 133.95 133.93 133.62 133.23 133.08 132.10 132.07 130.77 130.75 130.59 130.51 130.10 128.69 128.34 128.29 128.10 128.00 125.62 121.11 121.01 120.82 120.80 119.08 118.47 110.77 110.72 110.24 77.26 77.00 76.75 55.34 55.07 27.90 27.75 27.46 27.31 18.38 18.35 18.27 18.24
OMe
Ph
OMe
P
P
S
Ph
(R,R)-SDIPAMP
190 180 170 160 150 140 130 120 110 100 90 80 70 60 50 40 30 20 ppm

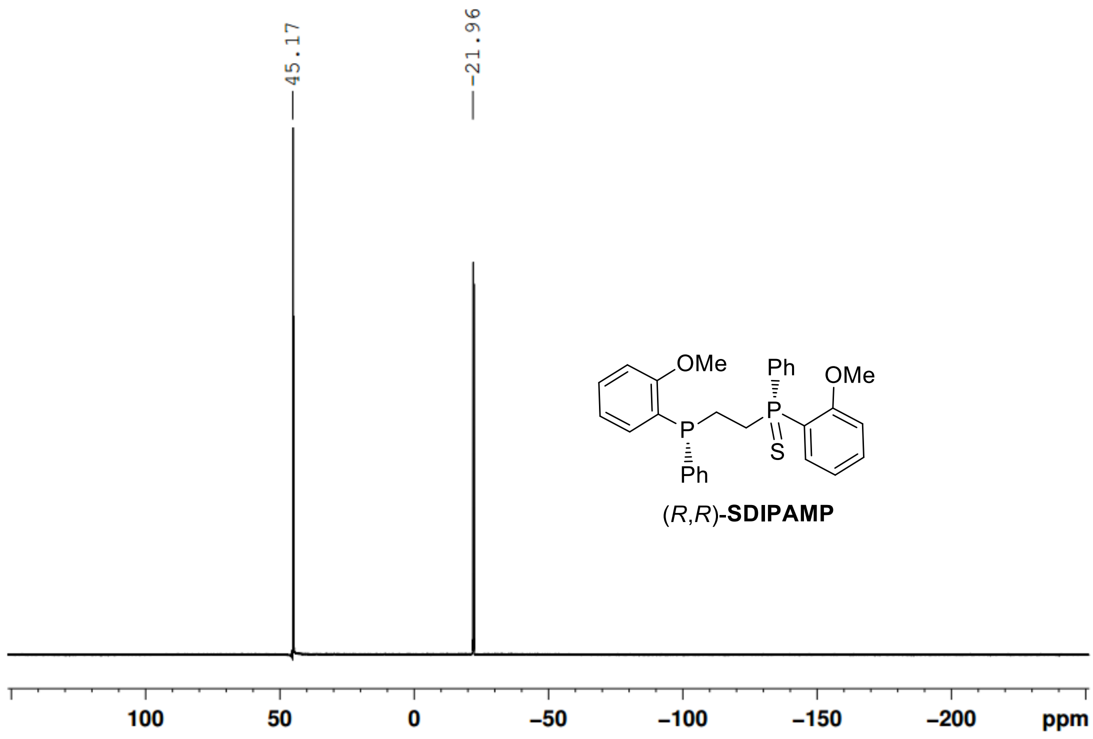
45.17
-21.96
OMe
Ph
OMe
P
P
S
Ph
(R,R)-SDIPAMP
100
50
0
-50
-100
-150
-200
ppm

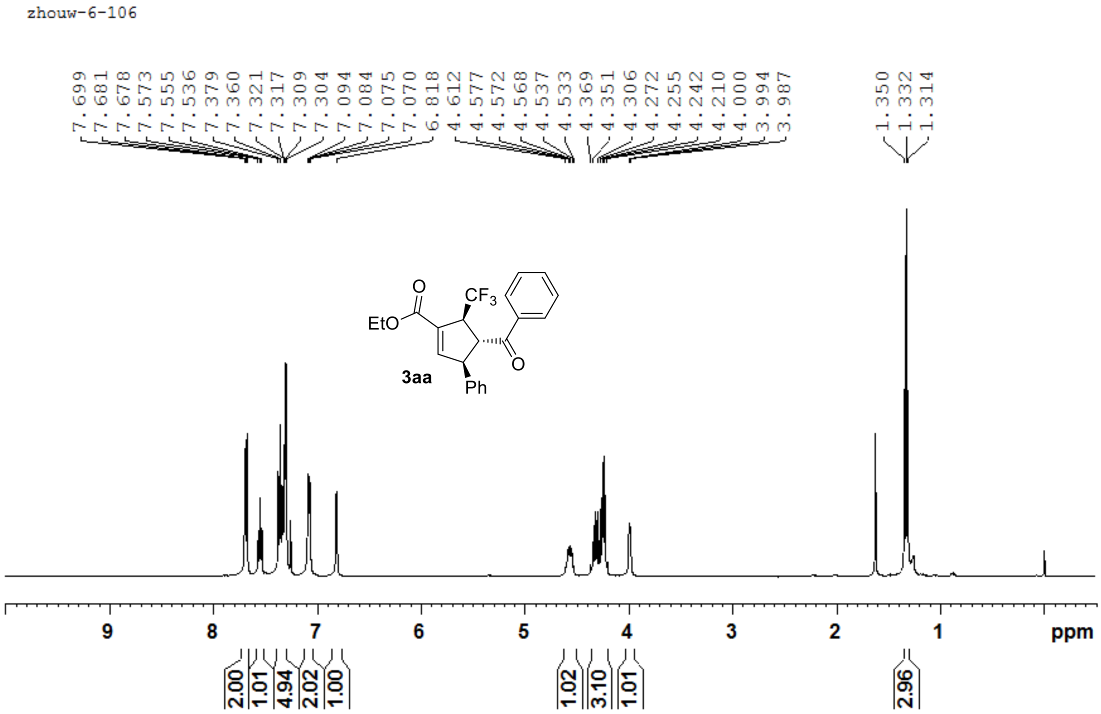
zhouw-6-106
7.699
7.681
7.678
7.573
7.555
7.536
7.379
7.360
7.321
7.317
7.309
7.304
7.094
7.084
7.075
7.070
6.818
4.612
4.577
4.572
4.568
4.537
4.533
4.369
4.351
4.306
4.272
4.255
4.242
4.210
4.000
3.994
3.987
1.350
1.332
1.314
O
EtO
CF3
O
3aa
Ph
9
8
7
6
5
4
3
2
1
ppm
2.00
1.01
4.94
2.02
1.00
1.02
3.10
1.01
2.96

zhouw-6-106c

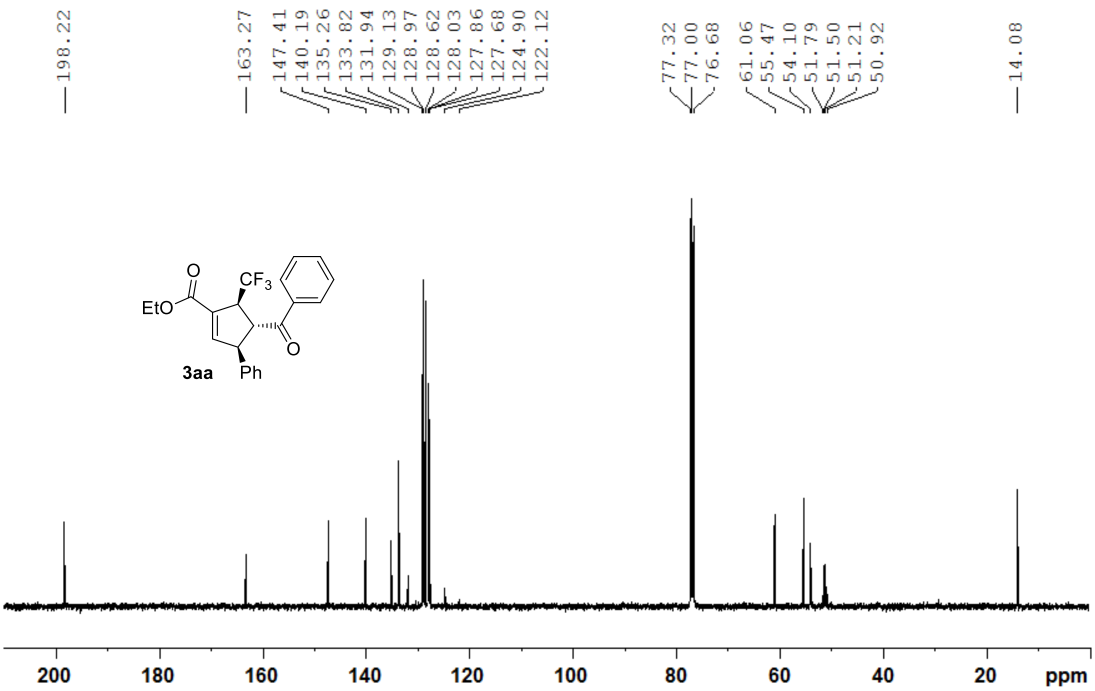

zhouw-6-106f

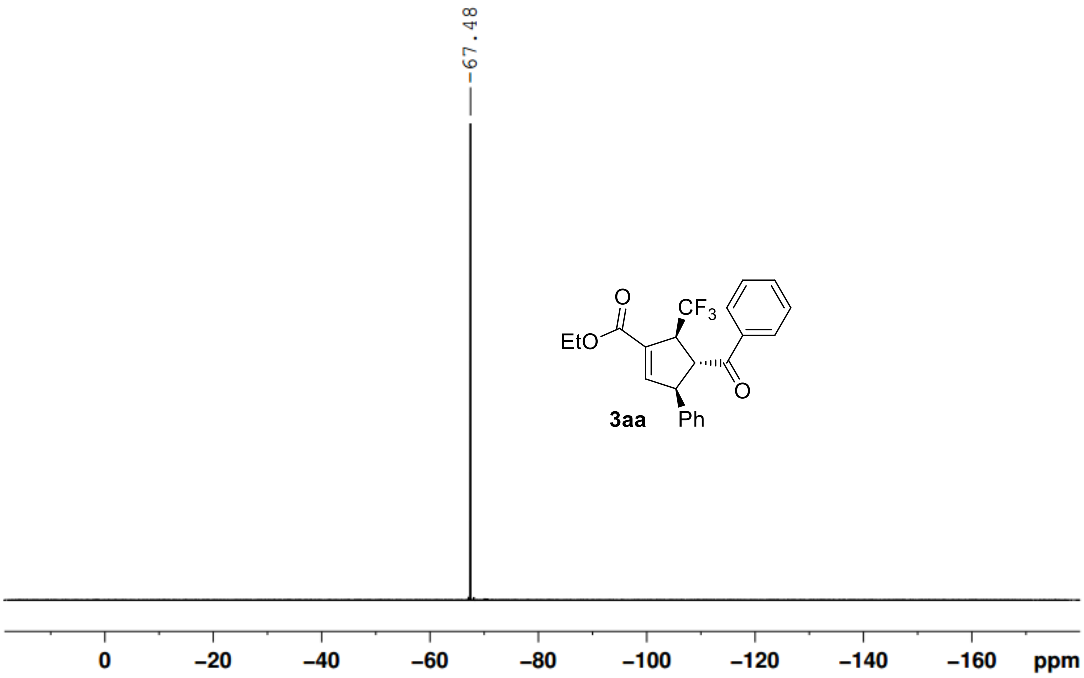

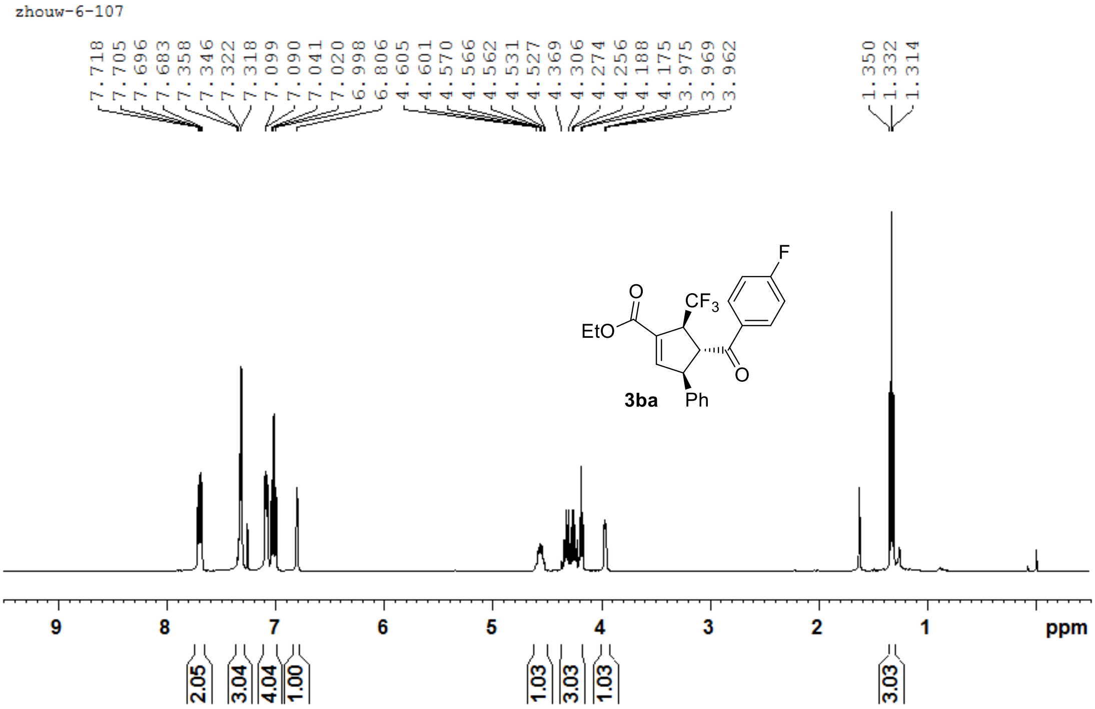
zhouw-6-107
7.718
7.705
7.696
7.683
7.358
7.346
7.322
7.318
7.099
7.090
7.041
7.020
6.998
6.806
4.605
4.601
4.570
4.566
4.562
4.531
4.527
4.369
4.306
4.274
4.256
4.188
4.175
3.975
3.969
3.962
1.350
1.332
1.314
F
O
CF3
EtO
O
3ba
Ph
9
8
7
6
5
4
3
2
1
ppm
2.05
3.04
4.04
1.00
1.03
3.03
1.03
3.03

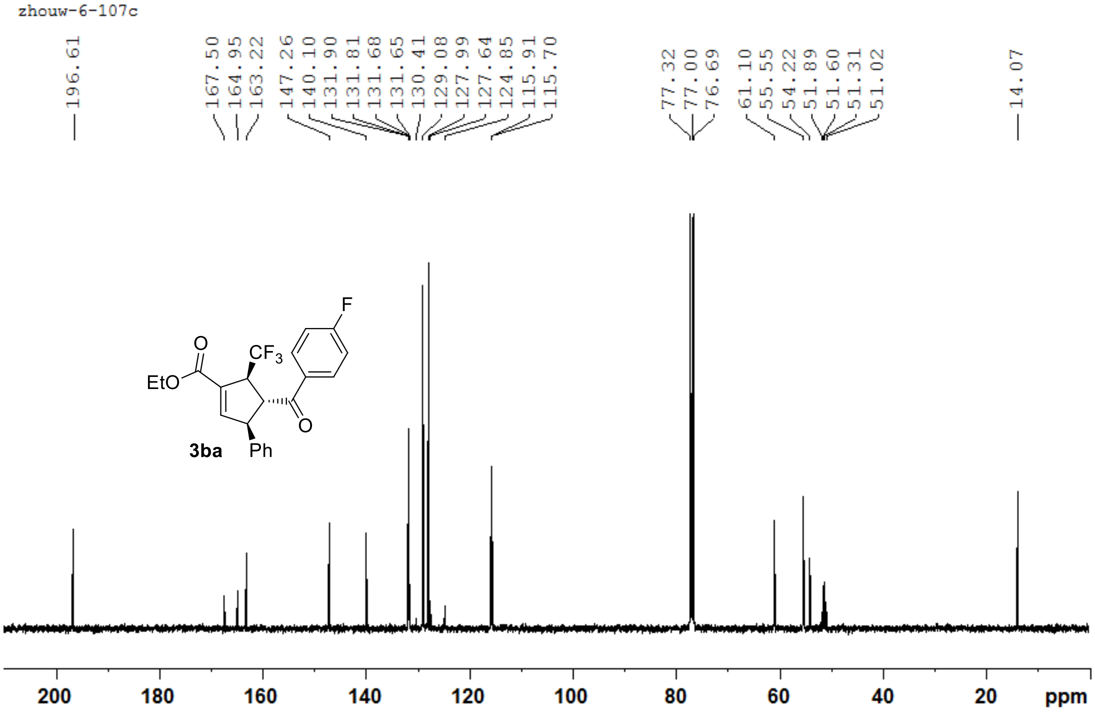
zhouw-6-107c
196.61
167.50
164.95
163.22
147.26
140.10
131.90
131.81
131.68
131.65
130.41
129.08
127.99
127.64
124.85
115.91
115.70
77.32
77.00
76.69
61.10
55.55
54.22
51.89
51.60
51.31
51.02
14.07
F
O
CF3
EtO
O
3ba
Ph
200
180
160
140
120
100
80
60
40
20
ppm

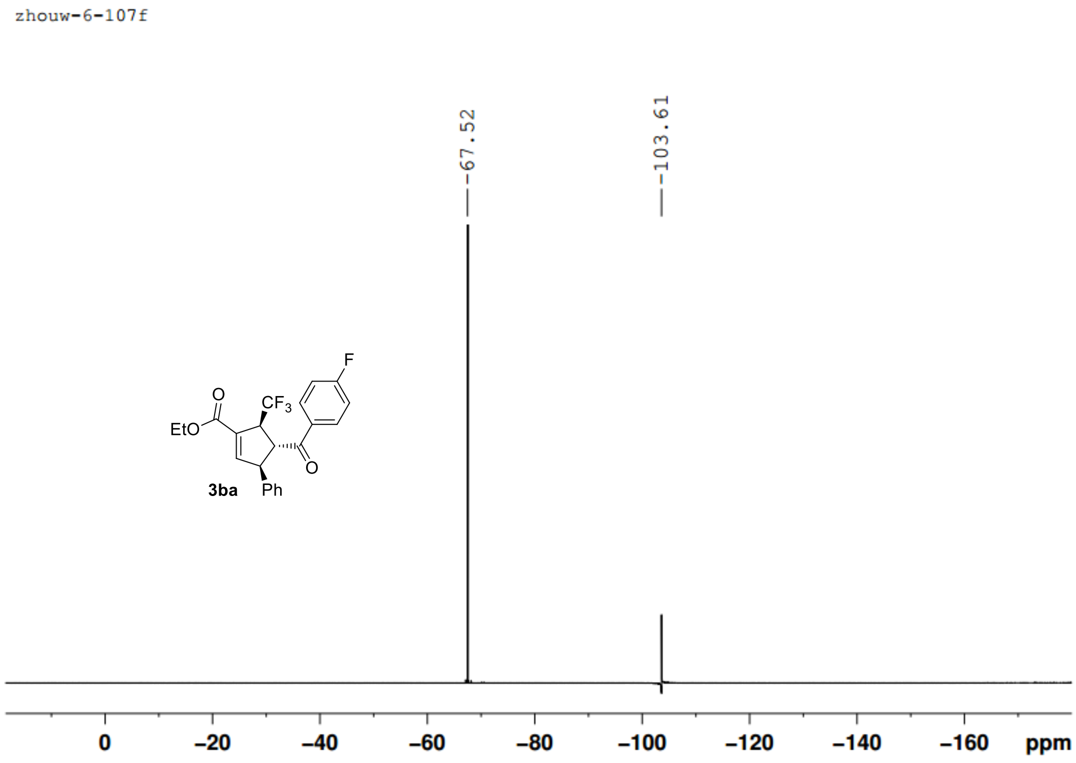
zhouw-6-107f
-67.52
-103.61
F
O
CF3
EtO
O
3ba
Ph
0
-20
-40
-60
-80
-100
-120
-140
-160
ppm

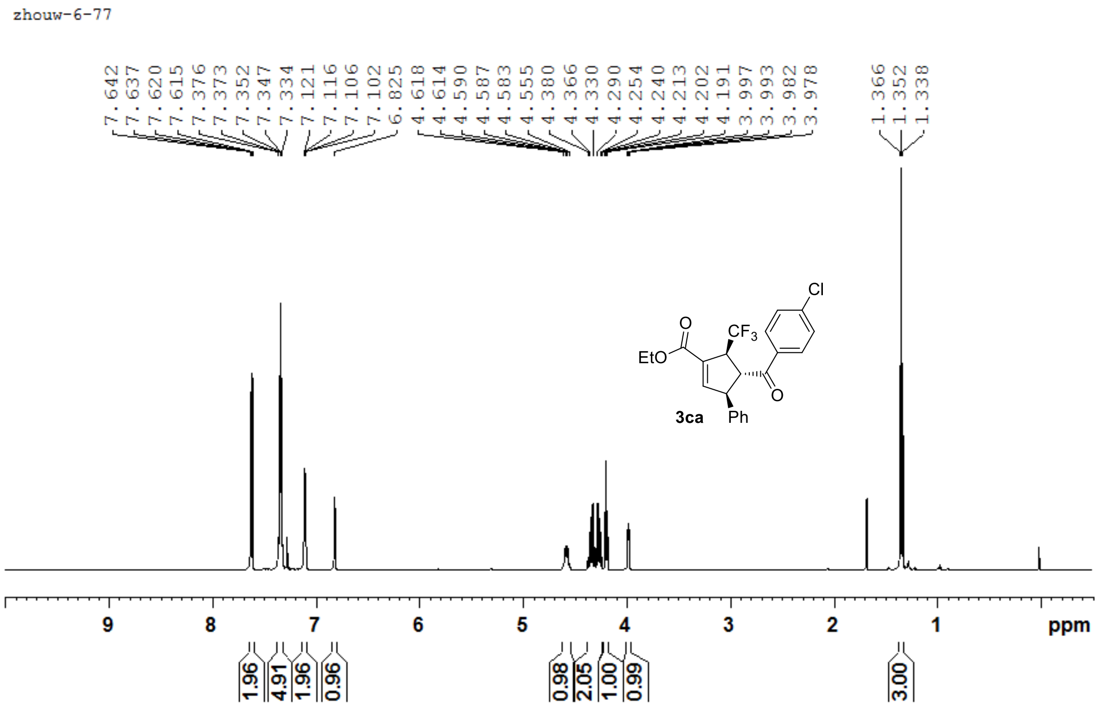
zhouw-6-77
7.642
7.637
7.620
7.615
7.376
7.373
7.352
7.347
7.334
7.121
7.116
7.106
7.102
6.825
4.618
4.614
4.590
4.587
4.583
4.555
4.380
4.366
4.330
4.290
4.254
4.240
4.213
4.202
4.191
3.997
3.993
3.982
3.978
1.366
1.352
1.338
Cl
O
CF3
EtO
O
3ca
Ph
9
8
7
6
5
4
3
2
1
ppm
1.96
4.91
1.96
0.96
0.98
2.05
1.00
0.99
3.00

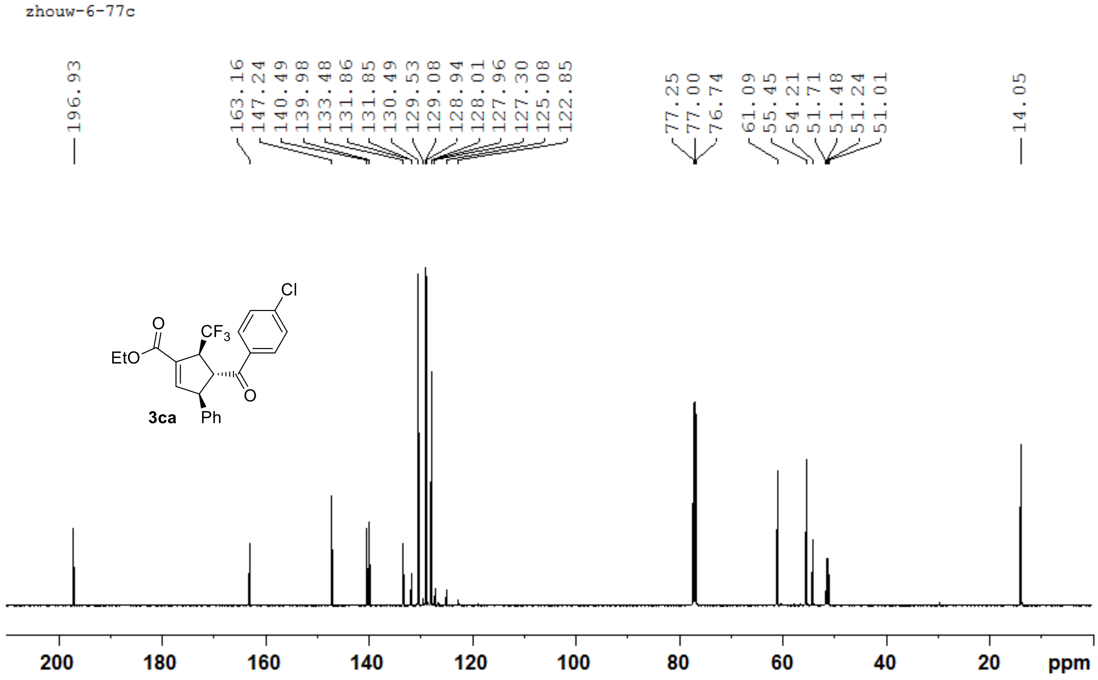
zhouw-6-77c
196.93
163.16
147.24
140.49
139.98
133.48
131.86
131.85
130.49
129.53
129.08
128.94
128.01
127.96
127.30
125.08
122.85
77.25
77.00
76.74
61.09
55.45
54.21
51.71
51.48
51.24
51.01
14.05
O
EtO
CF3
Cl
O
3ca
Ph
200
180
160
140
120
100
80
60
40
20
ppm

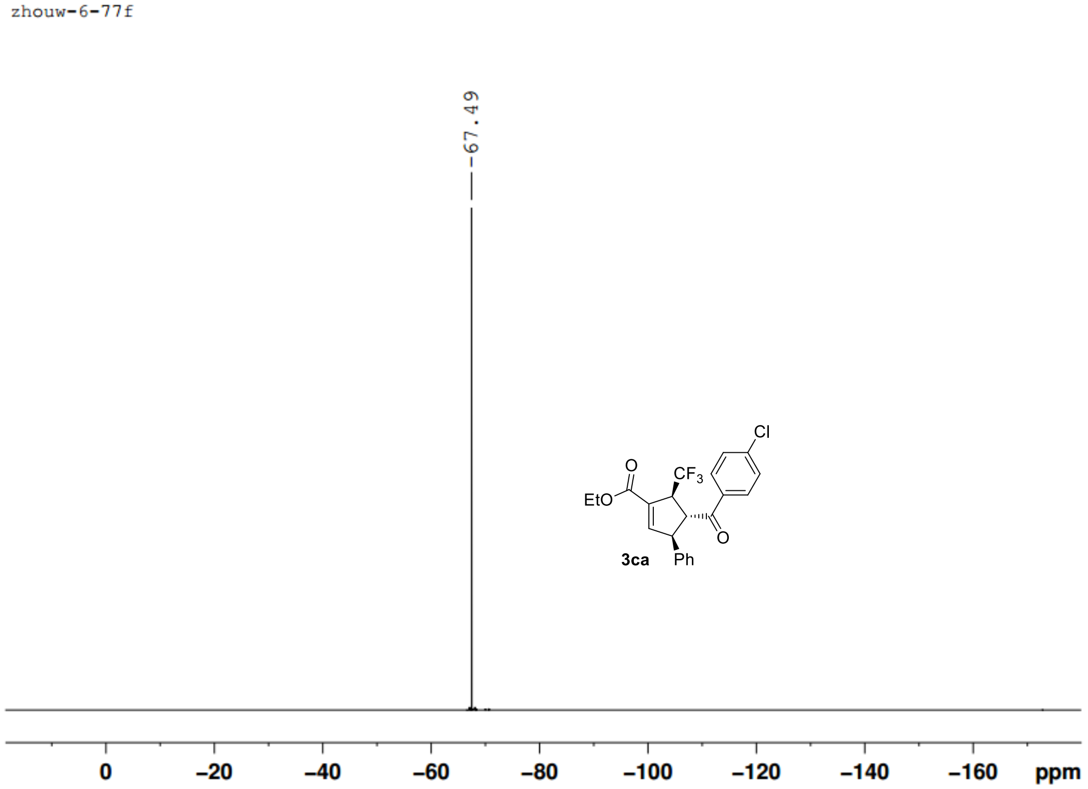
zhouw-6-77f
-67.49
Cl
O
CF3
EtO
O
3ca
Ph
0
−20
−40
−60
−80
−100
−120
−140
−160
ppm

zhouw-6-108

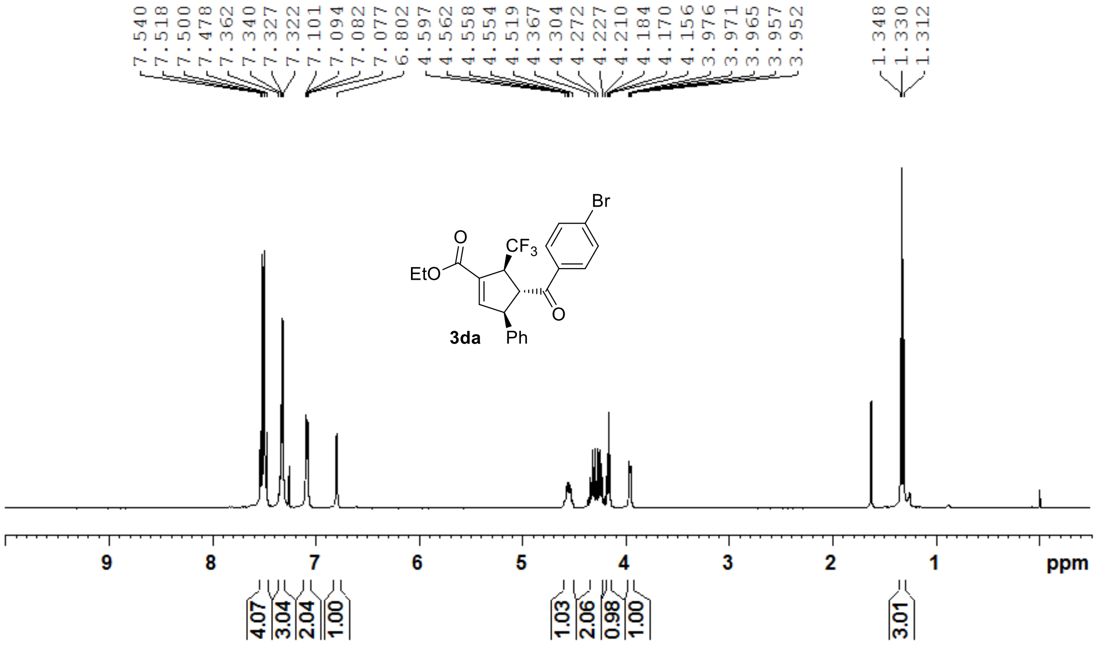

zhouw-6-108c

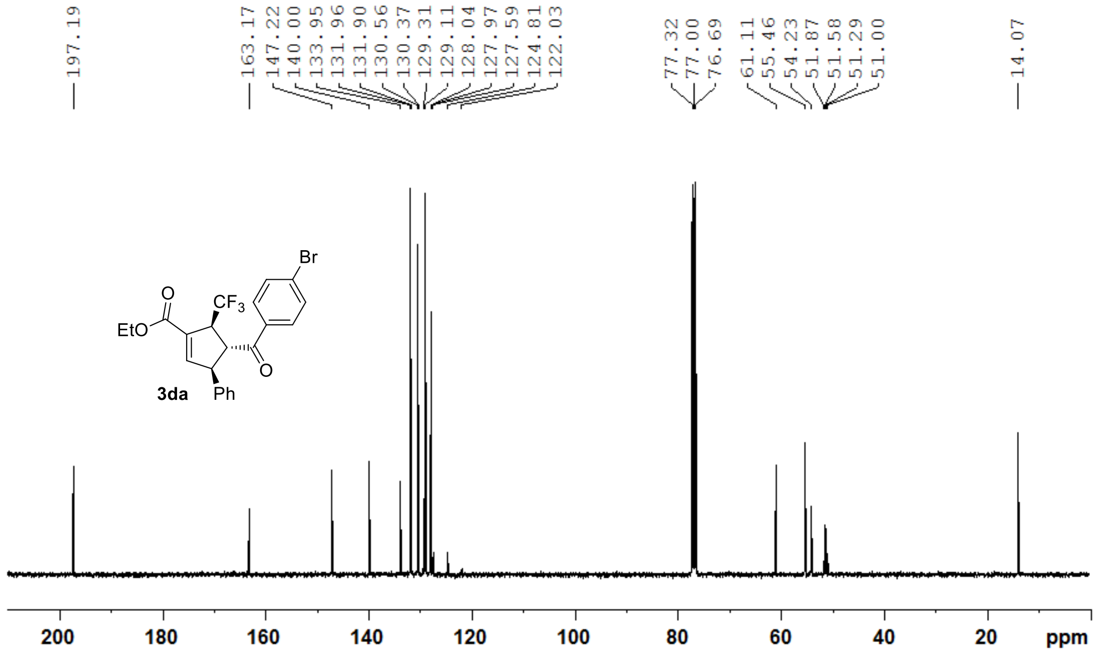

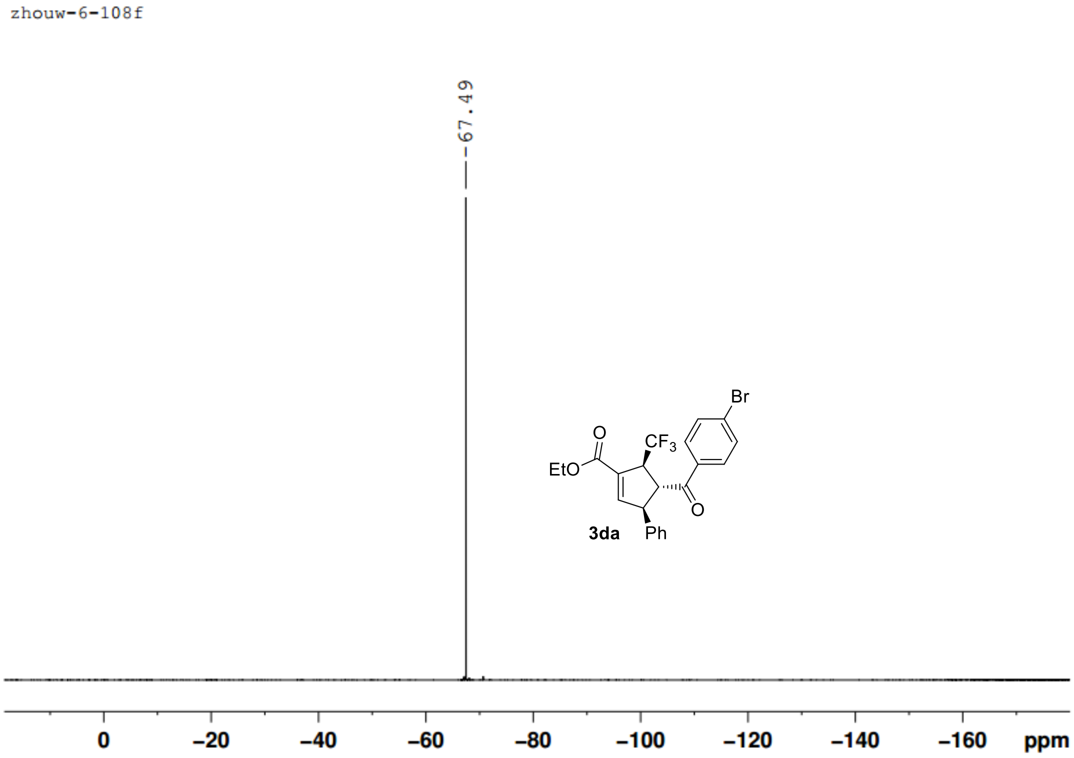
zhouw-6-108f
-67.49
Br
CF3
EtO
O
3da
Ph
0
−20
−40
−60
−80
−100
−120
−140
−160
ppm

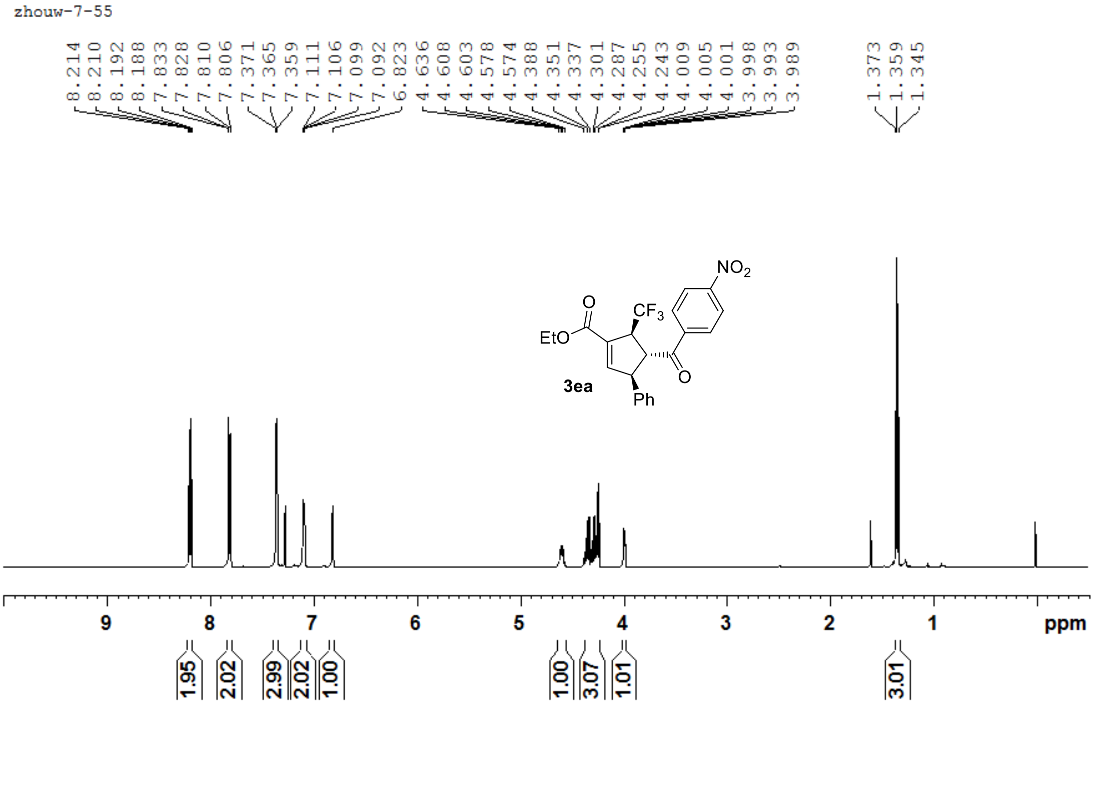
zhouw-7-55
8.214
8.210
8.192
8.188
7.833
7.828
7.810
7.806
7.371
7.365
7.359
7.111
7.106
7.099
7.092
6.823
4.636
4.608
4.603
4.578
4.574
4.388
4.351
4.337
4.301
4.287
4.255
4.243
4.009
4.005
4.001
3.998
3.993
3.989
1.373
1.359
1.345
NO2
CF3
EtO
O
3ea
Ph
9
8
7
6
5
4
3
2
1
ppm
1.95
2.02
2.99
2.02
1.00
1.00
3.07
1.01
3.01

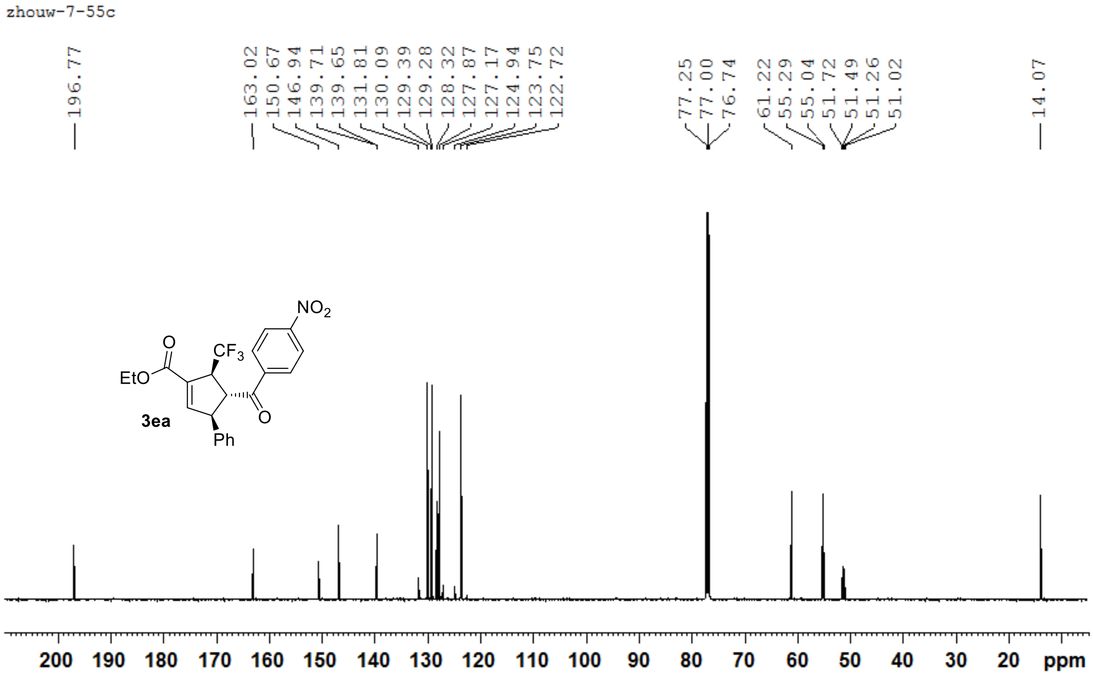
zhouw-7-55c
196.77
163.02
150.67
146.94
139.71
139.65
131.81
130.09
129.39
129.28
128.32
127.87
127.17
124.94
123.75
122.72
77.25
77.00
76.74
61.22
55.29
55.04
51.72
51.49
51.26
51.02
14.07
NO2
CF3
EtO
O
3ea
Ph
200 190 180 170 160 150 140 130 120 110 100 90 80 70 60 50 40 30 20 ppm

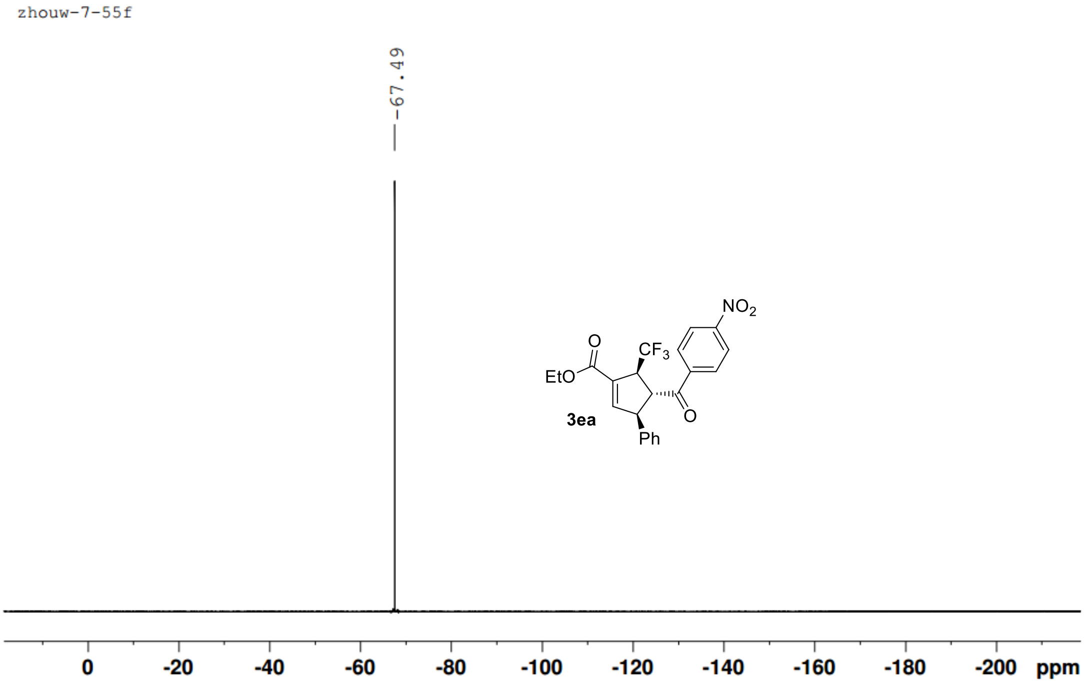
zhouw-7-55f
-67.49
NO2
CF3
EtO
O
3ea
Ph
0 -20 -40 -60 -80 -100 -120 -140 -160 -180 -200 ppm

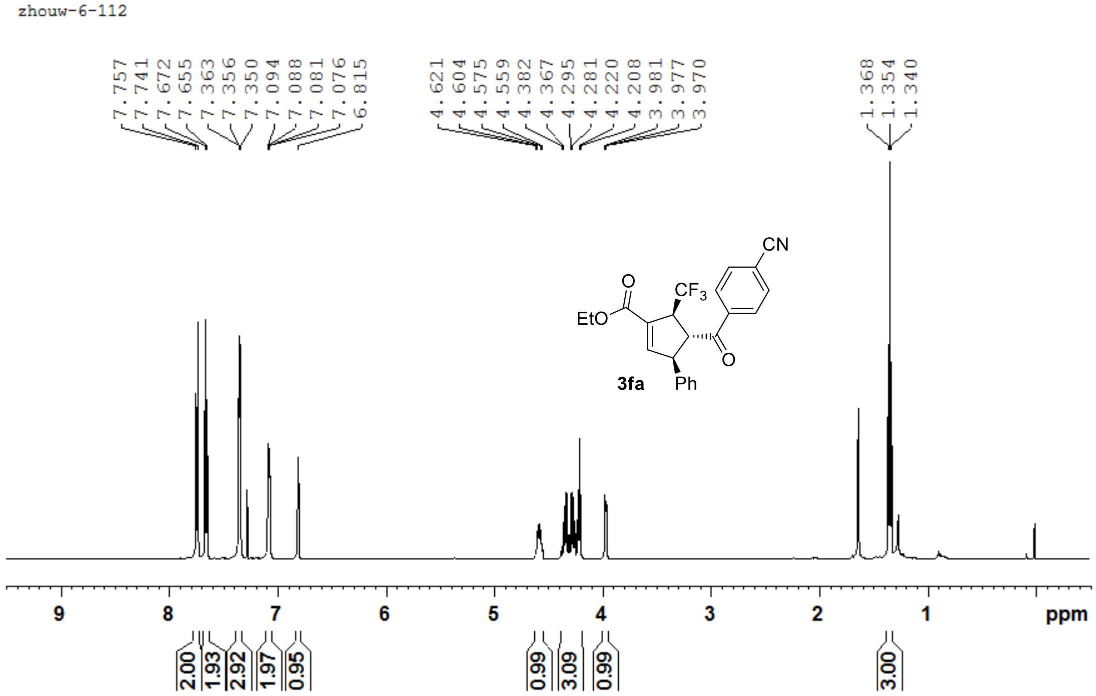

zhouw-6-112
7.757
7.741
7.672
7.655
7.363
7.356
7.350
7.094
7.088
7.081
7.076
6.815
4.621
4.604
4.575
4.559
4.382
4.367
4.295
4.281
4.220
4.208
3.981
3.977
3.970
1.368
1.354
1.340
CN
O
CF3
EtO
O
3fa
Ph
9
8
7
6
5
4
3
2
1
ppm
2.00
1.93
2.92
1.97
0.95
0.99
3.09
0.99
3.00

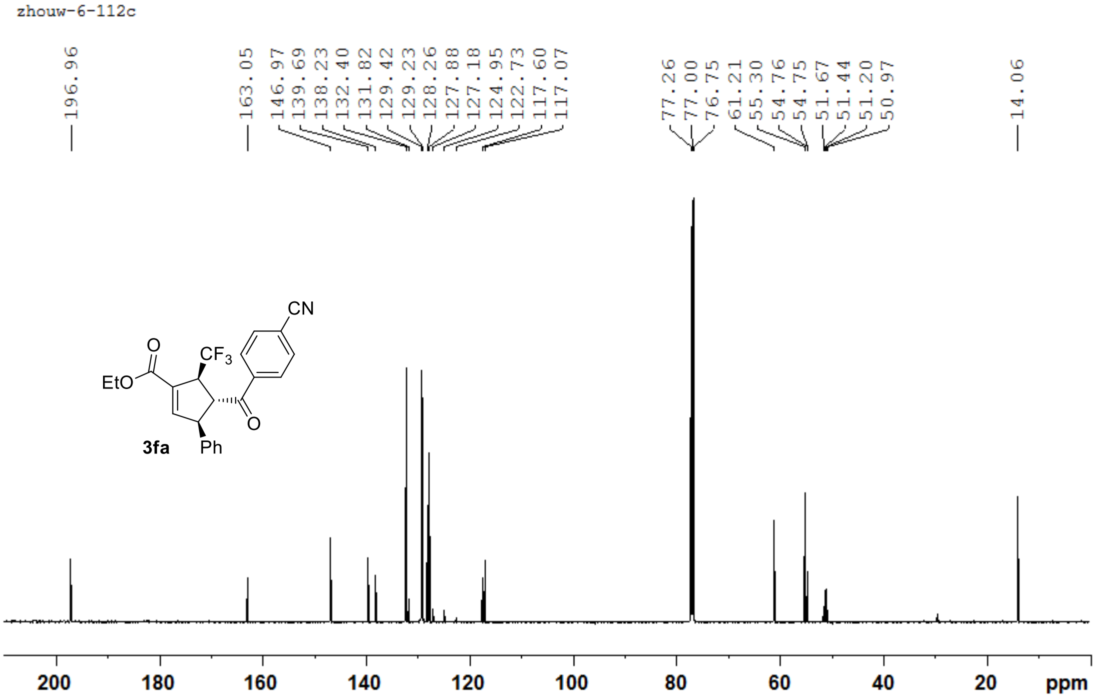

zhouw-6-112c
196.96
163.05
146.97
139.69
138.23
132.40
131.82
129.42
129.23
128.26
127.88
127.18
124.95
122.73
117.60
117.07
77.26
77.00
76.75
61.21
55.30
54.76
54.75
51.67
51.44
51.20
50.97
14.06
CN
O
CF3
EtO
O
3fa
Ph
200
180
160
140
120
100
80
60
40
20
ppm

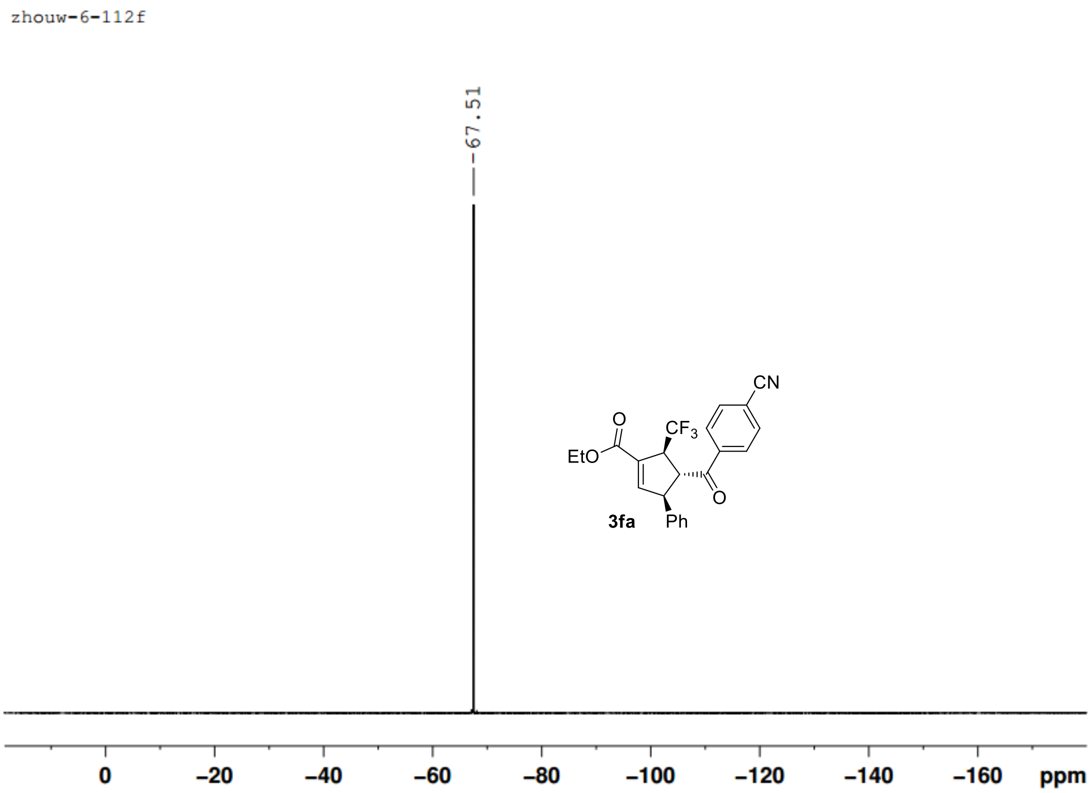
zhouw-6-112f
-67.51
CN
O
CF3
EtO
O
3fa
Ph
0
−20
−40
−60
−80
−100
−120
−140
−160
ppm

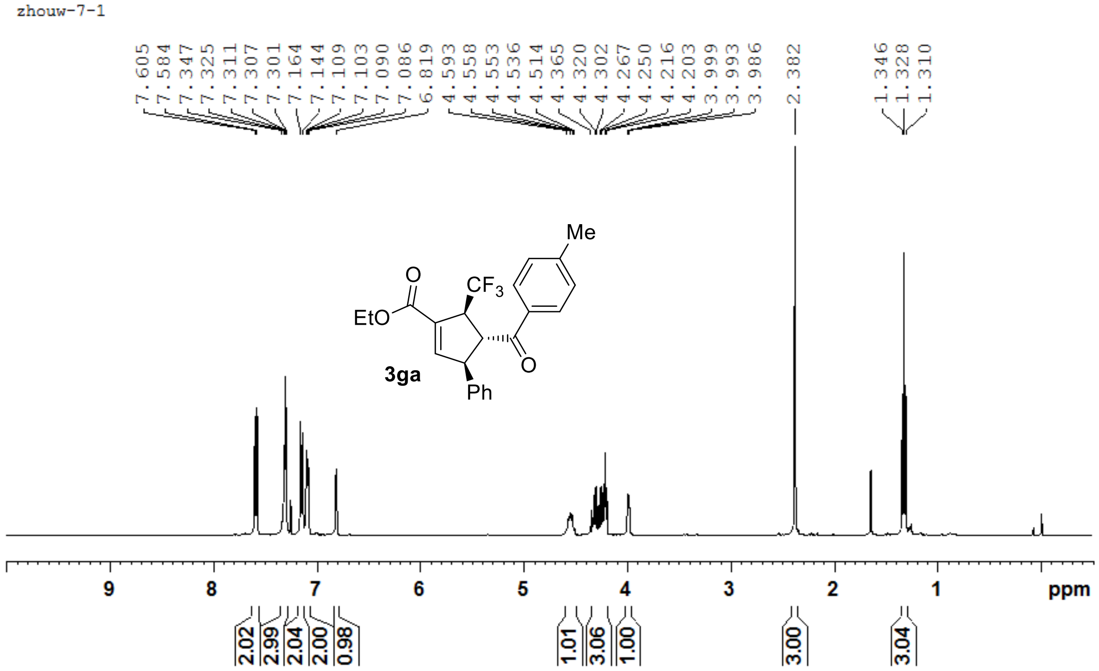
zhouw-7-1
7.605
7.584
7.347
7.325
7.311
7.307
7.301
7.164
7.144
7.109
7.103
7.090
7.086
6.819
4.593
4.558
4.553
4.536
4.514
4.365
4.320
4.302
4.267
4.250
4.216
4.203
3.999
3.993
3.986
2.382
1.346
1.328
1.310
Me
O
CF3
EtO
3ga
O
Ph
9
8
7
6
5
4
3
2
1
ppm
2.02
2.99
2.04
2.00
0.98
1.01
3.06
1.00
3.00
3.04

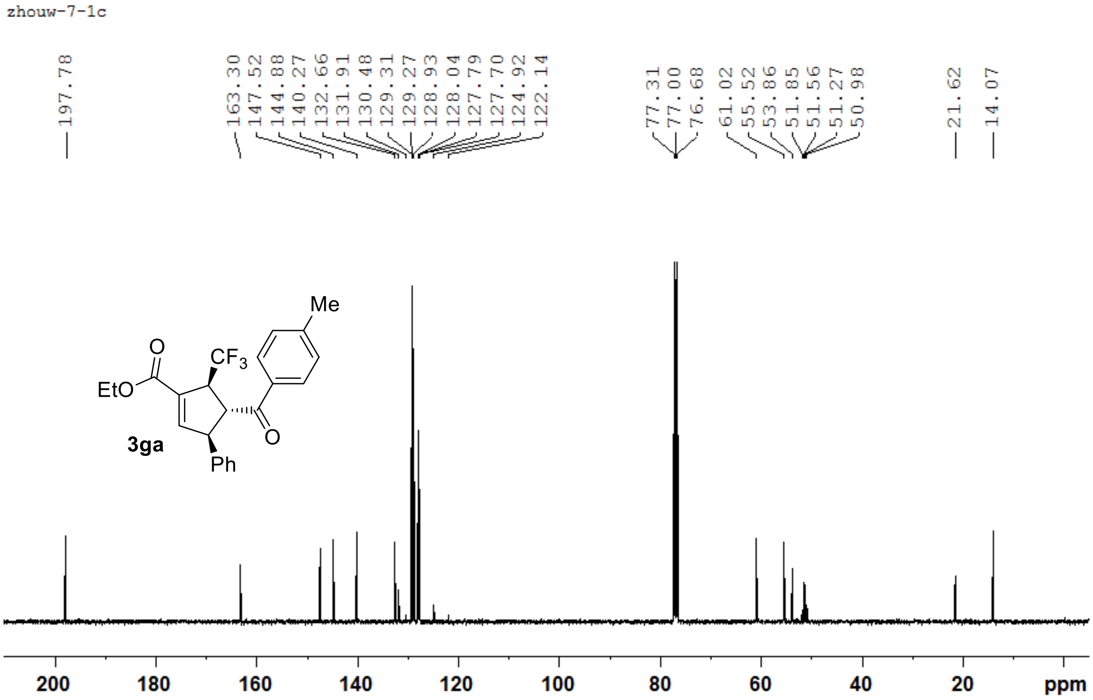
zhouw-7-1c
197.78
163.30
147.52
144.88
140.27
132.66
131.91
130.48
129.31
129.27
128.93
128.04
127.79
127.70
124.92
122.14
77.31
77.00
76.68
61.02
55.52
53.86
51.85
51.56
51.27
50.98
21.62
14.07
Me
O
CF3
EtO
O
3ga
Ph
200
180
160
140
120
100
80
60
40
20
ppm

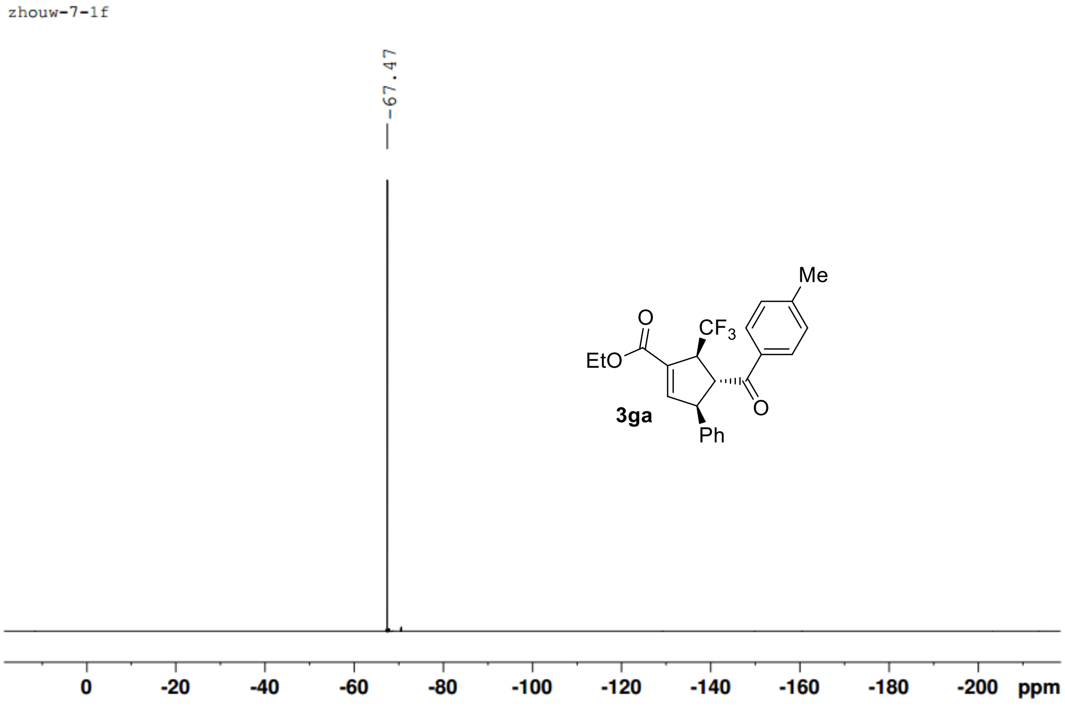
zhouw-7-1f
-67.47
Me
O
CF3
EtO
O
3ga
Ph
0
-20
-40
-60
-80
-100
-120
-140
-160
-180
-200
ppm

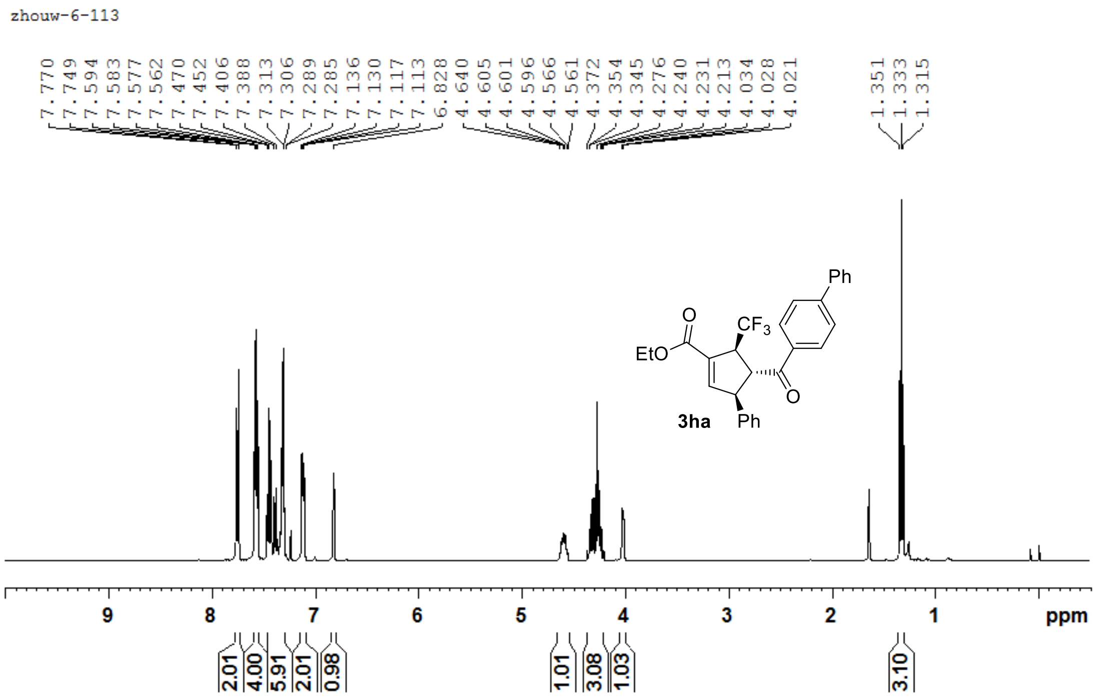
zhouw-6-113
7.770
7.749
7.594
7.583
7.577
7.562
7.470
7.452
7.406
7.388
7.313
7.306
7.289
7.285
7.136
7.130
7.117
7.113
6.828
4.640
4.605
4.601
4.596
4.566
4.561
4.372
4.354
4.345
4.276
4.240
4.231
4.213
4.034
4.028
4.021
1.351
1.333
1.315
Ph
O
CF3
EtO
O
3ha
Ph
9
8
7
6
5
4
3
2
1
ppm
2.01
4.00
5.91
2.01
0.98
1.01
3.08
1.03
3.10

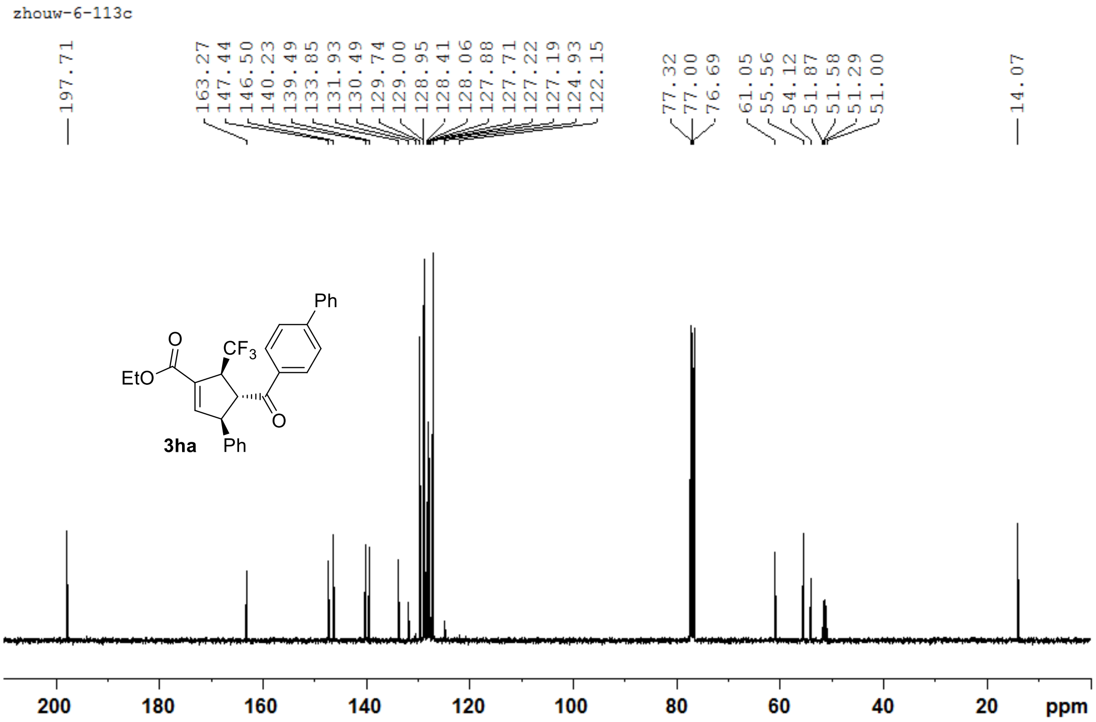
zhouw-6-113c
197.71
163.27
147.44
146.50
140.23
139.49
133.85
131.93
130.49
129.74
129.00
128.95
128.41
128.06
127.88
127.71
127.22
127.19
124.93
122.15
77.32
77.00
76.69
61.05
55.56
54.12
51.87
51.58
51.29
51.00
14.07
Ph
O
CF3
EtO
O
3ha
Ph
200
180
160
140
120
100
80
60
40
20
ppm

zhouw-6-113f

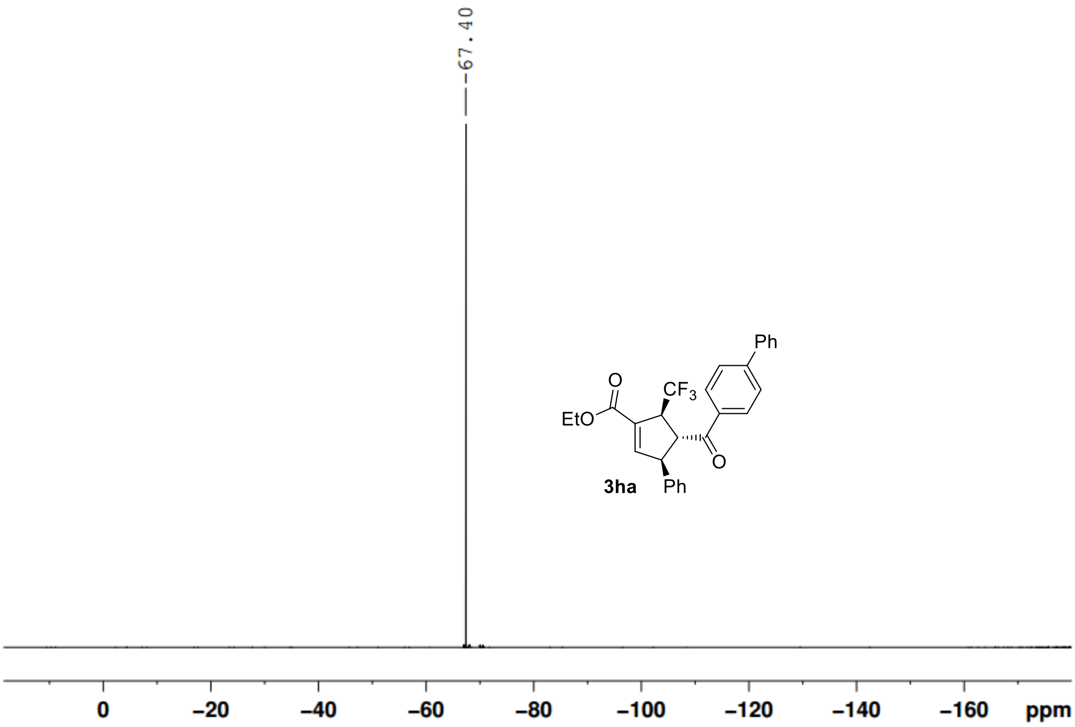

zhouw-6-114

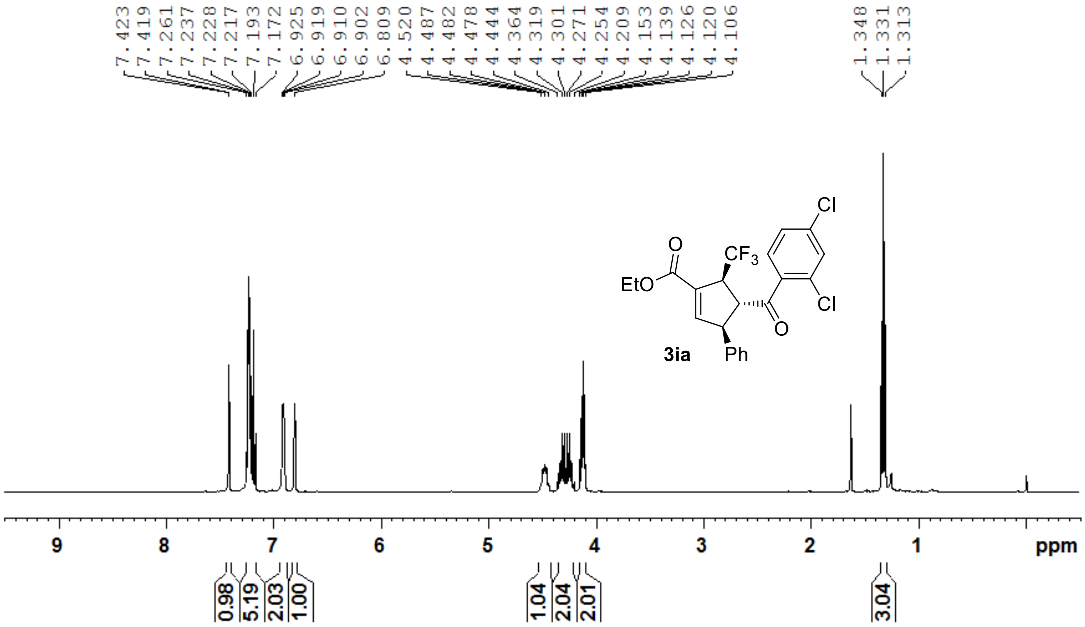

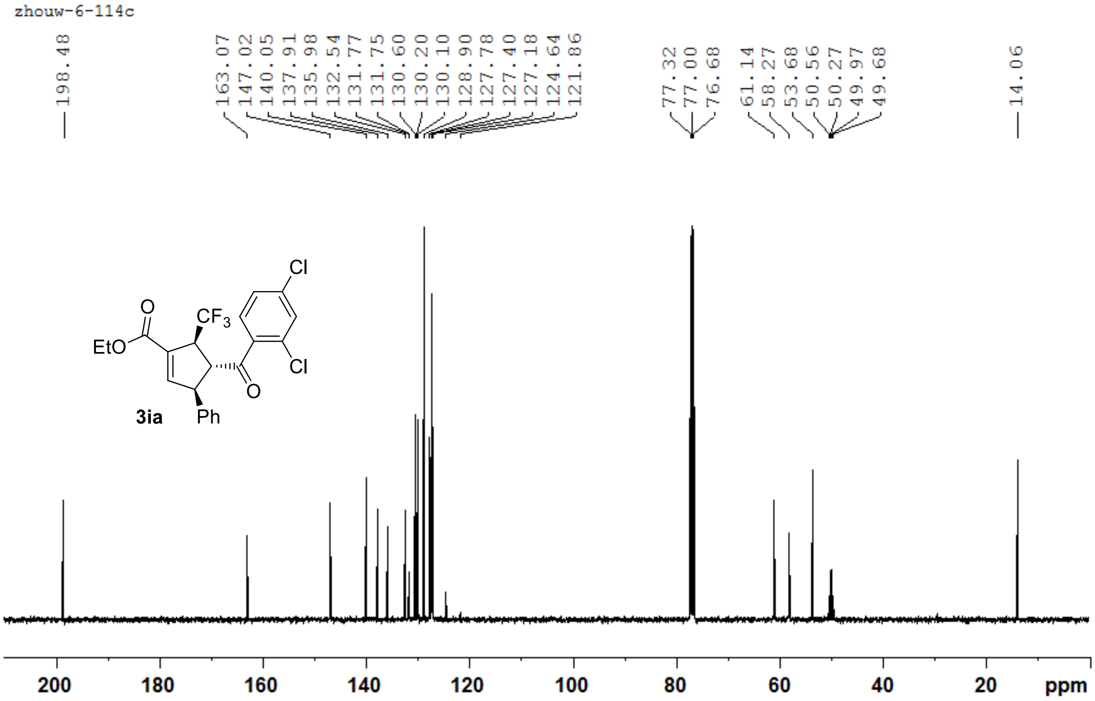
zhouw-6-114c
198.48
163.07
147.02
140.05
137.91
135.98
132.54
131.77
131.75
130.60
130.20
130.10
128.90
127.78
127.40
127.18
124.64
121.86
77.32
77.00
76.68
61.14
58.27
53.68
50.56
50.27
49.97
49.68
14.06
EtO
O
CF3
Cl
Cl
O
3ia
Ph
200
180
160
140
120
100
80
60
40
20
ppm

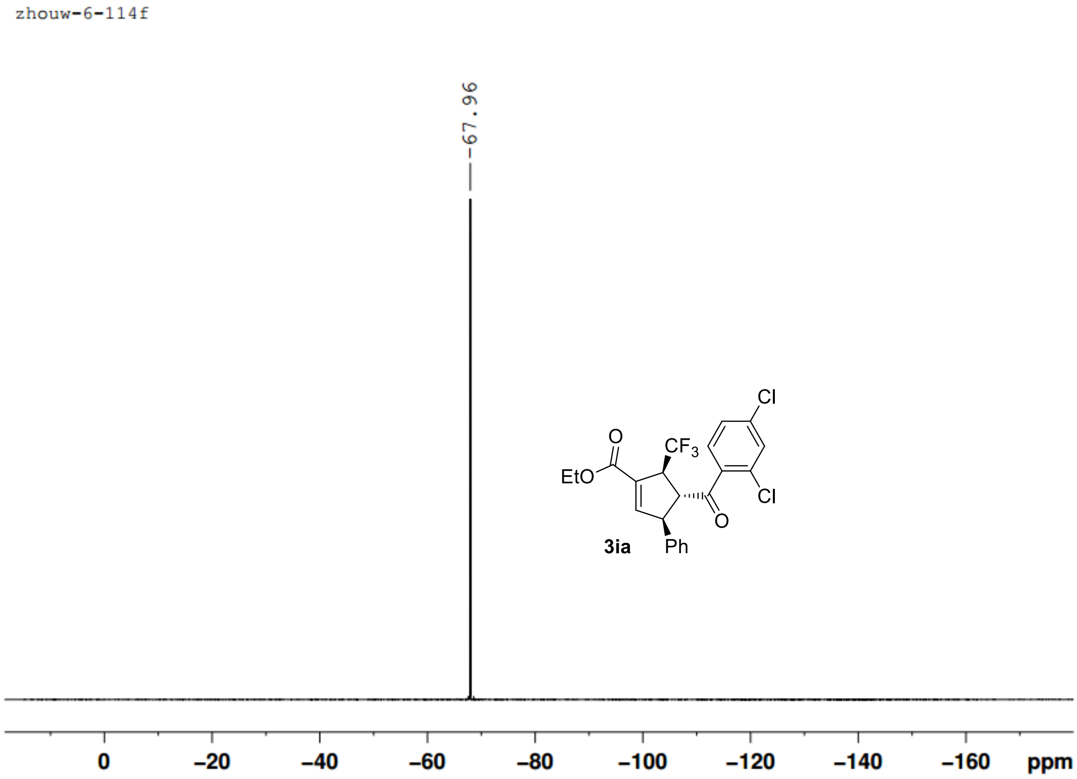
zhouw-6-114f
-67.96
EtO
O
CF3
Cl
Cl
O
3ia
Ph
0
–20
–40
–60
–80
–100
–120
–140
–160
ppm

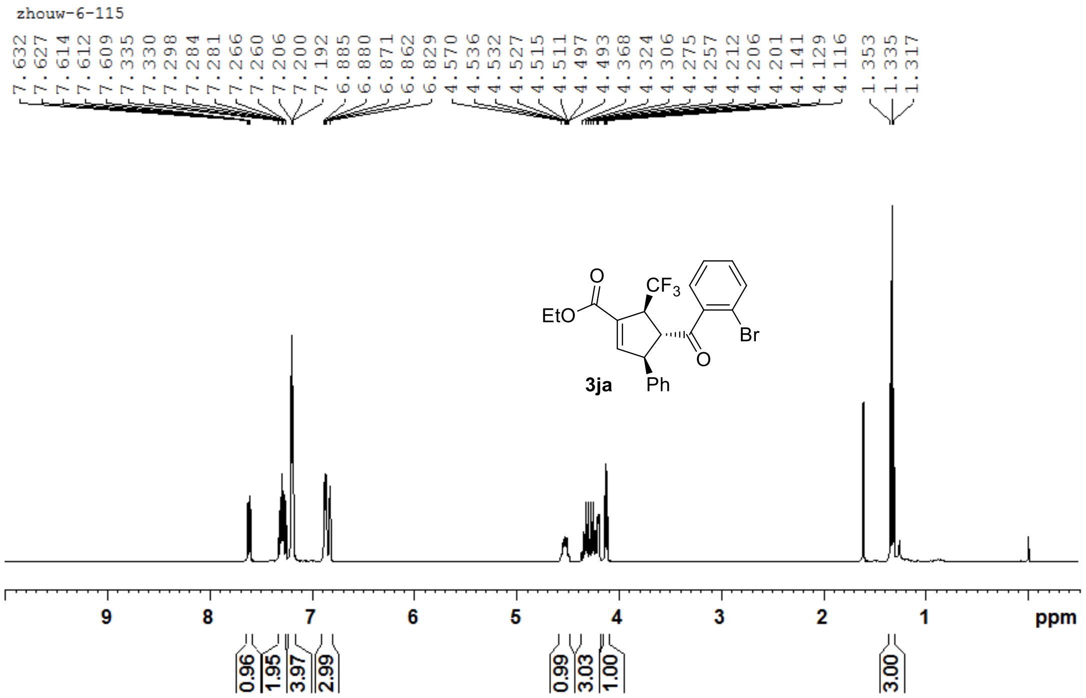
zhouw-6-115
7.632 7.627 7.614 7.612 7.609 7.335 7.330 7.298 7.284 7.281 7.266 7.260 7.206 7.200 7.192 6.885 6.880 6.871 6.862 6.829 4.570 4.536 4.532 4.527 4.515 4.511 4.497 4.493 4.368 4.324 4.306 4.275 4.257 4.212 4.206 4.201 4.141 4.129 4.116 1.353 1.335 1.317
O
CF3
EtO
Br
O
3ja
Ph
9 8 7 6 5 4 3 2 1 ppm
0.96 1.95 3.97 2.99 0.99 3.03 1.00 3.00

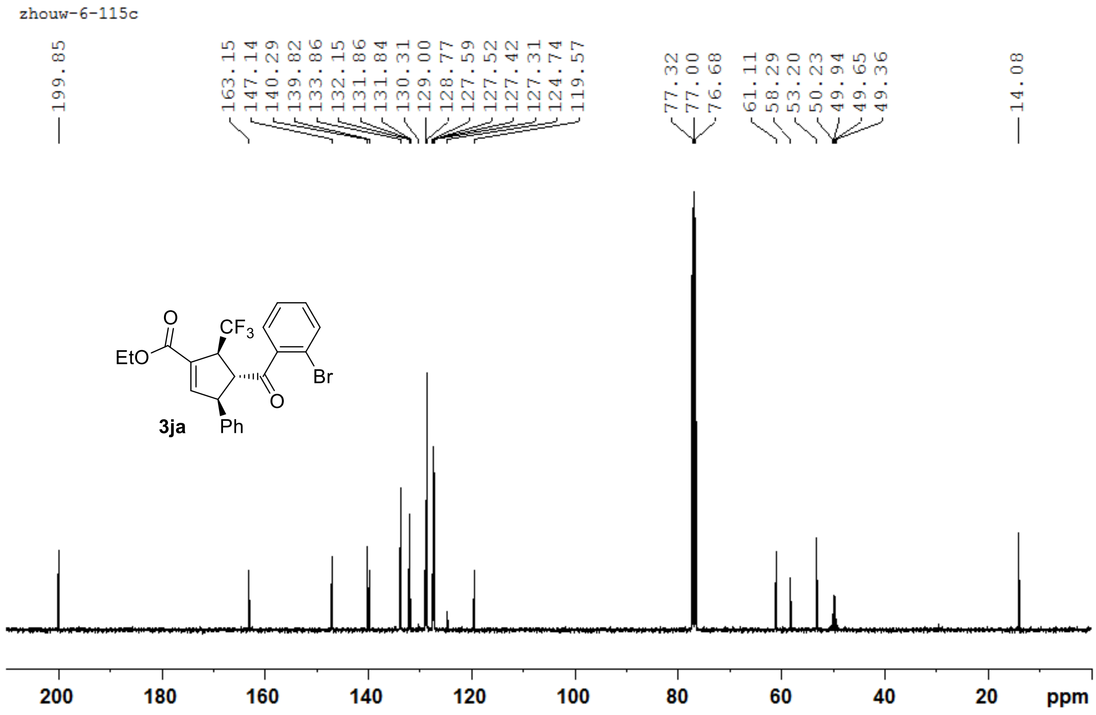
zhouw-6-115c
199.85
163.15 147.14 140.29 139.82 133.86 132.15 131.86 131.84 130.31 129.00 128.77 127.59 127.52 127.42 127.31 124.74 119.57
77.32 77.00 76.68
61.11 58.29 53.20 50.23 49.94 49.65 49.36
14.08
O
CF3
EtO
Br
O
3ja
Ph
200 180 160 140 120 100 80 60 40 20 ppm

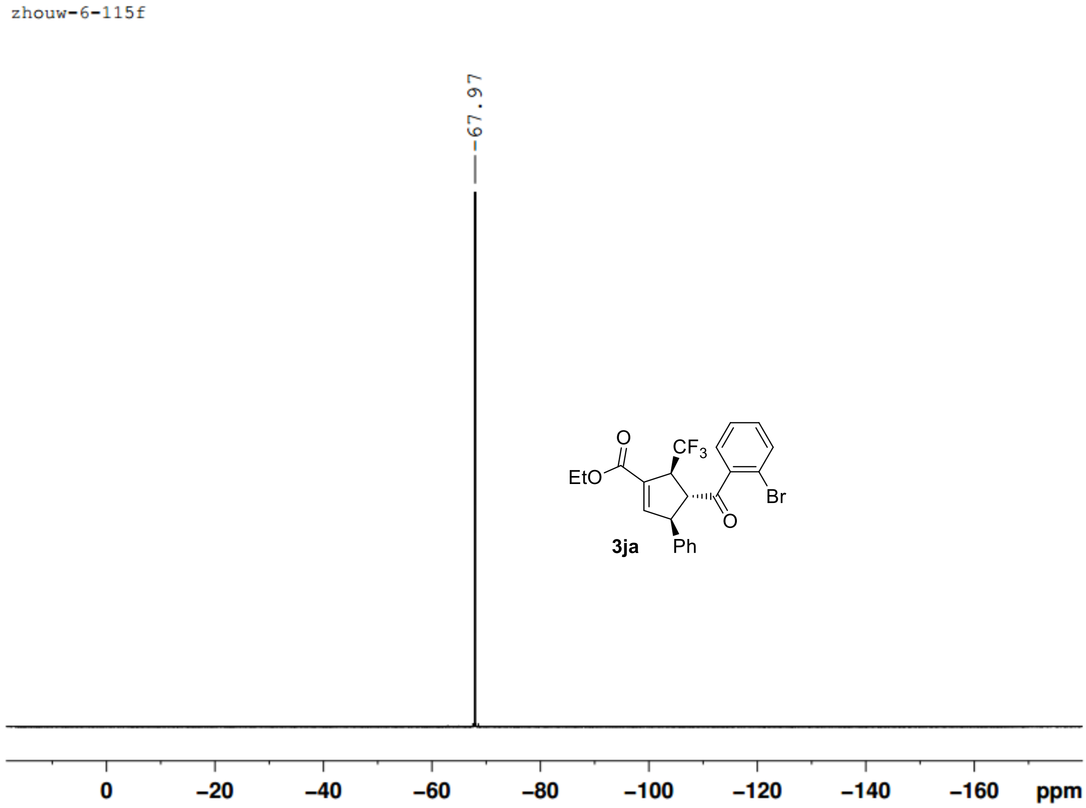
zhouw-6-115f
-67.97
EtO
O
CF3
O
Br
3ja
Ph
0
−20
−40
−60
−80
−100
−120
−140
−160
ppm

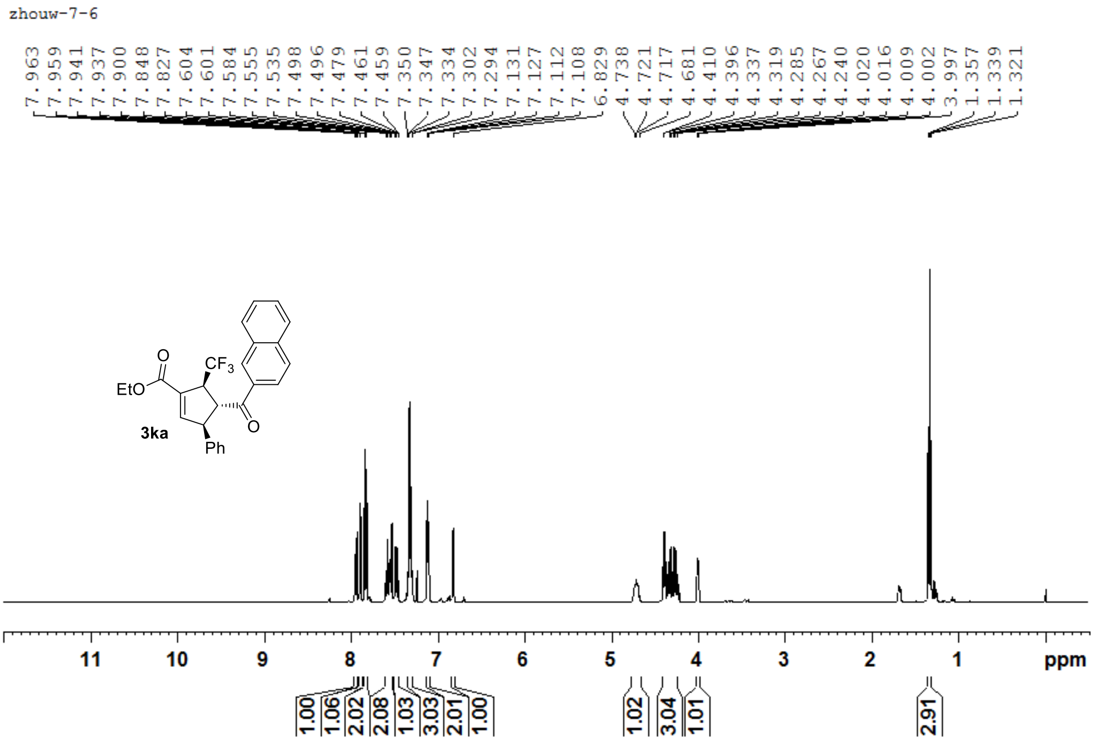
zhouw-7-6
7.963
7.959
7.941
7.937
7.900
7.848
7.827
7.604
7.601
7.584
7.555
7.535
7.498
7.496
7.479
7.461
7.459
7.350
7.347
7.334
7.302
7.294
7.131
7.127
7.112
7.108
6.829
4.738
4.721
4.717
4.681
4.410
4.396
4.337
4.319
4.285
4.267
4.240
4.240
4.020
4.016
4.009
4.002
3.997
1.357
1.339
1.321
EtO
O
CF3
O
3ka
Ph
11
10
9
8
7
6
5
4
3
2
1
ppm
1.00
1.06
2.02
2.08
1.03
3.03
2.01
1.00
1.02
3.04
1.01
2.91

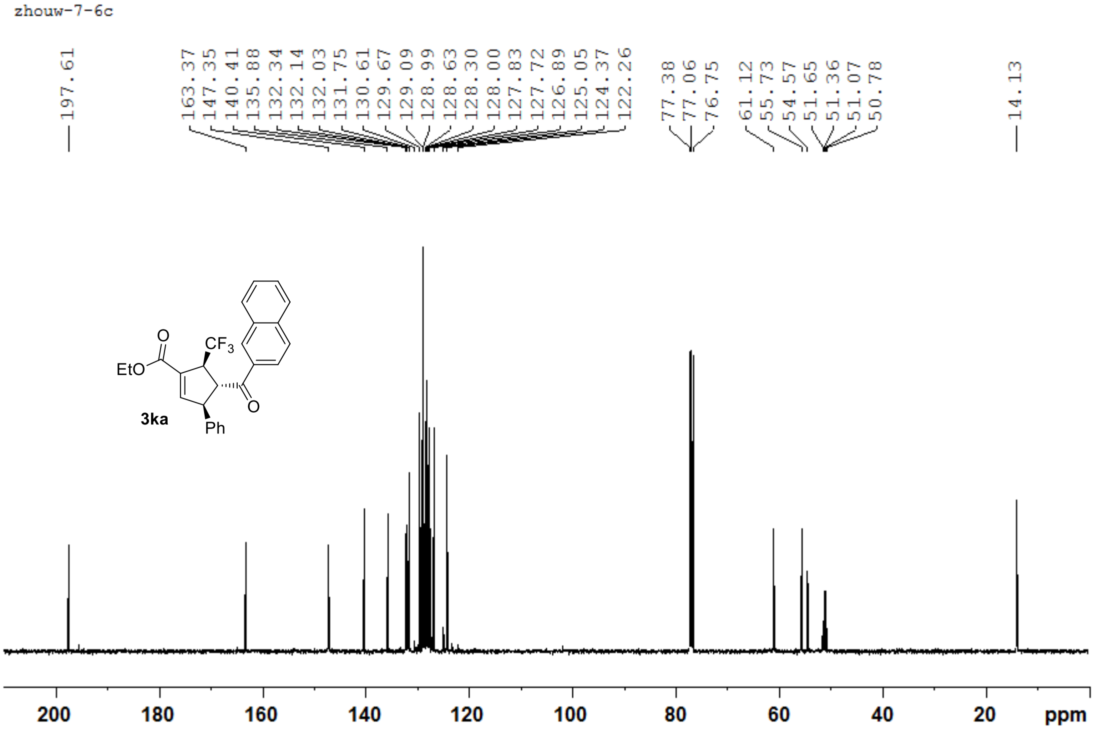
zhouw-7-6c
197.61
163.37
147.35
140.41
135.88
132.34
132.14
132.03
131.75
130.61
129.67
129.09
128.99
128.63
128.30
128.00
127.83
127.72
126.89
125.05
124.37
122.26
77.38
77.06
76.75
61.12
55.73
54.57
51.65
51.36
51.07
50.78
14.13
EtO
O
CF3
O
3ka
Ph
200 180 160 140 120 100 80 60 40 20 ppm

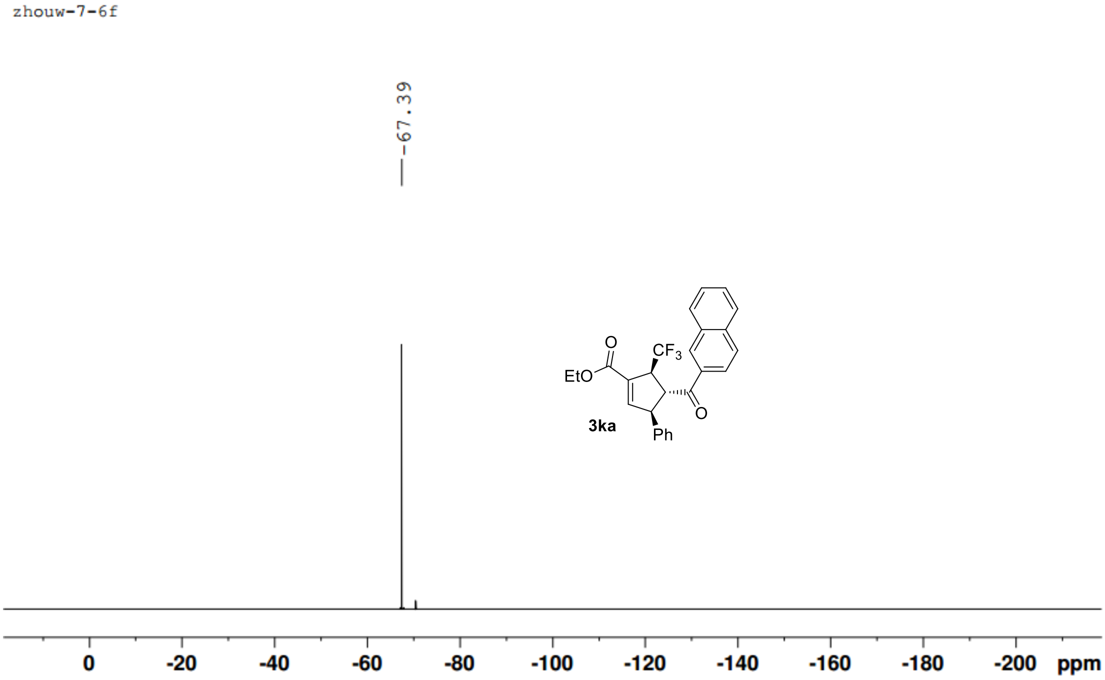
zhouw-7-6f
-67.39
EtO
O
CF3
O
3ka
Ph
0 -20 -40 -60 -80 -100 -120 -140 -160 -180 -200 ppm

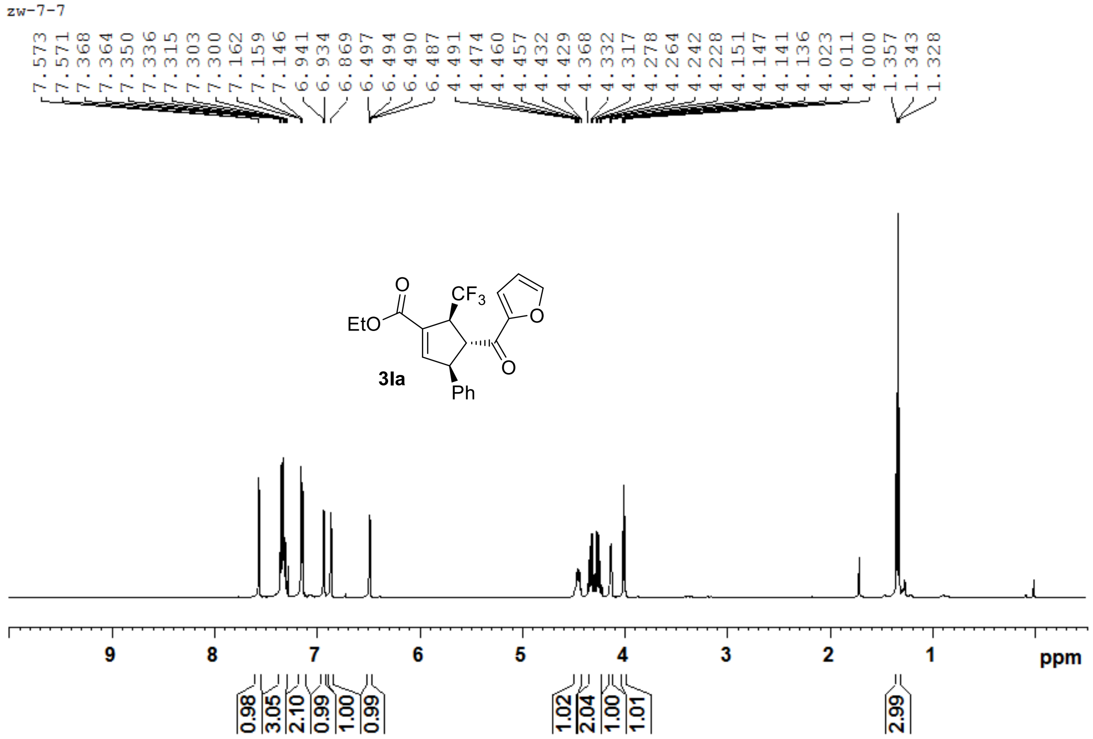
zw-7-7
7.573 7.571 7.368 7.364 7.350 7.336 7.315 7.303 7.300 7.162 7.159 7.146 6.941 6.934 6.869 6.497 6.494 6.490 6.487 4.491 4.474 4.460 4.457 4.432 4.429 4.368 4.332 4.317 4.278 4.264 4.242 4.228 4.151 4.147 4.141 4.136 4.023 4.011 4.000 1.357 1.343 1.328
EtO
O
CF3
O
O
3la
Ph
9 8 7 6 5 4 3 2 1 ppm
0.98 3.05 2.10 0.99 1.00 0.99
1.02 2.04 1.00 1.01
2.99

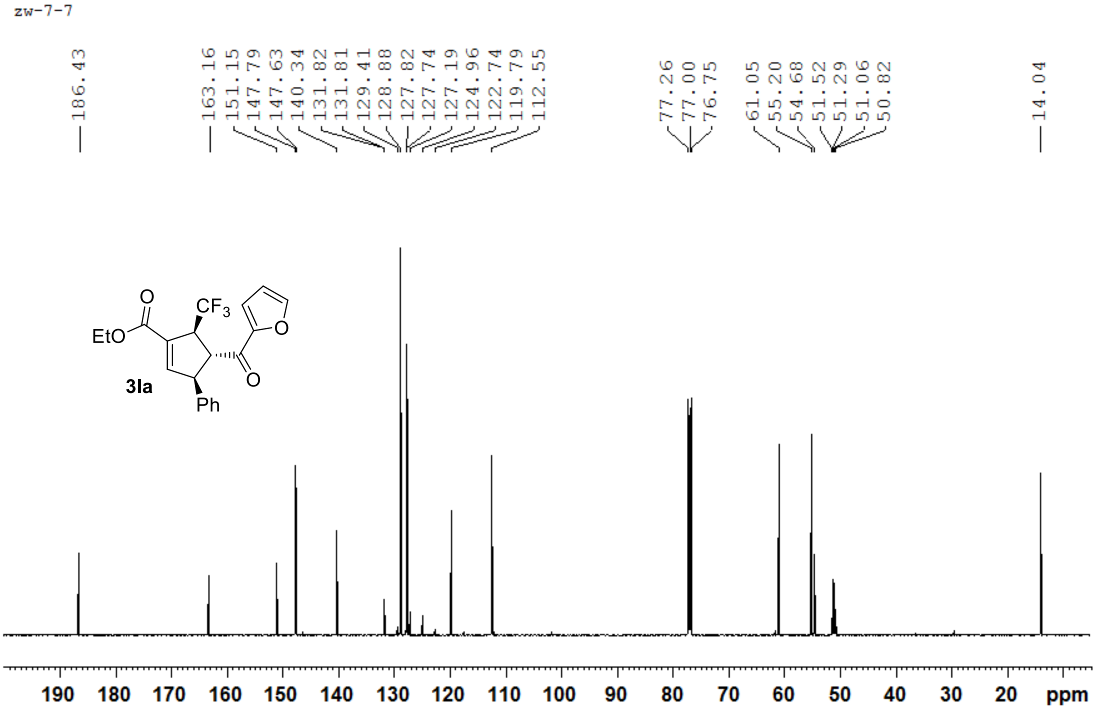
zw-7-7
186.43
163.16 151.15 147.79 147.63 140.34 131.82 131.81 129.41 128.88 127.82 127.74 127.19 124.96 122.74 119.79 112.55
77.26 77.00 76.75
61.05 55.20 54.68 51.52 51.29 51.06 50.82
14.04
EtO
O
CF3
O
O
3la
Ph
190 180 170 160 150 140 130 120 110 100 90 80 70 60 50 40 30 20 ppm

zhouw-7-7f

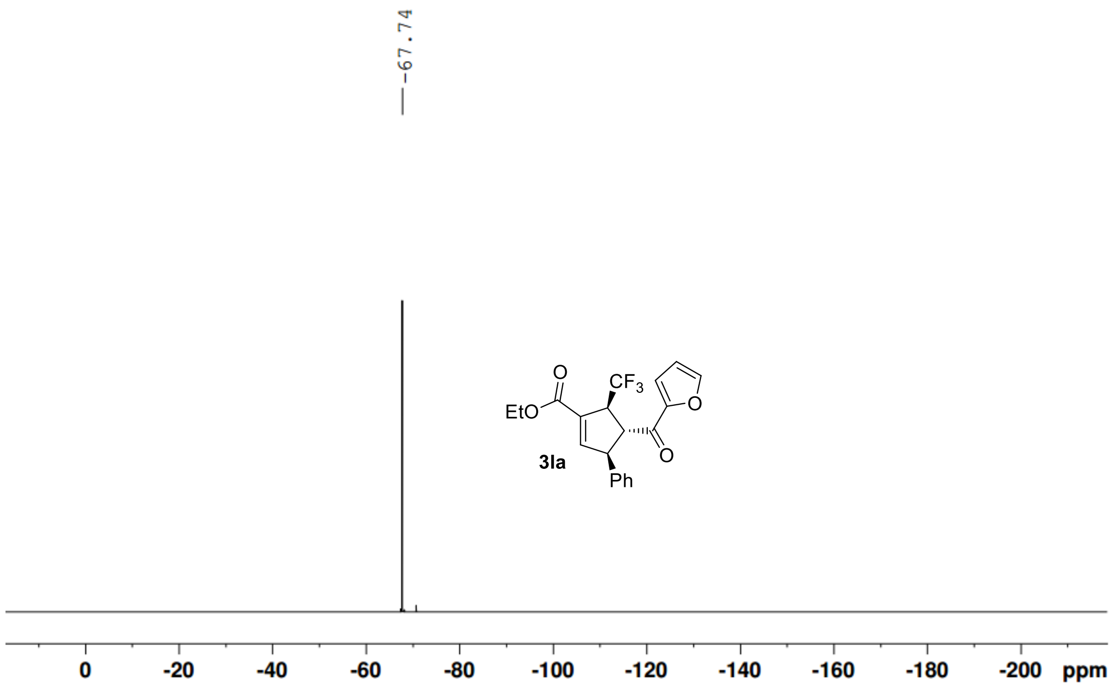

zw-7-8

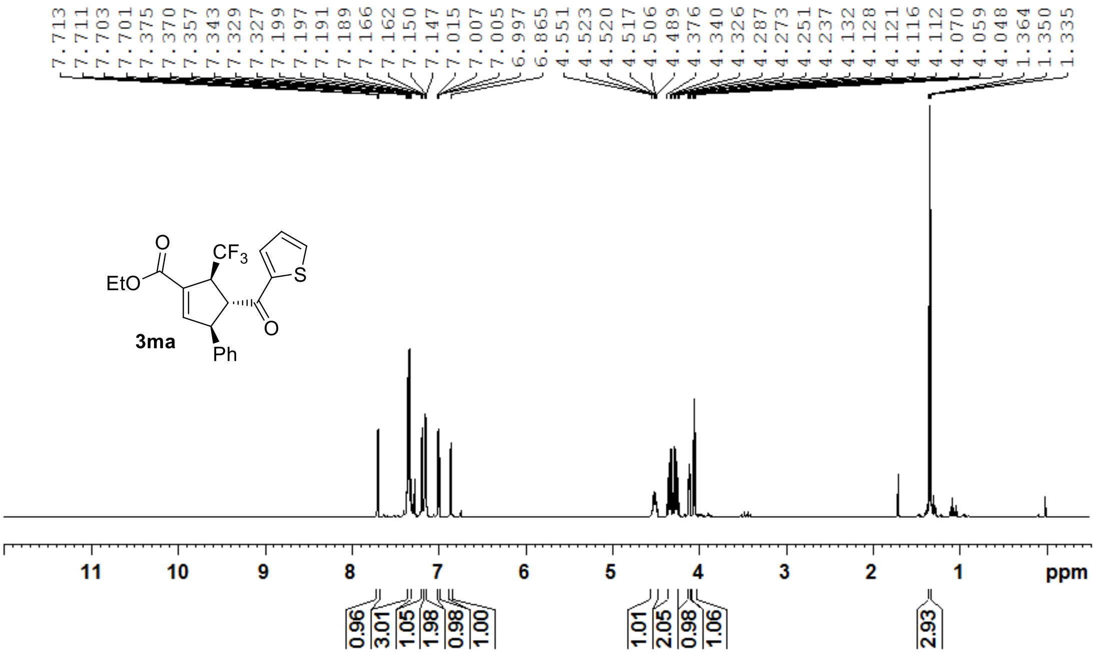

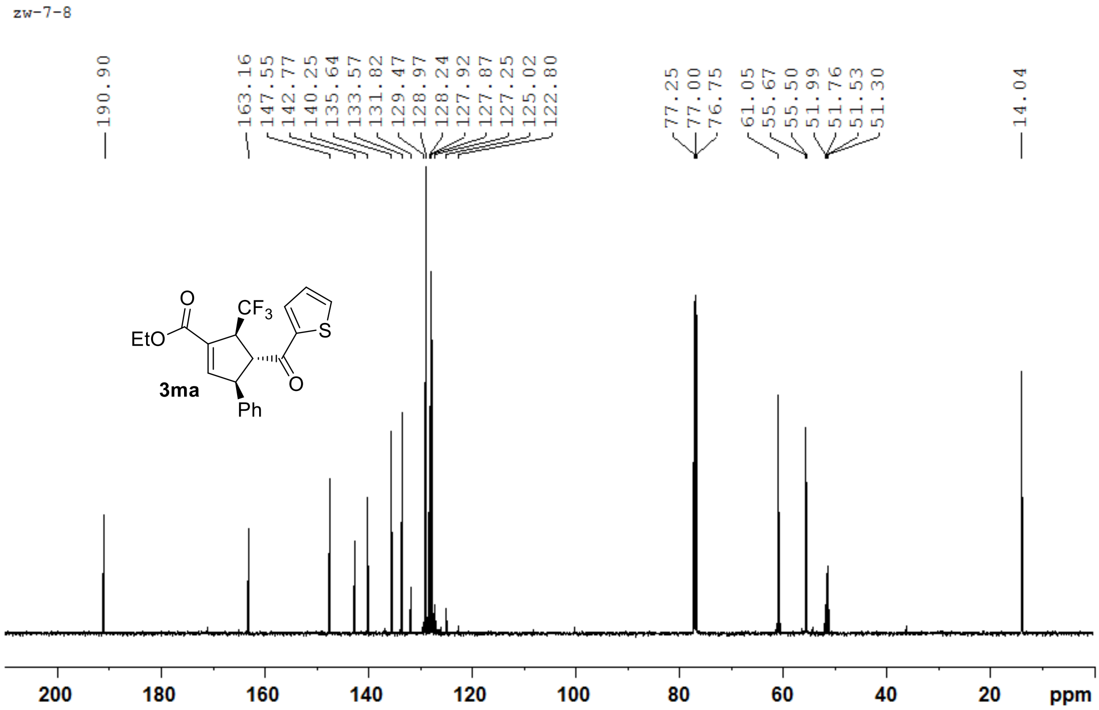
zw-7-8
190.90
163.16
147.55
142.77
140.25
135.64
133.57
131.82
129.47
128.97
128.24
127.92
127.87
127.25
125.02
122.80
77.25
77.00
76.75
61.05
55.67
55.50
51.99
51.76
51.53
51.30
14.04
CF3
EtO
O
S
3ma
Ph
200
180
160
140
120
100
80
60
40
20
ppm

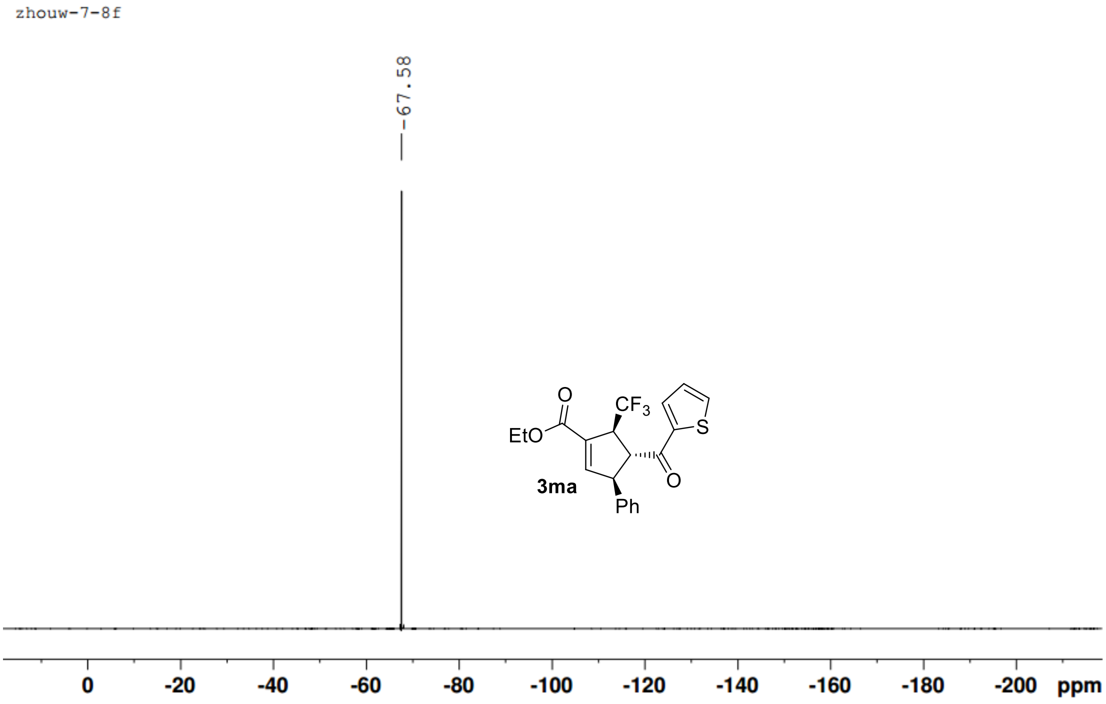
zhouw-7-8f
-67.58
CF3
EtO
O
S
3ma
Ph
0
-20
-40
-60
-80
-100
-120
-140
-160
-180
-200
ppm

zhouw-7-9

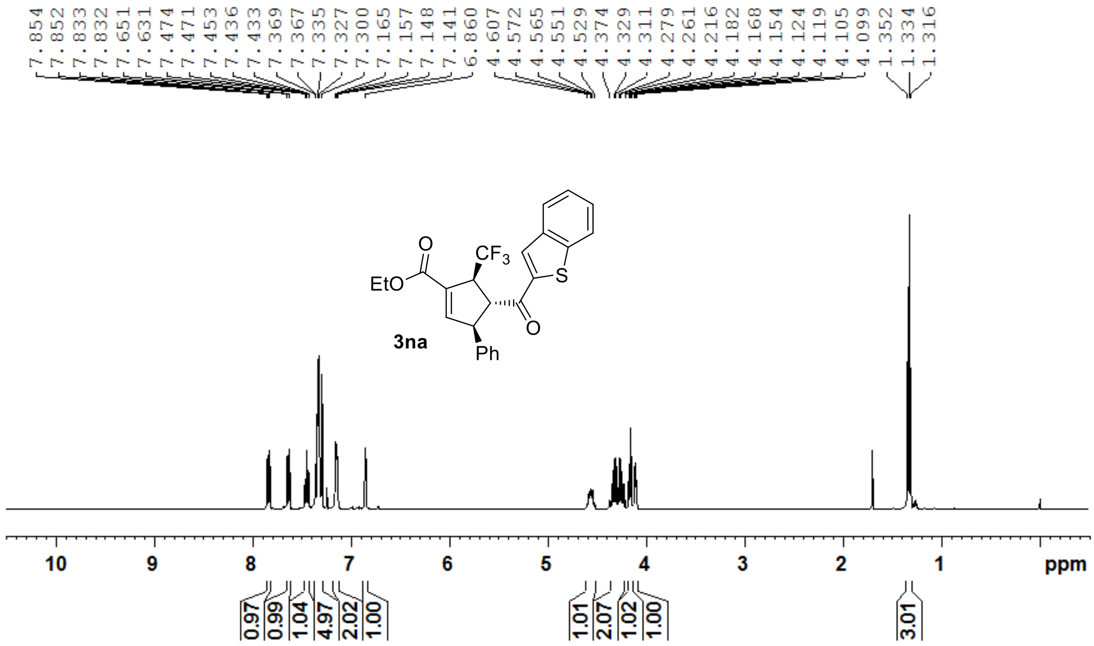

zhouw-7-9c

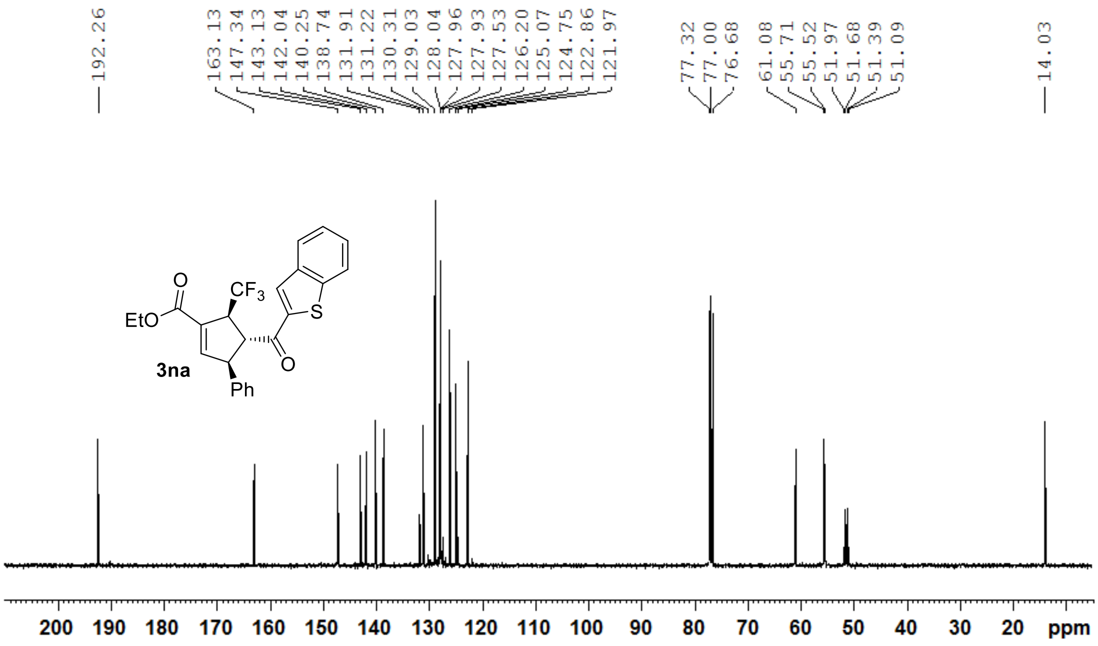

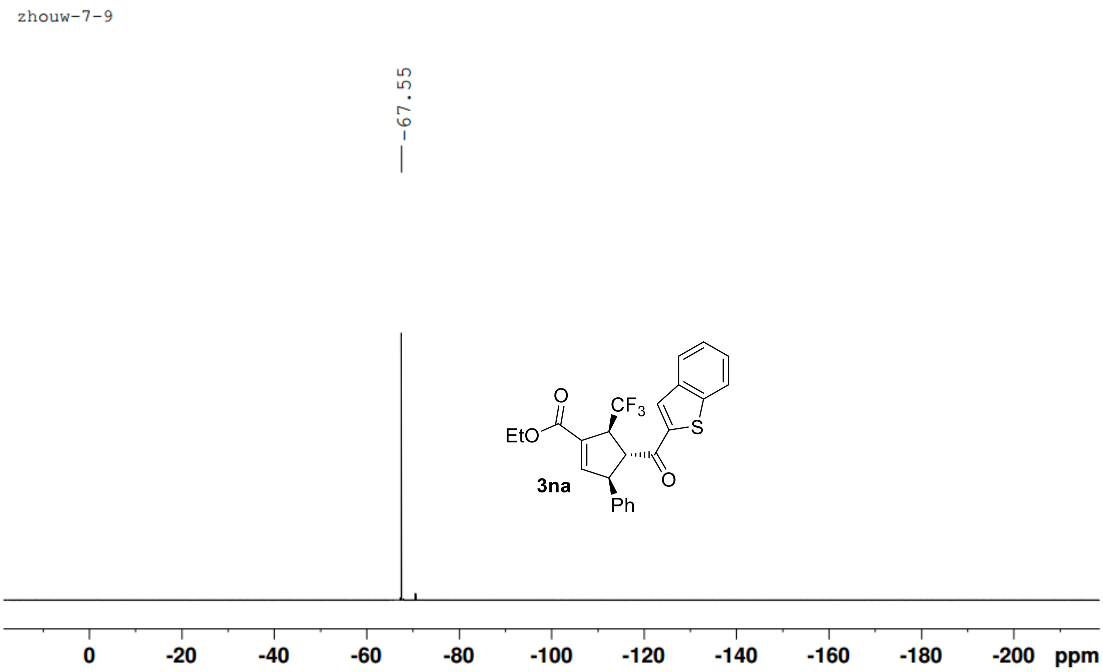
zhouw-7-9
-67.55
3na
EtO
O
CF3
S
Ph
0 -20 -40 -60 -80 -100 -120 -140 -160 -180 -200 ppm

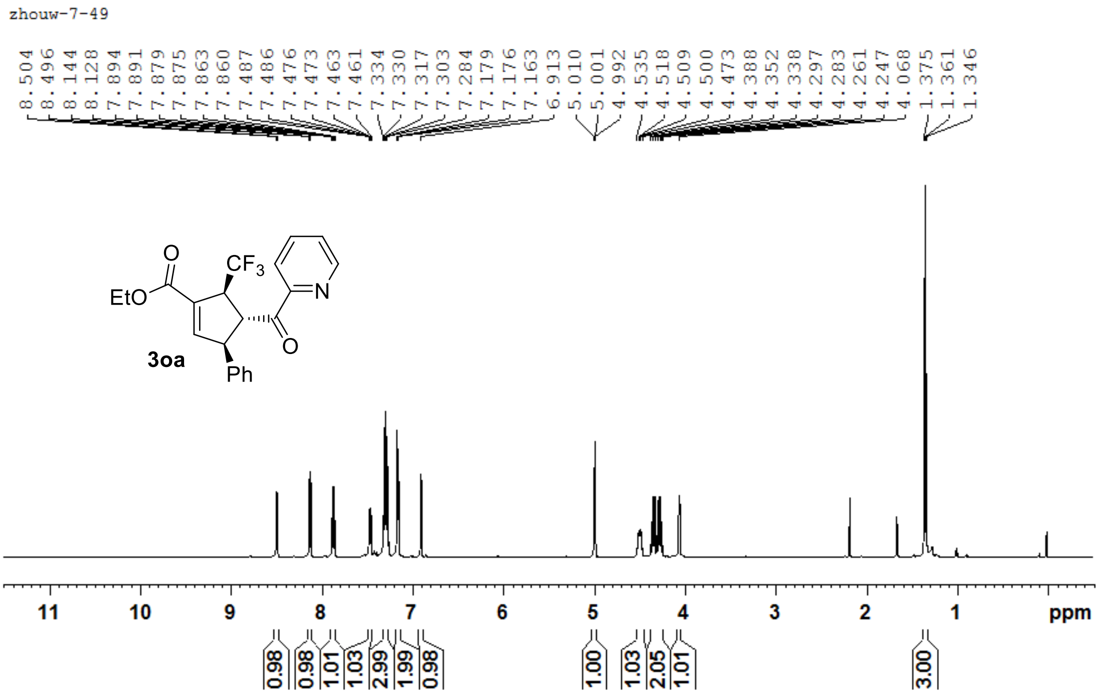
zhouw-7-49
8.504 8.496 8.144 8.128 7.894 7.891 7.879 7.875 7.863 7.860 7.487 7.486 7.476 7.473 7.463 7.461 7.334 7.330 7.317 7.303 7.284 7.179 7.176 7.163 6.913 5.010 5.001 4.992 4.535 4.518 4.509 4.500 4.473 4.388 4.352 4.338 4.297 4.283 4.261 4.247 4.068 1.375 1.361 1.346
3oa
EtO
O
CF3
N
Ph
11 10 9 8 7 6 5 4 3 2 1 ppm
0.98 0.98 1.01 1.03 2.99 1.99 0.98 1.00 1.03 2.05 1.01 3.00

zhouw-7-49c

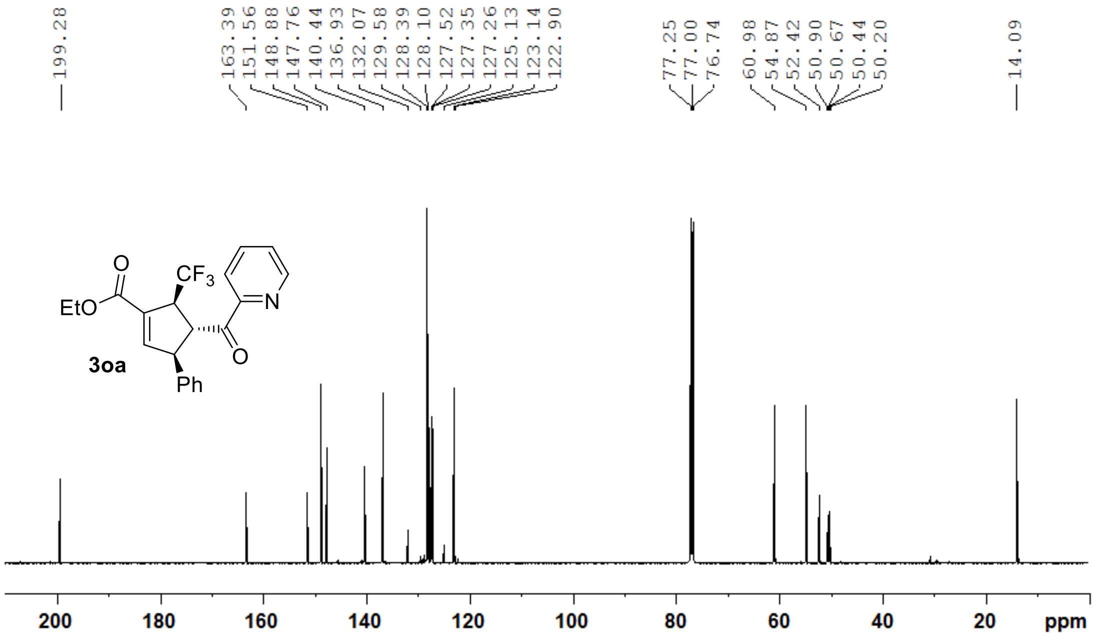

zhouw-7-49f

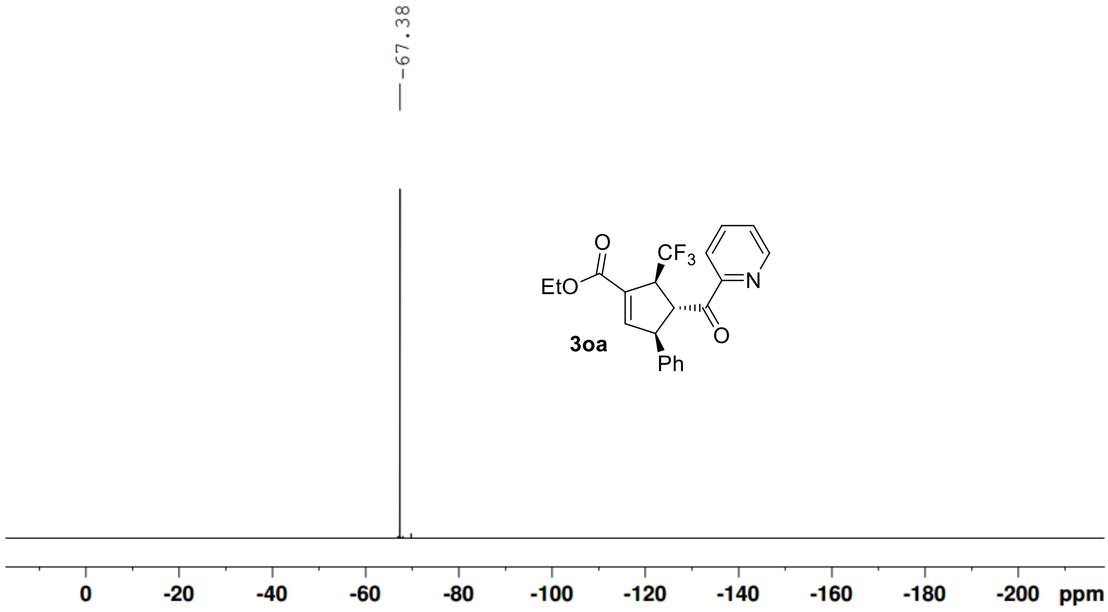

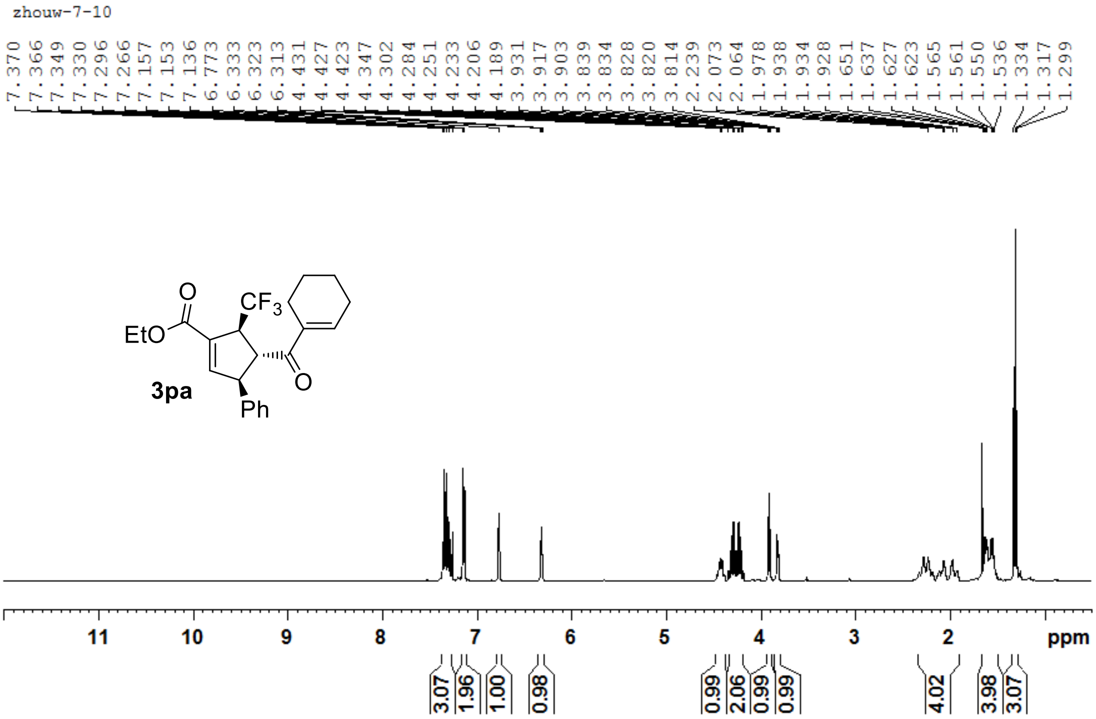
zhouw-7-10
7.370 7.366 7.349 7.330 7.296 7.266 7.157 7.153 7.136 6.773 6.333 6.323 6.313 4.431 4.427 4.423 4.347 4.302 4.284 4.251 4.233 4.206 4.189 3.931 3.917 3.903 3.839 3.834 3.828 3.820 3.814 2.239 2.073 2.064 1.978 1.938 1.934 1.928 1.651 1.637 1.627 1.623 1.565 1.561 1.550 1.536 1.334 1.317 1.299
EtO
O
CF3
O
3pa
Ph
11 10 9 8 7 6 5 4 3 2 ppm
3.07 1.96 1.00 0.98 0.99 2.06 0.99 0.99 4.02 3.98 3.07

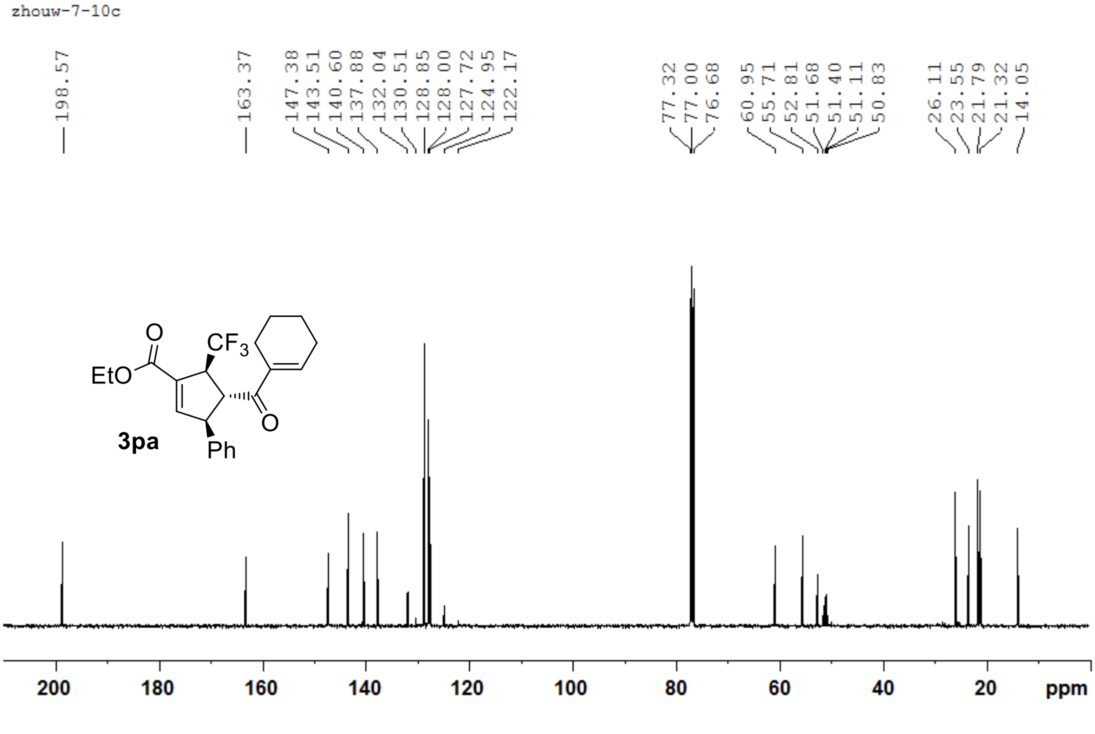
zhouw-7-10c
198.57 163.37 147.38 143.51 140.60 137.88 132.04 130.51 128.85 128.00 127.72 124.95 122.17 77.32 77.00 76.68 60.95 55.71 52.81 51.68 51.40 51.11 50.83 26.11 23.55 21.79 21.32 14.05
EtO
O
CF3
O
3pa
Ph
200 180 160 140 120 100 80 60 40 20 ppm

zhouw-7-10f

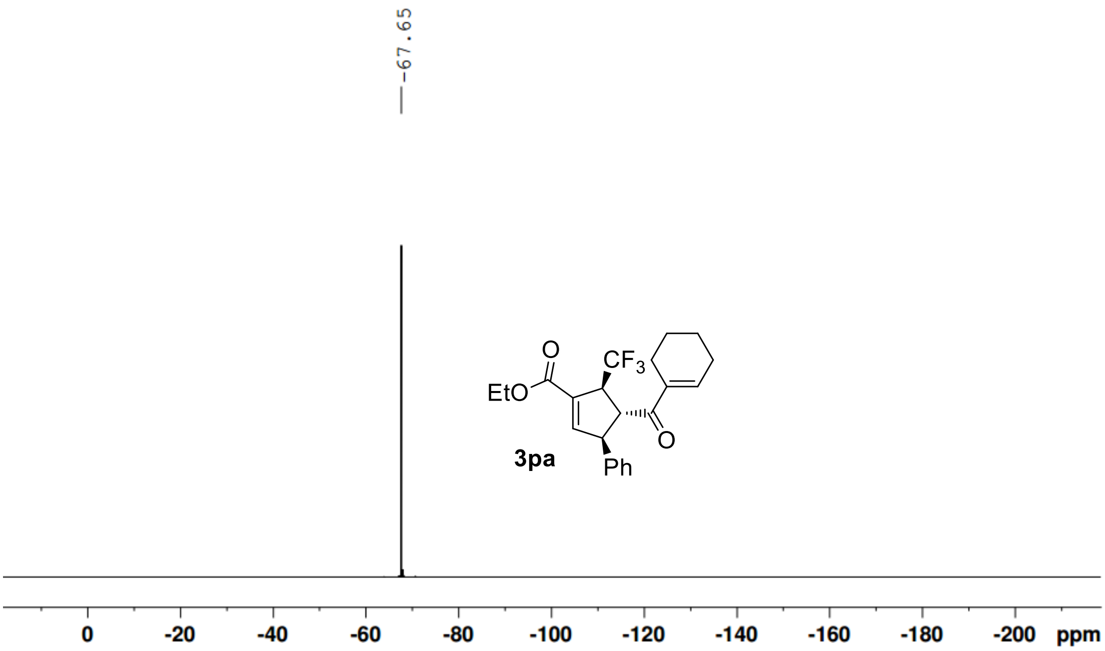

3pa

zhouw-7-54

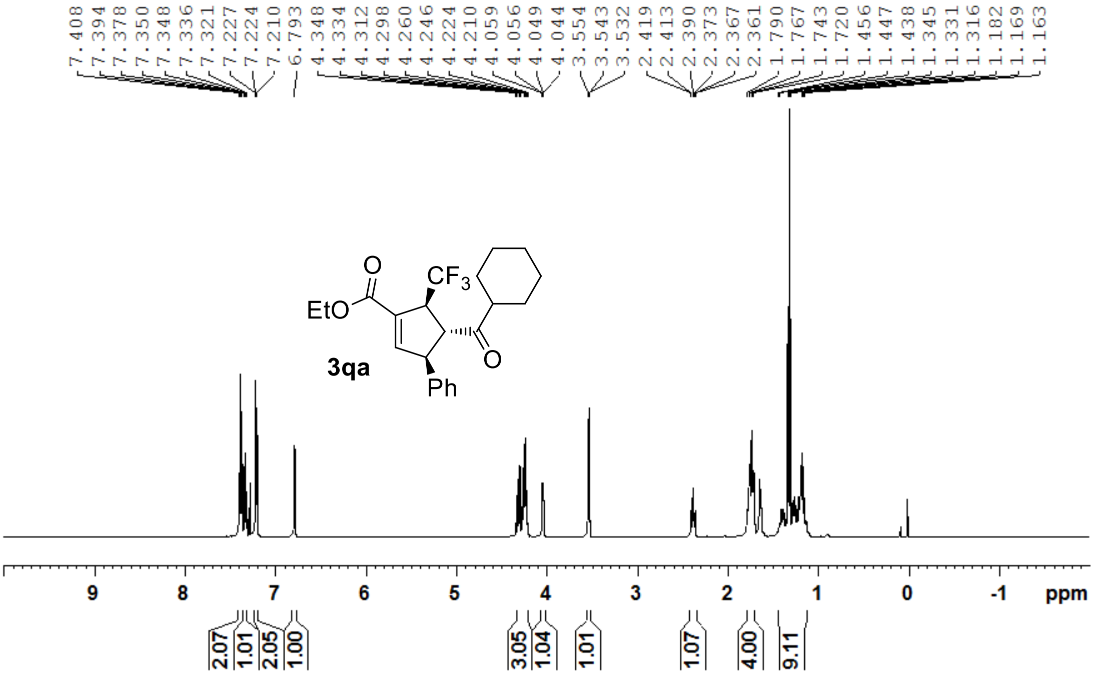

3qa

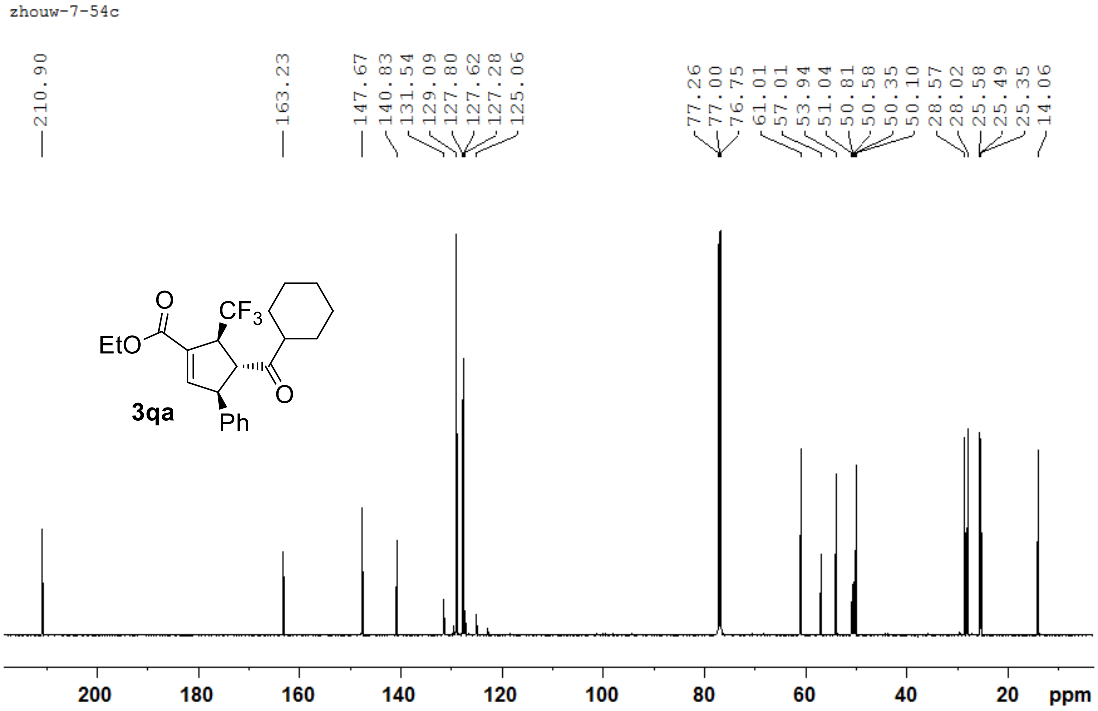
zhouw-7-54c
210.90
163.23
147.67
140.83
131.54
129.09
127.80
127.62
127.28
125.06
77.26
77.00
76.75
61.01
57.01
53.94
51.04
50.81
50.58
50.35
50.10
28.57
28.02
25.58
25.49
25.35
14.06
EtO
O
CF3
O
3qa
Ph
200
180
160
140
120
100
80
60
40
20
ppm

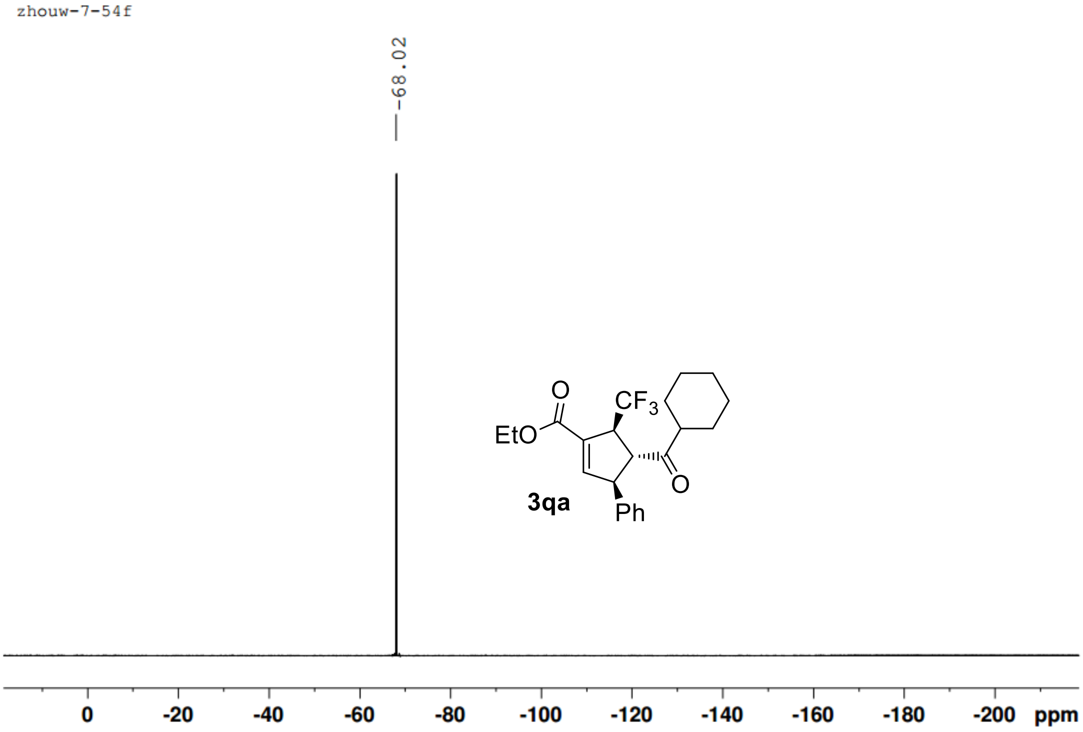
zhouw-7-54f
-68.02
EtO
O
CF3
O
3qa
Ph
0
-20
-40
-60
-80
-100
-120
-140
-160
-180
-200
ppm

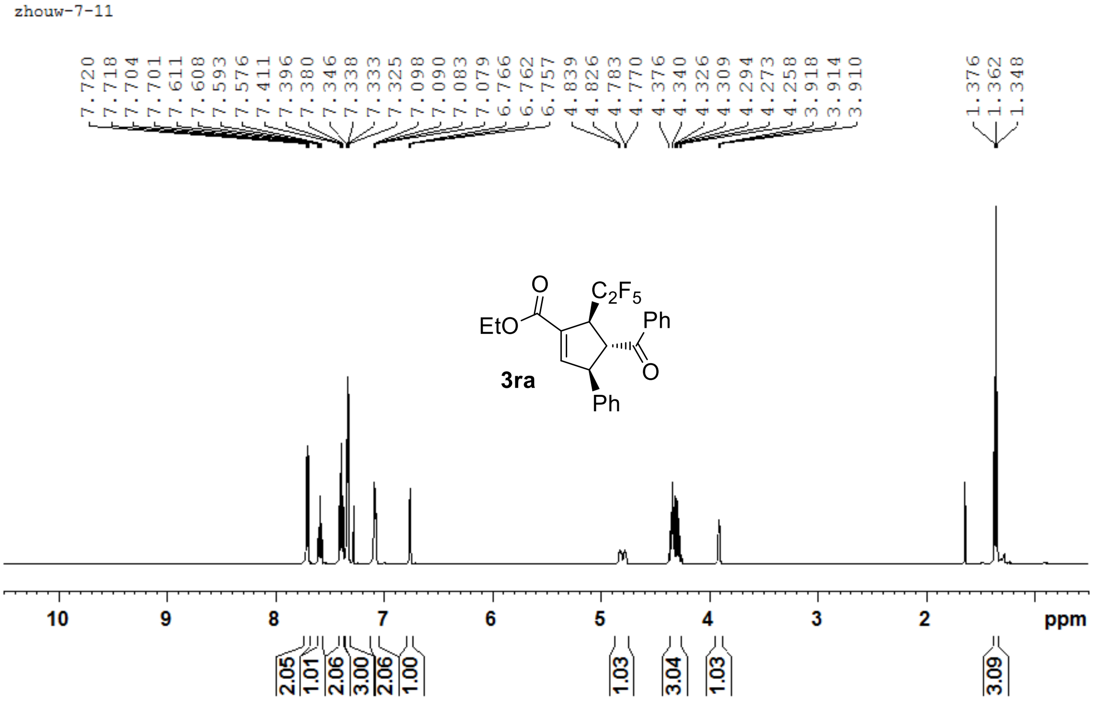
zhouw-7-11
7.720
7.718
7.704
7.701
7.611
7.608
7.593
7.576
7.411
7.396
7.380
7.346
7.338
7.333
7.325
7.098
7.090
7.083
7.079
6.766
6.762
6.757
4.839
4.826
4.783
4.770
4.376
4.340
4.326
4.309
4.294
4.273
4.258
3.918
3.914
3.910
1.376
1.362
1.348
EtO
O
C2F5
Ph
O
3ra
Ph
10
9
8
7
6
5
4
3
2
ppm
2.05
1.01
2.06
3.00
2.06
1.00
1.03
3.04
1.03
3.09

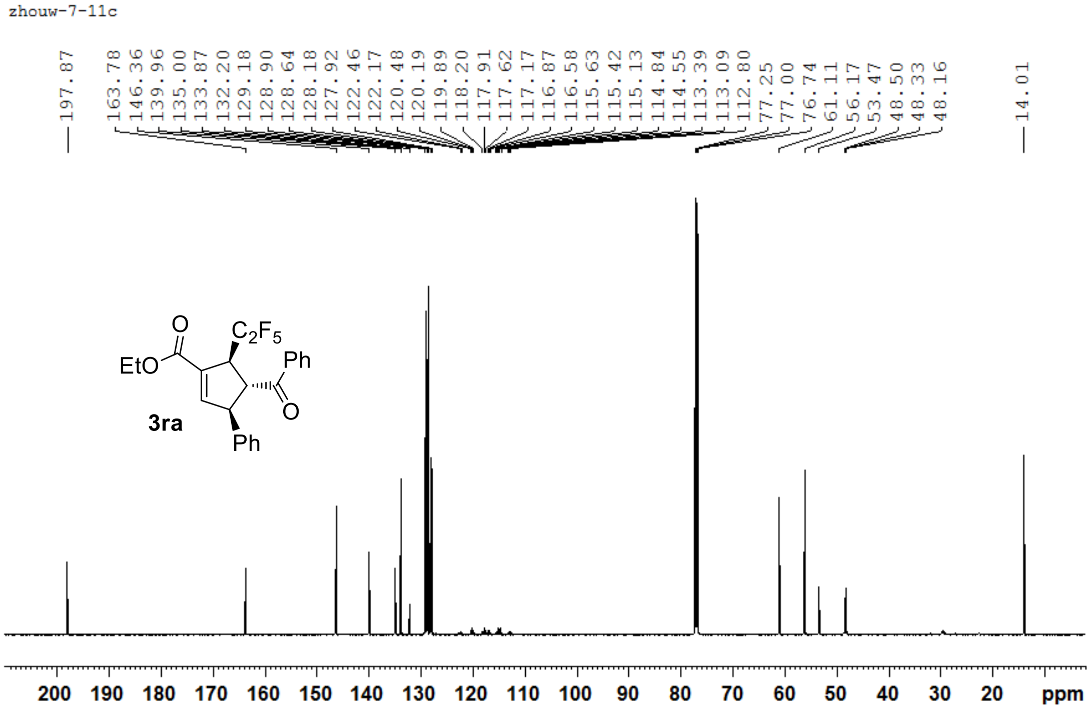
zhouw-7-11c
197.87
163.78
146.36
139.96
135.00
133.87
132.20
129.18
128.90
128.64
128.18
127.92
122.46
122.17
120.48
120.19
119.89
118.20
117.91
117.62
117.17
116.87
116.58
115.63
115.42
115.13
114.84
114.55
113.39
113.09
112.80
77.25
77.00
76.74
61.11
56.17
53.47
48.50
48.33
48.16
14.01
EtO
O
C2F5
Ph
O
3ra
Ph
200
190
180
170
160
150
140
130
120
110
100
90
80
70
60
50
40
30
20
ppm

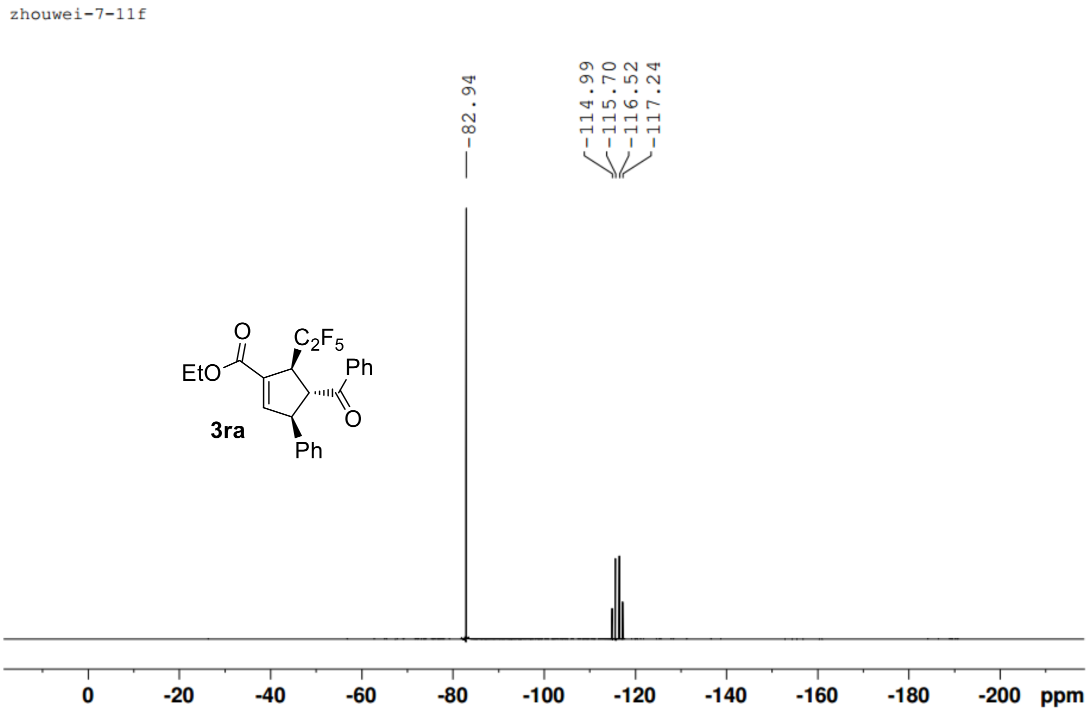
zhouwei-7-11f
-82.94
-114.99
-115.70
-116.52
-117.24
EtO
O
C_2F_5
Ph
O
3ra
Ph
0
-20
-40
-60
-80
-100
-120
-140
-160
-180
-200
ppm

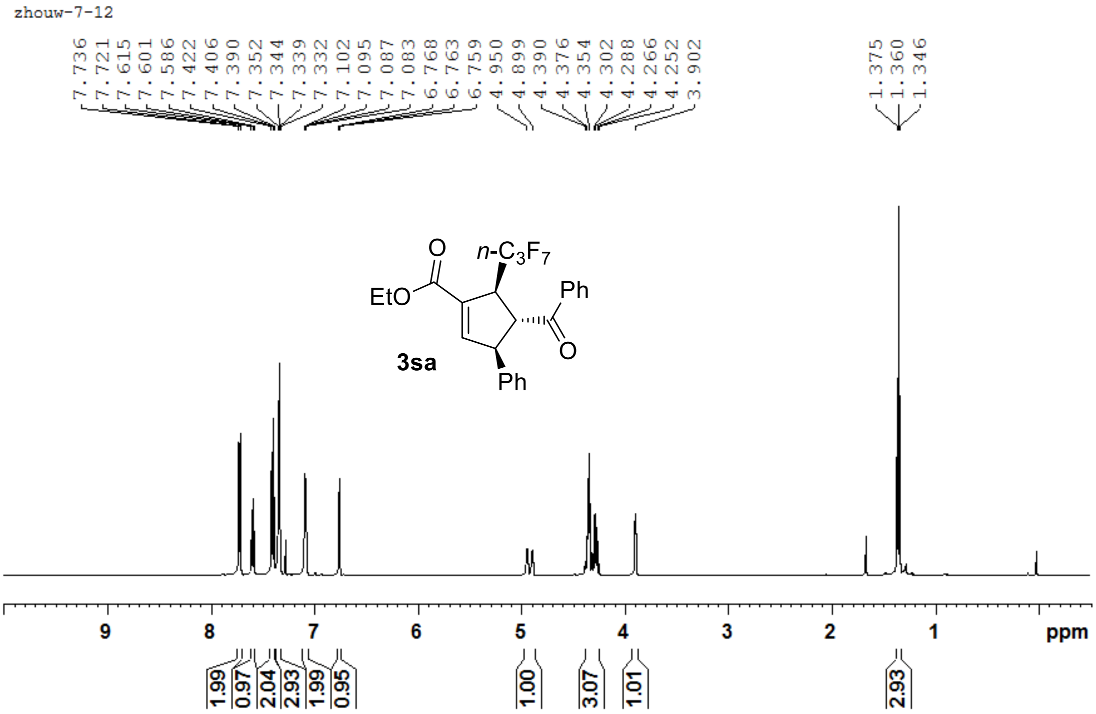
zhouw-7-12
7.736
7.721
7.615
7.601
7.586
7.422
7.406
7.390
7.352
7.344
7.339
7.332
7.102
7.095
7.087
7.083
6.768
6.763
6.759
4.950
4.899
4.390
4.376
4.354
4.302
4.288
4.266
4.252
3.902
1.375
1.360
1.346
EtO
O
n-C_3F_7
Ph
O
3sa
Ph
9
8
7
6
5
4
3
2
1
ppm
1.99
0.97
2.04
2.93
1.99
0.95
1.00
3.07
1.01
2.93

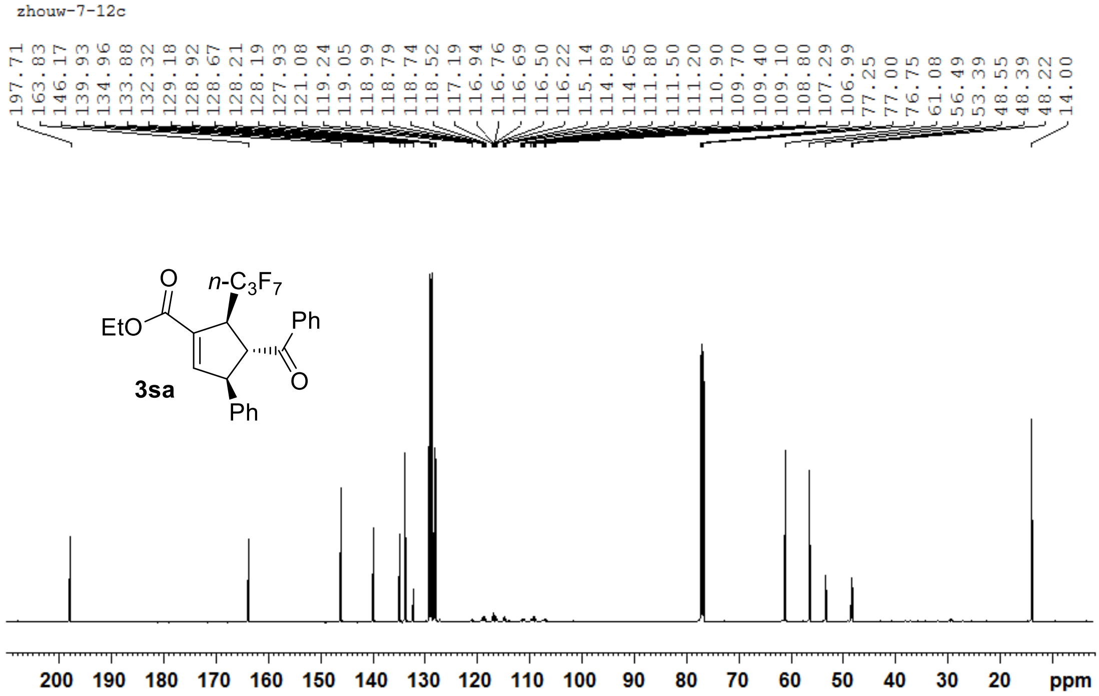
zhouw-7-12c
197.71
163.83
146.17
139.93
134.96
133.88
132.32
129.18
128.92
128.67
128.21
128.19
127.93
121.08
119.24
119.05
118.99
118.79
118.74
118.52
117.19
116.94
116.76
116.69
116.50
116.22
115.14
114.89
114.65
111.80
111.50
111.20
110.90
109.70
109.40
109.10
108.80
107.29
106.99
77.25
77.00
76.75
61.08
56.49
53.39
48.55
48.39
48.22
14.00
O
n-C3F7
EtO
Ph
3sa
O
Ph
200 190 180 170 160 150 140 130 120 110 100 90 80 70 60 50 40 30 20 ppm

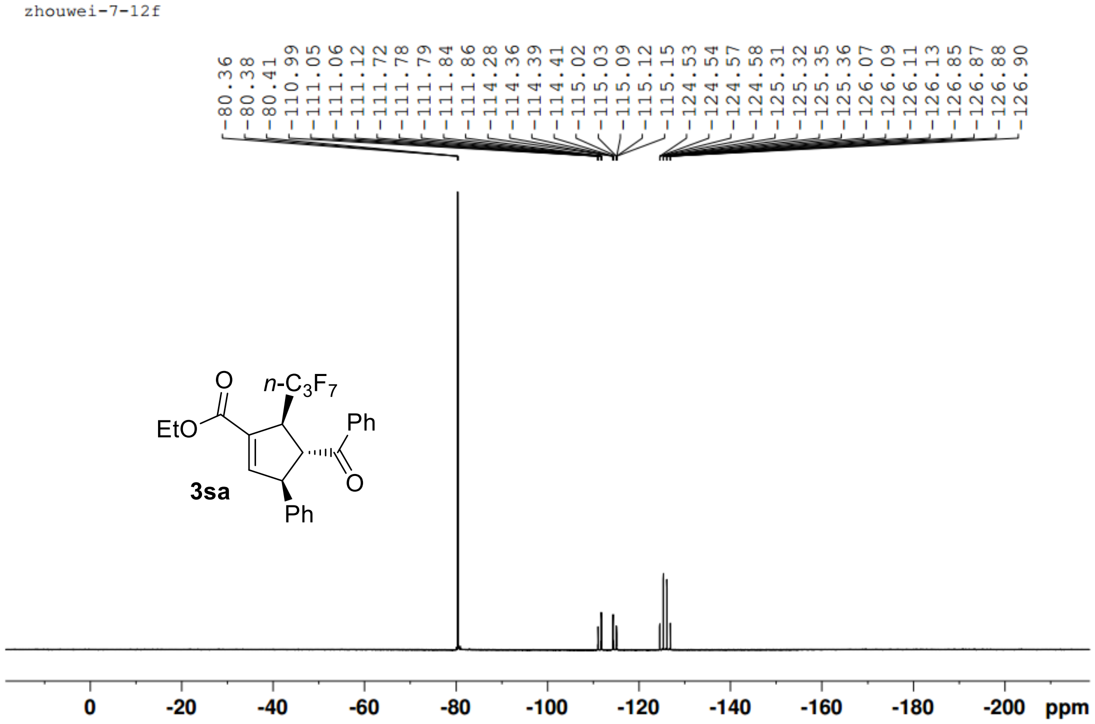
zhouwei-7-12f
-80.36
-80.38
-80.41
-110.99
-111.05
-111.06
-111.12
-111.72
-111.78
-111.79
-111.84
-111.86
-114.28
-114.36
-114.39
-114.41
-115.02
-115.03
-115.09
-115.12
-115.15
-124.53
-124.54
-124.57
-124.58
-125.31
-125.32
-125.35
-125.36
-126.07
-126.09
-126.11
-126.13
-126.85
-126.87
-126.88
-126.90
O
n-C3F7
EtO
Ph
3sa
O
Ph
0 -20 -40 -60 -80 -100 -120 -140 -160 -180 -200 ppm

zhouw-7-71

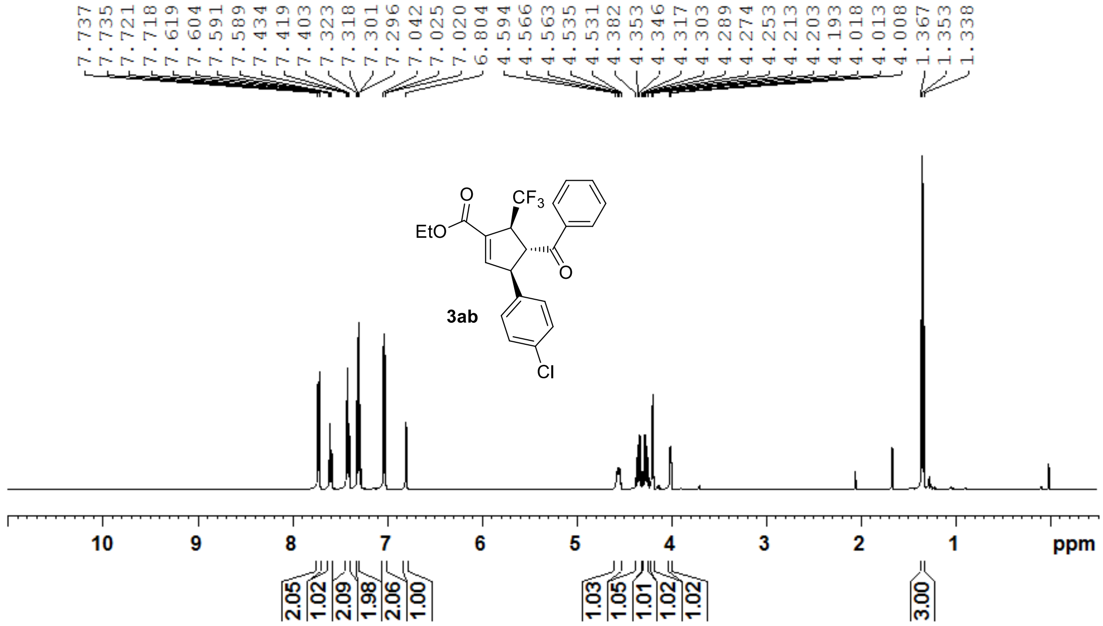

zhouw-7-71c

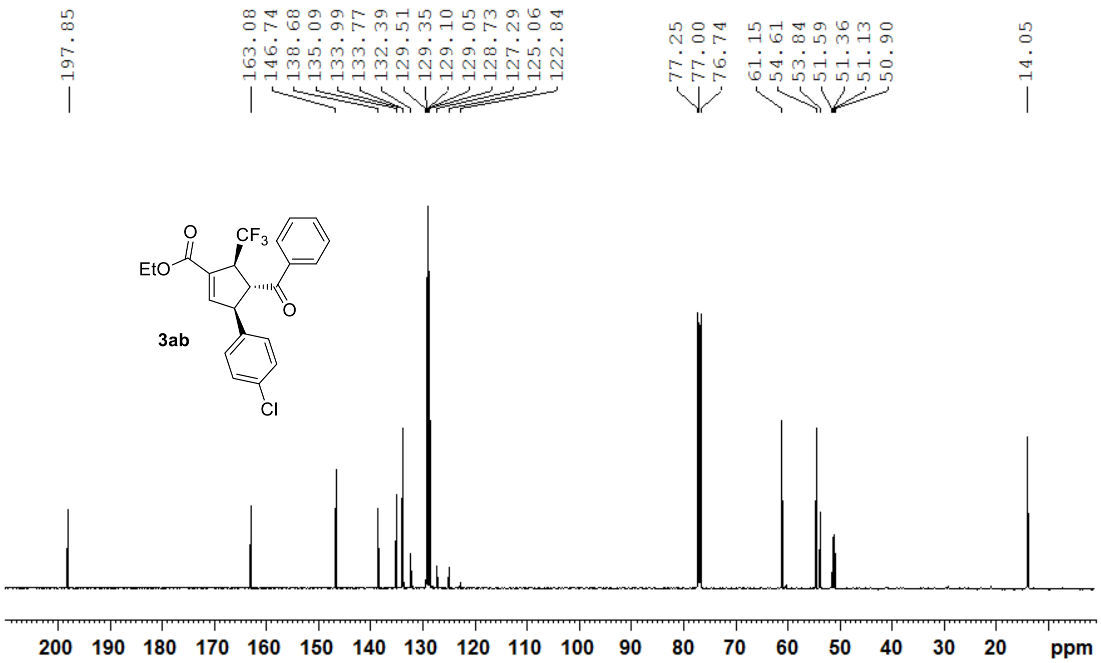

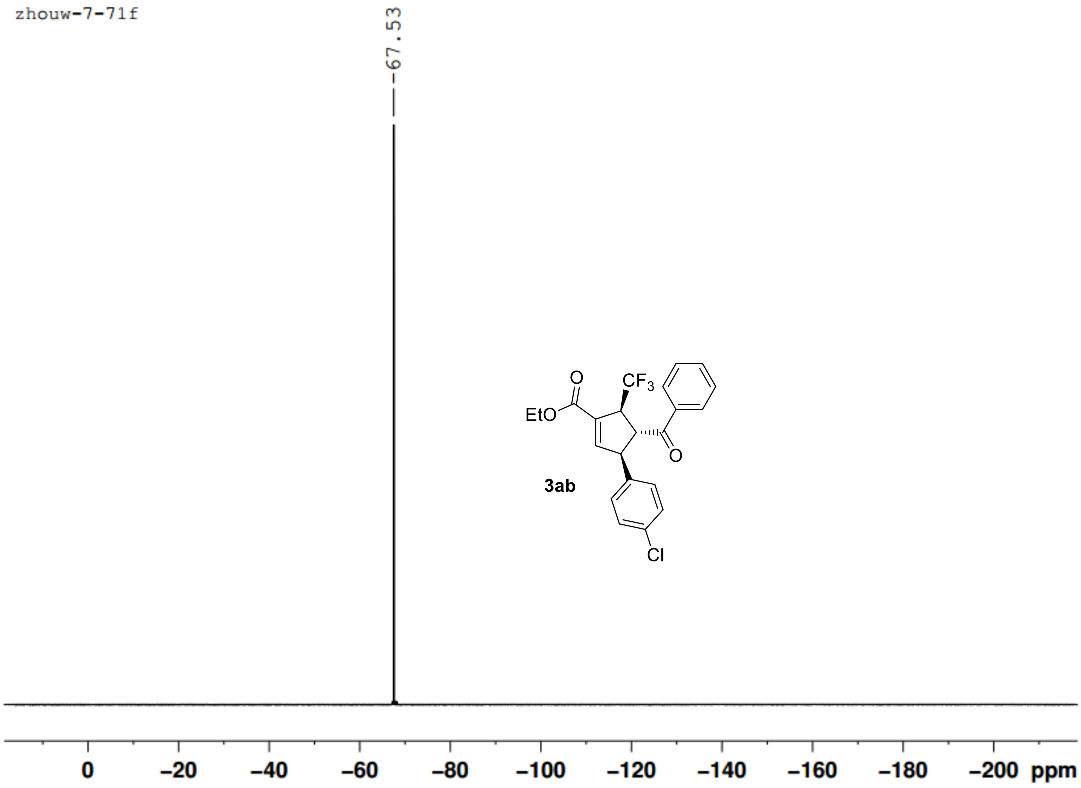
zhouw-7-71f
-67.53
EtO
O
CF3
3ab
Cl
0 −20 −40 −60 −80 −100 −120 −140 −160 −180 −200 ppm

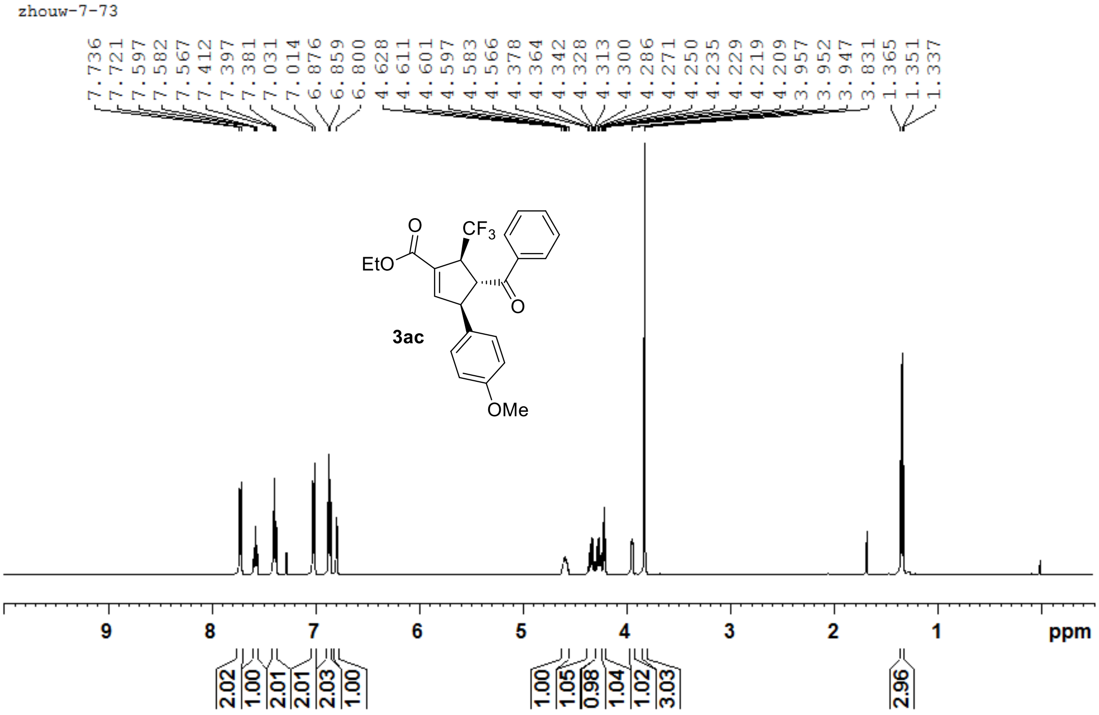
zhouw-7-73
7.736 7.721 7.597 7.582 7.567 7.412 7.397 7.381 7.031 7.014 6.876 6.859 6.800 4.628 4.611 4.601 4.597 4.583 4.566 4.378 4.364 4.342 4.328 4.313 4.300 4.286 4.271 4.250 4.235 4.229 4.219 4.209 3.957 3.952 3.947 3.831 1.365 1.351 1.337
EtO
O
CF3
3ac
OMe
9 8 7 6 5 4 3 2 1 ppm
2.02 1.00 2.01 2.01 2.03 1.00 1.00 1.05 0.98 1.04 1.02 3.03 2.96

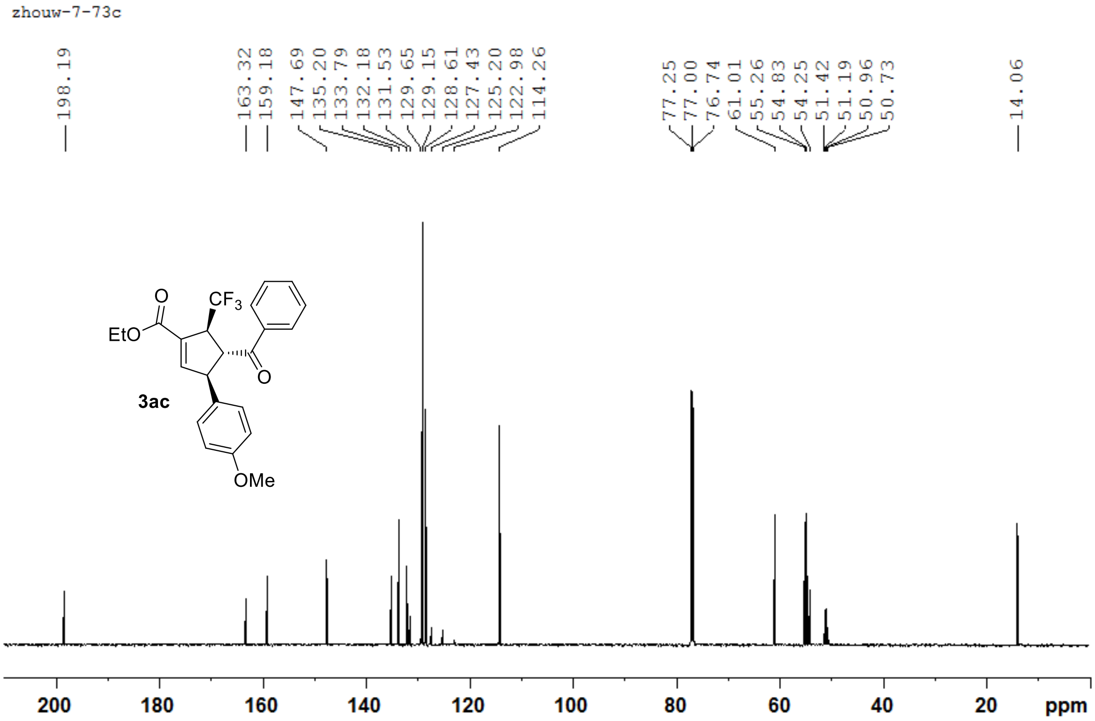
zhouw-7-73c
198.19
163.32
159.18
147.69
135.20
133.79
132.18
131.53
129.65
129.15
128.61
127.43
125.20
122.98
114.26
77.25
77.00
76.74
61.01
55.26
54.83
54.25
51.42
51.19
50.96
50.73
14.06
EtO
O
CF3
O
3ac
OMe
200
180
160
140
120
100
80
60
40
20
ppm

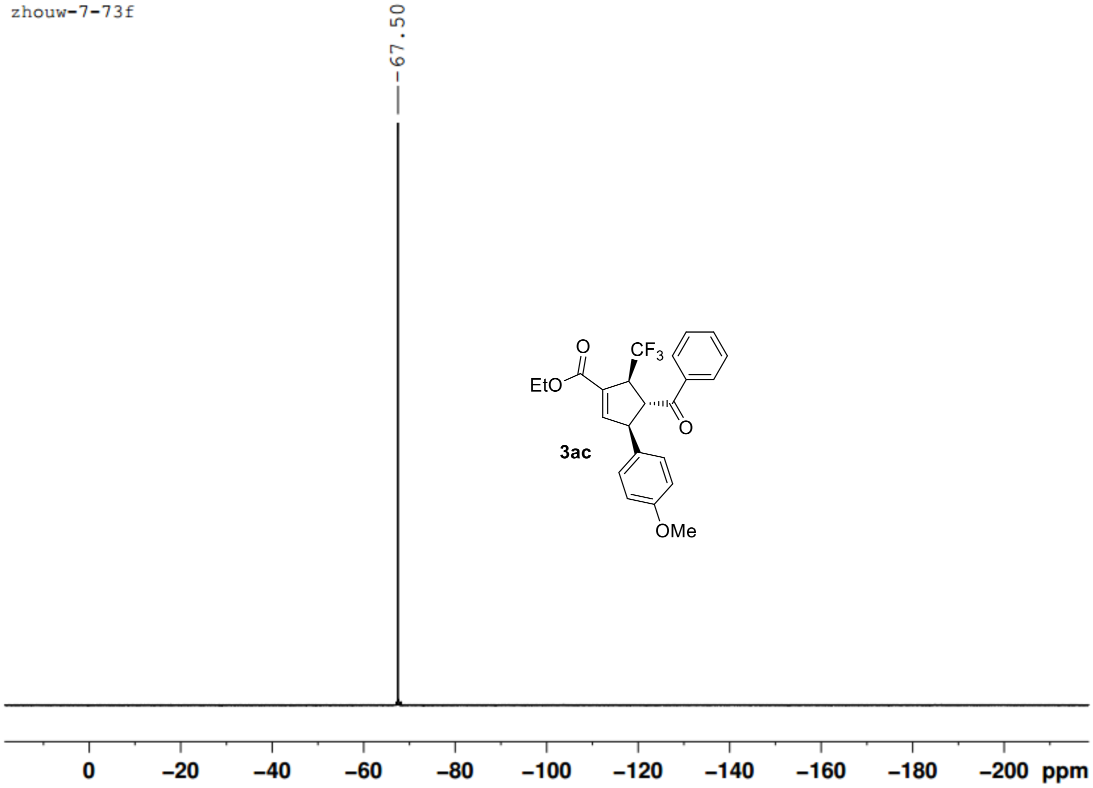
zhouw-7-73f
-67.50
EtO
O
CF3
O
3ac
OMe
0
−20
−40
−60
−80
−100
−120
−140
−160
−180
−200
ppm

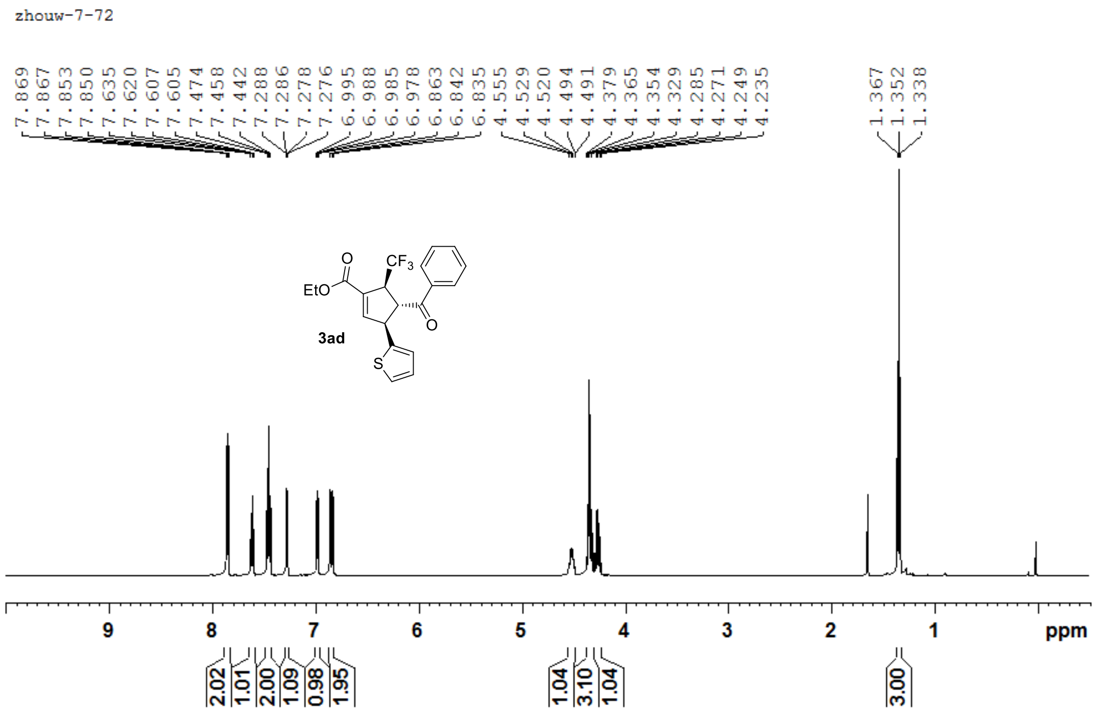
zhouw-7-72
7.869
7.867
7.853
7.850
7.635
7.620
7.607
7.605
7.474
7.458
7.442
7.288
7.286
7.278
7.276
6.995
6.988
6.985
6.978
6.863
6.842
6.835
4.555
4.529
4.520
4.494
4.491
4.379
4.365
4.354
4.329
4.285
4.271
4.249
4.235
1.367
1.352
1.338
EtO
O
CF3
O
S
3ad
9
8
7
6
5
4
3
2
1
ppm
2.02
1.01
2.00
1.09
0.98
1.95
1.04
3.10
1.04
3.00

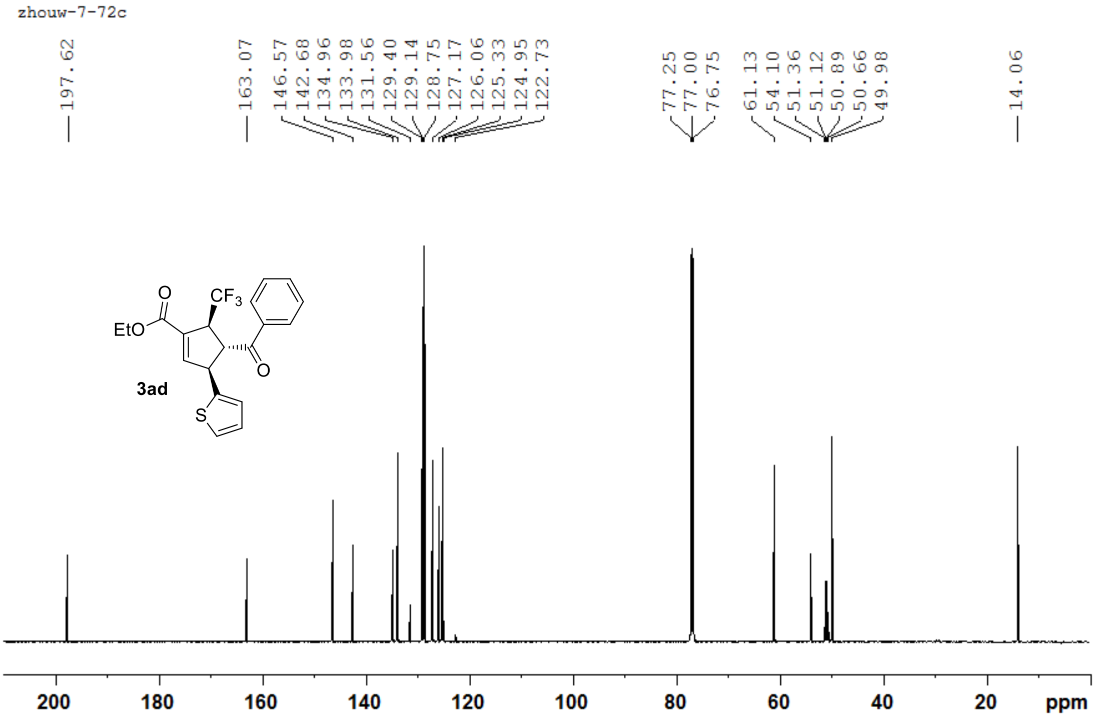
zhouw-7-72c
197.62
163.07
146.57
142.68
134.96
133.98
131.56
129.40
129.14
128.75
127.17
126.06
125.33
124.95
122.73
77.25
77.00
76.75
61.13
54.10
51.36
51.12
50.89
50.66
49.98
14.06
EtO
O
CF3
O
S
3ad
200
180
160
140
120
100
80
60
40
20
ppm

zhouw-7-72f

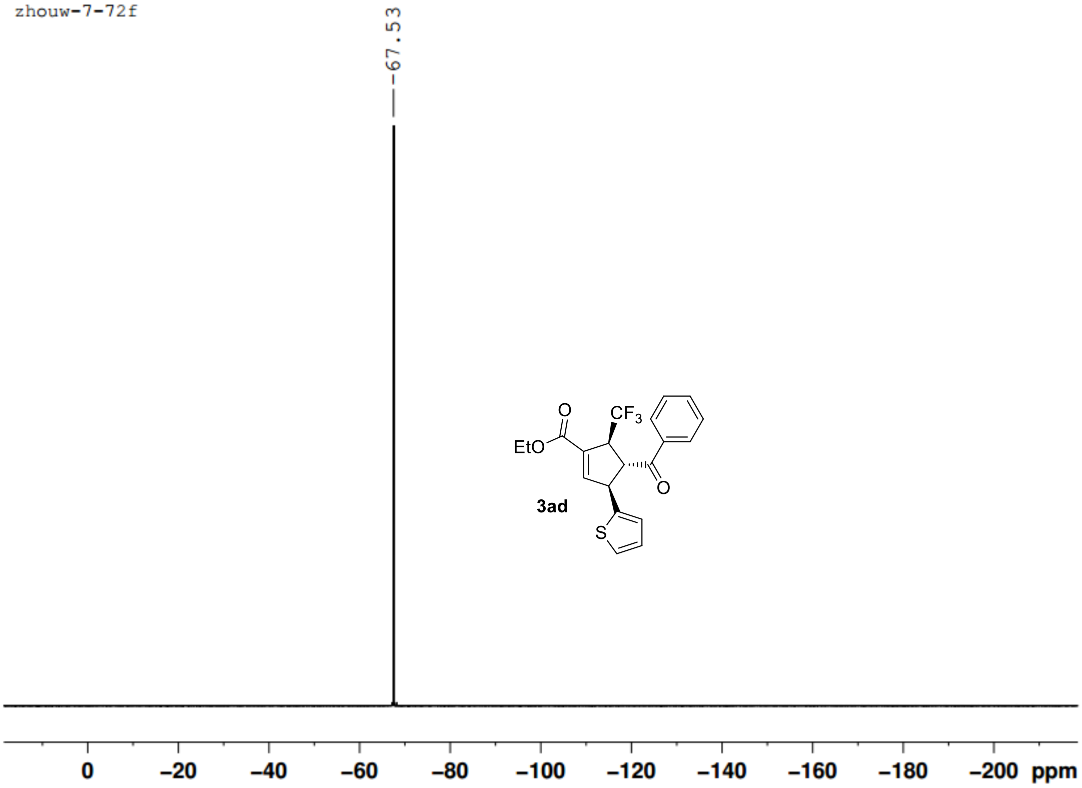

zhouw-6-117

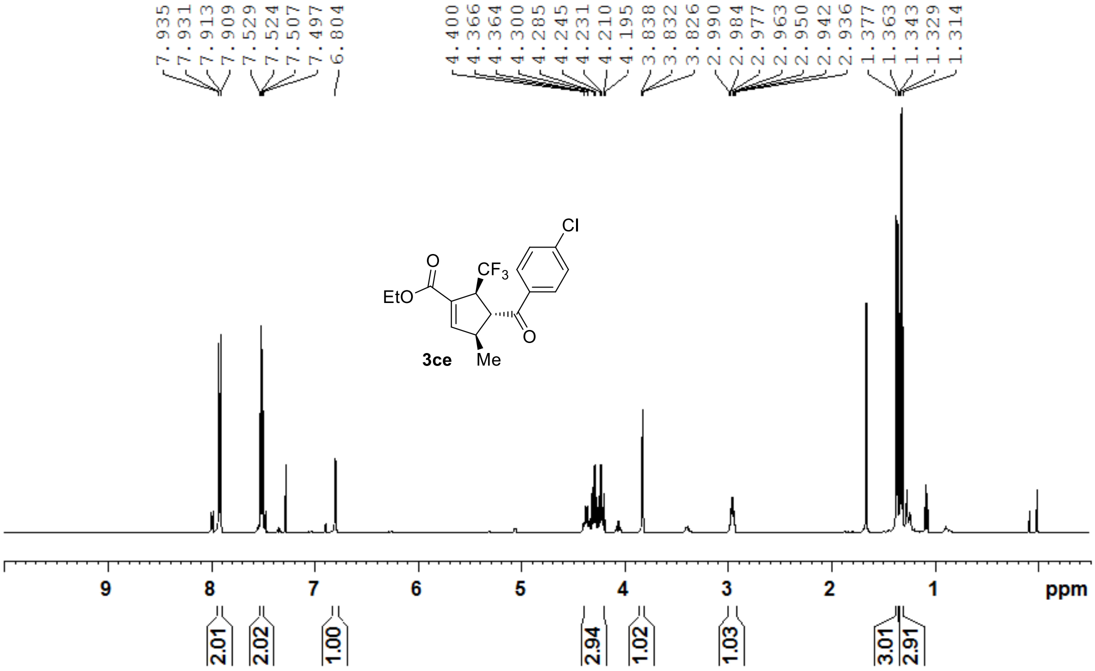

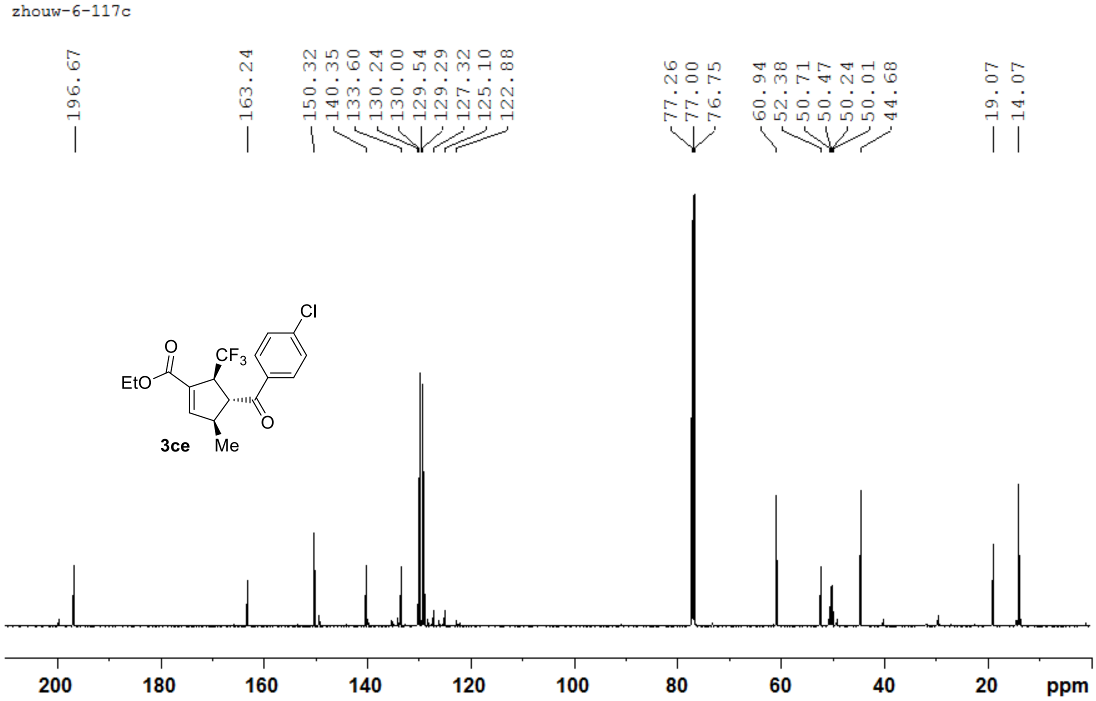
zhouw-6-117c
196.67
163.24
150.32
140.35
133.60
130.24
130.00
129.54
129.29
127.32
125.10
122.88
77.26
77.00
76.75
60.94
52.38
50.71
50.47
50.24
50.01
44.68
19.07
14.07
Cl
O
CF3
EtO
O
3ce
Me
200
180
160
140
120
100
80
60
40
20
ppm

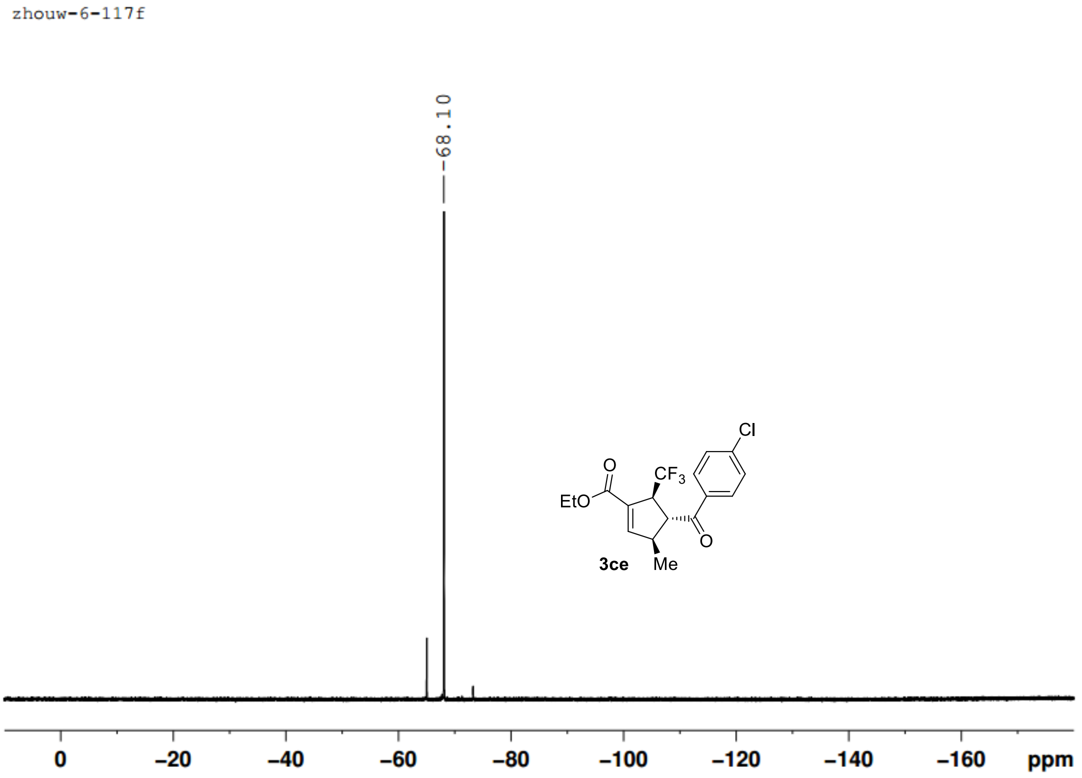
zhouw-6-117f
-68.10
Cl
O
CF3
EtO
O
3ce
Me
0
-20
-40
-60
-80
-100
-120
-140
-160
ppm

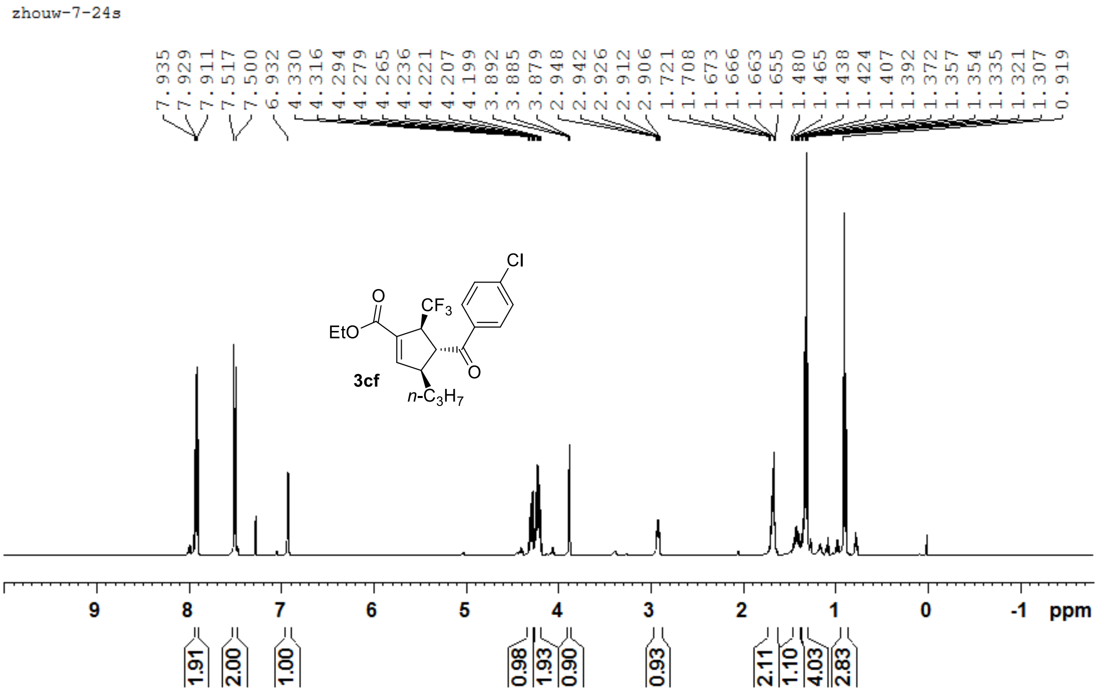
zhouw-7-24s
7.935 7.929 7.911 7.517 7.500 6.932 4.330 4.316 4.294 4.279 4.265 4.236 4.221 4.207 4.199 3.892 3.885 3.879 2.948 2.942 2.926 2.912 2.906 1.721 1.708 1.673 1.666 1.663 1.655 1.480 1.465 1.438 1.424 1.407 1.392 1.372 1.357 1.354 1.335 1.321 1.307 0.919
Cl
CF3
EtO
O
3cf
n-C3H7
9 8 7 6 5 4 3 2 1 0 -1 ppm
1.91 2.00 1.00 0.98 1.93 0.90 0.93 2.11 1.10 4.03 2.83

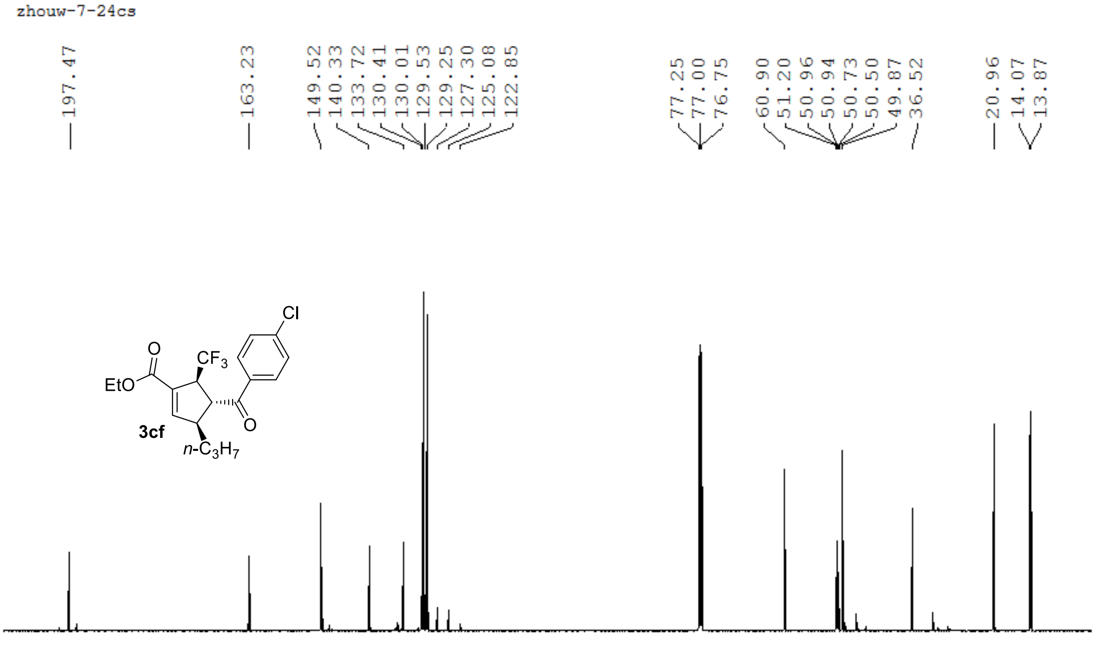
zhouw-7-24cs
197.47 163.23 149.52 140.33 133.72 130.41 130.01 129.53 129.25 127.30 125.08 122.85 77.25 77.00 76.75 60.90 51.20 50.96 50.94 50.73 50.50 49.87 36.52 20.96 14.07 13.87
Cl
CF3
EtO
O
3cf
n-C3H7
200 190 180 170 160 150 140 130 120 110 100 90 80 70 60 50 40 30 20 ppm

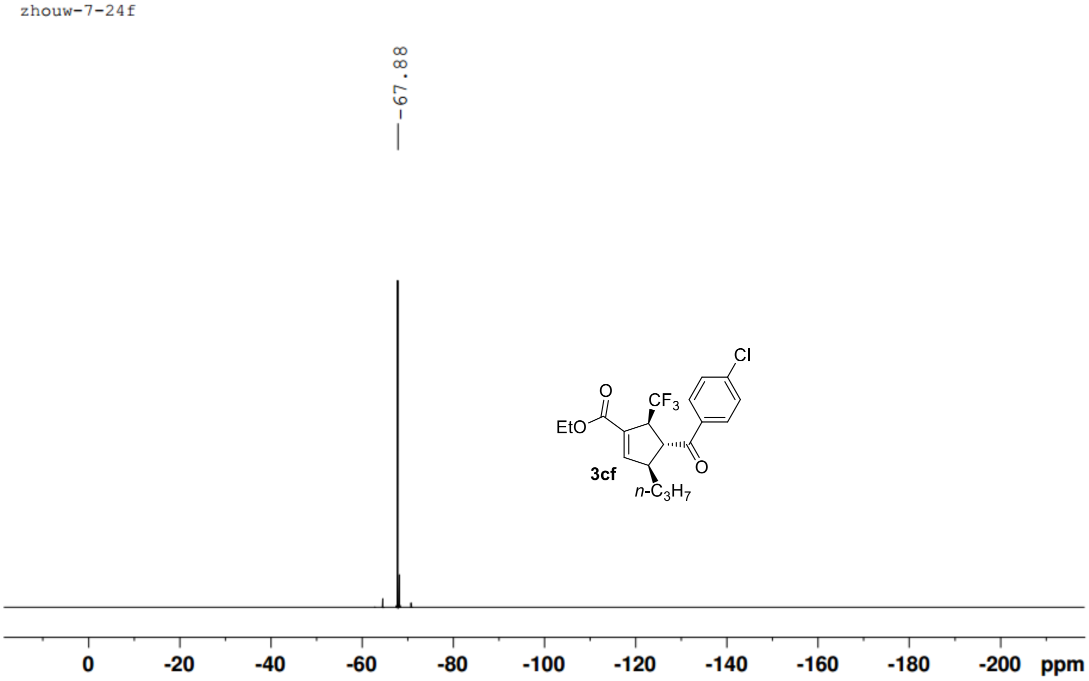
zhouw-7-24f
-67.88
Cl
O
CF3
EtO
O
3cf
n-C3H7
0
-20
-40
-60
-80
-100
-120
-140
-160
-180
-200
ppm

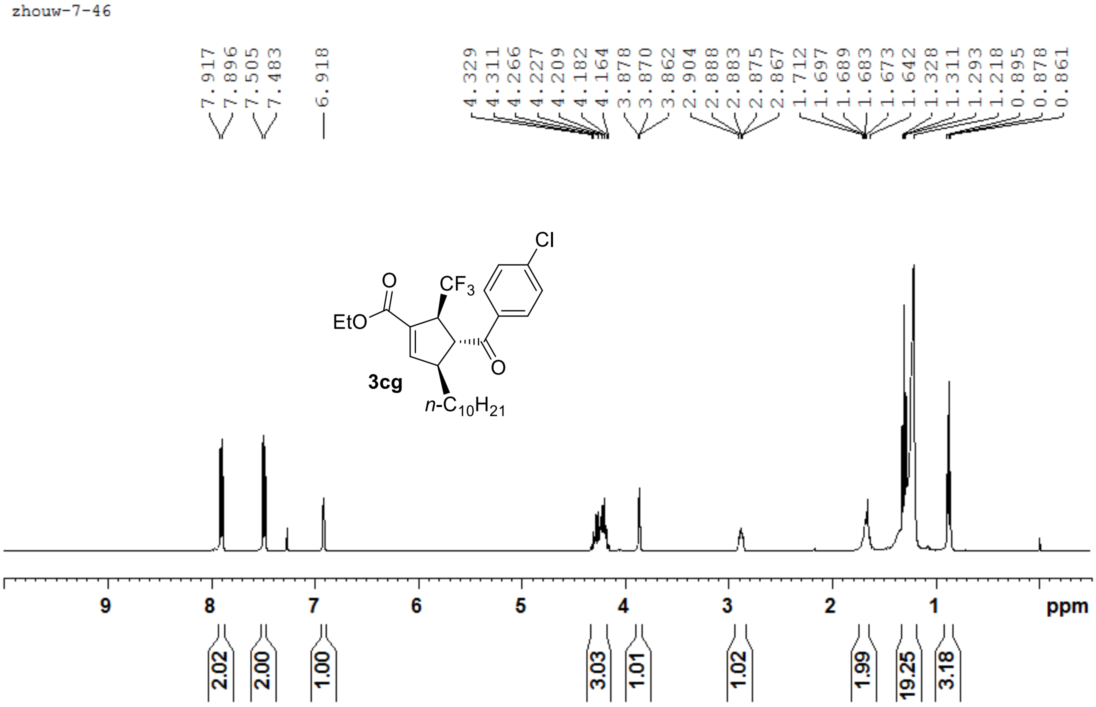
zhouw-7-46
7.917
7.896
7.505
7.483
6.918
4.329
4.311
4.266
4.227
4.209
4.182
4.164
3.878
3.870
3.862
2.904
2.888
2.883
2.875
2.867
1.712
1.697
1.689
1.683
1.673
1.642
1.328
1.311
1.293
1.218
0.895
0.878
0.861
Cl
O
CF3
EtO
O
3cg
n-C10H21
9
8
7
6
5
4
3
2
1
ppm
2.02
2.00
1.00
3.03
1.01
1.02
1.99
19.25
3.18

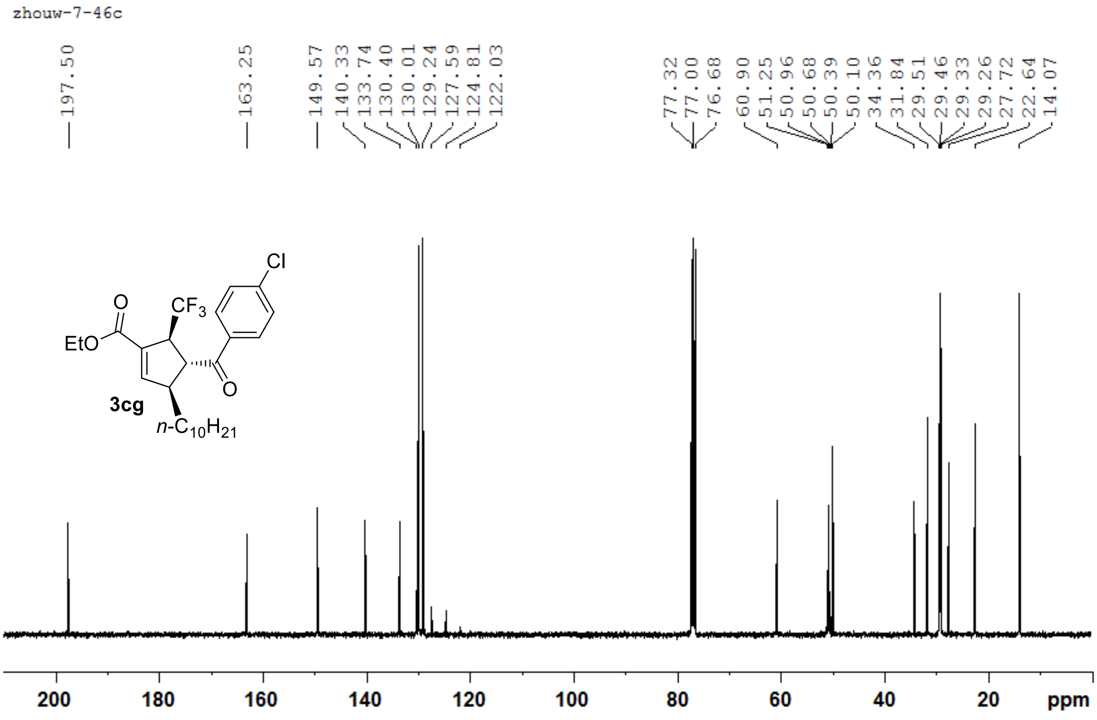
zhouw-7-46c
197.50
163.25
149.57
140.33
133.74
130.40
130.01
129.24
127.59
124.81
122.03
77.32
77.00
76.68
60.90
51.25
50.96
50.68
50.39
50.10
34.36
31.84
29.51
29.46
29.33
29.26
27.72
22.64
14.07
Cl
CF3
O
EtO
O
3cg
n-C10H21
200
180
160
140
120
100
80
60
40
20
ppm

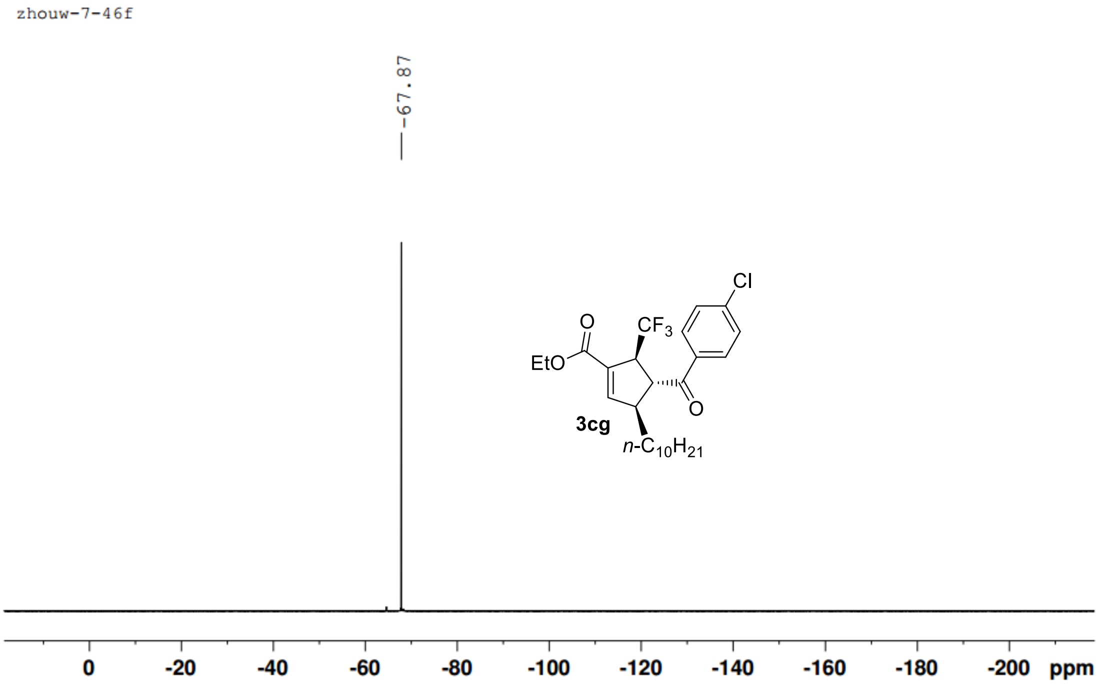
zhouw-7-46f
-67.87
Cl
CF3
O
EtO
O
3cg
n-C10H21
0
-20
-40
-60
-80
-100
-120
-140
-160
-180
-200
ppm

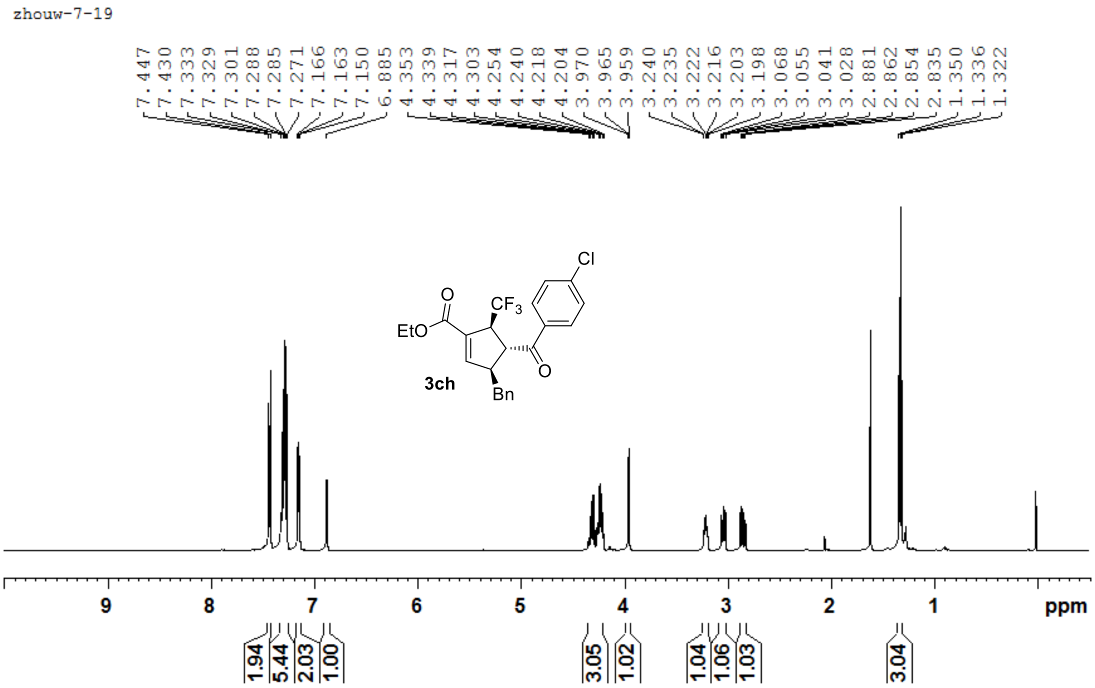
zhouw-7-19
7.447 7.430 7.333 7.329 7.301 7.288 7.285 7.271 7.166 7.163 7.150 6.885 4.353 4.339 4.317 4.303 4.254 4.240 4.218 4.204 3.970 3.965 3.959 3.240 3.235 3.222 3.216 3.203 3.198 3.068 3.055 3.041 3.028 2.881 2.862 2.854 2.835 1.350 1.336 1.322
EtO
O
CF3
Cl
O
3ch
Bn
9 8 7 6 5 4 3 2 1 ppm
1.94 5.44 2.03 1.00 3.05 1.02 1.04 1.06 1.03 3.04

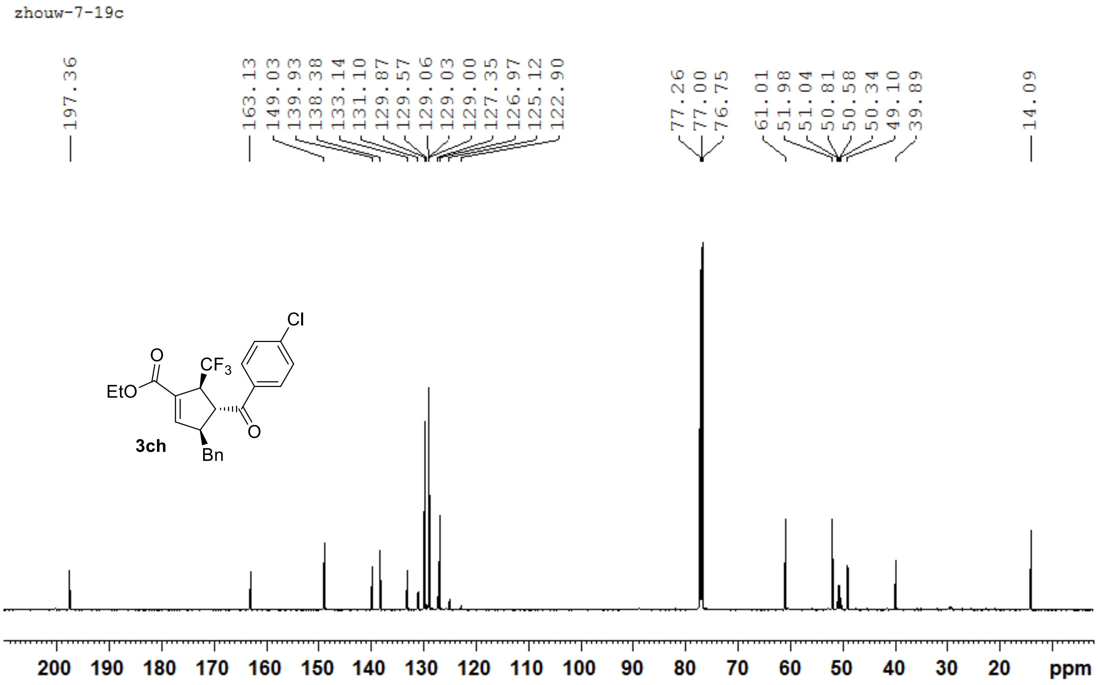
zhouw-7-19c
197.36
163.13 149.03 139.93 138.38 133.14 131.10 129.87 129.57 129.06 129.03 129.00 127.35 126.97 125.12 122.90
77.26 77.00 76.75
61.01 51.98 51.04 50.81 50.58 50.34 49.10 39.89
14.09
EtO
O
CF3
Cl
O
3ch
Bn
200 190 180 170 160 150 140 130 120 110 100 90 80 70 60 50 40 30 20 ppm

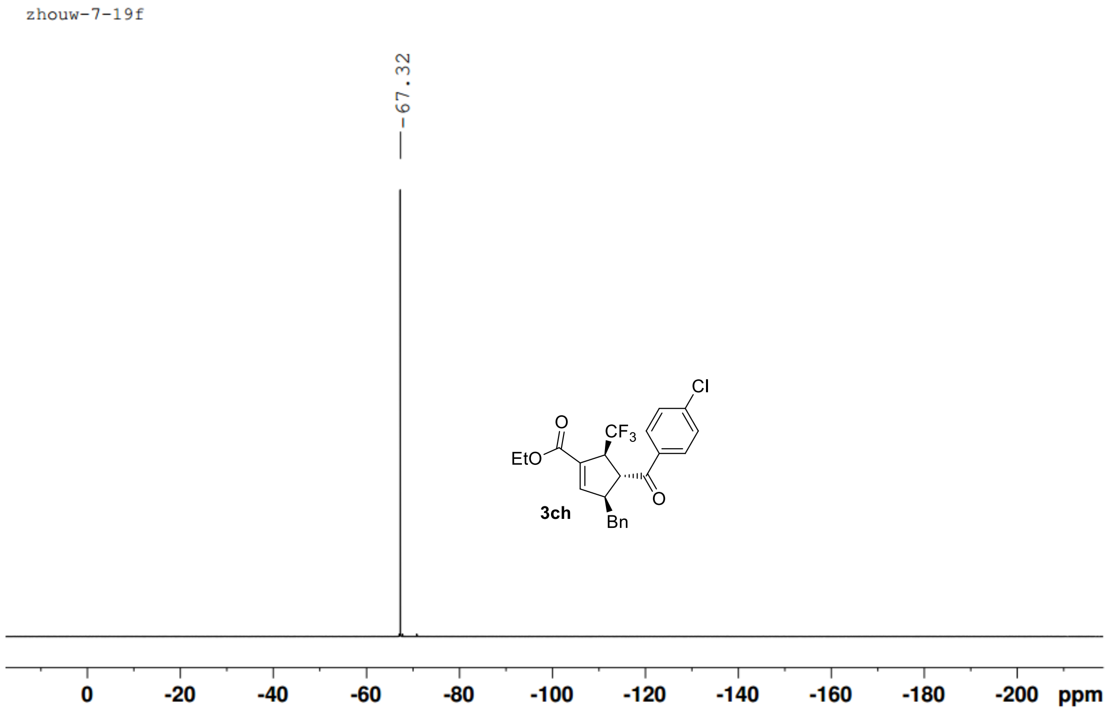
zhouw-7-19f
-67.32
EtO
O
CF3
Cl
O
3ch
Bn
0
-20
-40
-60
-80
-100
-120
-140
-160
-180
-200
ppm

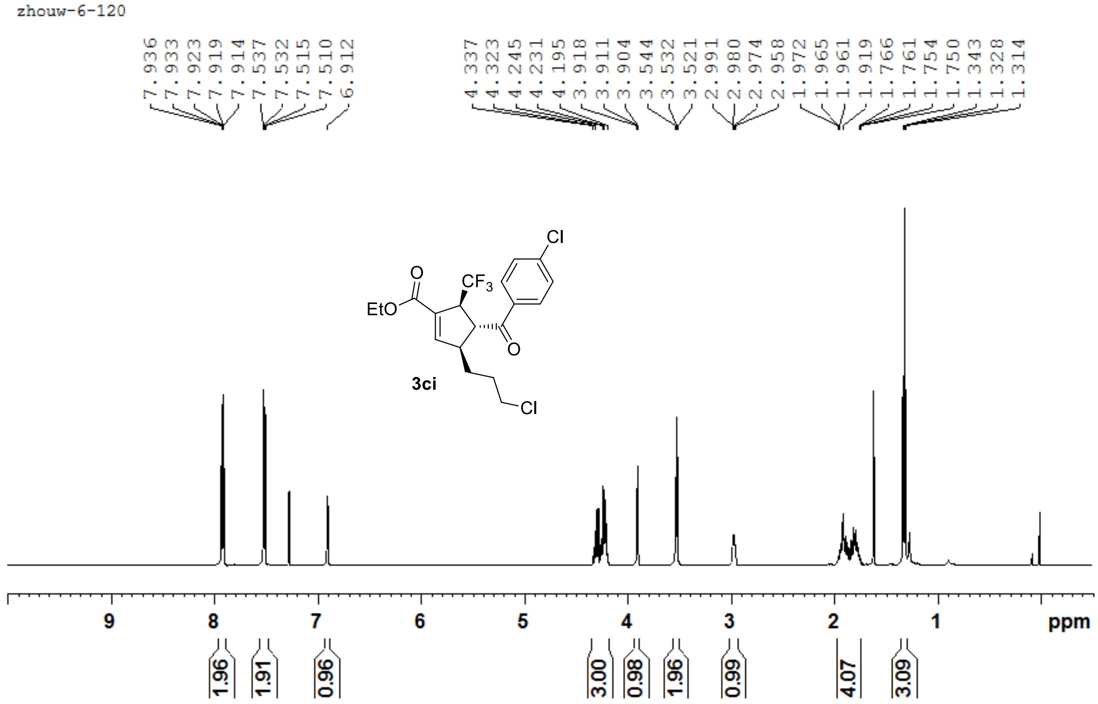
zhouw-6-120
7.936
7.933
7.923
7.919
7.914
7.537
7.532
7.515
7.510
6.912
4.337
4.323
4.245
4.231
4.195
3.918
3.911
3.904
3.544
3.532
3.521
2.991
2.980
2.974
2.958
1.972
1.965
1.961
1.919
1.766
1.761
1.754
1.750
1.343
1.328
1.314
EtO
O
CF3
Cl
O
3ci
Cl
9
8
7
6
5
4
3
2
1
ppm
1.96
1.91
0.96
3.00
0.98
1.96
0.99
4.07
3.09

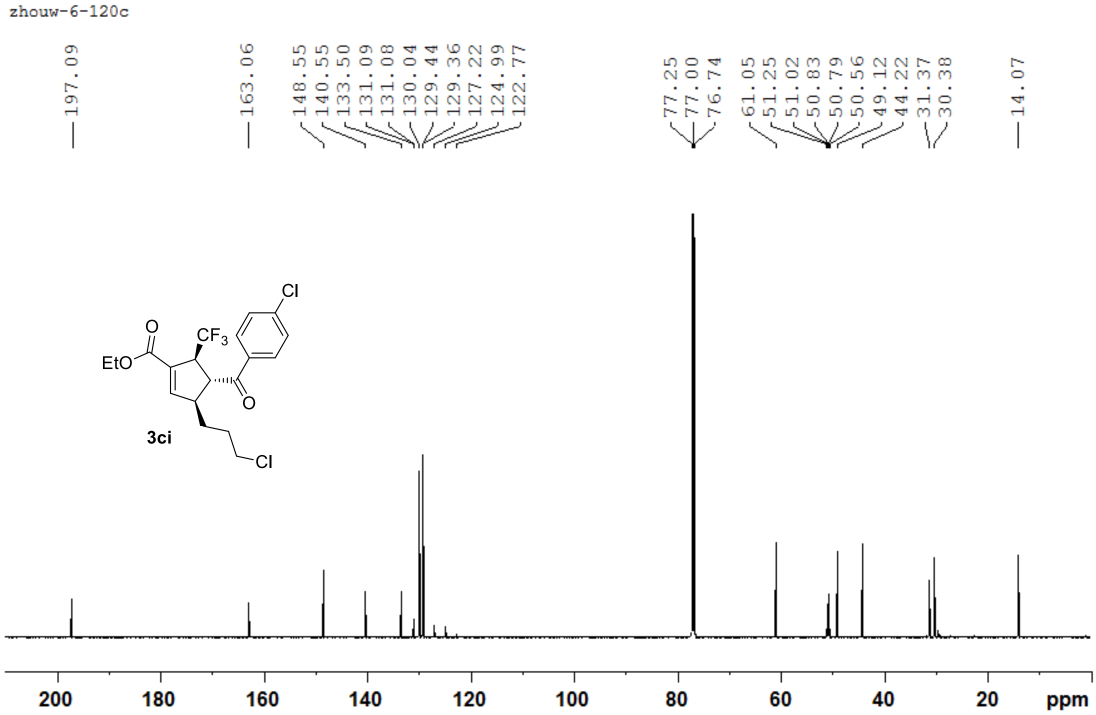
zhouw-6-120c
197.09
163.06
148.55
140.55
133.50
131.09
131.08
130.04
129.44
129.36
127.22
124.99
122.77
77.25
77.00
76.74
61.05
51.25
51.02
50.83
50.79
50.56
49.12
44.22
31.37
30.38
14.07
Cl
O
CF3
EtO
O
3ci
Cl
200
180
160
140
120
100
80
60
40
20
ppm

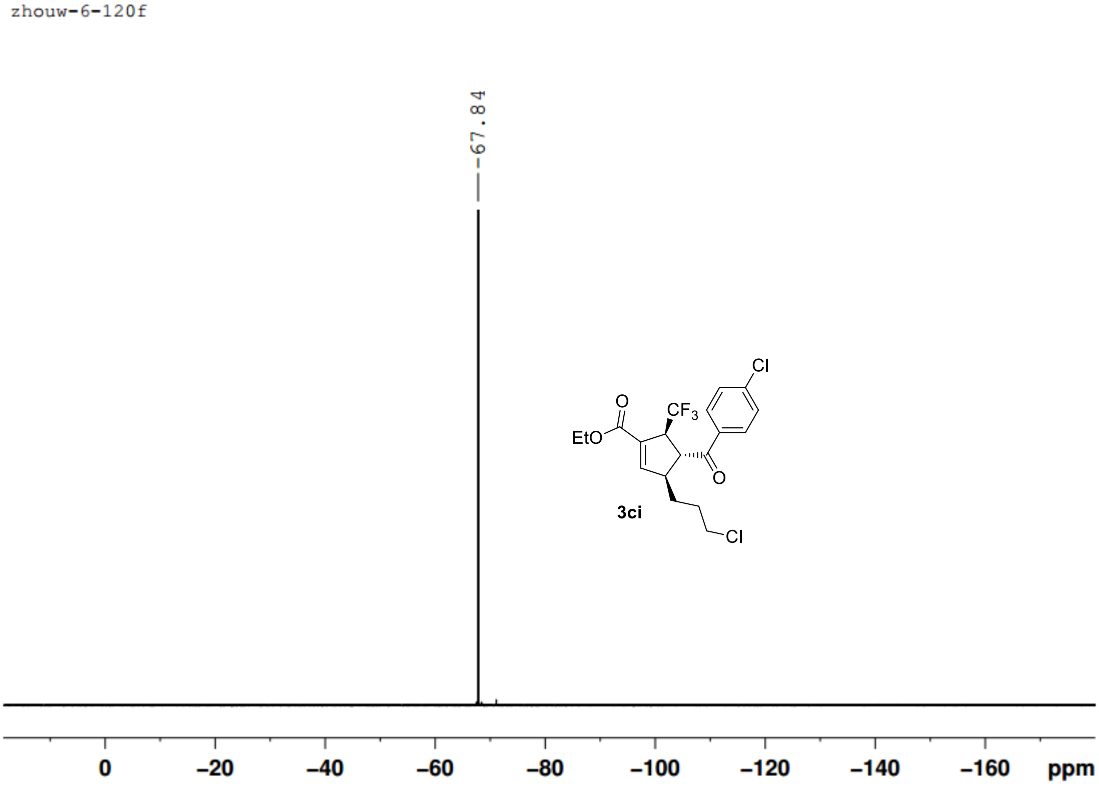
zhouw-6-120f
-67.84
Cl
O
CF3
EtO
O
3ci
Cl
0
–20
–40
–60
–80
–100
–120
–140
–160
ppm

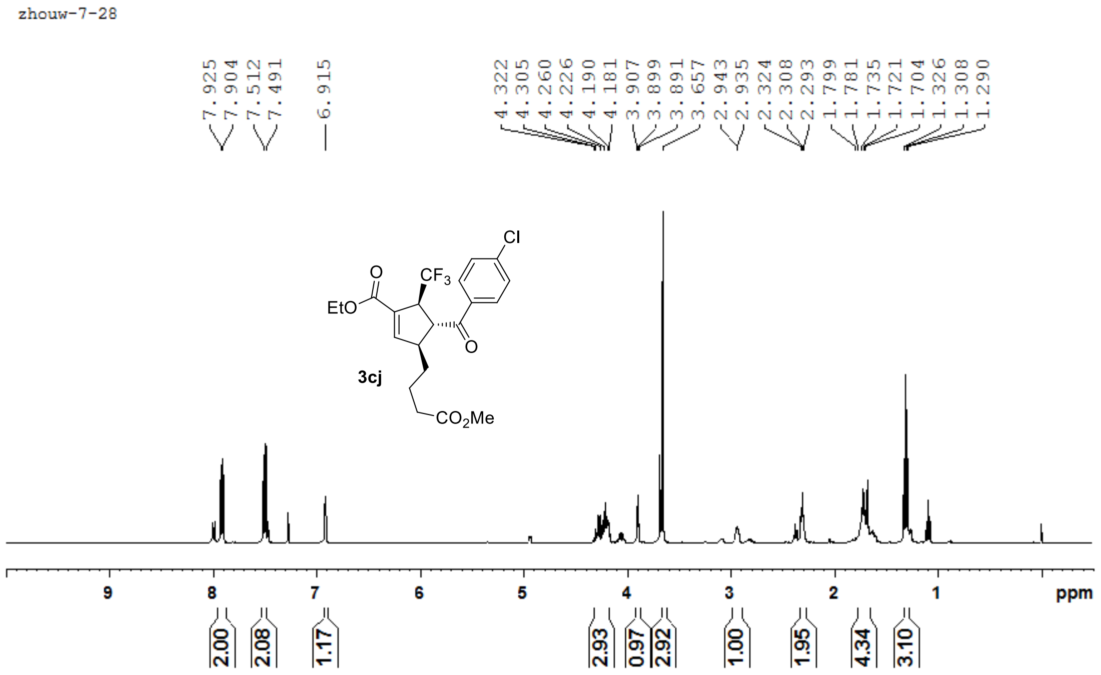
zhouw-7-28
7.925
7.904
7.512
7.491
6.915
4.322
4.305
4.260
4.226
4.190
4.181
3.907
3.899
3.891
3.657
2.943
2.935
2.324
2.308
2.293
1.799
1.781
1.735
1.721
1.704
1.326
1.308
1.290
Cl
O
CF3
EtO
O
3cj
CO2Me
9
8
7
6
5
4
3
2
1
ppm
2.00
2.08
1.17
2.93
0.97
2.92
1.00
1.95
4.34
3.10

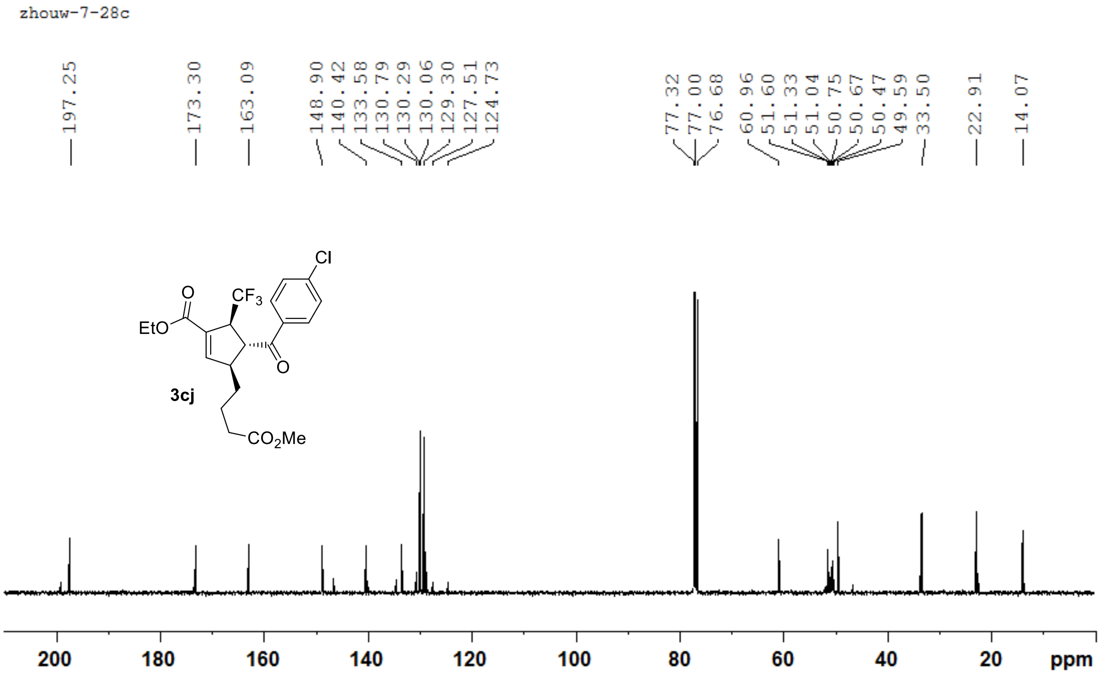
zhouw-7-28c
197.25
173.30
163.09
148.90
140.42
133.58
130.79
130.29
130.06
129.30
127.51
124.73
77.32
77.00
76.68
60.96
51.60
51.33
51.04
50.75
50.67
50.47
49.59
33.50
22.91
14.07
Cl
O
CF3
EtO
O
3cj
CO2Me
200
180
160
140
120
100
80
60
40
20
ppm

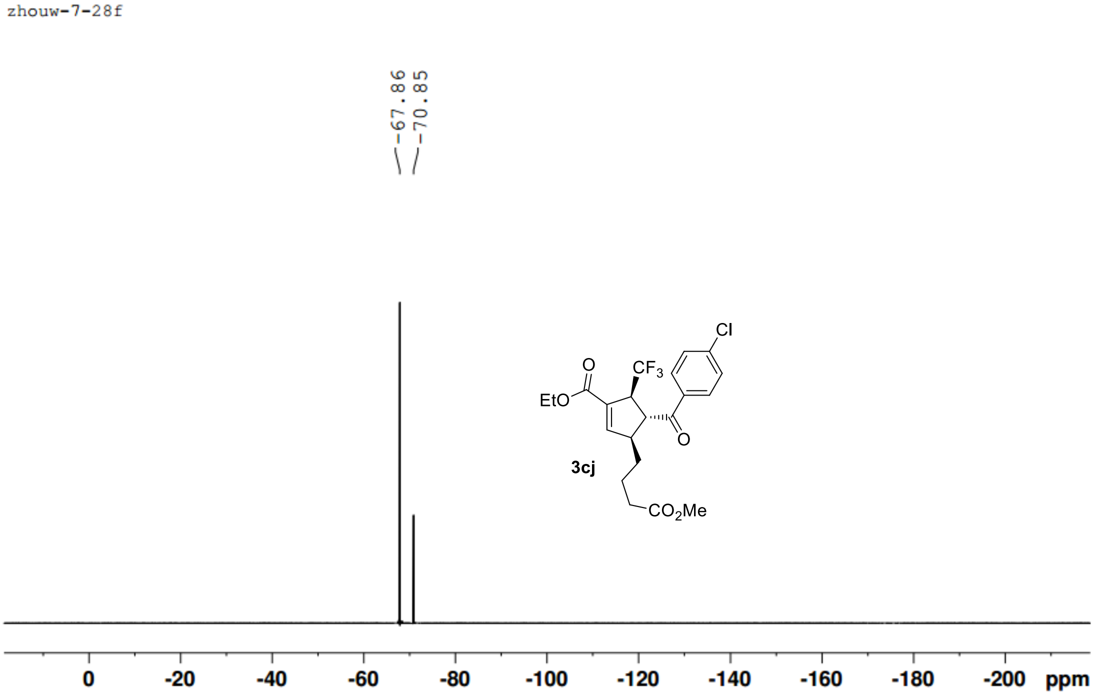
zhouw-7-28f
-67.86
-70.85
3cj
ppm

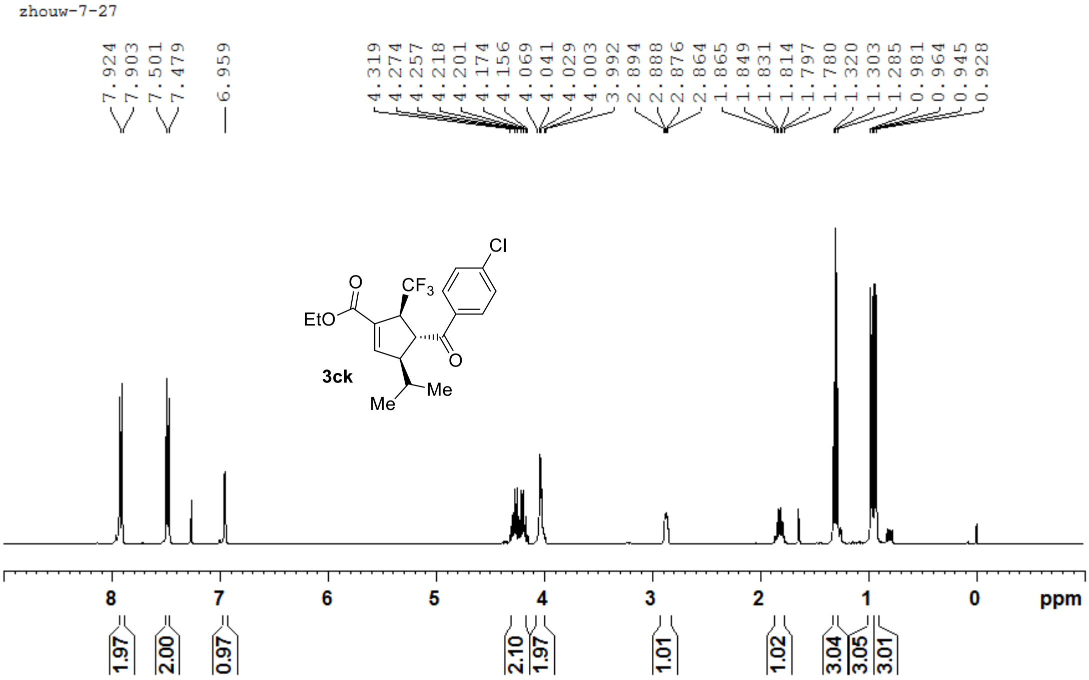
zhouw-7-27
3ck
ppm

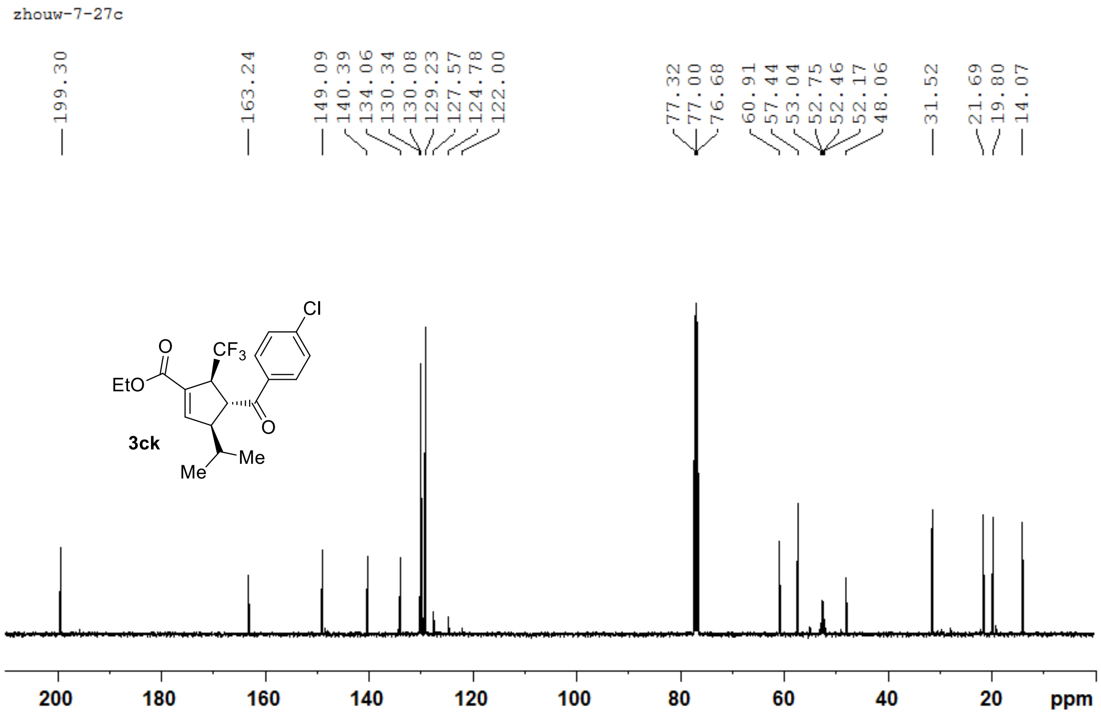
zhouw-7-27c
199.30
163.24
149.09
140.39
134.06
130.34
130.08
129.23
127.57
124.78
122.00
77.32
77.00
76.68
60.91
57.44
53.04
52.75
52.46
52.17
48.06
31.52
21.69
19.80
14.07
Cl
O
CF3
EtO
O
3ck
Me
Me
200
180
160
140
120
100
80
60
40
20
ppm

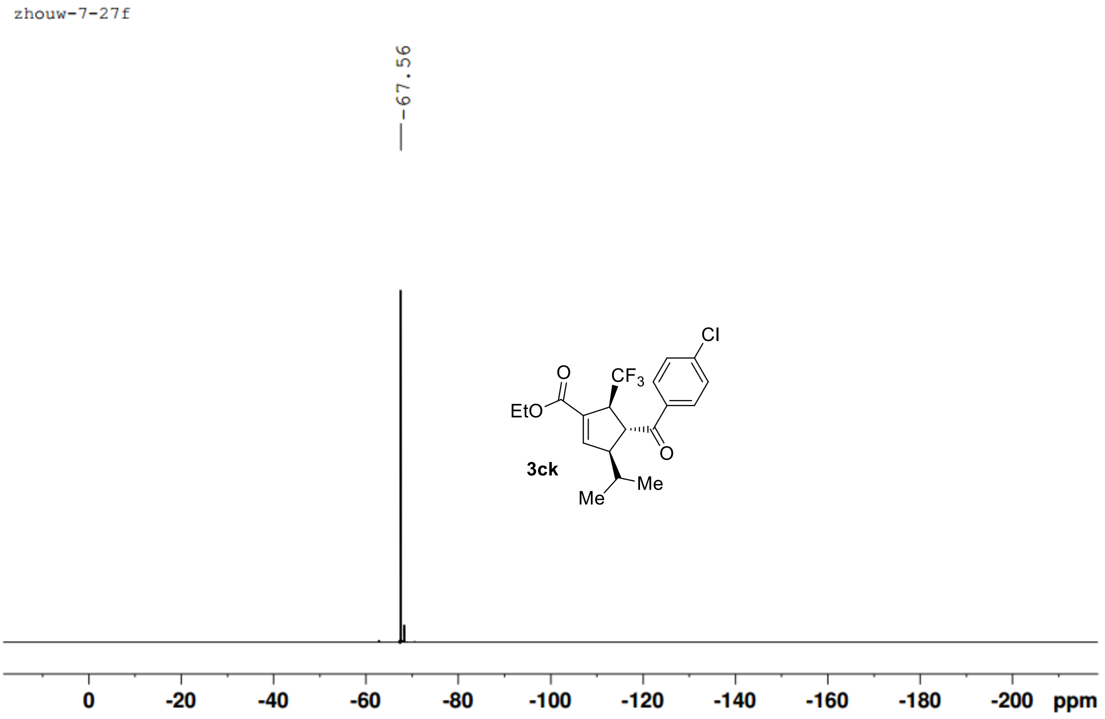
zhouw-7-27f
-67.56
Cl
O
CF3
EtO
O
3ck
Me
Me
0
-20
-40
-60
-80
-100
-120
-140
-160
-180
-200
ppm

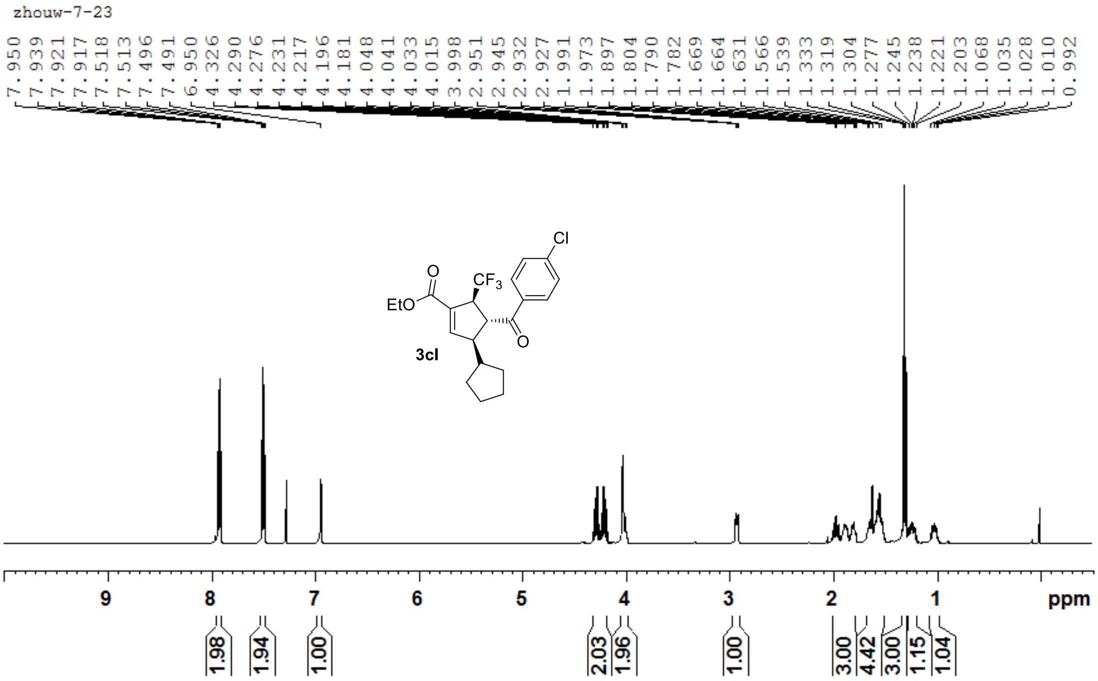
zhouw-7-23
7.950
7.939
7.921
7.917
7.518
7.513
7.496
7.491
6.950
4.326
4.290
4.276
4.231
4.217
4.196
4.181
4.048
4.041
4.033
4.015
3.998
2.951
2.945
2.932
2.927
1.991
1.973
1.897
1.804
1.790
1.782
1.669
1.664
1.631
1.566
1.539
1.333
1.319
1.304
1.277
1.245
1.238
1.221
1.203
1.068
1.035
1.028
1.010
0.992
Cl
CF3
O
EtO
O
3cl
9
8
7
6
5
4
3
2
1
ppm
1.98
1.94
1.00
2.03
1.96
1.00
3.00
4.42
3.00
1.15
1.04

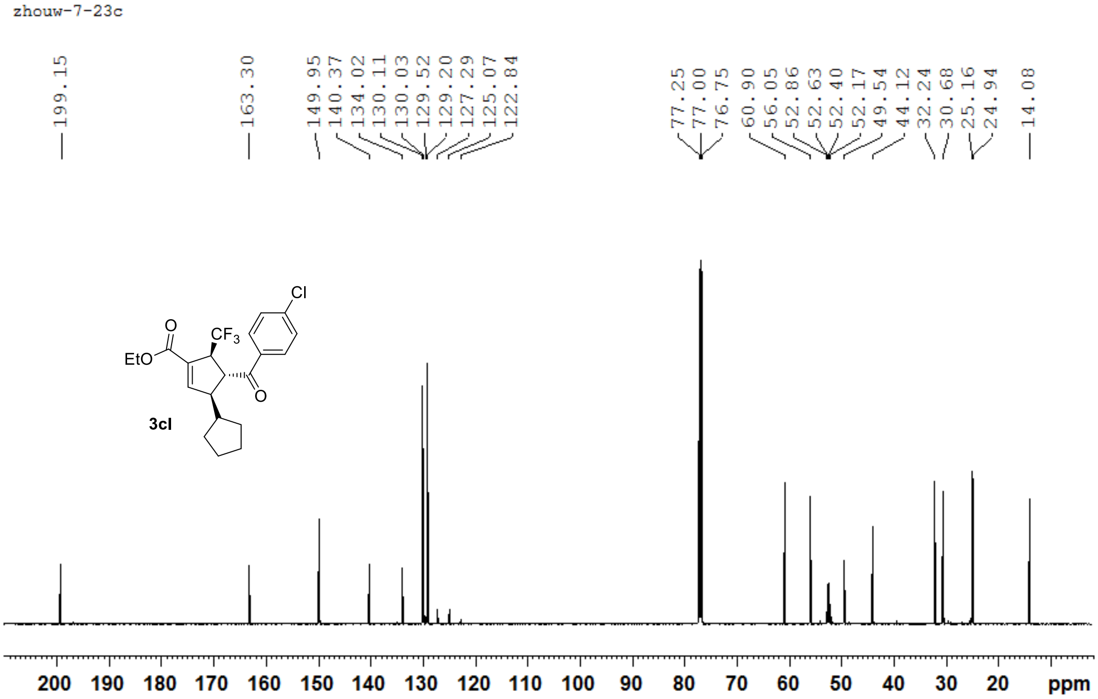
zhouw-7-23c
199.15
163.30
149.95
140.37
134.02
130.11
130.03
129.52
129.20
127.29
125.07
122.84
77.25
77.00
76.75
60.90
56.05
52.86
52.63
52.40
52.17
49.54
44.12
32.24
30.68
25.16
24.94
14.08
Cl
CF3
O
EtO
O
3cl
200
190
180
170
160
150
140
130
120
110
100
90
80
70
60
50
40
30
20
ppm

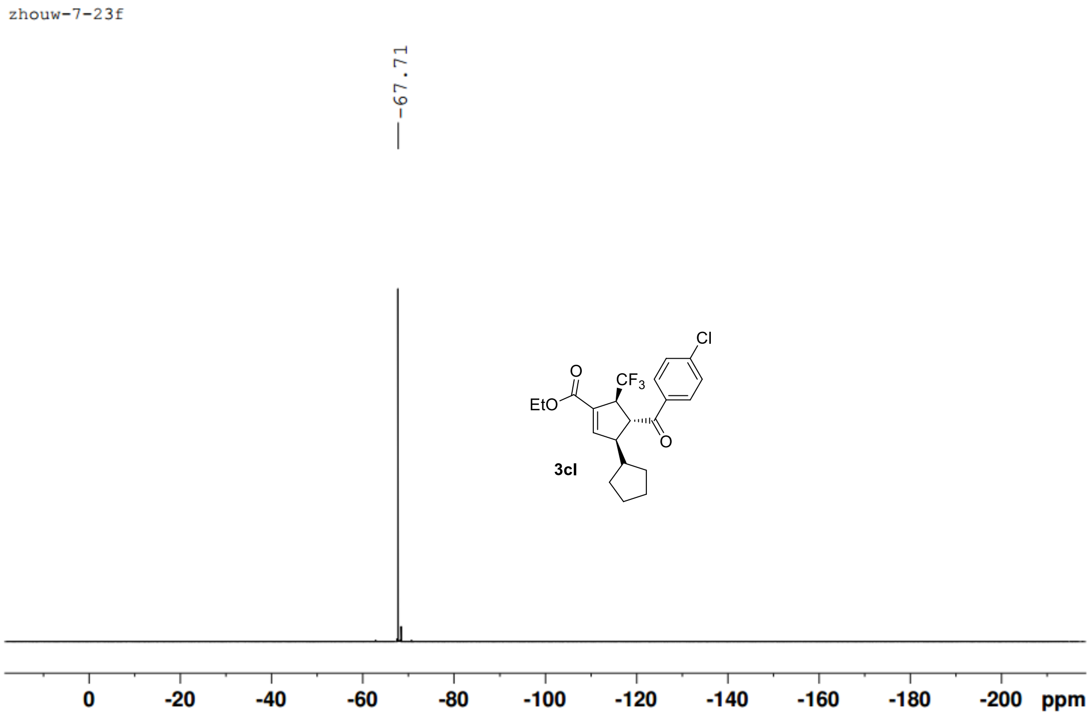
zhouw-7-23f
-67.71
Cl
O
CF3
EtO
O
3cl
0 -20 -40 -60 -80 -100 -120 -140 -160 -180 -200 ppm

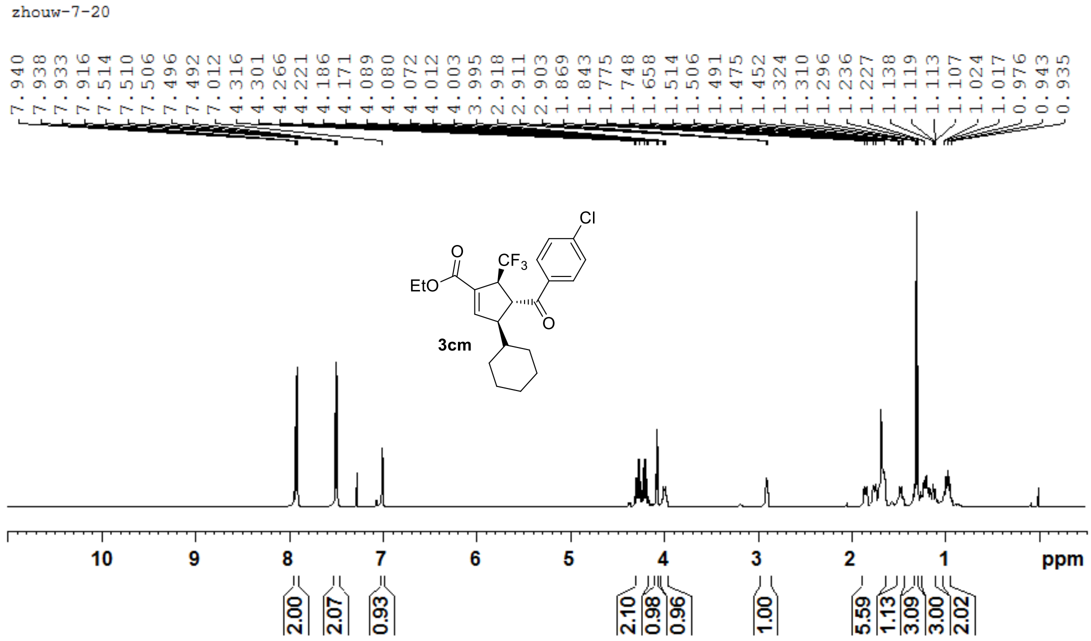
zhouw-7-20
7.940 7.938 7.933 7.916 7.514 7.510 7.506 7.496 7.492 7.012 4.316 4.301 4.266 4.221 4.186 4.171 4.089 4.080 4.072 4.012 4.003 3.995 2.918 2.911 2.903 1.869 1.843 1.775 1.748 1.658 1.514 1.506 1.491 1.475 1.452 1.324 1.310 1.296 1.236 1.227 1.138 1.119 1.113 1.107 1.024 1.017 0.976 0.943 0.935
Cl
O
CF3
EtO
O
3cm
10 9 8 7 6 5 4 3 2 1 ppm
2.00 2.07 0.93 2.10 0.98 0.96 1.00 5.59 1.13 3.09 3.00 2.02

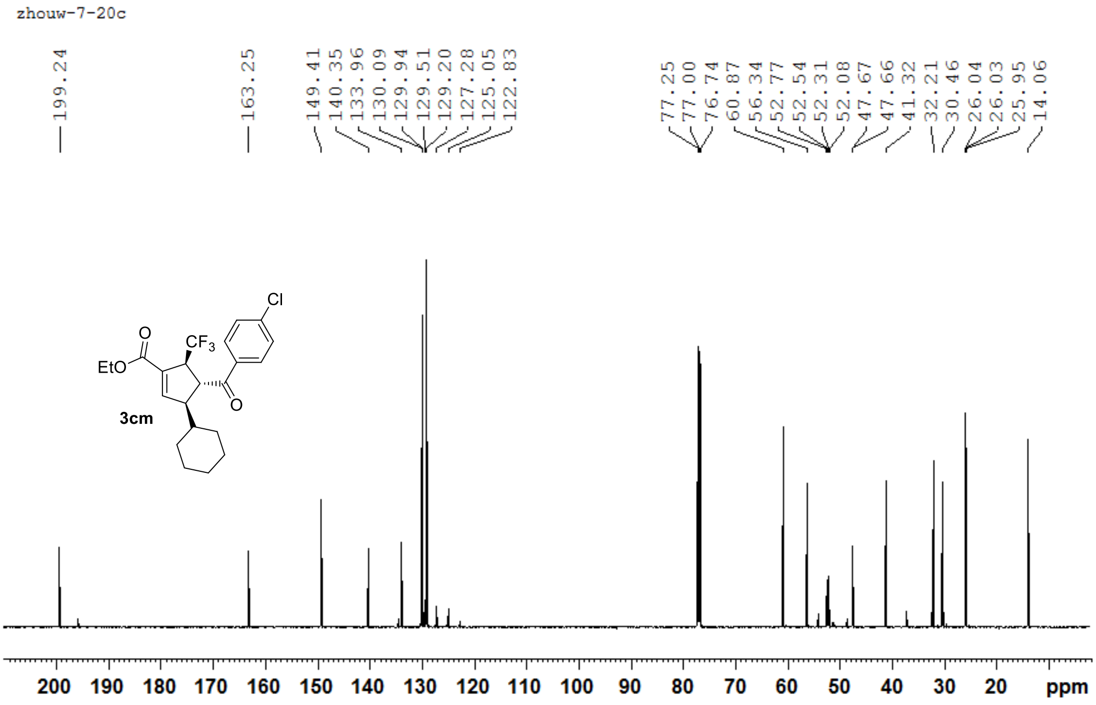
zhouw-7-20c
199.24
163.25
149.41
140.35
133.96
130.09
129.94
129.51
129.20
127.28
125.05
122.83
77.25
77.00
76.74
60.87
56.34
52.77
52.54
52.31
52.08
47.67
47.66
41.32
32.21
30.46
26.04
26.03
25.95
14.06
Cl
CF3
O
EtO
O
3cm
200 190 180 170 160 150 140 130 120 110 100 90 80 70 60 50 40 30 20 ppm

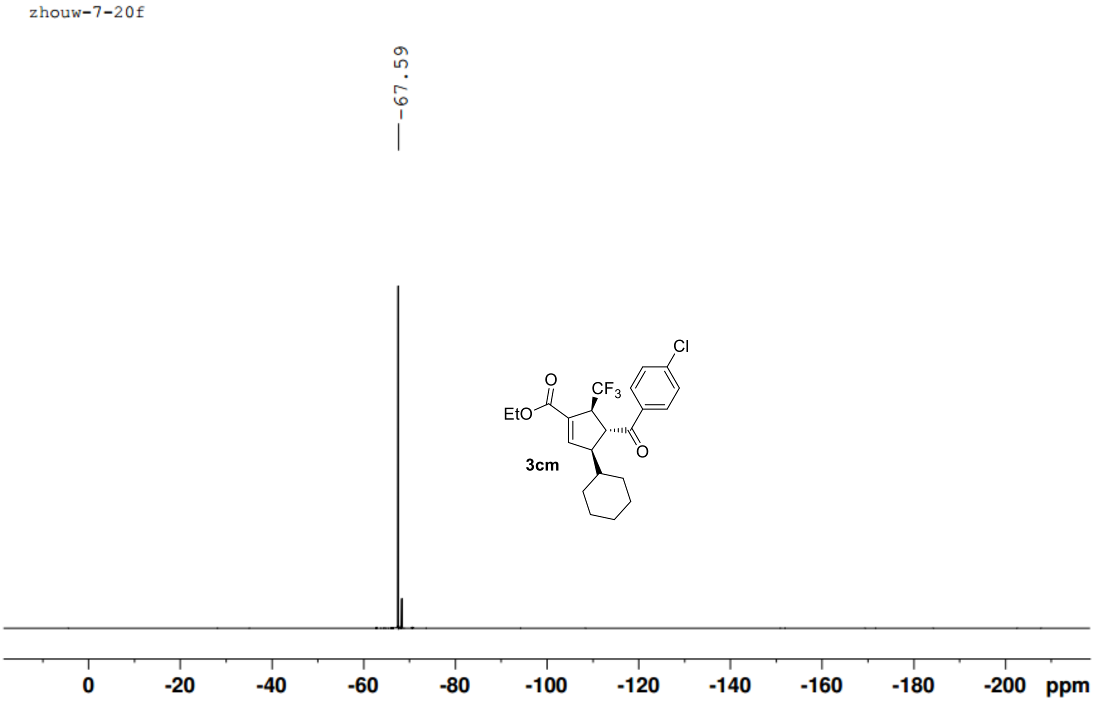
zhouw-7-20f
-67.59
Cl
CF3
O
EtO
O
3cm
0 -20 -40 -60 -80 -100 -120 -140 -160 -180 -200 ppm

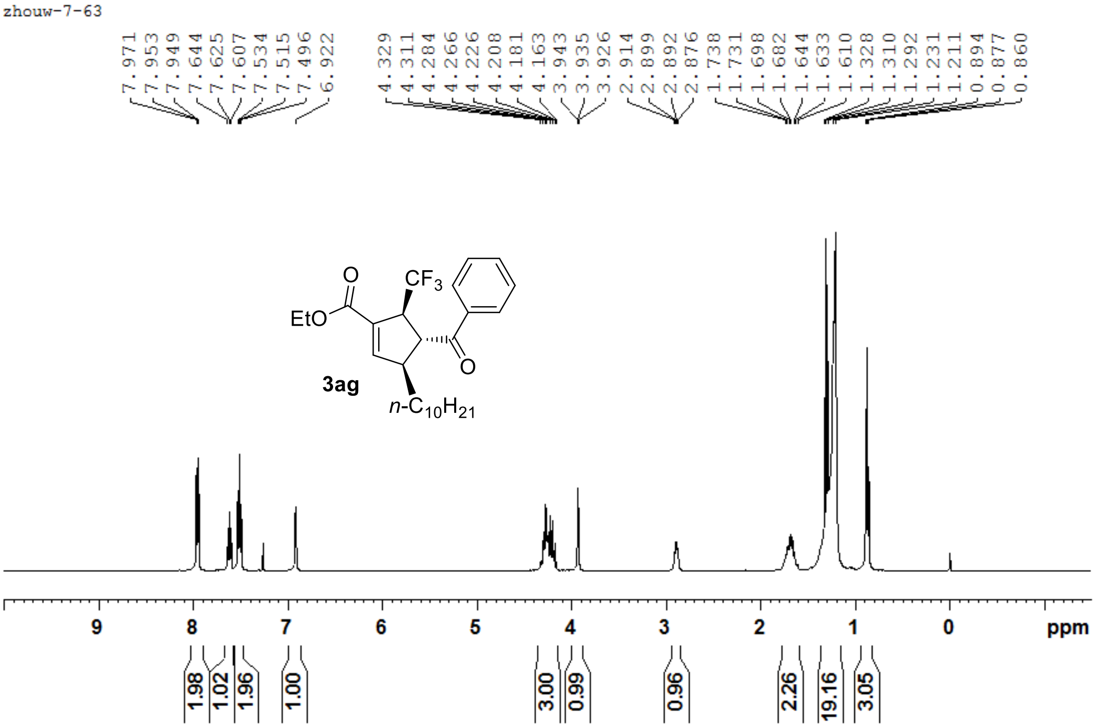
zhouw-7-63
7.971
7.953
7.949
7.644
7.625
7.607
7.534
7.515
7.496
6.922
4.329
4.311
4.284
4.266
4.226
4.208
4.181
4.163
3.943
3.935
3.926
2.914
2.899
2.892
2.876
1.738
1.731
1.698
1.682
1.644
1.633
1.610
1.328
1.310
1.292
1.231
1.211
0.894
0.877
0.860
EtO
O
CF3
O
3ag
n-C10H21
9
8
7
6
5
4
3
2
1
0
ppm
1.98
1.02
1.96
1.00
3.00
0.99
0.96
2.26
19.16
3.05

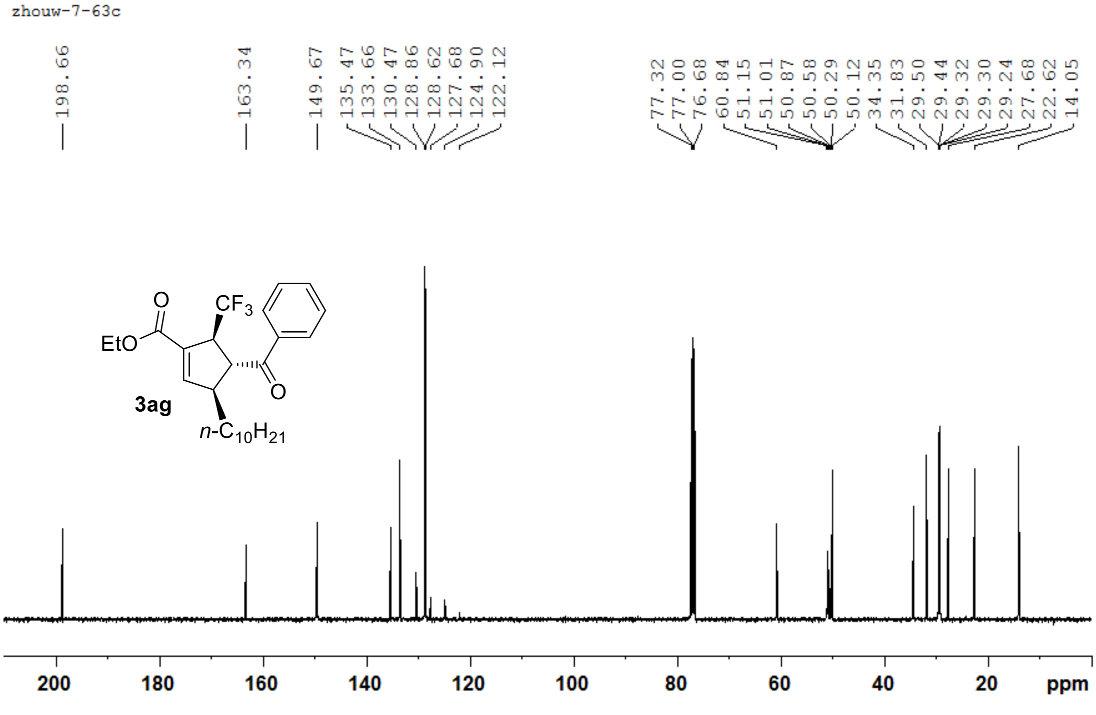
zhouw-7-63c
198.66
163.34
149.67
135.47
133.66
130.47
128.86
128.62
127.68
124.90
122.12
77.32
77.00
76.68
60.84
51.15
51.01
50.87
50.58
50.29
50.12
34.35
31.83
29.50
29.44
29.32
29.30
29.24
27.68
22.62
14.05
EtO
O
CF3
O
3ag
n-C10H21
200
180
160
140
120
100
80
60
40
20
ppm

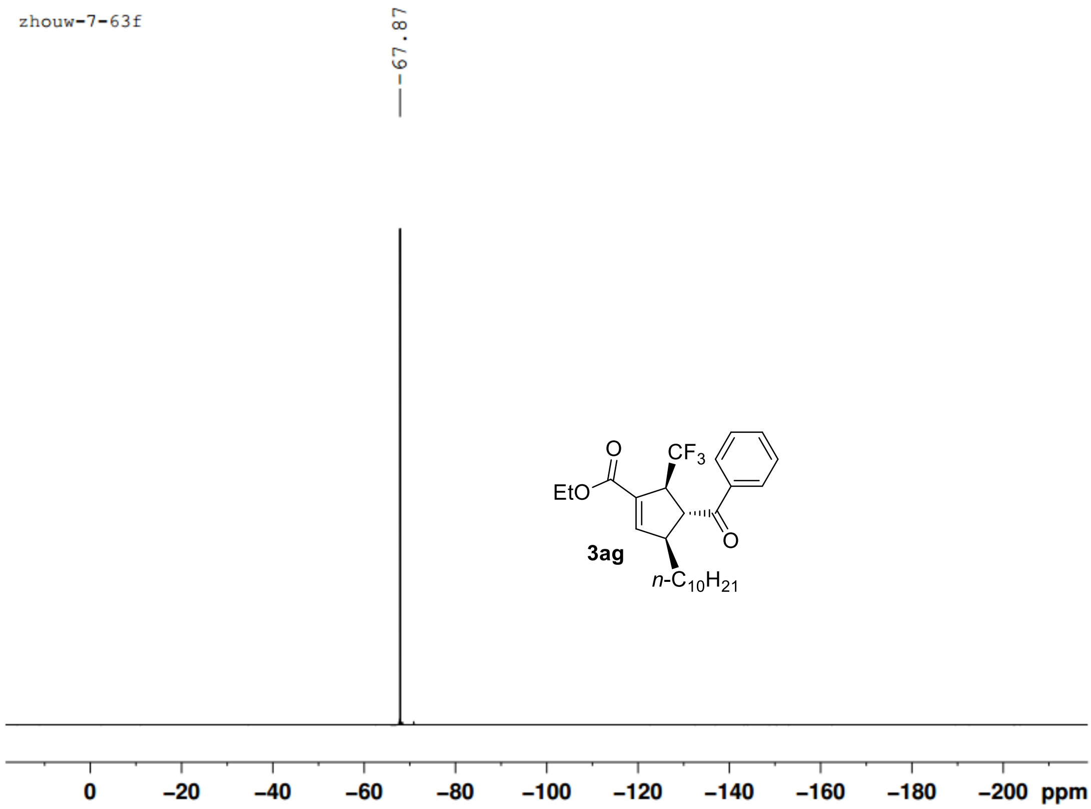
zhouw-7-63f
-67.87
3ag
EtO
CF3
n-C10H21
0 −20 −40 −60 −80 −100 −120 −140 −160 −180 −200 ppm

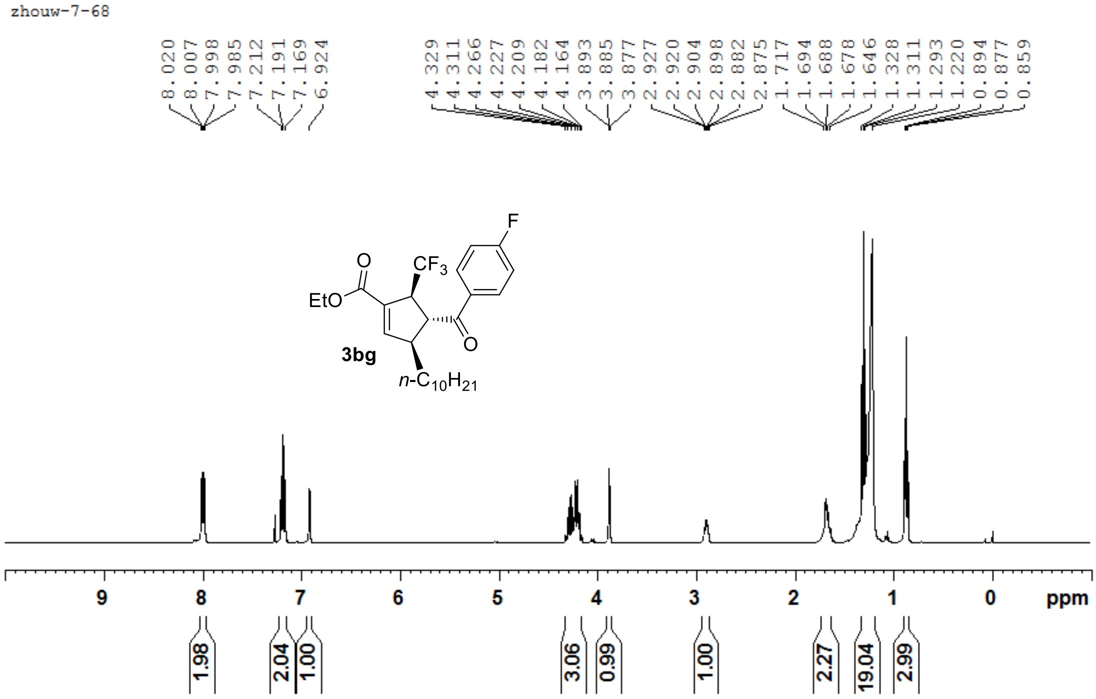
zhouw-7-68
8.020 8.007 7.998 7.985 7.212 7.191 7.169 6.924
4.329 4.311 4.266 4.227 4.209 4.182 4.164 3.893 3.885 3.877 2.927 2.920 2.904 2.898 2.882 2.875 1.717 1.694 1.688 1.678 1.646 1.328 1.311 1.293 1.220 0.894 0.877 0.859
3bg
EtO
CF3
F
n-C10H21
9 8 7 6 5 4 3 2 1 0 ppm
1.98 2.04 1.00 3.06 0.99 1.00 2.27 19.04 2.99

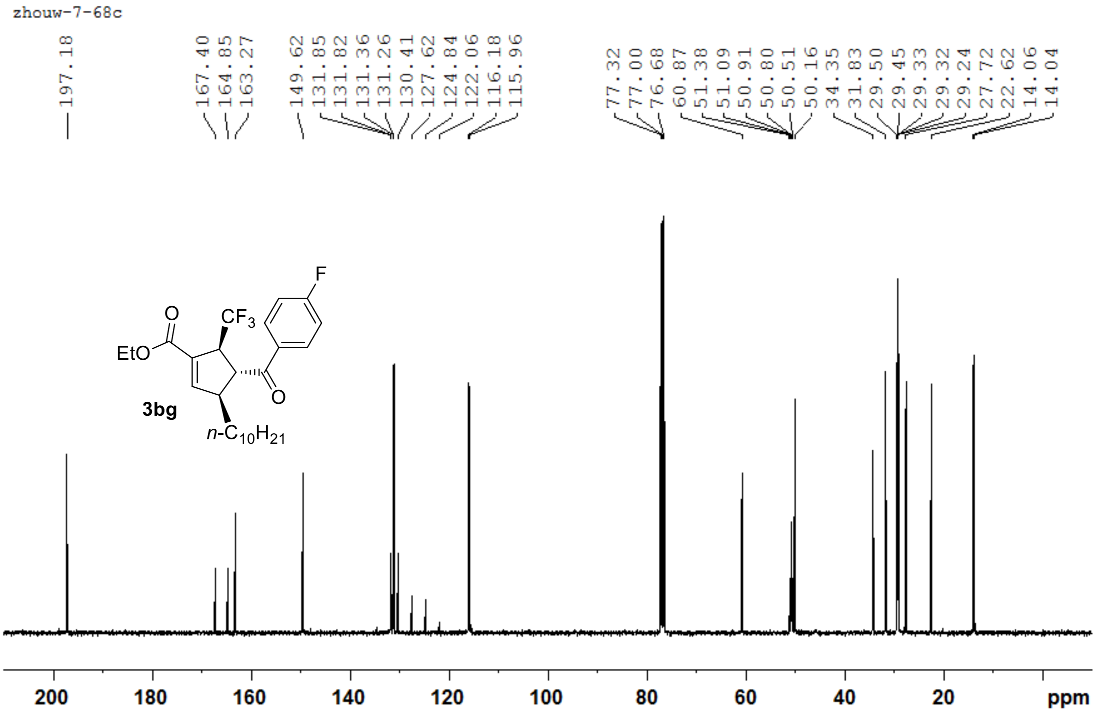
zhouw-7-68c
197.18
167.40
164.85
163.27
149.62
131.85
131.82
131.36
131.26
130.41
127.62
124.84
122.06
116.18
115.96
77.32
77.00
76.68
60.87
51.38
51.09
50.91
50.80
50.51
50.16
34.35
31.83
29.50
29.45
29.33
29.32
29.24
27.72
22.62
14.06
14.04
EtO
O
CF3
F
O
3bg
n-C10H21
200
180
160
140
120
100
80
60
40
20
ppm

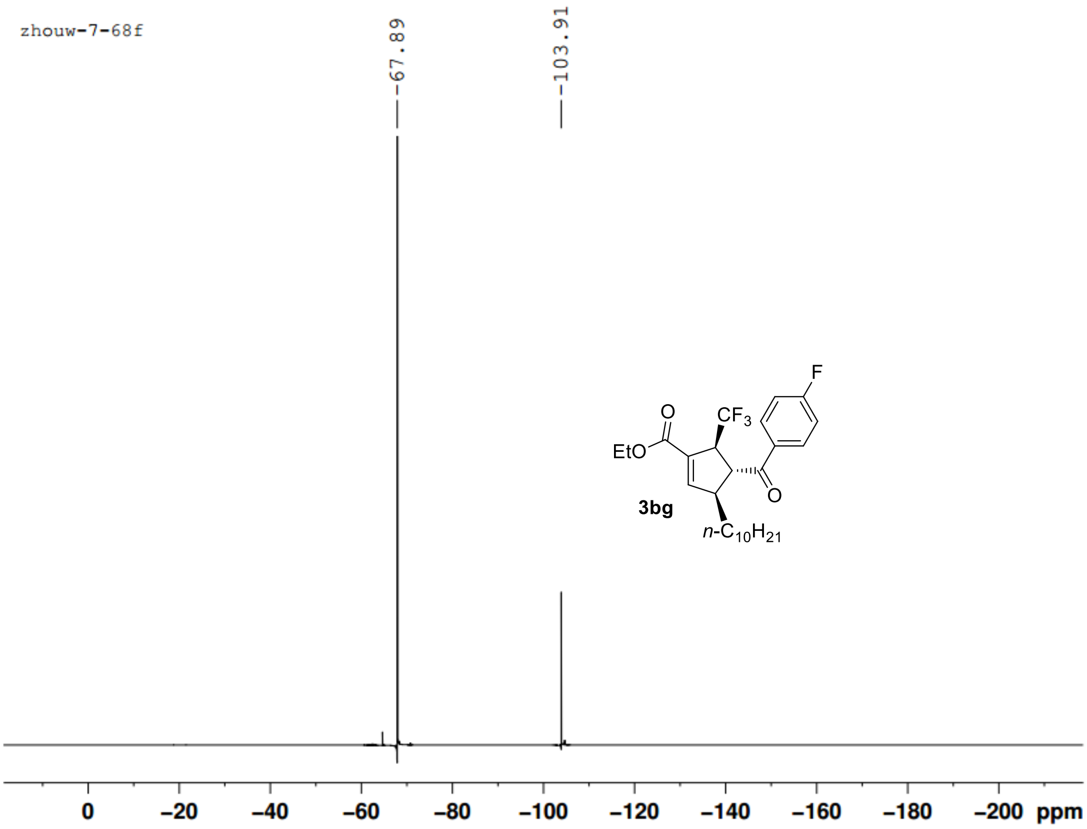
zhouw-7-68f
-67.89
-103.91
EtO
O
CF3
F
O
3bg
n-C10H21
0
−20
−40
−60
−80
−100
−120
−140
−160
−180
−200
ppm

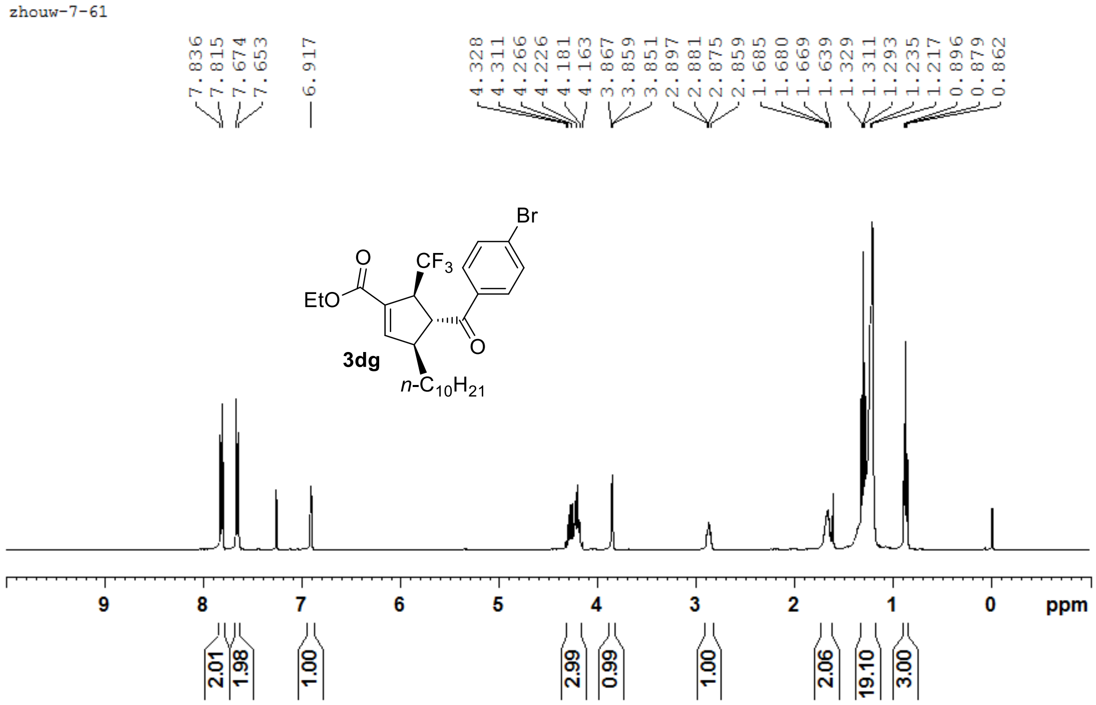
zhouw-7-61
7.836
7.815
7.674
7.653
6.917
4.328
4.311
4.266
4.226
4.181
4.163
3.867
3.859
3.851
2.897
2.881
2.875
2.859
1.685
1.680
1.669
1.639
1.329
1.311
1.293
1.235
1.217
0.896
0.879
0.862
Br
O
CF3
EtO
O
3dg
n-C10H21
9
8
7
6
5
4
3
2
1
0
ppm
2.01
1.98
1.00
2.99
0.99
1.00
2.06
19.10
3.00

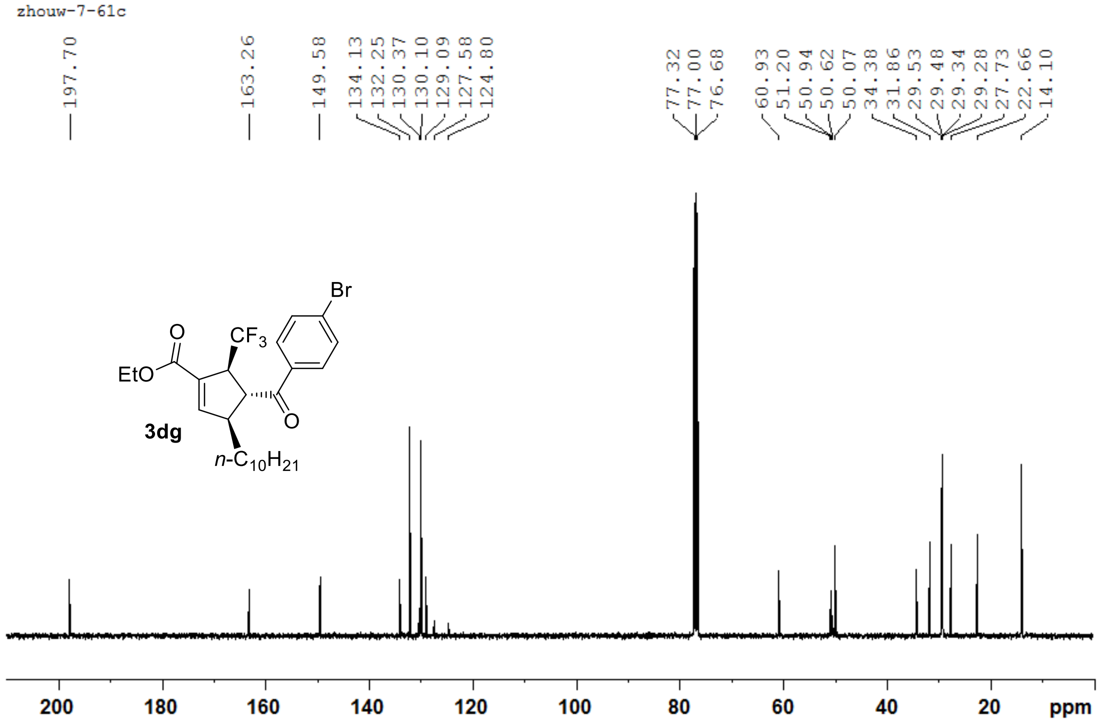
zhouw-7-61c
197.70
163.26
149.58
134.13
132.25
130.37
130.10
129.09
127.58
124.80
77.32
77.00
76.68
60.93
51.20
50.94
50.62
50.07
34.38
31.86
29.53
29.48
29.34
29.28
27.73
22.66
14.10
Br
O
CF3
EtO
O
3dg
n-C10H21
200
180
160
140
120
100
80
60
40
20
ppm

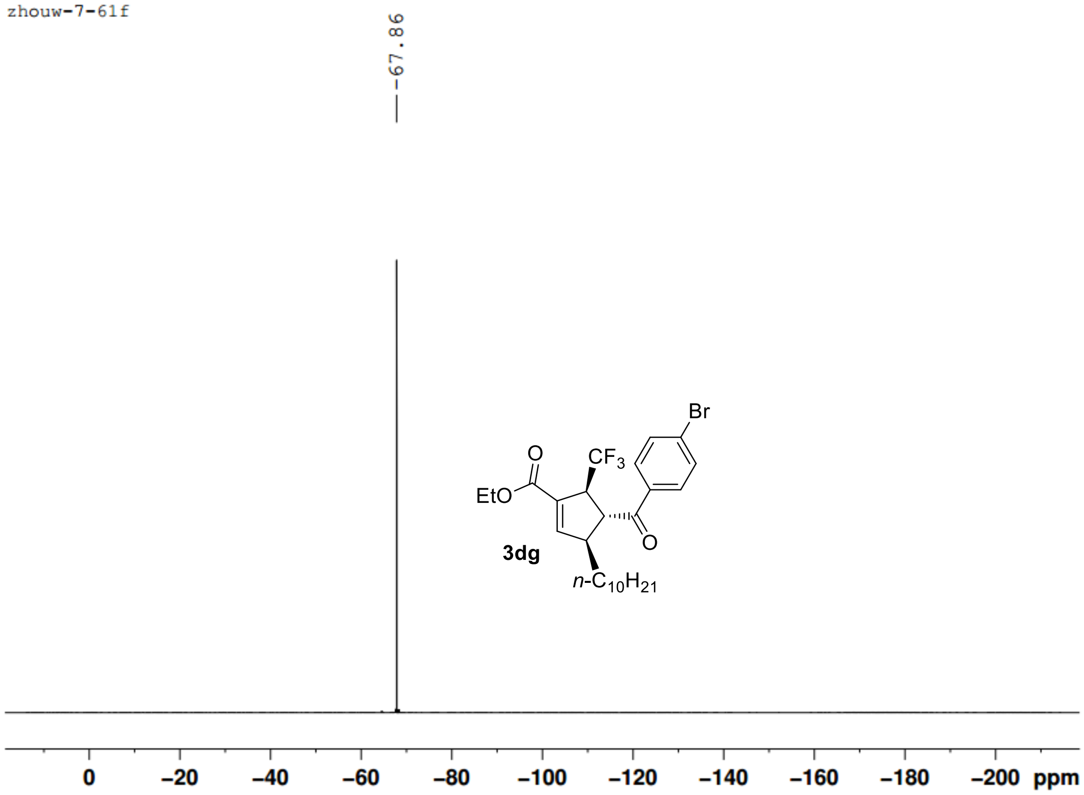
zhouw-7-61f
-67.86
Br
O
CF3
EtO
O
3dg
n-C10H21
0
-20
-40
-60
-80
-100
-120
-140
-160
-180
-200 ppm

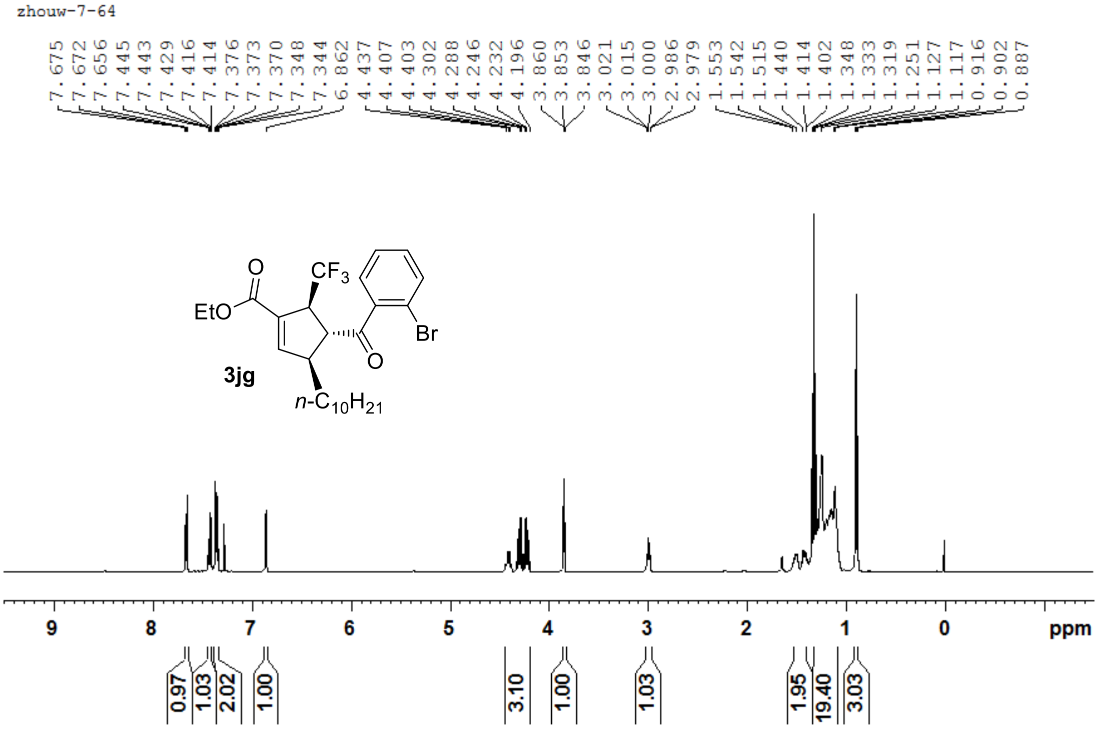
zhouw-7-64
7.675 7.672 7.656 7.445 7.443 7.429 7.416 7.414 7.376 7.373 7.370 7.348 7.344 6.862 4.437 4.407 4.403 4.302 4.288 4.246 4.232 4.196 3.860 3.853 3.846 3.021 3.015 3.000 2.986 2.979 1.553 1.542 1.515 1.440 1.414 1.402 1.348 1.333 1.319 1.251 1.127 1.117 0.916 0.902 0.887
O
CF3
EtO
Br
O
3jg
n-C10H21
9
8
7
6
5
4
3
2
1
0
ppm
0.97
1.03
2.02
1.00
3.10
1.00
1.03
1.95
19.40
3.03

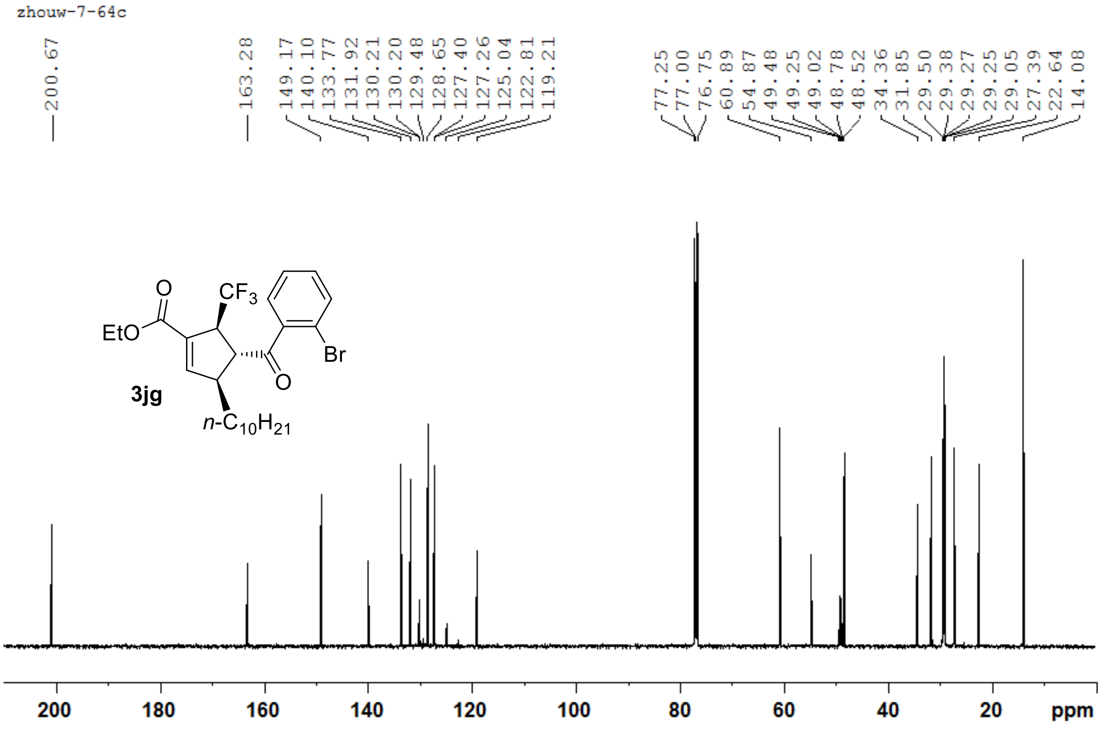
zhouw-7-64c
200.67
163.28
149.17
140.10
133.77
131.92
130.21
130.20
129.48
128.65
127.40
127.26
125.04
122.81
119.21
77.25
77.00
76.75
60.89
54.87
49.48
49.25
49.02
48.78
48.52
34.36
31.85
29.50
29.38
29.27
29.25
29.05
27.39
22.64
14.08
EtO
O
CF3
Br
O
3jg
n-C10H21
200
180
160
140
120
100
80
60
40
20
ppm

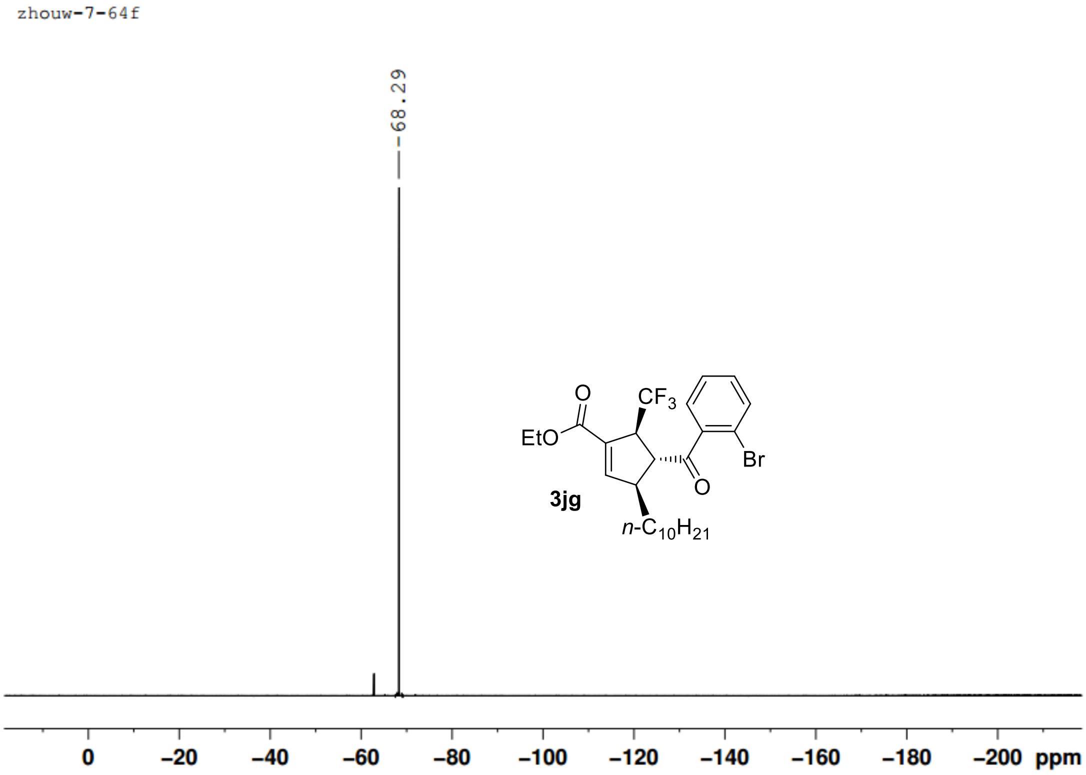
zhouw-7-64f
-68.29
EtO
O
CF3
Br
O
3jg
n-C10H21
0
–20
–40
–60
–80
–100
–120
–140
–160
–180
–200
ppm

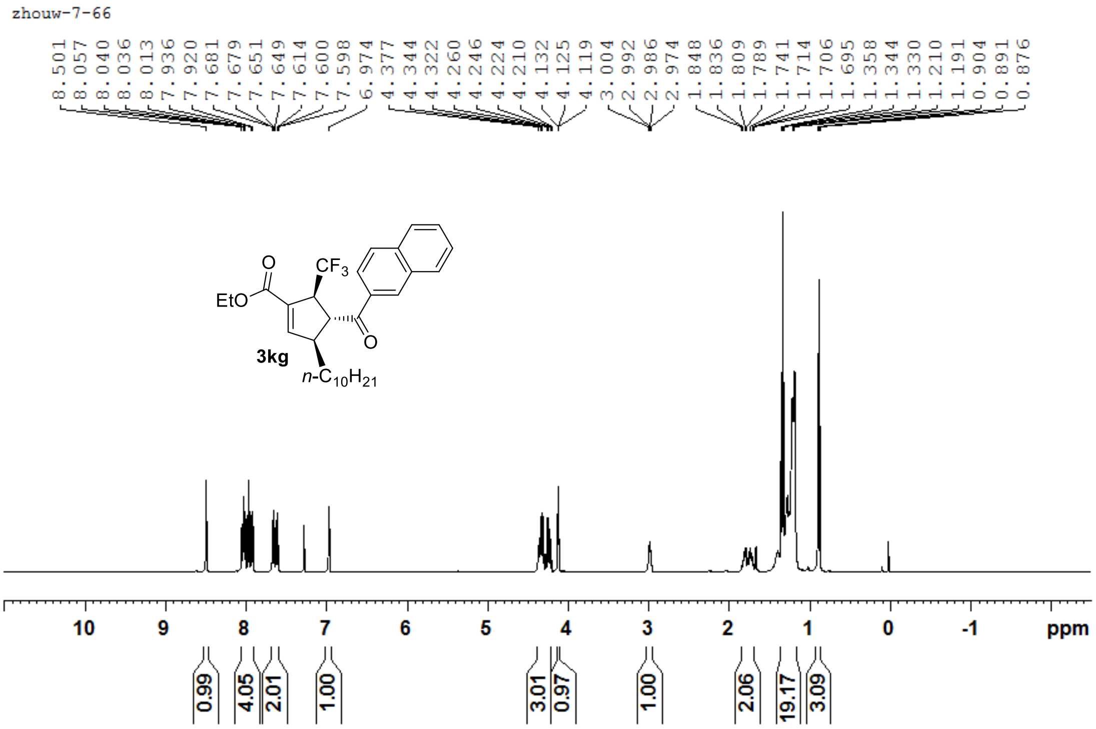
zhouw-7-66
8.501 8.057 8.040 8.036 8.013 7.936 7.920 7.681 7.679 7.651 7.649 7.614 7.600 7.598 6.974 4.377 4.344 4.322 4.260 4.246 4.224 4.210 4.132 4.125 4.119 3.004 2.992 2.986 2.974 1.848 1.836 1.809 1.789 1.741 1.714 1.706 1.695 1.358 1.344 1.330 1.210 1.191 0.904 0.891 0.876
EtO
O
CF3
O
3kg
n-C10H21
10 9 8 7 6 5 4 3 2 1 0 -1 ppm
0.99 4.05 2.01 1.00 3.01 0.97 1.00 2.06 19.17 3.09

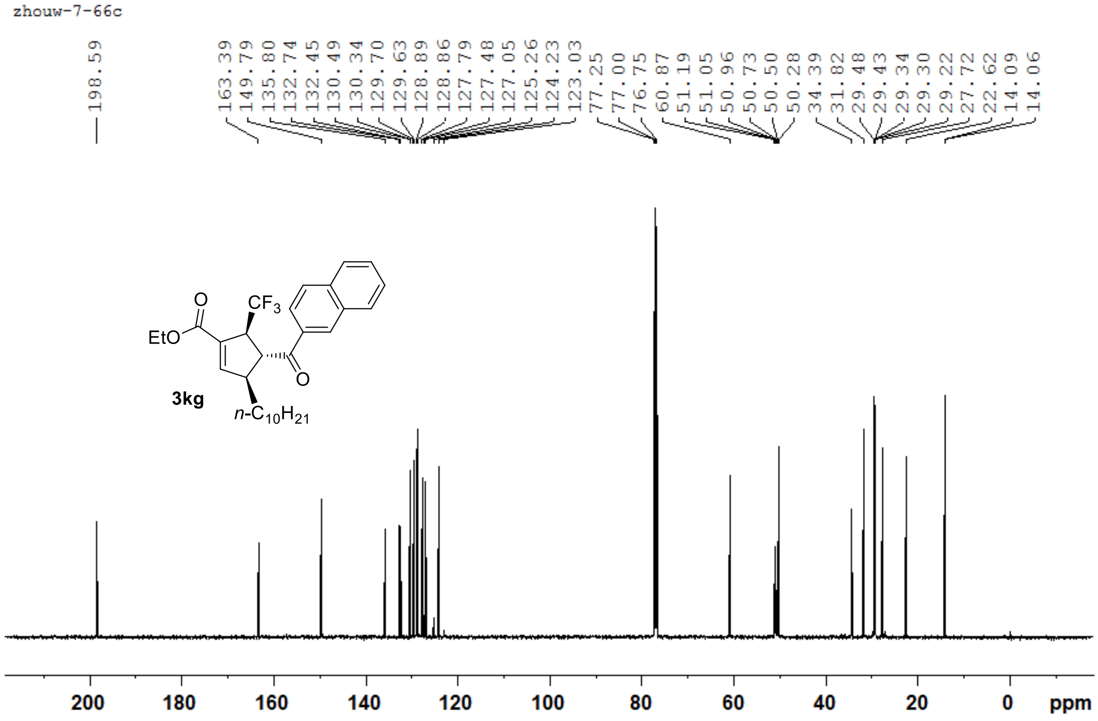
zhouw-7-66c
198.59
163.39 149.79 135.80 132.74 132.45 130.49 130.34 129.70 129.63 128.89 128.86 127.79 127.48 127.05 125.26 124.23 123.03 77.25 77.00 76.75 60.87 51.19 51.05 50.96 50.73 50.50 50.28 34.39 31.82 29.48 29.43 29.34 29.30 29.22 27.72 22.62 14.09 14.06
EtO
O
CF3
O
3kg
n-C10H21
200 180 160 140 120 100 80 60 40 20 0 ppm

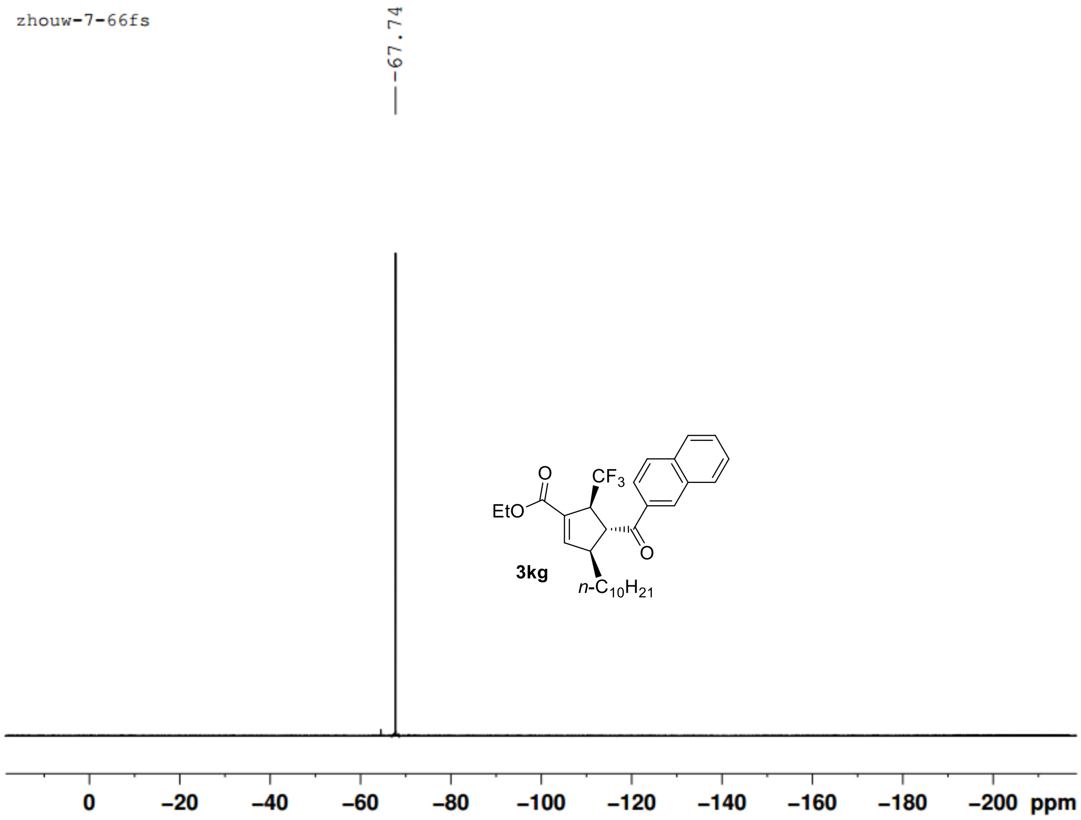
zhouw-7-66fs
-67.74
EtO
O
CF3
O
3kg
n-C10H21
0
−20
−40
−60
−80
−100
−120
−140
−160
−180
−200
ppm

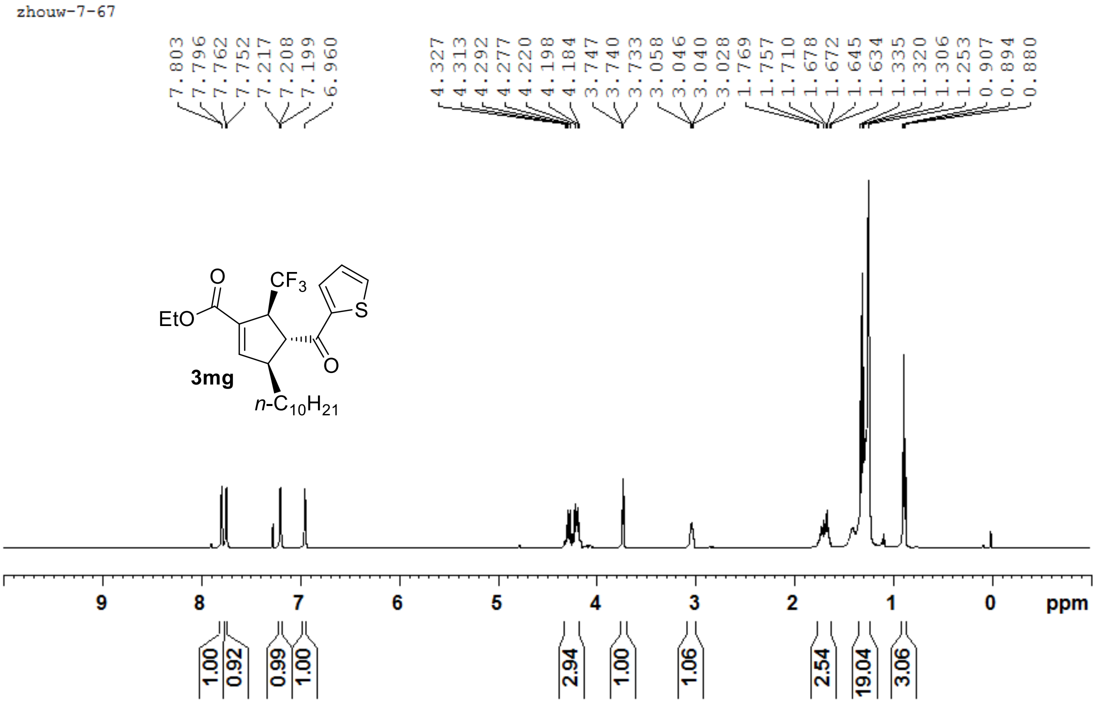
zhouw-7-67
7.803
7.796
7.762
7.752
7.217
7.208
7.199
6.960
4.327
4.313
4.292
4.277
4.220
4.198
4.184
3.747
3.740
3.733
3.058
3.046
3.040
3.028
1.769
1.757
1.710
1.678
1.672
1.645
1.634
1.335
1.320
1.306
1.253
0.907
0.894
0.880
EtO
O
CF3
S
O
3mg
n-C10H21
9
8
7
6
5
4
3
2
1
0
ppm
1.00
0.92
0.99
1.00
2.94
1.00
1.06
2.54
19.04
3.06

zhouw-7-67c

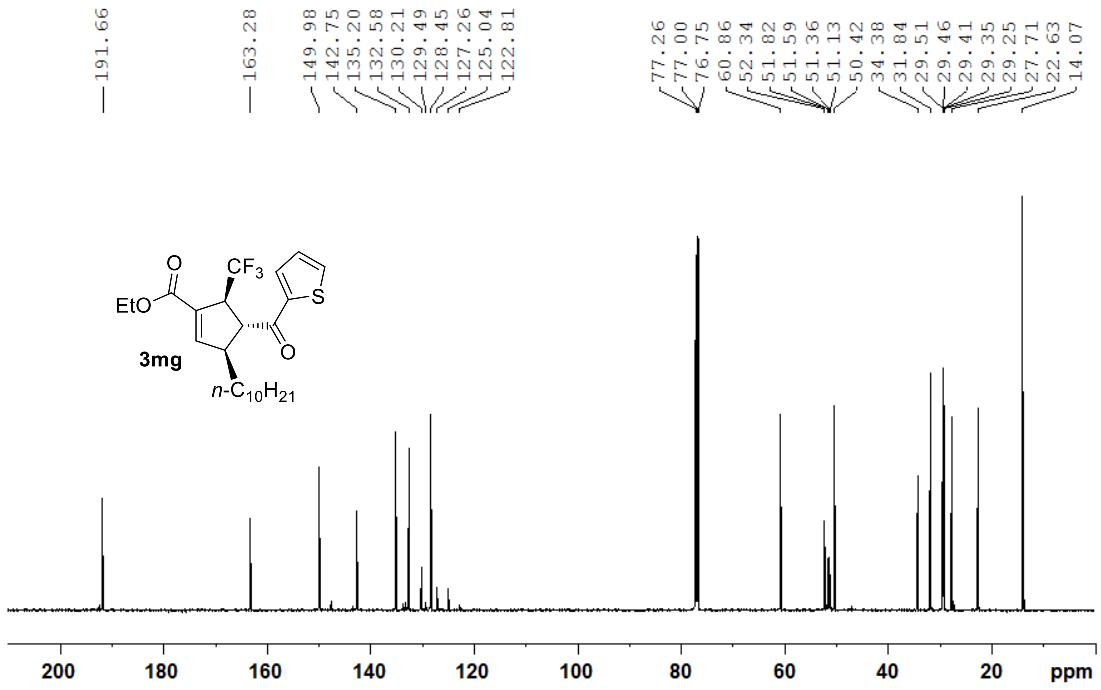

zhouw-7-67f

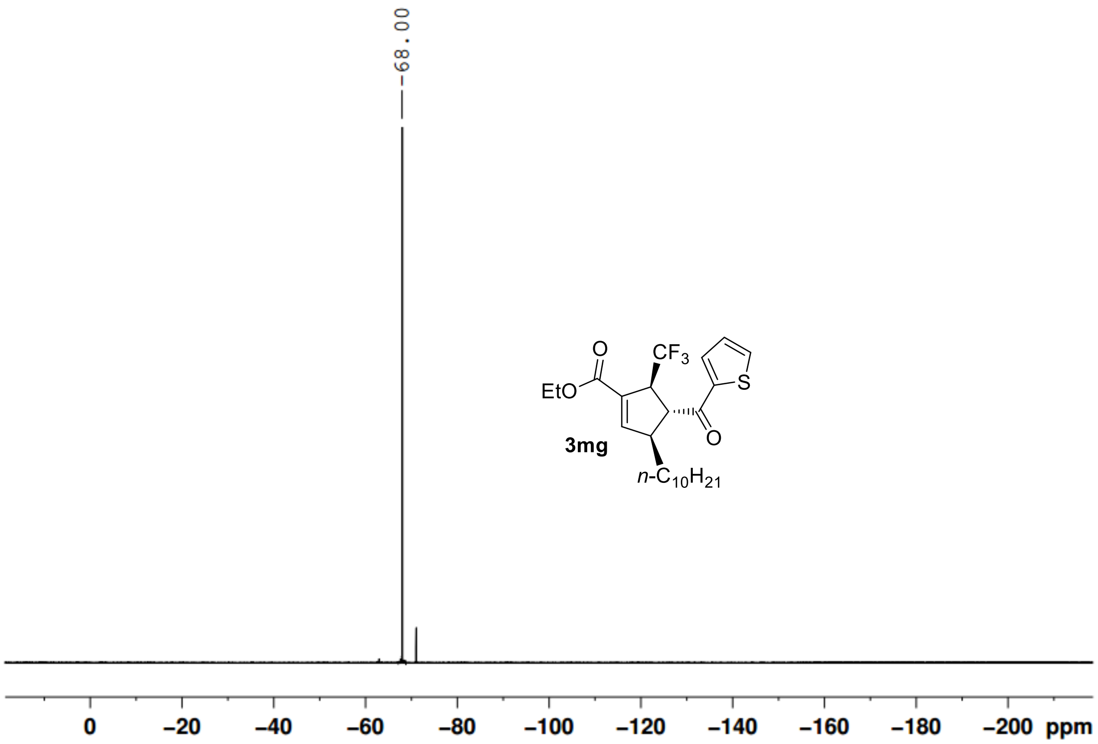

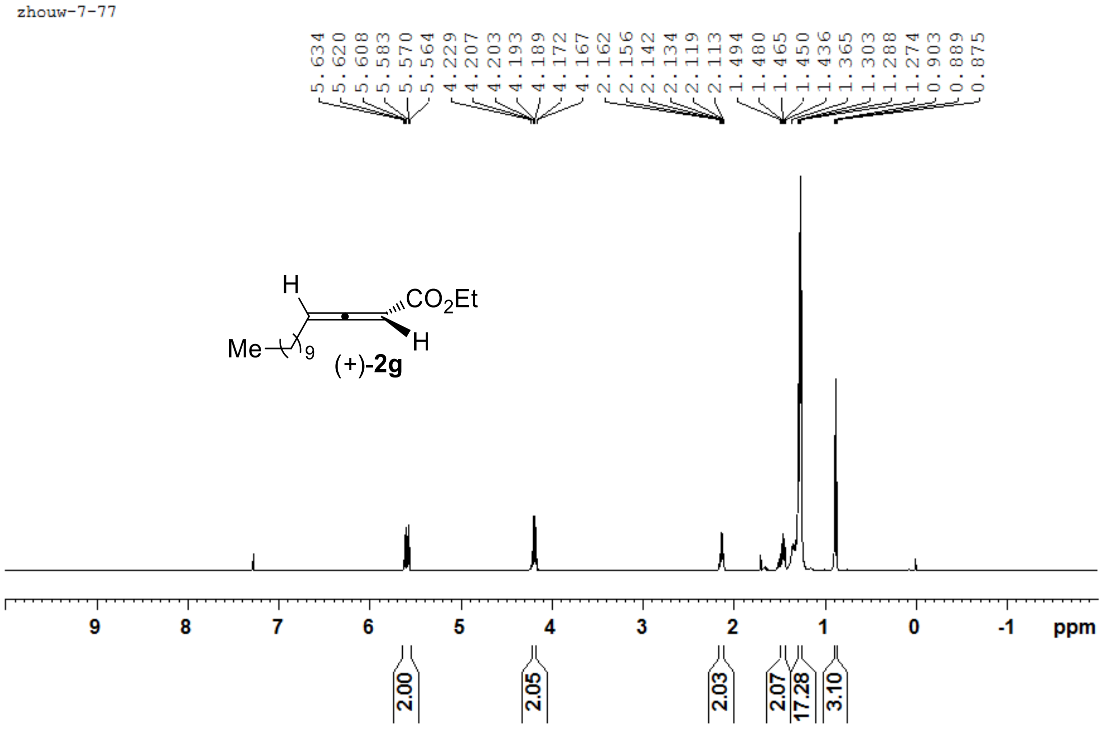
zhouw-7-77
5.634
5.620
5.608
5.583
5.570
5.564
4.229
4.207
4.203
4.193
4.189
4.172
4.167
2.162
2.156
2.142
2.134
2.119
2.113
1.494
1.480
1.465
1.450
1.436
1.365
1.303
1.288
1.274
0.903
0.889
0.875
H
CO2Et
H
Me
9
(+)-2g
9 8 7 6 5 4 3 2 1 0 -1 ppm
2.00
2.05
2.03
2.07
17.28
3.10

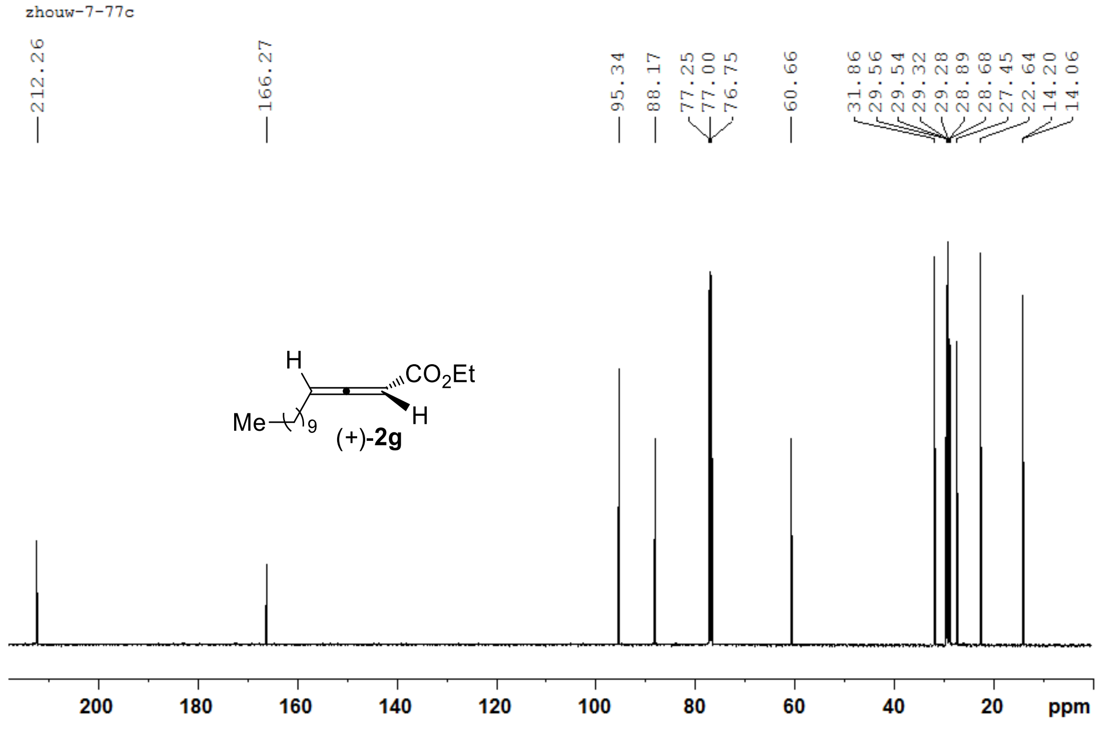
zhouw-7-77c
212.26
166.27
95.34
88.17
77.25
77.00
76.75
60.66
31.86
29.56
29.54
29.32
29.28
28.89
28.68
27.45
22.64
14.20
14.06
H
CO2Et
H
Me
9
(+)-2g
200 180 160 140 120 100 80 60 40 20 ppm
